# Seed size, endosperm and germination variation in sexual and apomictic *Boechera*

**DOI:** 10.3389/fpls.2022.991531

**Published:** 2022-11-18

**Authors:** Dorota Paczesniak, Marco Pellino, Richard Goertzen, Devan Guenter, Siegfried Jahnke, Andreas Fischbach, John T. Lovell, Timothy F. Sharbel

**Affiliations:** ^1^ Global Institute for Food Security (GIFS), University of Saskatchewan, Saskatoon, SK, Canada; ^2^ Breeding Research, Leibniz Institute of Plant Genetics and Crop Plant Research (IPK), Gatersleben, Germany; ^3^ Forschungszentrum Jülich, Institute of Bio- and Geosciences, IBG-2: Plant Sciences, Jülich, Germany

**Keywords:** apomixis, seed size, *Boechera*, endosperm, reproduction, Brassicaceae, life history traits, fitness

## Abstract

Asexual reproduction results in offspring that are genetically identical to the mother. Among apomictic plants (reproducing asexually through seeds) many require paternal genetic contribution for proper endosperm development (pseudogamous endosperm). We examined phenotypic diversity in seed traits using a diverse panel of sexual and apomictic accessions from the genus *Boechera*. While genetic uniformity resulting from asexual reproduction is expected to reduce phenotypic diversity in seeds produced by apomictic individuals, pseudogamous endosperm, variable endosperm ploidy, and the deviations from 2:1 maternal:paternal genome ratio in endosperm can all contribute to increased phenotypic diversity among apomictic offspring. We characterized seed size variation in 64 diploid sexual and apomictic (diploid and triploid) *Boechera* lineages. In order to find out whether individual seed size was related to endosperm ploidy we performed individual seed measurements (projected area and mass) using the *phenoSeeder* robot system and flow cytometric seed screen. In order to test whether individual seed size had an effect on resulting fitness we performed a controlled growth experiment and recorded seedling life history traits (germination success, germination timing, and root growth rate). Seeds with triploid embryos were 33% larger than those with diploid embryos, but no average size difference was found between sexual and apomictic groups. We identified a maternal effect whereby chloroplast lineage 2 had 30% larger seeds than lineage 3, despite having broad and mostly overlapping geographic ranges. Apomictic seeds were not more uniform in size than sexual seeds, despite genetic uniformity of the maternal gametophyte in the former. Among specific embryo/endosperm ploidy combinations, seeds with tetraploid (automomous) endosperm were on average smaller, and the proportion of such seeds was highest in apomicts. Larger seeds germinated more quickly than small seeds, and lead to higher rates of root growth in young seedlings. Seed mass is under balancing selection in *Boechera*, and it is an important predictor of several traits, including germination probability and timing, root growth rates, and developmental abnormalities in apomictic accessions.

## 1 Introduction

Offspring produced through asexual reproduction are genetically identical to the mother and to each other. Asexual reproduction through seeds in plants (apomixis) has been of wide interest from an applied perspective because of the potential to propagate and maintain desirable genotypes clonally (Navashin, 1933; Karpechenko, 1935; Jefferson, 1994; Thoenissen, 2001; Spillane et al., 2004). This idea to engineer apomixis in crop plants is based on the premise that genetic uniformity brought about by asexual reproduction will result in uniformity of phenotypic traits. An important underlying assumption - which requires empirical evaluation - is that the traits of interest are heritable, and have low levels of variation due to non-genetic factors.

Seed size variability in sexually-reproducing plants is widespread and common, and typically intra individual variation is greater than variation between individuals or populations (Thompson, 1984; Michaels et al., 1988; Obeso, 1993; Vaughton and Ramsey, 1998). Many fitness-related traits have been found to be associated with species- and/or individual level variation in seed size in flowering plants, including: probability and timing of germination and emergence (Dolan, 1984; Hendrix, 1984; Giles, 1990; Susko and Lovett-Doust, 2000; Benard and Toft, 2007; Harrison et al., 2007; Münzbergová and Plačková, 2010; Leverett and Jolls, 2014), reproductive output (Stanton, 1985; Giles, 1990), seedling growth rate (Giles, 1990; Eriksson, 1999; Susko and Lovett-Doust, 2000), survival (Simons and Johnston, 2000; Baraloto et al., 2005), competitive advantage (Wulff, 1986; Eriksson, 1999; Susko and Cavers, 2008), and plant size and probability of flowering through second year (Halpern, 2005). However, even if the increased size of an individual seed confers selective advantage in the offspring, taking into account the cost of maternal resources needed to build the seed can result in net zero advantage relative to seed size (Larios et al., 2014; Larios and Venable, 2018).

In apomictic *Taraxacum* species, considerable within-individual variation in seed mass (specifically achene, i.e. seed-containing fruit) was also found, and the probability of germination increased with seed mass (Tweney and Mogie, 1999). In another apomictic species, *Microlicia fasciculata*, coefficient of variation in germination time of seeds from individual mother plants was on average 15.9% (Ranal et al., 2016), but the relationship between seed size and other traits was not directly evaluated. To date, there are no direct comparisons of the amount of variability in seed size and its life history effects in apomictic and closely-related sexual species.

Seed heteromorphism may be adaptive, especially in heterogeneous environments (Westoby, 1981; Venable, 1985), and has been implicated in the success of invasive plant species (Ortmans et al., 2016; Molina-Montenegro et al., 2018). Alternatively, variation in seed size is the result of plant architecture and physiological constraints, e.g. position effects in inflorescence or fruit (McGinley et al., 1987; Giles, 1990; Wolfe, 1992), or a product of kin conflict (Trivers, 1974; Haig and Westoby, 1989).

Imprinted maternally- and paternally-derived alleles of genes responsible for transfer of resources to developing seeds are expected to have opposite effects on endosperm growth (Haig and Westoby, 1991; Haig, 2013), with the effects of matrigenic and patrigenic expression to respectively reduce and increase seed size (Scott et al., 1998; Leblanc et al., 2002; Pennington et al., 2008; Li and Dickinson, 2010). The conflict of interest over resource allocation is expected to be more pronounced when offspring are less closely related to each other, e.g. in outcrossing rather than selfing populations (De Jong et al., 2005), which empirical studies have confirmed (Willi, 2013; Raunsgard et al., 2018). Most apomictic species evolved from sexual ancestors (Bell, 1982), but the requirement to produce endosperm through a sexual process is often retained (pseudogamous apomixis, Nogler, 1984). This could be because the role of patrigenic expression for proper endosperm function is a constraint for the evolution of autonomous (i.e. derived from an unfertilized central cell) endosperm.

The genus *Boechera* Á. Löve & D. Löve (formerly *Arabis* L.; Brassicaceae) presents a model system in which the impacts of reproductive mode (sexual vs. apomictic) and ploidy level can be studied separately, because of the presence of diploid sexual as well as diploid and polyploid (predominantly triploid) apomictic forms (Böcher, 1951; Al-Shehbaz and Windham, 2010). The presence of apomictic diploids is exceptional among apomictic taxa (reviewed in: Beck et al., 2012). The genus is native to North America, with a wide range spanning from northern Mexico to Canada and Greenland (Al-Shehbaz and Windham, 2010). Sexual species are predominantly selfing (Roy, 1995; Schranz et al., 2005; Beck et al., 2012), but occasionally hybridize (Dobes et al., 2004; Kantama et al., 2007; Beck et al., 2012), which has led to formation of multiple independent apomictic lineages (Koch et al., 1999; Koch et al., 2003; Kiefer et al., 2009; Mau et al., 2015).

Notably, there is reproductive variation in seed development, with some sexual *Boechera* producing low levels (<3%) of apomictic seeds, while some apomictic lineages produce up to 13% of sexual seeds (Aliyu et al., 2010). Sexual seeds are typical for angiosperms, with diploid mother plants producing a seed composed of a diploid embryo and a triploid (3x) endosperm, the latter composed of 2 maternal and 1 paternal genomes (2m:1p). Importantly, this parental genome ratio in endosperm is also the most prevalent among apomictic seeds, despite differing ploidy (2x embryo: 6x [4m:2p] endosperm; 3x embryo: 9x [6m:3p] endosperm, Aliyu et al., 2010; *sensu* “balanced apomicts” Mau et al., 2021). However, deviations from the 2:1 maternal to paternal genome ratio in endosperm are also present. Firstly, there are diploid apomictic lineages with predominantly (>85%) pentaploid endosperm (2x embryo: 5x [4m:1p] endosperm; Aliyu et al., 2010; sensu “unbalanced apomicts” Lovell et al., 2013; Mau et al., 2021). Secondly, a wide range of variation in endosperm ploidy (between 4x and 12x) was found at small frequencies, including both male- and female-biased ploidy ratios (Aliyu et al., 2010; Voigt-Zielinski et al., 2012).

Thus, despite asexual (clonal) generation of the embryo, apomictic seed development in *Boechera* is characterized by variation in endosperm ploidy together with diversity in maternal to paternal genome ratios. In order to understand the effects of this variation on seed size, we surveyed seed size variation in a diverse set of sexual and apomictic lineages. Using multiparametric measurements of individual seeds in conjunction with flow cytometry, we investigated the relationship between seed size and endosperm ploidy variation. Size-dependent selection coefficients were subsequently contrasted between sexual and apomictic seedlings in a controlled growth experiment in which the effects of seed size on multiple life history traits were estimated.

## 2 Materials and methods

### 2.1 Plant material

In this study we used a total of 64 *Boechera* accessions, i.e. plants descended from a single seed of wild collected plants (**Supplementary table 1**) from a seed collection in the Sharbel laboratory. The same accessions have been used in previous studies in which reproductive mode (apomictic vs. sexual) were distinguished by flow cytometric seed screening (FCSS, Aliyu et al., 2010; Mau et al., 2015). For two accessions (ES744 and TS78) the FCSS in this study (see *Methods: Flow cytometric seed screen*) did not confirm previously assigned reproductive mode, therefore we excluded these accessions from further analyses.

**Figure 1:**
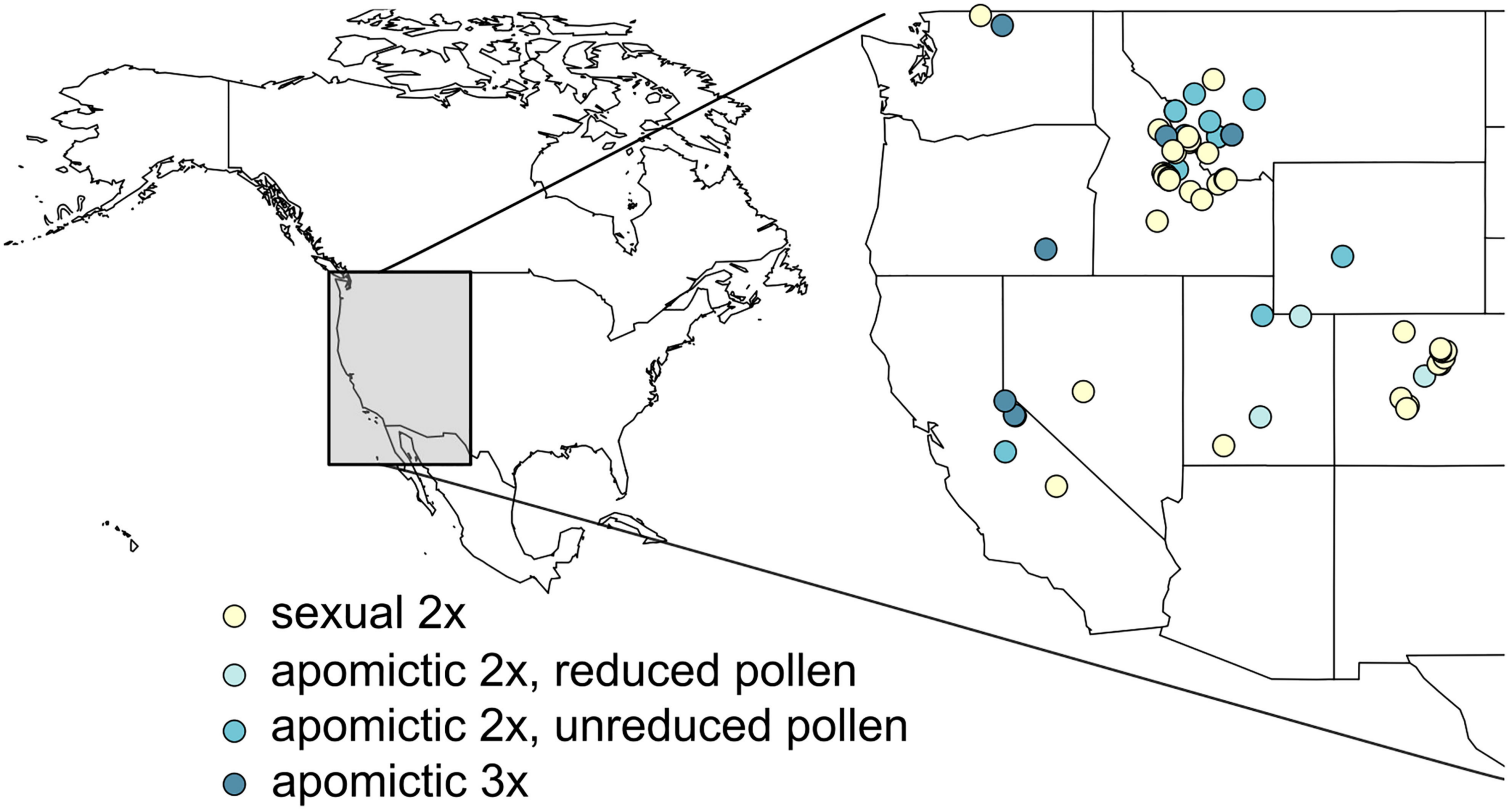
Locations from which 62 *Boechera* accessions used in this study were originally collected. Diploid sexual, two kinds of diploid apomictic (with reduced and unreduced pollen; see Aliyu et al., 2010; Mau et al., 2021), and triploid apomictic accessions are denoted by different colours.

Accessions were originally collected across western United States (**Figure 1**, **Supplementary table 1**). They belong to 13 (plus one unidentified) of the currently recognized ≥109 *Boechera* taxa (Al-Shehbaz and Windham, 2010), and represent the major three (I, II and III) of 7 chloroplast haplotype lineages (Kiefer et al., 2009). Two groups of apomicts were used, those which produce a majority of meiotically-reduced (x) and unreduced (2x) pollen (see Aliyu et al., 2010; Mau et al., 2021). Accessions for which chloroplast haplotypes were not characterized in Schranz et al. (2005) were sequenced as in Kiefer et al. (2009). We extracted DNA from leaf samples snap-frozen in liquid nitrogen using DNeasy^®^ Plant Mini Kit (QIAGEN Inc.) following the manufacturer’s protocol, or following the DNA extraction protocol from Mau et al. (2021). We amplified cp-DNA fragments following methods of Kiefer et al. (2009) using GoTaq^®^ Green Master Mix (Promega) or 2X Taq FroggaMix (FroggaBio). The amplicons were extracted from agarose gels and purified using GeneJET Gel Extraction Kit (Thermo Fisher Scientific) and sequenced using ABI3730XL DNA Analyzer (Thermo Fisher Scientific) at the National Research Council Canada sequencing facility in Saskatoon. The newly obtained haplotypes were then matched to the existing dataset (Kiefer et al., 2009) after correcting minor erroneous misalignments in the published alignment (Christiane Kiefer, personal communication). We used TCS v 1.21 (Clement et al., 2000) with fixed connection limit at 6 steps and gaps treated as 5th state, with an exception of accessions TS195 and TS218 which contained a large (107 bp) deletion that was not found in previously described haplotypes. In order to connect these two haplotypes to the existing haplotype network we used a setting where gaps were treated as missing data (**Supplementary table 1**, **Supplementary table 2**).

Plants were grown in common garden conditions (18°C, 16 h light/14°C, 8 h dark), at the University of Saskatchewan greenhouse facility starting in January 2016. All flowers were isolated (i.e. bagged) when flowering began to ensure self-fertilization. The onset of flowering varied widely (between February and November 2016), and the seeds were harvested when siliques began to desiccate (between May 2016 and January 2017).

### 2.2 Scans of bulk seed samples; seed size, seed size variation

We measured seed size from 3 individual plants each for 62 accessions (diploid sexual *n*=38, diploid apomictic *n*=14, triploid apomictic *n*=9) using a flatbed scanner (Epson Expression 11000XL, resolution 800 dpi). We used image analysis to determine seed area: seed particles were detected on white background based on colour filtering and projected area recorded using ImageJ software (Schneider et al., 2012). *Boechera* seeds on a flat surface always lay in the same orientation, therefore projected area is a good proxy for seed size. We manually checked the images for erroneous measurements and excluded multiple touching seeds that were measured as one particle or small debris. This procedure was repeated twice, in descending and ascending order, leaving 121272 seeds for analysis (on average *n*=652 seeds per individual, SD=242.1).

We used linear mixed-effect models to investigate the effects of four fixed factors on average seed size: ploidy level (diploid, triploid), reproductive mode (sexual, apomictic), species identity (13 species plus one unidentified) and chloroplast lineage (3 lineages). We used accession (62 accessions) and individual (186 individuals, nested under accession) as random factors. We used likelihood ratio tests to compare the fit of the full model to alternative models from which we removed the fixed factors one by one, in order to find a model which best explains the data.

We used coefficient of variation as the measure of seed size variability. In order to test whether reproductive mode (sexual, apomictic) and ploidy level (triploid, diploid) affected seed size variability (CV) we fit generalized linear mixed-effects models with gamma-distributed errors and a log-link function, using accession (62 accessions) as a random factor. We achieved better model fits with gamma-distributed errors than with normal distribution. We then used likelihood ratio tests to compare the fit of the full model to alternative models from which we removed the fixed factors one by one, in order to find a model which best explains the data.

### 2.3 Individual seed phenotyping with *phenoSeeder*

The *phenoSeeder* robot system, which was developed for handling and phenotyping individual seeds (Jahnke et al., 2016), was used to measure projected two-dimensional seed area and seed mass for 31 of the 64 *Boechera* accessions. We phenotyped 96 seeds (1 plate) from one individual per accession, which were later analyzed using flow cytometry (see below). For 25 of these 31 accessions we phenotyped a total of 3 individuals (3 plates, 96 seeds each) for use in the controlled growth experiment (see below) whereby one of the three individuals was the same as used for flow cytometry.

In total we had 106 96-well plates. We inspected all plates, and excluded from the analysis wells that were empty or had multiple seeds. This left on average 78.3 seeds per plate (SD=9.9), with a total *n*=8303 seeds (for flow cytometry: *n* =2380 seeds, 1 plate each for 31 accessions; for growth experiment N=5923 seeds, 3 individual plates for each of 25 accessions). Values of mass ≤ 0 for some seeds (71 observations) indicated an error with balance tarring, and in some cases the untarred balance reading could also be slightly above zero. Thus based on the distribution of mass values we identified a cut-off point for erroneous values around zero as the local minimum of the density function at 8.64 ng (**Supplementary figure 1**). We excluded values below 8.64 ng (total 97 observations) from further analyses, which left 8206 seeds for which we had a measurement of mass.

For the seed area measurements there were 630 cases of missing data, 787 cases of failed measurement, and 3 values of zero (total 1420 values). One reason for the relatively high proportion (7.6%) of missing data is because the images from 5 plates were not saved by accident, and thus seed area could not be reconstructed.

Visual inspection of the relationship between seed mass and seed area (**Supplementary figure 2**) identified some extreme off-diagonal outliers. Such outliers can arise due to measurement errors in two ways: first, those with multiplied mass values relative to seed area can arise when seed area is measured for an individual seed and then the robot arm picks up 2 or more seeds and puts them into the balance. A second source of such outliers is when the areas of two or more touching seeds are measured, followed by only a single seed being picked up for measurement of mass. To remove such outliers we identified all points that for a given accession were in the top 1^st^ percentile (top 1%) for one measure and not in 10^th^ percentile (top 10%) for the other measure (**Supplementary figure 2**). After removal of these outliers there were 8207 seeds remaining (2360 for flow cytometry, and 5847 for growth experiment) for which we had reliable seed mass or seed area measurements. Of those, for 6787 (66.70% of initially processed seeds) we had seed area values (on average 67.20 seeds per plate, SD=9.63, *n_plates_* =101), and for 8110 (79.70%) we had seed mass values (on average 76.51 seeds per plate, SD=10.00, *n_plates_* =106). For 6714 seeds (65.98% of 10176 initially processed seeds) both area and mass were available.

Apart from seed mass and projected area we also reconstructed volume from a series of 36 two-dimensional projections per seed, for 7 of the 31 accessions (one 96-well plate each). For these 7 plates the correlation of reconstructed volume and projected area was weaker than that between projected area and seed mass (correlation coefficients r=0.53 and r=0.78 respectively). This is in part because many volume reconstructions failed (volume values close to zero, **Supplementary figure 3**). After identifying a cut-off value for failed measurements as a local minimum of density function (**Supplementary figure 3**) and removing samples for which volume reconstructions failed, the correlation coefficient between seed area and volume was r=0.63. Because of the high failure rate and low correlation with seed area, we did not reconstruct volumes for the remaining 24 accessions and did not use these volume data in further analyses.

### 2.4 Flow cytometric seed screen

FCSS measurements were performed following a protocol from Mau et al. (2021) using a CytoFLEX flow cytometer with plate loader (BeckmanCoulter, Indianapolis, IN, USA) equipped with a 488 nm (blue) laser. The positions of embryo and endosperm peaks were measured using CytExpert Software v2.3 (BeckmanCoulter) following a protocol from Mau et al. (2021). As a control for diploid peak position we used up to two seeds per plate of an obligate sexual *B. stricta*, accession ES854. We screened 2380 seeds (31 accessions, on average *n*=76.67, SD=11.47 per accession) using flow cytometry. We excluded cases for which we could not assign either embryo or endosperm ploidy (179 cases, on average *n*=5.77 per accession, SD=11.34). These include some cases where two peaks were detected in the FCCS, but for which the pattern could not be explained by what is known about gamete and endosperm formation in *Boechera*; i.e. peaks which most likely represented an endoreduplication of the endosperm peak (3x:6x, 5x:10x, 6x:12x, and 9x:18x, where x is the number of chromosome sets). One seed scored as 4x:13x was included in the 4x:12x group, because it most likely represents a measurement error which is increased for peaks located further away from the diploid reference.

For each accession we calculated the proportion of seeds with a unique embryo and endosperm ploidy combination. Similarly to previous studies, we characterized reproductive mode based on the most prevalent (dominant) seed type: in sexual *Boechera*, seeds with a diploid (2x) embryo and triploid (3x) endosperm are most common (>95%), while in diploid apomictic accessions that produce unreduced (2x) and reduced (1x) pollen the most common seeds (>90%) have diploid embryos and respectively hexaploid (6x) or pentaploid (5x) endosperm (Aliyu et al., 2010).

For two accessions (151 seeds) the assignment of reproductive mode based on this experiment differed from previous assignment (Aliyu et al., 2010): TS78 is apomictic – here it had 93.67% sexual seeds; ES744 is sexual – here it had 80% apomictic seeds (2:5 embryo:endosperm ratio). As we are unsure how this mistake originated (as an error in labelling, or using a non-dominant seed for seed increase) we excluded these two accessions from further analyses.

First, we tested whether the proportions of non-dominant seed types differed between sexual and apomictic plants using a chi-squared test. We then tested whether non-dominant seed types were associated with seed size (seed mass and seed projected area, both log-transformed) using linear mixed effect models using seed type (dominant or non-dominant seed, for each accession) as response variable. In these models we included accession as random factor. We ran separate models including all accessions, and for sexual, apomictic 2x:6x, and apomictic 2x:5x groups separately. In each case we used likelihood ratio tests to compare the fit of the full model to alternative reduced models (here: intercept-only models) in order to find a model which best fits the data.

Next, we tested whether specific embryo and endosperm ploidy combinations (ploidy classes) among the non-dominant seeds in of the three groups (apomictic 2x:6x, apomictic 2x:5x, and sexual) were associated with a change in seed mass relative to the dominant seed type. We excluded from this analysis the seed classes for which there were less than 3 seeds per class (7 seeds of 5 classes from apomictic 2x:6x group, 5 seeds of 3 classes from apomictic 2x:5x group, and 3 seeds of 2 classes from sexual group). We fit linear mixed effect models for each group separately, testing whether ploidy class was associated with seed mass (log transformed); accession was included as random factor. Similarly as for other models we used likelihood ratio tests to compare the fit of the full model to alternative reduced models (here: intercept-only models) to ensure best fit to the data.

### 2.5 Growth experiment

We used 12 apomictic and 13 sexual accessions in a controlled growth experiment. For each accession we used 3 individuals for which a 96-well plate of seeds had been measured using the *phenoSeeder*, total *n*=5923 seeds (See: *Individual seed phenotyping with phenoSeeder* for details). Seeds were sterilized using 70% ethanol and 10% bleach, and plated onto sterile square petri plates 12×12 cm (9.19 seeds per plate, SD=1.84). We used half-strength Murashige and Skoog (MS) growth medium, supplemented with 0.1% sucrose and 1% agar, pH adjusted to 5.8. After losses during sterilization, rinsing and handling, 5750 seeds were planted, on average 76.67 seeds per individual (SD=10.09). We excluded from further analyses the two accessions for which the assignment of reproductive mode based on flow cytometry in this experiment differed from previous assignment in our collection (Aliyu et al., 2010): TS78 (216 seeds), and ES744 (237 seeds) (see *Flow cytometric seed screen* for details).

Of the 5297 remaining seeds in the growth experiment (11 apomictic and 12 sexual accessions) reliable seed area or mass values were collected for 5233 (98.9%) (after excluding failed and erroneous measurements, see *Individual seed phenotyping* for details), and for 4184 seeds (79.0%) both seed area and mass values had been measured. The experiment was performed in two blocks, started in March 2018 and in October 2018 respectively, with each block further divided into 2 sub-blocks, where the second sub-block was planted one day later than the first (**Table 1**).

**Table 1:** Planting dates and numbers of seeds used in the growth experiment.

Block	Planted date	Sub-block	Number of plates	Number of seeds
1	22 Mar 2018	1	142	1305
1	23 Mar 2018	2	146	1323
2	4 Oct 2018	3	129	1203
2	5 Oct 2018	4	158	1466

After 7 days of stratification at 4°C in the dark, the seeds were transferred to a growth chamber with 16/8 hours light/dark cycle and 22°C constant temperature. After transferring to growth chamber, germination was checked and recorded daily for 18 days, and plates from each sub-block were scanned after 2, 4, 7, 10, 13, and 16 days for subsequent root length measurements. We used an Epson expression 11000XL scanner with 400 dpi resolution. At the end of the plate phase of the growth experiment (after 21 days for block 1 and 20 days for block 2 in the growth chamber) all plates were photographed using a digital camera Nikon D7200, 50mm focal length. Images were saved as uncompressed files 4000×6000 pixels in size, resolution 96 dpi (block 1), or 4016×6016 pixels, 300 dpi (block 2). For seedlings whose roots had grown longer than the plate, the roots were arranged so that the whole root was visible for photographing. A subset of seedlings from sub-blocks 1 and 2 was transferred to soil (of 11 out of 12 accessions, 371 total seedlings, on average 33.73 per accession, SD=9.02) on 19 and 20 April respectively. We selected seedlings representing all seed sizes which had a root at least ~2 cm long, because smaller seedlings typically fail to establish in soil. The plants were grown in common garden conditions, for the first 5 weeks in a climate chamber and then transferred to a greenhouse facility at the University of Saskatchewan (18°C, 16 h light/14°C, 8 h dark). We recorded new flowering (date of emergence of a first flower) at least once a week until the second week of April 2019. Flowering time was calculated as the number of days since germination for each plant. From 216 of these plants we collected mature seeds to be used in future studies. We used coefficient of variation (CV) as the measure of variation in flowering time per accession.

We measured roots from scanned images and from photographs using WinRHIZO ver. Reg 2013e (Regent Instruments Canada Inc.). Initial root growth rate (RGR-I) was calculated as the slope of linear regression of log-transformed root lengths for all seedlings for which we had at least 3 length measurements measured during the plate phase of growth experiment. Due to time constraints at the end of the plate phase we were not able to photograph individual seedlings with all roots spread-out. We therefore used projected root area as a measure of total root size, as root length measurements would be inaccurate in cases where adjacent separate roots were measured as one (**Supplementary figure 4**). We calculated total root growth rate (RGR-T) as root projected area divided by the number of days the seedling had been growing (from germination day to the end of the plate phase of the growth experiment).

Based on the photos taken at the end of the plate phase we scored developmental abnormalities among all seedlings. We assigned seedling as “normal” when a single seedling with two green cotyledons (and leaves at later developmental stages), hypocotyl, and a root developed from an individual seed. Other seedlings were classified as “abnormal” (**Supplementary figure 6**).

#### 2.5.1 Growth experiment statistical analyses

As a model selection procedure for all models described below, we used likelihood ratio tests, comparing the fit of the full model to alternative reduced models in order to find the model that best described the data. We used generalized linear mixed-effect models (GLMMs) to investigate whether average seed size (projected area and seed mass measured with *phenoSeeder*) differed between the sexual and apomictic accessions. In these GLMMs we used individual nested under accession as random factors.

We then used GLMMs to analyze the effects of seed size (seed mass and seed area) and reproductive mode (sexual, apomictic) on germination success. In further analyses we considered only seed mass, which turned out to be a better predictor of seed traits (see Results). We then used GLMMs to analyze the effects of seed mass and the reproductive mode (sexual, apomictic) on germination timing and seedling development (normal vs. abnormal development). Seed mass and seed area (where included) were scaled by their standard deviations of the mean. In these models we used accession and plate nested under sub-block as random factors.

We used GLMMs to analyze the effects of seed mass and the reproductive mode (sexual, apomictic) on seedling root growth using log-scaled root growth rate as response variable. In these models we used accession as random factor; we excluded individual, sub-block and plate to achieve model convergence. We also analyzed the effect of reproductive mode on individual variation in seedling root growth. We calculated coefficients of variation (CV) per individual and used GLMMs with log-transformed CV values as a response variable and accession as random factor.

We used GLMMs to analyze the effects of seed mass and reproductive mode (sexual, apomictic) on flowering time. In order to increase the normality of the flowering time data distribution we used Tukey’s ladder of powers method as implemented in the R package *rcompanion* (Mangiafico, 2019) to find the best lambda parameter for the power transformation, and used transformed values as a response variable. We used accession as random factor in this model.

We used a Wilcoxon rank sum test to compare the average variation in flowering time (CV values per accession).

#### 2.5.2 Analyses of selection

To estimate the type and strength of selection on seed mass in sexual and apomictic accessions we used regression-based selection gradient analysis (Lande and Arnold, 1983). We used two fitness measures: germination efficiency, and a composite measure of germination efficiency and normal seedling development. In this analysis we included twins (i.e. polyembryony) as normally developed, because in all twin cases at least one of the pair developed normally. Other than twins, abnormal seedlings were unlikely to develop until adulthood (See Results and **Supplementary figure 6**). We estimated linear and quadratic univariate selection gradients, doubling the coefficients and standard errors for the second-order terms from the regression analyses, including both linear and quadratic terms (see Stinchcombe et al., 2008). We used GLMMs with the respective fitness measures as response variable and accession as random factor. Seed mass was scaled by the respective standard deviation of the mean in sexual and apomictic groups. We plotted the estimated fitness functions to provide graphical representations and to allow for the identification of possible intermediate fitness extremes in the data.

We used R for data manipulation and statistical analyses (R Core Team, 2019); R packages *lme4* (Bates et al., 2015) for fitting GLMMs, *effects* (Fox, 2003; Fox and Weisberg, 2019) and *emmeans* (Lenth, 2019) for predictions of GLMMs and pairwise contrasts.

## 3 Results

### 3.1 Mean seed size explained by ploidy level, species identity and chloroplast lineage

**Figure 2:**
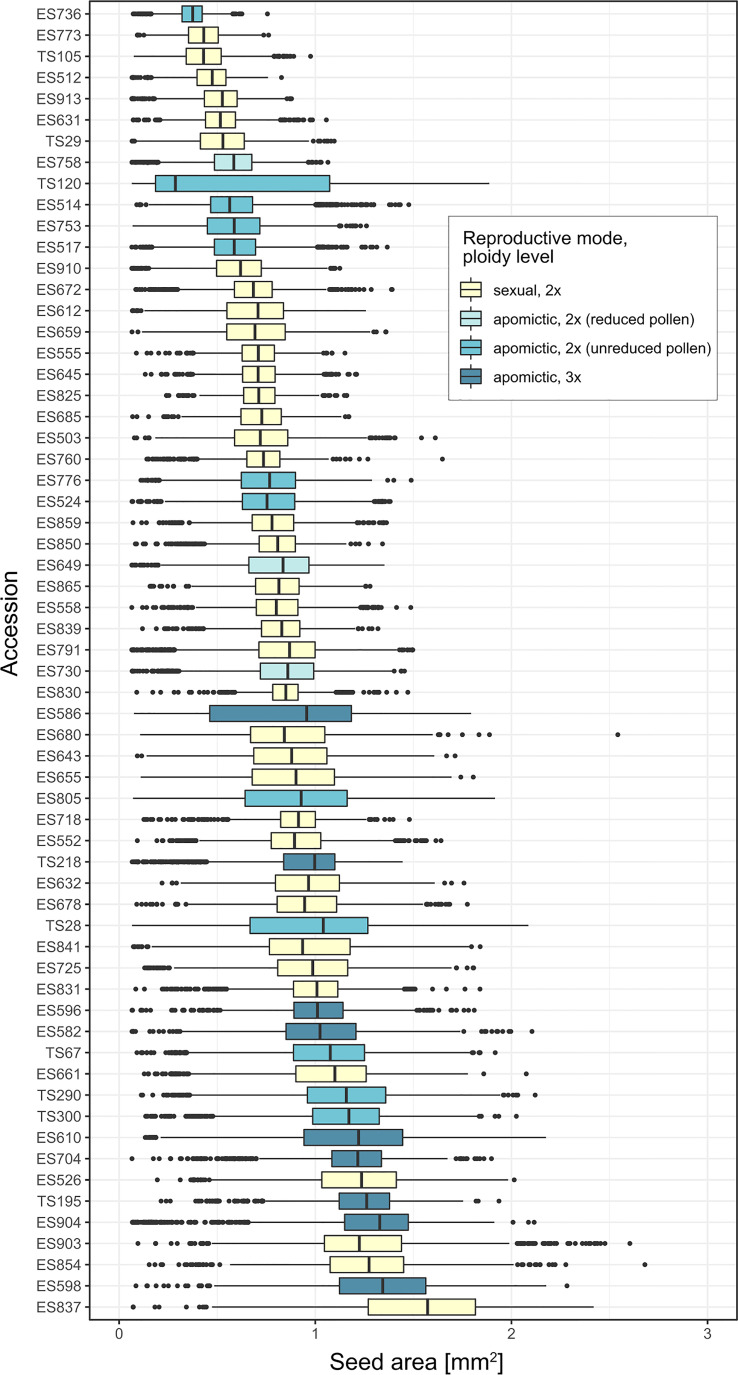
Boxplots of projected seed area [mm^2^] per accession, ordered by mean seed area.

There was large variation in average seed size among the 62 *Boechera* accessions (median range: 0.29 – 1.57 mm^2^), as well as large variation within accessions (**Figure 2**).

A comparison of a model including ploidy level, species identity, and chloroplast lineage with the full model including these factors and the reproductive mode demonstrated that the reduced model did not significantly decrease the model fit (χ^2 =^ 0.46, DF=1, *P*=0.50). Further reducing the model resulted in significantly worse fits (**Supplementary table 3**), which suggested that three factors: ploidy level, species identity and chloroplast lineage contributed to explaining diversity in mean seed area in this sample.

**Figure 3:**
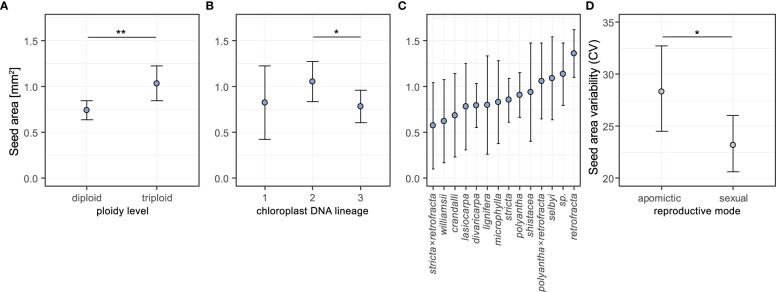
Effects of ploidy level **(A)**, chloroplast DNA lineage **(B)** and species **(C)** on seed area, and effects of reproductive mode on seed area coefficient of variation **(D)** estimated from general linear mixed-effect models (see text for details of GLMMs). In the analysis of seed area for each of the three factors the estimated means were averaged over the levels of the other two factors using weights proportional to their frequencies in the data set. Error bars represent 95% confidence intervals. Significance levels: ∗ < 0.05, ∗∗ < 0.01, ∗∗∗ < 0.001.

Seeds of triploid accessions were 33.3% larger than those of diploids (**Figure 3A**, pairwise contrast diploid-triploid: Z=-3.105, *P*=0.002; pairwise contrasts results are averaged over the levels of the other two factors). There was a difference in mean seed area between chloroplast lineage 2 and 3 – the former had on average 29.6% larger seeds (Z= 2.56, *P*= 0.028; **Figure 3B**). There were no differences found in the other two pairwise contrasts (lineage 1 vs 2: Z= -0.833, *P*=0.683; 1 vs 3: Z= 0.168, *P*=0.985; **Figure 3B**). Full model results are in **Supplementary table 4**. The estimated means for individual species varied between 0.57 and 1.36 mm^2^ (**Figure 3C**). Estimates of random effects demonstrated that between-accession variation was four times larger than the variation between individuals within accessions (**Supplementary table 4**).

### 3.2 Seed size variability: reproductive mode, but not ploidy level had an effect on the coefficient of variation in seed size

We used coefficient of variation (CV) as a measure of seed size variability, and investigated the effects of reproductive mode and ploidy level on this trait using generalized linear mixed-effects models. The likelihood ratio tests of alternative GLMMs demonstrated that reproductive mode, but not ploidy level, had an effect on the seed size variability (**Supplementary table 5**). Apomictic individuals had on average 19.8% higher coefficients of variation in seed size than sexual individuals (Z= 2.148, *P*= 0.032; **Figure 3D**, see also **Supplementary table 6**).

Next, we investigated whether larger seed size variation in apomictic individuals was driven primarily by small seeds, large seeds, or both. In order to do so, we split the full dataset based on median seed size value for each individual into two datasets: above- and below-median. We then calculated individual CVs and found best-fitting models using likelihood ratio tests.

For the above-median dataset the model including reproductive mode did not have a significantly better fit than an alternative model including only intercept (χ^2 =^ 0.970, DF=1, *P*=0.325), demonstrating that reproductive mode does not explain the variation in seed size in this portion of the data. In contrast, for the below-median dataset the model including reproductive mode had a significantly better fit than intercept-only model (χ^2 =^ 6.9833, DF=1, *P*=0.008). The coefficient of variation for apomictic individuals was on average 27.3% larger than that of sexual individuals in this dataset. These results indicate that the increased variation in seed size among apomictic individuals is driven primarily by small seeds (and at least some of these small seeds are viable, see *Seed size was positively associated with the probability of germination*).

### 3.3 In both apomictic and sexual accessions non-dominant seed types were on average smaller than the dominant seed types

**Figure 4:**
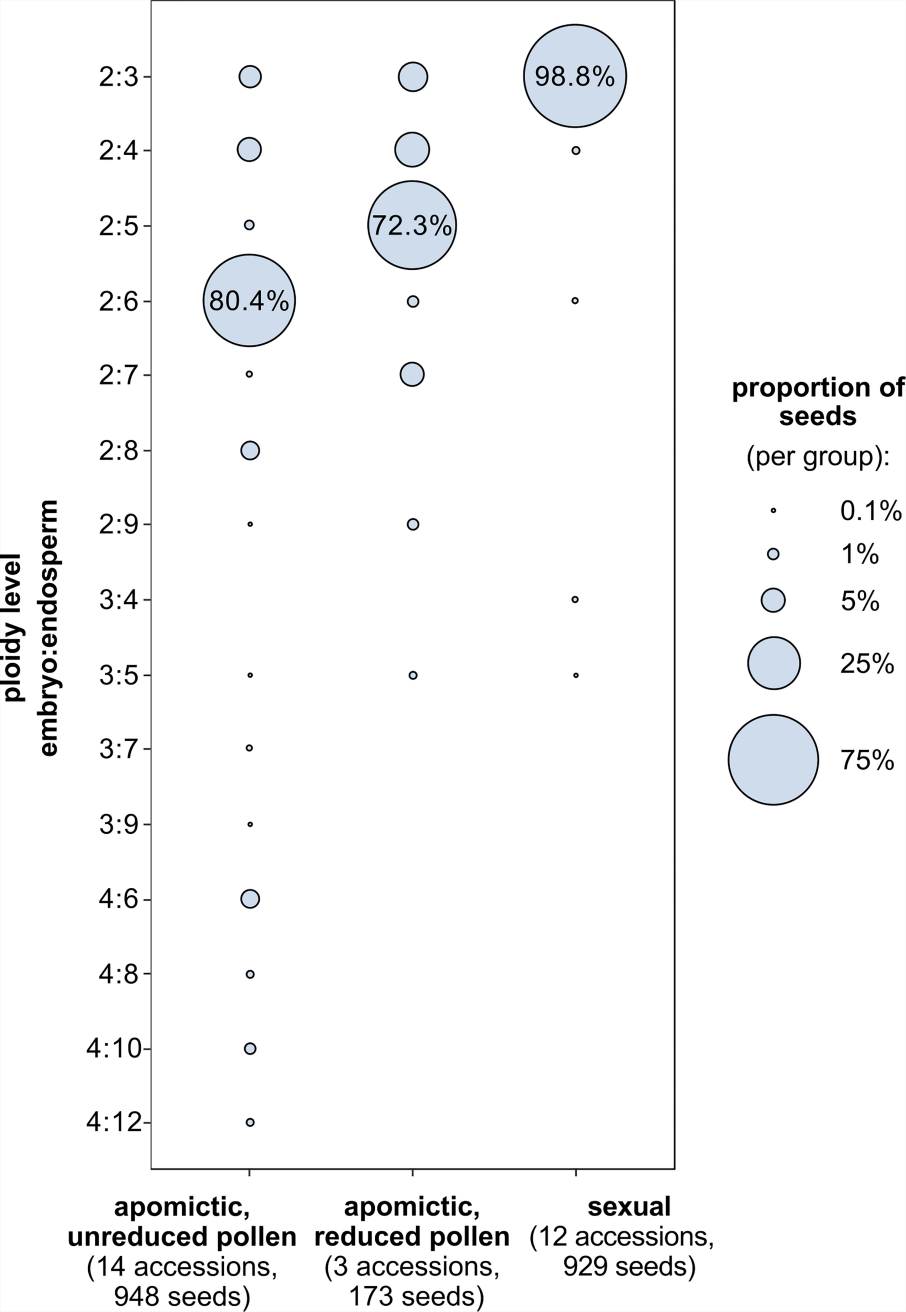
Proportions of seed types characterized by embryo and endosperm ploidy combinations (y-axis) among sexual and diploid apomictic *Boechera*. Ploidy level denotes the number of chromosome sets (x) in the embryo and endosperm. Among apomictic accessions there are two groups: those that produce unreduced (2x) and reduced (1x) pollen (“balanced” and “unbalanced” *sensu* Mau et al., 2021), which produce seeds with predominantly hexaploid (6x) and pentaploid (5x) endosperm respectively.

For 29 accessions analysed using FCSS we confirmed previously assigned reproductive mode (Aliyu et al., 2010; Mau et al., 2015, **Supplementary table 1**): 12 sexual accessions (96.6% to 100% of seeds with 2x:3x embryo:endosperm ploidy), 14 apomictic accessions with predominantly hexaploid endosperm (50.0% to 97.3% seeds with 2x:6x embryo:endosperm ploidy) and 3 apomictic accessions with predominantly pentaploid endosperm (58.5% to 85.9% seeds with 2x:5x embryo:endosperm ploidy). Apart from the most common seed type, most accessions (6 of the sexuals and all the apomicts) produced other (non-dominant) types of seeds, characterized by different ploidy of endosperm and/or embryo (**Figure 4**). Apomicts had a larger proportion of non-dominant seed types than sexuals (χ^2 =^ 185.3, DF=1, *P*<0.001).

**Figure 5:**
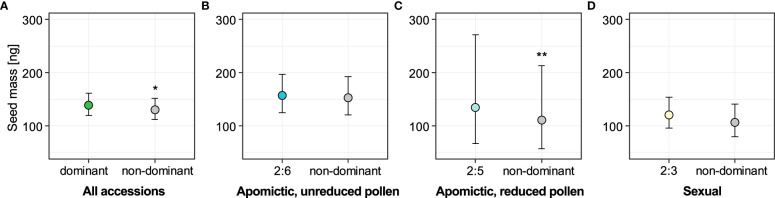
Effect of seed type (dominant vs. non-dominant) on seed mass for all accessions **(A)** and for the three reproductive mode and ploidy groups **(B-D)**. Sexual accessions produce predominantly seeds with 2x:3x embryo:endosperm ploidy; apomictic accessions with unreduced and reduced pollen produce predominantly 2x:6x and 2x:5x seeds, respectively. Non-dominant seeds are all other seed classes with known embryo and endosperm ploidy in each group. Error bars represent 95% confidence intervals. Significance levels: ∗ < 0.05, ∗∗ < 0.01, ∗∗∗ < 0.001.

Next, we tested whether the non-dominant seeds were associated with seed size variation. Out of 2050 seeds for which we had ploidy information, we also had seed size measurements obtained with *phenoSeeder*: mass for 2010 and projected area for 1746 seeds. We used a linear mixed-effects model to test whether seed mass was affected by seed type, the latter considered as a binary fixed factor (dominant and non-dominant seed types were coded as 0 and 1 respectively). Model including “seed type” factor fit the data significantly better than the alternative reduced model including intercept only (χ^2 =^ 6.43, DF=1, *P*=0.011). Seeds from the non-dominant ploidy group were on average 6.69% lighter than dominant seeds in an analysis including all 29 accessions (**Figure 5A**, see also **Supplementary table 7**). We then separated the dataset into groups defined by reproductive mode and dominant endosperm ploidy, and again tested the association of seed mass with seed type (dominant *vs.* non-dominant seeds). For all three of the groups (sexual, apomictic 2x:6x and apomictic 2x:5x) the non-dominant seeds were lighter than the dominant seeds (**Figures 5B-D**, see also **Supplementary table 7**), but we found strong support for this effect only for apomictic accessions with dominant 2x:5x seed type (χ^2 =^ 10.64, DF=1, *P*=0.001). Non-dominant seeds deviating from a 2x:5x embryo:endosperm ploidy ratio had on average 18.8% lower mass (**Figure 5C**). For sexual accessions and apomictic accessions with dominant 2x:6x seeds, the non-dominant seeds were on average 13.2% and 2.6% lighter, respectively (**Figures 5B, D**), but the models including seed type did not fit the data better than reduced intercept-only models (sexual: χ^2 =^ 2.45, DF=1, *P*=0.12; apomictic 2x:6x: χ^2 =^ 0.65, DF=1, *P*=0.42).

There was no association between seed type (dominant vs. non-dominant seeds) and projected seed area (likelihood ratio test between a model with “seed type” binary factor and an alternative reduced model including intercept only: χ^2 =^ 0.242, DF=1, *P*=0.623). This suggests that seed mass is a more informative measure of seed size than projected seed area.

### 3.4 None of the non-dominant seed ploidy classes had on average heavier seeds than the dominant seeds in both apomictic and sexual groups

**Figure 6:**
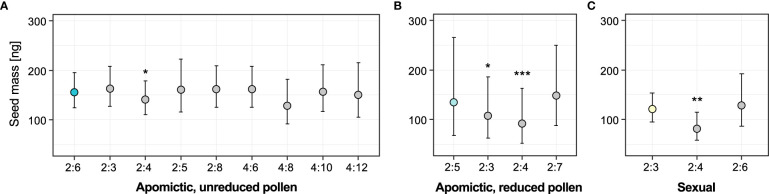
Effects of ploidy classes (specific seed embryo:endosperm ploidy combinations) on seed mass. **(A)** Apomictic accessions with unreduced pollen (with predominantly 2x:6x seeds), **(B)** Apomictic accessions with reduced pollen (with predominantly 2x:5x seeds), **(C)** Sexual accessions (with predominantly 2x:3x seeds). Coloured circles represent the reference (dominant) seed class for each group, and grey circles are the non-dominant seed classes in each group. Error bars represent 95% confidence intervals. Significance levels of the effect of individual ploidy classes: ∗ < 0.05, ∗∗ < 0.01, ∗∗∗ < 0.001.

We used linear mixed-effects models to test whether seed mass was associated with specific embryo:endosperm combinations (ploidy classes) for the three groups of accessions: “balanced” apomictic that produce unreduced pollen (seeds with predominantly 2x:6x embryo:endosperm ploidy ratio), “unbalanced” apomictic that produce reduced pollen (predominantly 2x:5x seeds), and sexual (predominantly 2x:3x seeds). Models including “ploidy class” factor fit the data significantly better than the alternative reduced models including intercept for two of the groups: apomictic, reduced pollen (χ^2 =^ 25.01, DF=3, *P*<0.001) and sexual (χ^2 =^ 9.15, DF=2, *P*=0.010). For the apomictic group with unreduced pollen the full model did not fit the data better than intercept-only model (χ^2 =^ 8.16, DF=8, *P*=0.418). The results of full models are in **Supplementary table 8**. In all three groups the ploidy classes of non-dominant seeds were either smaller or not different from the dominant seed type (**Figure 6**). In all groups, the apomictic seeds with tetraploid (autonomous) endosperm were significantly lighter than the dominant class (on average 10.1% lighter than 2x:6x seeds, 37.4% lighter than 2x:5x seeds, and 38.9% lighter than 2x:3x seeds). In the apomictic group that produced reduced pollen, non-dominant sexual seeds (2x:3x embryo:endosperm ratio) were 22.24% lighter than the dominant 2x:5x seeds (**Figure 6B**). In contrast, sexual seeds produced by apomictic 2x:6x accessions were not significantly different from the dominant seed class (**Figure 6A**). Similarly, 2x:6x seeds produced by the sexual accessions were not significantly different from sexual seeds (**Figure 6C**).

### 3.5 Sexual and apomictic accessions used in growth experiment did not differ in average seed size or seed size variability

We chose a subset of accessions for the growth experiment which were representative of the larger sample (62 accessions) used in this study in terms of average seed size (see section: “*Mean seed size explained by ploidy level, species identity and chloroplast lineage*”). To confirm this, we tested whether the accessions used in the growth experiment (12 diploid sexual and 11 diploid apomictic accessions, **Supplementary table 1**) differed in average seed size (projected area and seed mass measured with *phenoSeeder*). The effect of reproductive mode doesn’t significantly explain seed mass or projected seed area: models including reproductive mode as fixed factor were not significantly different from alternative models containing only intercept (seed mass: χ^2 =^ 0.063, DF= 1, *P*= 0.802; seed area: χ^2 =^ 0.018, DF=1, *P*=0.894). Thus, there was no difference in average seed size between sexual and apomictic accessions, which confirmed our choice of representative samples in the growth experiment. We also measured seed size variability: among all 62 accessions we found that apomictic individuals had higher coefficients of variation (CV) than sexual individuals (**Figure 3D**), whereas in the subset chosen for growth experiment the individual CVs of seed mass did not differ between apomictic and sexual groups (comparison between GLMM including reproductive mode and intercept-only model χ^2 =^ 0.835, DF= 1, *P*= 0.361). Failing to detect a difference in seed size variability in this subset is likely due to much lower statistical power: here the individual CVs were calculated based on average on 74.9 seeds per individual, 8.7 times fever seeds than in the analysis based on bulk seed scans (on average 652 seeds per individual).

### 3.6 Seed mass is under balancing selection in *Boechera*, and selection against small seeds is stronger in apomictic than in sexual accessions

Overall 73.5% of all seeds (N=5297) germinated within the duration of the experiment, 74.2% of sexual seeds (N=2706), and 72.9% of apomictic seeds (N=2591). We first tested whether germination efficiency was associated with reproductive mode and seed size (seed area and mass) using a GLMM. The model including main effects of seed mass, seed area, reproductive mode and the interaction between reproductive mode and seed mass best fit the data (**Supplementary table 9**). Larger seeds had higher probability of germination, but the slope of this relationship differed between sexual and apomictic accessions (**Supplementary figure 5**, **Supplementary table 10**). The effects of seed mass were larger than that of seed area, which again suggests that seed mass is a more informative measure of seed size than projected seed area (also see “*In both apomictic and sexual accessions non-dominant seed types were on average smaller than the dominant seed types*”).

We scored seedling development among 3827 seedlings (on average 57.98 per individual, SD=23.53) as normal or abnormal, and categorised the developmental abnormalities into several distinct categories (**Supplementary figure 6**, **Supplementary figure 7**). Among apomictic accessions 96.8% of seedlings were classified as normal, compared to 92.6% among sexuals, and there was a strong effect of reproductive mode on the difference in proportions of normal *vs*. abnormal seedlings (χ^2 =^ 32.41, DF=1, *P*<0.001). Most categories of developmental abnormalities were found among both sexual and apomictic accessions (**Supplementary figure 7**). We tested whether seed mass and reproductive mode affected seedling development (normal vs. abnormal development) and found a significant effect of reproductive mode, seed mass and the interaction of seed mass and reproductive mode on seedling development (**Supplementary table 13**). There were more abnormally-developed seedlings among sexual seeds. Within apomictic accessions, there were more developmentally-abnormal seedlings among lighter seeds. There was no relationship among seed mass and seedling developmental abnormalities among sexual accessions. (**Supplementary Figure 8**, **Supplementary table 14**)

**Figure 7:**
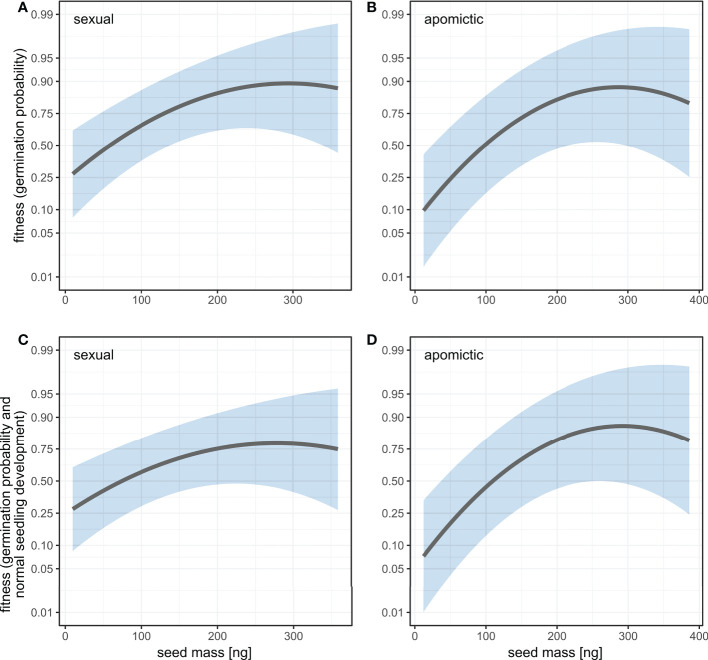
Fitness as a function of seed mass estimated using univariate linear and quadratic selection gradients for two fitness measures: germination probability **(A, B)** and a composite measure of germination probability and normal seedling development **(C, D)** in sexual **(A, C)** and apomictic **(B, D)** accessions. Fitness is plotted on logit scale. Shaded areas represent 95% confidence intervals.

We estimated linear and quadratic selection coefficients for seed mass using two fitness measures: germination probability and a composite measure of germination probability and normal seedling development. For both measures the linear selection coefficients are positive and have higher values for apomictic accessions (**Table 2**), which means selection against small seeds is stronger in apomicts than in sexuals (**Figure 7**). The difference between sexual and apomictic accessions is more pronounced for the composite fitness measure, which signifies size-dependence of developmental abnormalities among apomicts. The quadratic selection gradients are negative, which together with the shape of the fitness function demonstrates balancing selection on seed mass (**Figure 7**, **Table 2**). The estimated maxima of the fitness function for the two fitness measures, germination probability and the composite measure, are at 292.0 ng and 277.9 ng respectively in sexual accessions, and at 287.8 ng and 291.7 ng in apomictic accessions.

**Table 2:** Standardized univariate linear (β) and quadratic (γ) selection gradients (± SE) for seed mass in sexual and apomictic diploid *Boechera*.

fitness measure	reproductive mode	β	γ
germination probability	sexual	0.862^***^ (± 0.109)	-0.406^***^ ( ± 0.107)
	apomictic	1.081^***^ (± 0.092)	-0.673^***^ (± 0.095)
germination probability and normal seedling development	sexual	0.602^***^ (± 0.098)	-0.330^***^ (± 0.099)
	apomictic	1.164^***^ (± 0.094)	-0.686^***^ (± 0.096)

Bold values indicate statistically significant selection gradients, significance levels: * < 0.05, ** < 0.01, *** < 0.001.

### 3.7 Heavier seeds germinated earlier, and sexual seeds tended to germinate later than apomictic seeds

**Figure 8:**
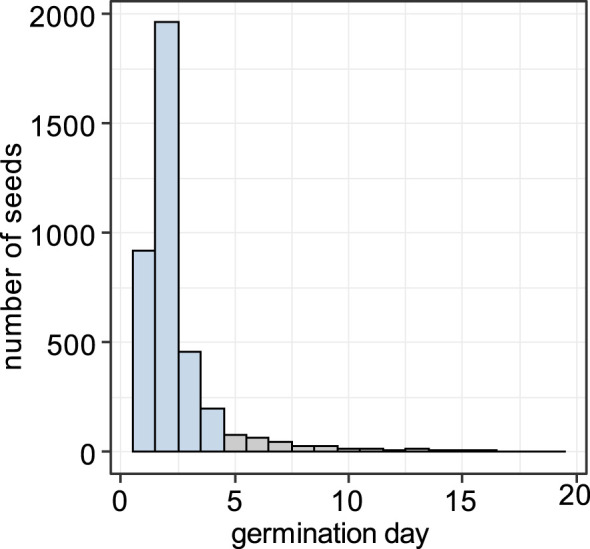
Histogram of germination timing. Seeds that germinated within the first 4 days (blue) were treated as “early” and those that germinated later than 4 days (gray) as “late”.

Germination in the growth experiment began 1 day after transferring seeds into the growth chamber, and 91.0% of the seeds that eventually germinated did so within the first 4 days (**Figure 8**). We defined the seeds that germinated within first 4 days as “early”, and the ones that germinated later than 4 days as “late”.

We then tested whether seed mass was associated with early or late germination. We did not consider seed area in further analyses because seed mass was a better predictor of seed traits (see previous results, **Supplementary figure 5**), and we had seed mass values for more seeds than both area and mass values. We selected best-fitting model based on likelihood-ratio tests (**Supplementary table 11**).

**Figure 9:**
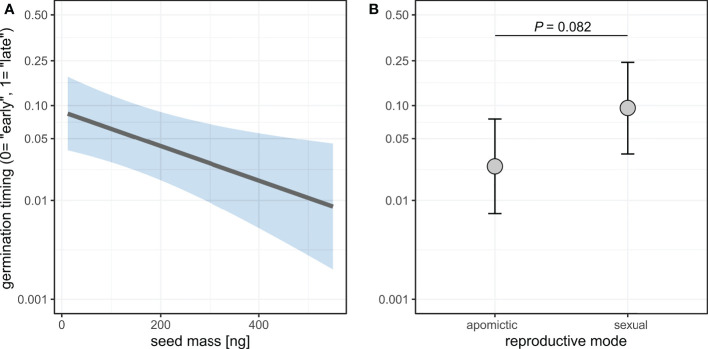
Effects of seed mass **(A)** and reproductive mode **(B)** on germination timing (“early” *vs*. “late” germination). Germination probability is plotted on a logit scale. Shaded area **(A)** and error bars **(B)** represent 95% confidence intervals.

Seed mass was significantly associated with the timing of germination, with heavier seeds germinating earlier (**Figure 9**, **Supplementary table 12**). The model also demonstrated a weak effect of reproductive mode whereby sexual seeds germinated on average later than apomictic seeds (Odds ratio=4.13, *P*=0.082). The chi squared test for equality of proportions is consistent with this result, as there was a significant difference in the proportions of “early” and “late” germinating seeds between sexual and apomictic groups (χ^2 =^ 31.615, DF=1, *P*<0.001).

### 3.8 Seedling root growth was higher for heavier seeds, and apomictic seedlings had on average higher root growth than sexual seedlings

Total root growth rate (RGR-T) is the root projected area measured at the end of the plate phase of the growth experiment divided by the number of days since germination. We found a significant effect of reproductive mode, seed mass, seedling development, and the interaction of seedling development and reproductive mode on total root growth (**Supplementary table 15**).

**Figure 10:**
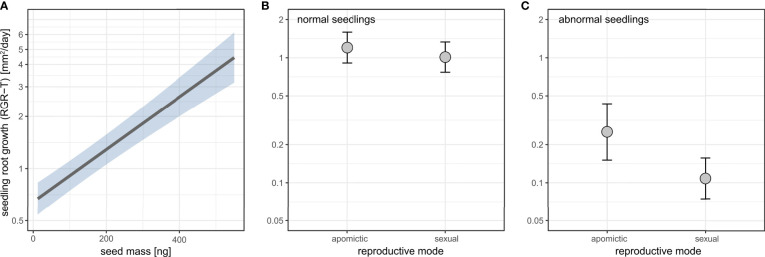
The effects of seed mass **(A)** and reproductive mode **(B, C)** on seedling root growth rate (RGR-T) in normal **(B)** and abnormal **(C)** seedlings. Seedling root growth rate (RGR-T) is plotted on log scale. Shaded area **(A)** and error bars **(B, C)** represent 95% confidence intervals.

Seed mass positively affected seedling root growth (**Figure 10A**, **Supplementary table 16**). Apomictic seedlings had on average higher root growth, and this effect was stronger for abnormal seedlings (**Figure 10B, C**, **Supplementary table 16**).

A large amount of variability in root growth rate was found both among sexual and apomictic accessions (**Supplementary figure 9**). There was no effect of reproductive mode on individual variability (measured as coefficient of variation, CV) in total root growth (RGR-T): the model including reproductive mode did not significantly better fit the data than an alternative model containing intercept only (**Supplementary table 17**).

Initial root growth rate (RGR-I) was calculated as the slope of linear regression of log-transformed root lengths for all seedlings for which we had at least 3 root length measurements (*n*=3839 seedlings). Of those, we had reliable seed mass data for 3441 seeds. We found no effect of reproductive mode on initial root growth rate (**Supplementary table 18**), but similarly to the total root growth rate (RGR-T), there was a positive relationship between seed mass and RGR-I (**Supplementary figure 10**, **Supplementary table 19**).

### 3.9 Flowering time and its variability does not depend on reproductive mode or seed size

Of the 485 plants transferred to the greenhouse 14 (2.9%) died, and 27 (5.6%) did not flower during the course of the experiment. We excluded 22 plants (4.5%) where flowering date was missed (i.e. when the first flowers noticed were clearly older than a week). We excluded accession TS78 for which the assignment of reproductive mode based on flow cytometry in this experiment differed from previous assignment in our collection (51 plants). Of 371 remaining plants, we had reliable seed mass values for 362. Neither seed mass nor reproductive mode had an effect on the flowering time (**Supplementary tables 20, 21**; **Supplementary figure S11**). As a measure of variability in flowering time we calculated coefficient of variation (CV) or each of the 6 sexual and 5 apomictic accessions (371 plants). Sexual accessions had on average higher coefficient of variation (mean CV_sex_= 9.42, SD= 4.57) than apomictic accessions (mean CV_apo_= 5.77, SD= 1.38), but the effect of reproductive mode was weak (Wilcoxon rank sum exact test: W = 6, *P* = 0.126).

## 4 Discussion

Despite many disadvantages associated with asexual reproduction (see Lovell et al., 2017), naturally occurring apomictic plants are highly successful from an evolutionary perspective (Hojsgaard & Hörandl, 2019). On a large geographic (metapopulation) scale, apomictic plants can encompass as much, if not more, genetic and phenotypic variability compared to their sexual relatives (Bierzychudek, 1985; Karbstein et al., 2021) a pattern which is influenced both by historical factors (e.g. origins from sexual relatives) and a suite of characteristics associated with asexual reproduction (see: Hojsgaard and Hörandl, 2019; Karbstein et al., 2021). On smaller scales of both time and geography, apomixis can provide advantages over sexual congeners by nature of clonal reproduction (Wickell et al., 2017). The ability of apomictic plants to produce clonal embryos during seed production is considered a highly desirable and disruptive trait which could significantly improve global agriculture (Conner and Ozias-Akins, 2017; Underwood and Mercier, 2022).

In contrast to pseudogamy in asexual animals, whereby physiological activation of the unfertilized egg cell by sperm is required (Beukeboom and Vrijenhoek, 1998) fertilization of the ovule’s binucleate central cell is required in many apomictic plants for proper endosperm formation (Conner and Ozias-Akins, 2017). Apomictic *Boechera* exhibit high levels of phenotypic variability during seed development (Aliyu et al., 2010) which may have implications for fitness, and we have thus examined several seed traits associated with fitness in a diverse set of sexual and apomictic accessions of *Boechera* under controlled conditions. Importantly, as hybridization underlies multiple origins of the apomixis phenotype in *Boechera* (Kantama et al., 2007; Beck et al., 2012), the broad geographic and phylogenetic (i.e. cpDNA) sampling here encompasses multiple independently-derived asexual lineages from which statistical comparisons between reproductive modes can be made.

### 4.1 History and ploidy have significant influences on seed size in *Boechera*

To begin with, we have shown several factors besides reproductive mode which contribute to explaining diversity in mean seed area, including ploidy level, species identity and chloroplast lineage. For example, seeds with triploid embryos were 33% larger than those with diploid embryos (**Figure 3**), but no effect was found between sex and apomixis. It is unclear whether increased ploidy affected embryo and/or endosperm proliferation, or whether it had an effect on cell (i.e. organ) size in *Boechera.* Our sampling showed that between-accession variation was four times larger than the variation between individuals within accessions, as was found in comparisons of phenotypic traits associated with seed development and apomixis penetrance between both diploid and triploid *Boechera*, which showed that trait variation was mainly genotype-specific rather than being associated with ploidy (Voigt-Zielinski et al., 2012).

Importantly, chloroplast (cp) lineage 2 had 30% larger seeds than cp lineage 3, despite having broad and mostly overlapping geographic ranges (Kiefer et al., 2009). This implies either a maternal or phylogenetic (i.e. history) effect on seed size. Seed size has been shown to be determined by maternal effects in other *Brassica*, for example through upregulation of hormones and developmental genes associated with silique (pod) structure in *B. napus* (Li et al., 2019). In angiosperms, seed size can be influenced by variation on many organisational levels, much of which is associated with maternal effects (Phillips and Evans, 2020). As these differences in size did not translate into identical differences when using seed mass (see below), we suspect that the differences in size measured here are related to the development of seed wings, as has been shown to be a defining characteristic in *Boechera* (Windham et al., 2016). The association of seed size with cp lineage and species (**Figure 3**) supports our choice of a diverse set of accessions from which contrasts between sexual vs apomictic reproductive can be made.

Apomictic seeds are not more uniform in size than sexual seeds, despite genetic uniformity of the maternal gametophyte in the former. In apomicts, variability in seed size is higher (bulk seed scans, **Figure 3**) or equal (in the smaller sample used for growth experiment) to the variability in sexual seeds. The result of higher variability is driven by small seeds, which could be possibly explained by a higher proportion of aborted seeds among apomictic lineages (although not all small seeds are inviable - see below) associated with mutation accumulation (Lovell et al., 2017). Finally, the “costly” hybrid origin of apomictic *Boechera* lineages (Rushworth and Mitchell-Olds, 2021), rather than apomixis *per se*, could also underlie the seed size variability measured here.

#### 4.1.1 Endosperm balance number and seed size

Considering the lack of association between seed type (dominant vs. non-dominant seeds) and projected seed area, we conclude that seed mass is a more informative measure of seed size and associated fitness effects compared to projected seed area. Overall, seeds with other than dominant embryo and endosperm ploidy combinations (**Figure 4**) were lighter than the dominant type (**Figure 5A**). Apomicts had a larger proportion of non-dominant seed types compared to sexuals, and non-dominant seeds were 18.8% lighter than the dominant seeds in unbalanced apomicts (i.e. those producing dominant 2x:5x seed type; **Figure 5C**).

Among specific embryo/endosperm ploidy combinations, seeds with tetraploid (automomous – developing without fertilization of the central cell) endosperm (2x:4x) are on average significantly smaller (**Figure 6**) in all groups, and the proportion of such seeds is highest in apomicts (**Figure 4**). Importantly, the consistently small size of apomictic seeds with autonomous endosperm formation both demonstrate an extreme deviation from the normal sexual EBN (i.e. no paternal alleles), and support EBN theory whereby a relative increase in male:female genome dosage is expected to lead to larger seeds (Haig and Westoby, 1989). In support of this, seeds of the 4:8 ploidy class, while having a weak effect, showed a tendency for smaller size compared to other seed ploidy classes (**Figure 6A**).

Voigt-Zielinski et al. (2012) showed that pseudogamy (i.e. fertilization of the central cell) is likely under selection for proper seed development in apomictic *Boechera*, even though all embryos are genetically identical to the mother plant. Hence, in apomicts the maintenance of endosperm balance number (EBN) in balanced diploids (2x embryo: 6x [4m:2p] endosperm) and triploids (3x embryo: 9x [6m:3p] endosperm) appears to be a requirement for proper endosperm (and seed) formation. Nonetheless, in *Boechera* the widespread occurrence of “unbalanced” diploid apomicts characterized by pentaploid endosperm (2x embryo: 5x [4m:1p] endosperm) demonstrates that strict maintenance of the EBN is not always necessary for proper seed formation (Mau et al., 2021).

Apomixis spread in natural populations is mediated by rare haploid pollen produced by unbalanced apomicts, which occasionally fertilize haploid sexually-derived egg cells to initiate a new apomictic lineage (Mau et al., 2021; Mau et al., 2022). Thus, we hypothesize that the ability of some apomicts to tolerate variation in the EBN exists as a factor among the repertoire of genetic elements underlying apomixis in *Boechera*. This is supported by unbalanced apomicts which produced non-dominant sexual seeds (2x:3x embryo:endosperm ratio) which were 22.24% lighter than their dominant 2x:5x seeds (**Figure 6B**). In contrast, sexual seeds produced by apomictic 2x:6x accessions were not significantly different from the dominant seed class (**Figure 6A**), nor were 2x:6x and 2x:3x seeds produced by the sexual accessions (**Figure 6C**). These data show that the production of 2x:5x dominant seeds in unbalanced apomicts can no longer be balanced by the addition of a male gamete to the central cell, thereby implying the action of a novel genetic factor(s) underlying tolerance to the EBN. In the context of applying apomixis to crop plants, a completely autonomous apomict which produces proper endosperm in the absence of fertilization would be ideal.

Finally, as all seeds in this experiment were derived through selfing, no new alleles had been introduced which could potentially influence seed size. Meiosis has been shown to occur during apomictic pollen development in some lineages of *Boechera* (Böcher, 1951) and hence there is the possibility of recombination leading to allelic variation in pollen within a selfing plant, with subsequent effects on endosperm formation through (e.g.) parent of origin effects.

### 4.2 Fitness effects of seed size variation in sexual and apomictic *Boechera*

The data so far demonstrate that both apomictic and sexual *Boechera* produce variable seed mass and area, and that dominant seeds are typically heavier than non-dominant seeds (**Figure 5A**), which begs the question as to whether larger seeds are selectively favourable with respect to survival of most offspring.

There was a small effect of reproductive mode on germination timing with sexuals tending to germinate later (**Figure 9**). Larger seeds germinated more quickly than small seeds (**Figure 9**), and this is consistent with larger seeds also leading to higher rate of root growth (**Figure 10**). Furthermore, in both sexuals and apomicts, abnormal seedlings with slower root growth were associated with smaller seeds (**Figure 10**). The effect was larger considering abnormal seedling development, which in apomicts more often come from small seeds, whereas in sexuals there is no association between seed size and normal vs. abnormal seedlings. Abnormal apomictic seedlings had higher root growth rates than abnormal sexual seedlings, which may be reflective of their hybrid origin (i.e. heterotic root development) rather than apomixis. The same trend is present for normal seedlings, but the effect is very small.

Heavier seeds mean higher offspring success in *Boechera*. Seed mass was an important predictor of several traits: heavier seeds had higher probability of germination, they germinated earlier, had higher root growth rates, and in apomictic accessions produced higher proportions of normally developed seedlings. Estimations of linear and quadratic selection coefficients for seed mass using germination probability and a composite measure of germination probability and normal seedling development are positive and have higher values for apomictic accessions (**Table 2**), and thus selection against small seeds is stronger in apomicts than in sexuals (**Figure 7**).

However, the selection gradient analysis demonstrated balancing selection, meaning that the largest seeds were at a fitness disadvantage (**Figure 7**, **Table 2**), and that there is an intermediate fitness maximum in seed size. The difference between sexual and apomictic accessions is more pronounced for the composite fitness measure, which signifies size-dependence of developmental abnormalities among apomicts. If one considers variability in pollen and seed formation, in addition to bidirectional gene flow between sexual and apomictic *Boechera* (Aliyu et al., 2010; Mau et al., 2021; Mau et al., 2022), there is the possibility of changes to the EBN during seed formation which favour the male (pollen) genome and larger seed size (*sensu* Haig and Westoby, 1991). Hence, our observation of balancing selection acting on apomictic seeds may reflect a selective mechanism to limit endosperm proliferation arising from such changes, even though larger seeds mean more successful offspring. Alternatively, selection against larger seeds in apomicts, which arise through polyploidy or changes to the EBN, may reflect resulting deleterious genome dosage imbalances in the highly-imprinted endosperm tissue.

## Data availability statement

All nucleotide sequence data is included in supplementary material. Newly obtained chloroplast sequences have been deposited in GenBank https://www.ncbi.nlm.nih.gov/genbank/, accession numbers OP763345-OP763384. Further inquiries can be directed to the corresponding author.

## Author contributions

DP and TFS conceived the study, and DP, TFS, JTL, SJ and MP planned the experiment. DP analysed all data. MP was responsible for logistical planning, seed collection and planting. RG performed flow cytometric analyses. DP and DG performed seed size analyses. DP, SJ and AF performed *phenoSeeder* analyses. DP and TFS wrote the manuscript.

## Funding

DP was supported by funding from the Swiss National Science Foundation (Project number: P2EZP3_152243). Experimental work was co-funded by a grant from the Global Institute of Food Security at the University of Saskatchewan to TS, and the operational funds of TS at the Leibniz Institute for Plant Genetics and Crop Plant Science (IPK Gatersleben).

## Acknowledgments

We thank Johanna Roussel for developing initial *phenoSeeder* protocols for *Boechera* spp.; Andres Posso-Terranova, Zahida Irin, Nazmul Hasan, and Heidi Block for help with greenhouse operations and plant care; Angie Li for laboratory assistance; Rongli Shi for advice on root scanning; Pierre-Luc Pradier for photography setup; Erik Schranz for seed material; Martin Mau and Liza Fomenko for DNA samples; Tuomas Hämälä and Jukka Jokela for advice on statistical methods. We acknowledge experiments which preceded this study and failed due to biological, technical, and unknown reasons.

## Conflict of interest

The authors declare that the research was conducted in the absence of any commercial or financial relationships that could be construed as a potential conflict of interest.

## Publisher’s note

All claims expressed in this article are solely those of the authors and do not necessarily represent those of their affiliated organizations, or those of the publisher, the editors and the reviewers. Any product that may be evaluated in this article, or claim that may be made by its manufacturer, is not guaranteed or endorsed by the publisher.

## Supplementary material

SUPPLEMENTARY TABLE 1 Information about Boechera accessions used in the study.

SUPPLEMENTARY TABLE 2 Alignment of chloroplast sequences used for haplotype definition. The trnL intron and the trnL-trnF intergenic spacer regions were assembled into one sequence (separated by three XXX) and missing bases at the 3' and 5' ends were substituted with N. Newly obtained sequences are aligned to the existing dataset (Kiefer et al., 2009)
