# Supplementary material for: Seed size, endosperm and germination variation in sexual and apomictic Boechera
Source: Front Plant Sci. 2022 Nov 18;13:991531. doi: 10.3389/fpls.2022.991531 (PMC9716183; doi:10.3389/fpls.2022.991531)
Supplement: SUPPLEMENTARY TABLE 2 — Alignment of chloroplast sequences used for haplotype definition. The trnL intron and the trnL-trnF intergenic spacer regions were assembled into one sequence (separated by three XXX) and missing bases at the 3' and 5' ends were substituted with N. Newly obtained sequences are aligned to the existing dataset (Kiefer et al., 2009) [file Table_2.docx]

#NEXUS

Begin data;

Dimensions ntax=270 nchar=1100;

Format datatype=DNA gap=- missing='N';

Matrix

AY257718 AATTGGATTGAGCCTTGGTATGGAAACCTACTAAGTGATAACTTTCAAATTCAGAGAAACCCTGGAATTAAcaatgggcAATCCTGAGCCAAATCCTGGTTTACGTGAACAAACCGGAGTTTAGAAAGCGAGAAAAAA-GGGATAGGTGCAGAGACTCAATGGAAGCTGTTCTAACAAATGGAGTTCACTACCTTGTGTTGATAAAGGAATCCTTCGATCAAAACTTCAAATCAAAAAG-GATGAAGGAGAAAAACCTATATTGTCTAAATATA------GGTAACACAAAA-CGATCTCAAAAATGACGACCTGAATCTCGATTTCTATTTTTTT--ATAAACAAAATCGAAATGATGTGAATCAATTCGAA-----GTTTAAGAAAtaatattcattGATCAAATGATTCTCTTCATAGTCTGATAGATCCGTGGTGGAActtattaaTCGGACGAGAATAAAGATAGAGTCCCATTTTAC-ATGTCAATACTGACAACAATGAAATTTATAGTAAGATGXXXGCTCTACT-CCCCAAAAAGGTCTGTTTGACACCTTACCTtttt-ttag-TTATTATCCA-TTTGAATTATTTAGAATCTATATCAGTTTTCATTTTCAAACTTAGAAAGTCTTCTTTTATTTATAAGATCCAAGAAATTCCCGGTCCAAAACTTTTTTAATTTACTACtttt------gagttTCTTTTCATTGACAGAGACCCAAGTCATATATTAAAATGATACTGATacttc--------------------------------------cgtaaTGGTCGGCATAGCTTAATTGCGGAGGACTGAAAATCCTTGTGTCACCATT--------------------------------------------------------------------------------------------AGTAAAATTATAC-TGATACTTCAGTAGATGATACCTCAGTAATGGTGGACATAGC-------TTTTTTG--CAGAGGACTGAAAATC------CTTGTGTCACCATTCGTAAAATGAGGATGATACTTCGGTAATGGCCGGGATAGCTCAgttg

AY257719 AATTGGATTGAGCCTTGGTATGGAAACCTACTAAGTGATAACTTTCAAATTCAGAGAAACCCTGGAATTAAcaatgggcAATCCTGAGCCAAATCCTGGTTTACGTGAACAAACCGGAGTTTAGAAAGCGAGAAAAAA-GGGATAGGTGCAGAGACTCAATGGAAGCTGTTCTAACAAATGGAGTTCACTACCTTGTGTTGATAAAGGAATCCTTCGATCAAAACTTCAAATCAAAAAG-GATGAAGGAGAAAAACCTATATTGTCTAAATATA------GGTAACACAAAA-CGATCTCAAAAATGACGACCTGAATCTCGATTTCTATTTTTTT--ATAAACAAAATCGAAATGATGTGAATCAATTCGAA-----GTTTAAGAAAtaatattcattGATCAAATGATTCTCTTCATAGTCTGATAGATCCGTGGTGGAActtattaaTCGGACGAGAATAAAGATAGAGTCCCATTTTAC-ATGTCAATACTGACAACAATGAAATTTATAGTAAGATGXXXGCTCTACT-CCCCAAAAAGGTCTGTTTGACACCTTACCTtttt-ttag-TTATTATCCA-TTTGAATTATTTAGAATCTATATCAGTTTTCATTTTCAAACTTAGAAAGTCTTCTTTTATTTATAAGATCCAAGAAATTCCCGGTCCAAAACTTTTTTAATTTACTACtttt------gagttTCTTTTCATTGACAGAGACCCAAGTCATATATTAAAATGATACTGATacttc--------------------------------------cgtaaTGGTCGGCATAGCTTAATTGCGGAGGACTGAAAATCCTTGTGTCACCATT--------------------------------------------------------------------------------------------AGTAAAATGATAC-TGATACTTCAGTAGATGATACCTCAGTAATGGTGGACATAGC-------TTTTTTG--CAGAGGACTGAAAATC------CTTGTGTCACCATTCGTAAAATGAGGATGATACTTCGGTAATGGCCGGGATAGCTCAgttg

AY257720 AATTGGATTGAGCCTTGGTATGGAAACCTACTAAGTGATAACTTTCAAATTCAGAGAAACCCTGGAATTAAcaatgggcAATCCTGAGCCAAATCCTGGTTTACGTGAACAAACCGGAGTTTAGAAAGCGAGAAAAAA-GGGATAGGTGCAGAGACTCAATGGAAGCTGTTCTAACAAATGGAGTTCACTACCTTGTGTTGATAAAGGAATCCTTCGATCAAAACTTCAAATCAAAAAG-GATGAAGGAGAAAAACCTATATTGTCTAAATATA------GGTAACACAAAA-CGATCTCAAAAATGACGACCTGAATCTCGATTTCTATTTTTTT--ATAAACAAAATCGAAATGATGTGAATCAATTCGAA-----GTTTAAGAAAtaatattcattGATCAAATGATTCTCTTCATAGTCTGATAGATCCGTGGTGGAActtattaaTCGGACGAGAATAAAGATAGAGTCCCATTTTAC-ATGTCAATACTGACAACAATGAAATTTATAGTAAGATGXXXGCTCTACT-CCCCAAAAAGGTCTGTTTGACACCTTACCTtttt-ttag-TTATTATCCA-TTTGAATTATTTAGAATCTATATCAGTTTTCATTTTCAAACTTAGAAAGTCTTCTTTTATTTATAAGATCCAAGAAATTCCCGGTCCAAAACTTTTTTAATTTACTACtttt------gagttTCTTTTCATTGACAGAGACCCAAGTCATATATTAAAATGATACTGATacttc--------------------------------------cgtaaTGGTCGGCATAGCTTAATTGCGGAGGACTGAAAATCCTTGTGTCACCATT--------------------------------------------------------------------------------------------AGTAAAATGATAC-TGATACTTCAGTAGATGATACCTCAGTAATGGTGGACATAGC-------TTTTTTG--CAGAGGACTGAAAATC------CTTGTGTCACCATTAGTAAAATGATACTGATACTTCGGTAATGGCCGGGATAGCTCAgttg

trnLF_FW AATTGGATTGAGCCTTGGTATGGAAACCTACTAAGTGATAACTTTCAAATTCAGAGAAACCCTGGAATTAACAATGGGCAATCCTGAGCCAAATCCTGGTTTACGTGAACAAACCGGAGTTTAGAAAGCGAGAAAAAA-GGGATAGGTGCAGAGACTCAATGGAAGCTGTTCTAACAAATGGAGTTCACTACCTTGTGTTGATAAAGGAATCCTTCGATCAAAACTTCAAATCAAAAAG-GATGAAGGAGAAAAACCTATATTGTCTAAATATA------GGTAACACAAAA-CGATCTCAAAAATGACGACCTGAATCTCGATTTCGATTTTGTTT-ATAAA-AAAATCGAAATGATGTGAATCAATTCGAA-----GTTTAAGAAATAATATTCATTGATCAAATGATTCTCTTCATAGTCTGATAGATCCGTGGTGGAACTTATTAATCGGACGAGAATAAAGATAGAGTCCCATTTTAC-ATGTCAATACTGACAACAATGAAATTTATAGTAAGATGXXXGCTCTACT-CCCCAAAAAGGTCTGTTTGACACCTTACCTTTTT-TTAG-TTATTATCCA-TTTGAATTATTTCGAATCTATATCAGTTTTCATTTTCAAACTTAGAAAGTCTTCTTTTATTTATAAGATGCAAGAAATTCCCGGTCCAAAACTTTTTGAATTTACTACTTTT------GAGTTTCTTTTCATTGACAGAGACCCAAGTCATATATTAAAATGATACTGATACTTC--------------------------------------CGTAATGGTCGGCATAGCTTAATTGCGGAGGACTGAAAATCCTTGTGTCACCATT--------------------------------------------------------------------------------------------AGTAAAATGATAA-TGATACTTCAGTAGATGATACCTCAGTAATGGTGGACATAGC-------TTTTTTG--CAGAGGACTGAAAATC------CTTGTGTCACCATTCGTAAAATGAGGATGATACTTCGGTAATGGCCGGGATAGCTCAgttg

trnLF_FY AATTGGATTGAGCCTTGGTATGGAAACCTACTAAGTGATAACTTTCAAATTCAGAGAAACCCTGGAATTAACAATGGGCAATCCTGAGCCAAATCCTGGTTTACGTGAACAAACCGGAGTTTAGAAAGCGAGAAAAAA-GGGATAGGTGCAGAGACTCAATGGAAGCTGTTCTAACAAATGGAGTTCACTACCTTGTGTTGATAAAGGAATCCTTCGATCAAAACTTCAAATCAAAAAG-GATGAAGGAGAAAAACCTATATTGTCTAAATATA------GGTAACACAAAA-CGATCTCAAAAATGACGACCTGAATCTCGATTTCTATTTTTTT--ATAAACAAAATCGAAATGATGTGAATCAATTCGAA-----GTTTAAGAAATAATATTCATTGATCAAATGATTCTCTTCATAGTCTGATAGATCCGTGGTGGAACTTATTAATCGGACGAGAATAAAGATAGAGTCCCATTTTAC-ATGTCAATACTGACAACAATGAAATTTATAGTAAGATGXXXGCTCTACT-CCCCAAAAAGGTCTGTTTGACACCTTACCTTTTT-TTAG-TTATTATCCA-TTTGAATTATTTAGAATCTATATCAGTTTTCATTTTCAAACTTAGAAAGTCTTCTTTTATTTATAAGATCCAAGAAATTCCCGGTCCAAAACTTTTTGAATTTACTACTTTT------GAGTTTCTTTTCATTGACAGAGACCCAAGTCATATATTAAAATGATACTGATACTTC--------------------------------------CGTAATGGTCGGCATAGCTTAATTGCGGAGGACTGAAAATCCTTGTGTCACCATT--------------------------------------------------------------------------------------------AGTAAAATGATAA-TGATACTTCAGTAGATGATACCTCAGTAATGGTGGACATAGC-------TTTTTTG--CAGAGGACTGAAAATC------CTTGTGTCCTCATTCGTAAAATGAGGATGATACTTCGGTAATGGCCGGGATAGCTCAgttg

trnLF_GE AATTGGATTGAGCCTTGGTATGGAAACCTACTAAGTGATAACTTTCAAATTCAGAGAAACCCTGGAATTAAcaatgggcAATCCTGAGCCAAATCCTGGTTTACGTGAACAAACCGGAGTTTAGAAAGCGAGAAAAAA--GGATAGGTGCAGAGACTCAATGGAAGCTGTTCTAACAAATGGAGTTCACTACCTTGTGTTGATAAAGGAATCCTTCGATCAAAACTTCAAATCAAAAAG-GATGAAGGAGAAAAACCTATATTGTCTAAATATA------GGTAACACAAAA-CGATCTCAAAAATGACGACCTGAATCTCGATTTCTATTTTTTT--ATAAACAAAATCGAAATGATGTGAATCAATTCGAA-----GTTTAAGAAAtaatattcattGATCAAATGATTCTCTTCATAGTCTGATAGATCCGTGGTGGAActtattaaTCGGACGAGAATAAAGATAGAGTCCCATTTTAC-ATGTCAATACTGACAACAATGAAATTTATAGTAAGATGXXXGCTCTACT-CCCCAAAAAGGTCTGTTTGACACCTTACCTtttt-ttag-TTATTATCCA-TTTGAATTATTTAGAATCTATATCAGTTTTCATTTTCAAACTTAGAAAGTCTTCTTTTATTTATAAGATCCAAGAAATTCCCGGTCCAAAACTTTTTTAATTTACTACtttt------gagttTCTTTTCATTGACAGAGACCCAAGTCATATATTAAAATGATACTGATacttc--------------------------------------cgtaaTGGTCGGCATAGCTTAATTGCGGAGGACTGAAAATCCTTGTGTCACCATT--------------------------------------------------------------------------------------------AGTAAAATGATAC-TGATACTTCAGTAGATGATACCTCAGTAATGGTGGACATAGC-------TTTTTTG--CAGAGGACTGAAAATC------CTTGTGTCACCATTCGTAAAATGAGGATGATANNNNNNNNNNNNNNNNNNNNNNNNNnnnn

trnLF_GF NNNNNNNNNNNNNNNNNNNNNNNNNNNNNNNNNNGTGATAACTTTCAAATTCAGAGAAACCCTGGAATTAAcaatgggcAATCCTGAGCCAAATCCTGGTTTACGTGAACAAACCGGAGTTTAGAAAGCGAGAAAAAA--GGATAGGTGCAGAGACTCAATGGAAGCTGTTCTAACAAATGGAGTTCACTACCTTGTGTTGATAAAGGAATCCTTCGATCAAAACTTCAAATCAAAAAG-GATGAAGGAGAAAAACCTATATTGTCTAAATATA------GGTAACACAAAA-CGATCTCAAAAATGACGACCTGAATCTCGATTTCTATTTTTTT--ATAAACAAAATCGAAATGATGTGAATCAATTCGAA-----GTTTAAGAAAtaatattcattGATCAAATGATTCTCTTCATAGTCTGATAGATCCGTGGTGGAActtattaaTCGGACGAGAATAAAGATAGAGTCCCATTTTAC-ATGTCAATACTGACAACAATGAAATTTATAGTAAGATGXXXGCTCTACT-CCCCAAAAAGGTCTGTTTGACACCTTACCTtttt-ttag-TTATTATCCA-TTTGAATTATTTAGAATCTATATCAGTTTTCATTTTCAAACTTAGAAAGTCTTCTTTTATTTATAAGATCCAAGAAATTCCCGGTCCAAAACTTTTTTAATTTACTACtttt------gagttTCTTTTCATTGACAGAGACCCAAGTCATATATTAAAATGATACTGATacttc--------------------------------------cgtaaTGGTCGGCATAGCTTAATTGCGGAGGACTGAAAATCCTTGTGTCACCATT--------------------------------------------------------------------------------------------AGTAAAATGATAC-TGATACTTCAGTAGATGATACCTCAGTAATGGTGGACATAGC-------TTTTTTG--CAGAGGACTGAAAATC------CTTGTGTCACCATTAGTAAAATGATACTGATACNNNNNNNNNNNNNNNNNNNNNNNNnnnn

trnLF_GM NNNNNNNNNNNNNNNNNNNNNNNNNNCCTACTAAGTGATAACTTTCAAATTCAGAGAAACCCTGGAATTAAcaatgggcAATCCTGAGCCAAATCCTGGTTTACGTGAACAAACCGGAGTTTAGAAAGCGAGAAAAA--GGGATAGGTGCAGAGACTCAATGGAAGCTGTTCTAACAAATGGAGTTCACTACCTTGTGTTGATAAAGGAATCCTTCGATCAAAACTGCAAATAAAAAAG-GATGAAGGAGAAAAACCTATATTGTCTAAATATA------GGTAACACAAAA-CGATCTCAAAAATGACGACCTGAATCTCGATTTCTCTTTTTTT--ATAAACAAAATCGAAATGATGTGAATCAATTCGAA-----GTTTAAGAAAtaatattcattGATCAAATGATTCTCTTCATAGTCTGATAGATCCGTGGTGGAActtattaaTCGGCCGAGAATAAAGATAGAGTCCCATTTTAC-ATGTCAATACTGACAACAATGAAATTTATAGTAAGATGXXXGCTCTACT-CCCCAAAAAGGTCTGTTTGACACCTTACCTTTTT-TTAG-TTATTATCCA-TTTGAATTATTTAGAATCTATATCTGTTTTCATTTTCAAACTTAGAAAGTCTTCTTTTATTTATAAGATCCAAGAAATTCCCGGTCCAAAACTTTTTTTATTTACTACTTTTTATTTTGAGTTTCTTTTCATTGACAGAGACCCAAGTCATATATTAAAATGATACTGATacttc--------------------------------------cgtaaTGGTCGGCATAGCTTAATTGCGGAGGACTGAAAATCCTTGTGTCACCATT--------------------------------------------------------------------------------------------------------------------------------------------------------------------------------------------------------AGTAAAATGATACTGATACTTCGGTAATGGCCGGGATAGCTCAgttg

AY257692 AATTGGATTGAGCCTTGGTATGGAAACCTACTAAGTGATAACTTTCAAATTCAGAGAAACCCTGGAATTAAcaatgggcAATCCTGAGCCAAATCCTGGTTTACGTGAACAAACCGGAGTTTAGAAAGCGAGAAAAAA-GGGATAGGTGCAGAGACTCAATGGAAGCTGTTCTAACAAATGGAGTTCACTACCTTGTGTTGATAAAGGAATCCTTCGATCAAAACTTCAAATCAAAAAG-GATGAAGGAGAAAAACCTATATTGTCTAAATATA------GGTAACACAAAA-CGATCTCAAAAATGACGACCTGAATCTCGATTTCTATTTTTTT--ATAAACAAAATCGAAATGATGTGAATCAATTCGAA-----GTTTAAGAAAtaatattcattGATCAAATGATTCTCTTCATAGTCTGATAGATCCGTGGTGGAActtattaaTCGGACGAGAATAAAGATAGAGTCCCATTTTAC-ATGTCAATACTGACAACAATGAAATTTATAGTAAGATGXXXGCTCTACT-CCCCAAAAAGGTCTGTTTGACACCTTACCTtttt-ttag-TTATTATCGA-TTTGAATTATTTAGAATCTATATCAGTTTTCATTTTCAAACTTAGAAAGTCTTCTTTTATTTATAAGATCCAAGAAATTCCCGGTCCAAAACTTTTTTAATTTACTACtttt------gagttTCTTTTCATTGACAGAGACCCAAGTCATATATTAAAATGATACTGATacttc--------------------------------------cgtaaTGGTCGGCATAGCTTAATTGCCGAGGACTGAAAATCCTTGTGTCACCATT--------------------------------------------------------------------------------------------AGTAAAATGCGAA-TGATACTTCAGTAGATGATACCTCAGTAATGGTGGACATAGC-------TTTTTTG--CAGAGGACTGAAAATC------CTTGTGTCACCATTCGTAAAATGAGGATGATACTTCGGTAATGGCCGGGATAGCTCAgttg

AY257693 AATTGGATTGAGCCTTGGTATGGAAACCTACTAAGTGATAACTTTCAAATTCAGAGAAACCCTGGAATTAAcaatgggcAATCCTGAGCCAAATCCTGGTTTACGTGAACAAACCGGAGTTTAGAAAGCGAGAAAAAA-GGGATAGGTGCAGAGACTCAATGGAAGCTGTTCTAACAAATGGAGTTCACTACCTTGTGTTGATAAAGGAATCCTTCGATCAAAACTTCAAATCAAAAAG-GATGAAGGAGAAAAACCTATATTGTCTAAATATA------GGTAACACAAAA-CGATCTCAAAAATGACGACCTGAATCTCGATTTCTATTTTTTT--ATAAACAAAATCGAAATGATGTGAATCAATTCGAA-----GTTTAAGAAAtaatattcattGATCAAATGATTCTCTTCATAGTCTGATAGATCCGTGGTGGAActtattaaTCGGACGAGAATAAAGATAGAGTCCCATTTTAC-ATGTCAATACTGACAACAATGAAATTTATAGTAAGATGXXXNCTCTACT-CCCCAAAAAGGTCTGTTTGACACCTTACCTtttt-ttag-TTATTATCGA-TTTGAATTATTTAGAATCTATATCAGTTTTCATTTTCAAACTTAGAAAGTCTTCTTTTATTTATAAGATCCAAGAAATTCCCGGTCCAAAACTTTTTTAATTTACTACtttt------gagttTCTTTTCATTGACAGAGACCCAAGTCATATATTAAAATGATACTGATacttc--------------------------------------cgtaaTGGTCGGCATAGCTTAATTGCGGAGGACTGAAAATCCTTGTGTCACCATT--------------------------------------------------------------------------------------------AGTAAAATGAGAA-TGATACTTCAGTAGATGATACCTCAGTAATGGTGGACATAGC--TTTTTTTTTTTG--CAGAGGACTGAAAATC------CTTGTGTCACCATTCGTAAAATGAGGATGATACTTCGGTAATGGCCGGGATAGCTCAgttg

AY257694 AATTGGATTGAGCCTTGGTATGGAAACCTACTAAGTGATAACTTTCAAATTCAGAGAAACCCTGGAATTAAcaatgggcAATCCTGAGCCAAATCCTGGTTTACGTGAACAAACCGGAGTTTAGAAAGCGAGAAAAAA-GGGATAGGTGCAGAGACTCAATGGAAGCTGTTCTAACAAATGGAGTTCACTACCTTGTGTTGATAAAGGAATCCTTCGATCAAAACTTCAAATCAAAAAG-GATGAAGGAGAAAAACCTATATTGTCTAAATATA------GGTAACACAAAA-CGATCTCAAAAATGACGACCTGAATCTCGATTTCTATTTTTTT--ATAAACAAAATCGAAATGATGTGAATCAATTCGAA-----GTTTAAGAAAtaatattcattGATCAAATGATTCTCTTCATAGTCTGATAGATCCGTGGTGGAActtattaaTCGGACGAGAATAAAGATAGAGTCCCATTTTAC-ATGTCAATACTGACAACAATGAAATTTATAGTAAGATGXXXNCTCTACT-CCCCAAAAAGGTCTGTTTGACACCTTACCTtttt-ttag-TTATTAGCGA-TTTGAATTATTTAGAATCTATATCAGTTTTCATTTTCAAACTTAGAAAGTCTTCTTTTATTTATAAGATCCAAGAAATTCCCGGTCCAAAACTTTTTTAATTTACTACtttt------gagttTCTTTTCATTGACAGAGACCCAAGTCATATATTAAAATGATACTGATacttc--------------------------------------cgtaaTGGTCGGCATAGCTTAATTGCGGAGGACTGAAAATCCTTGTGTCACCATT--------------------------------------------------------------------------------------------AGTAAAATGAGAA-TGATACTTCAGTAGATGATACCTCAGTAATGGTGGACATAGC--TTTTTTTTTTTG--CAGAGGACTGAAAATC------CTTGTGTCACCATTCGTAAAATGAGGATGATACTTCGGTAATGGCCGGGATAGCTCAgttg

AY257695 AATTGGATTGAGCCTTGGTATGGAAACCTACTAAGTGATAACTTTCAAATTCAGAGAAACCCTGGAATTAAcaatgggcAATCCTGAGCCAAATCCTGGTTTACGTGAACAAACCGGAGTTTAGAAAGCGAGAAAAAA-GGGATAGGTGCAGAGACTCAATGGAAGCTGTTCTAACAAATGGAGTTCACTACCTTGTGTTGATAAAGGAATCCTTCGATCAAAACTTCAAATCAAAAAG-GATGAAGGAGAAAAACCTATATTGTCTAAATATA------GGTAACACAAAA-CGATCTCAAAAATGACGACCTGAATCTCGATTTCTATTTTTTT--ATAAACAAAATCGAAATGATGTGAATCAATTCGAA-----GTTTAAGAAAtaatattcattGATCAAATGATTCTCTTCATAGTCTGATAGATCCGTGGTGGAActtattaaTCGGACGAGAATAAAGATAGAGTCCCATTTTAC-ATGTCAATACTGACAACAATGAAATTTATAGTAAGATGXXXNNNNNNCT-CCCCAAAAAGGTCTGTTTGACACCTTACCTtttt-ttag-TTATTATCGA-TTTGAATTATTTAGAATCTATATCAGTTTTCATTTTCAAACTTAGAAAGTCTTCTTTTATTTATAAGATCCAAGAAATTCCCGGTCCAAAACTTTTTTCATTTACTACtttt------gagttTCTTTTCATTGACAGAGACCCAAGTCATATATTAAAATGATACTGATacttc--------------------------------------cgtaaTGGTCGGCATAGCTTAATTGCGGAGGACTGAAAATCCTTGTGTCACCATT--------------------------------------------------------------------------------------------AGTAAAATGAGAA-TGATACTTCAGTAGATGATACCTCAGTAATGGTGGACATAGC--TTTTTTTTTTTG--CGGAGGACTGAAAATC------CTTGTGTCACCATTCGTAAAATGAGGATGATACTTCGGTAATGGCCGGGATAGCTCAGTNn

AY257696 AATTGGATTGAGCCTTGGTATGGAAACCTACTAAGTGATAACTTTCAAATTCAGAGAAACCCTGGAATTAAcaatgggcAATCCTGAGCCAAATCCTGGTTTACGTGAACAAACCGGAGTTTAGAAAGCGAGAAAAAA-GGGATAGGTGCAGAGACTCAATGGAAGCTGTTCTAACAAATGGAGTTCACTACCTTGTGTTGATAAAGGAATCCTTCGATCAAAACTTCAAATCAAAAAG-GATGAAGGAGAAAAACCTATATTGTCTAAATATA------GGTAACACAAAA-CGATCTCAAAAATGACGACCTGAATCTCGATTTCTATTTTTTT--ATAAACAAAATCGAAATGATGTGAATCAATTCGAA-----GTTTAAGAAAtaatattcattGATCAAATGATTCTCTTCATAGTCTGATAGATCCGTGGTGGAActtattaaTCGGACGAGAATAAAGATAGAGTCCCATTTTAC-ATGTCAATACTGACAACAATGAAATTTATAGTAAGATGXXXGCTCTACT-CCCCAAAAAGGTCTGTTTGACACCTTACCTtttt-ttag-TTATTATCGA-TTTGAATTATTTAGAATCTATATCAGTTTTCATTTTCAAACTTAGAAAGTCTTCTTTTATTTATAAGATCCAAGAAATTCCCGGTCCAAAACTTTTTTAATTTACTACtttt------gagttTCTTTTCATTGACAGAGACCCAAGTCATATATTAAAATGATACTGATacttc--------------------------------------cgtaaTGGTCGGCATAGCTTAATTGCGGAGGACTTAAAATCCTTGTGTCACCATT--------------------------------------------------------------------------------------------AGTAAAATGAGAA-TGATACTTCAGTAGATGATACCTCAGTAATGGTGGACATAGC--TTTTTTTTTTTG--CAGAGGACTGAAAATC------CTTGTGTCACCATTCGTAAAATGAGGATGATACTTCGGTAATGGCCGGGATAGCTCAGTTn

AY257697 AATTGGATTGAGCCTTGGTATGGAAACCTACTAAGTGATAACTTTCAAATTCAGAGAAACCCTGGAATTAAcaatgggcAATCCTGAGCCAAATCCTGGTTTACGTGAACAAACCGGAGTTTAGAAAGCGAGAAAAAA-GGGATAGGTGCAGAGACTCAATGGAAGCTGTTCTAACAAATGGAGTTCACTACCTTGTGTTGATAAAGGAATCCTTCGATCAAAACTTCAAATCAAAAAG-GATGAAGGAGAAAAACCTATATTGTCTAAATATA------GGTAACACAAAA-CGATCTCAAAAATGACGACCTGAATCTCGATTTCTATTTTTTT--ATAAACAAAATCGAAATGATGTGAATCAATTCGAA-----GTTTAAGAAAtaatattcattGATCAAATGATTCTCTTCATAGTCTGATAGATCCGTGGTGGAActtattaaTCGGACGAGAATAAAGATAGAGTCCCATTTTAC-ATGTCAATACTGACAACAATGAAATTTATAGTAAGATGXXXGCTCTACT-CCCCAAAAAGGTCTGTTTGACACCTTACCTtttt-ttag-TTATTATCGA-TTTGAATTATTTAGAATCTATATCAGTTTTCATTTTCAAACTTAGAAAGTCTTCTTTGATTTATAAGATCCAAGAAATTCCCGGTCCAAAACTTTTTTAATTTACTACtttt------gagttTCTTTTCATTGACAGAGACCCAAGTCATATATTAAAATGATACTGATacttc--------------------------------------cgtaaTGGTCGGCATAGCTTAATTGCGGAGGACTGAAAATCCTTGTGTCACCATT--------------------------------------------------------------------------------------------AGTAAAATGAGAA-TGATACTTCAGTAGATGATACCTCAGTAATGGTGGACATAGC--TTTTTTTTTTTG--CAGAGGACTGAAAATC------CTTGTGTCAC--------AAATGAGGATGATACTTCGGTAATGGCCGGGATAGCTCAgttg

AY257698 NNNNNNNNNNNNNNNNNNNATGGAAACCTACTAAGTGATAACTTTCAAATTCAGAGAAACCCTGGAATTAAcaatgggcAATCCTGAGCCAAATCCTGGTTTACGTGAACAAACCGGAGTTTAGAAAGCGAGAAAAAA-GGGATAGGTGCAGAGACTCAATGGAAGCTGTTCTAACAAATGGAGTTCACTACCTTGTGTTGATAAAGGAATCCTTCGATCAAAACTTCAAATCAAAAAG-GATGAAGGAGAAAAACCTATATTGTCTAAATATA------GGTAACACAAAA-CGATCTCAAAAATGACGACCTGAATCTCGATTTCTATTTTTTT--ATAAACAAAATCGAAATGATGTGAATCAATTCGAA-----GTTTAAGAAAtaatattcattGATCAAATGATTCTCTTCATAGTCTGATAGATCCGTGGTGGAActtattaaTCGGACGAGAATAAAGATAGAGTCCCATTTTAC-ATGTCAATACTGACAACAATGAAATTTATAGTAAGATGXXXGCTCTACT-CCCCAAAAAGGTCTGTTTGACACCTTACCTtttt-ttag-TTATTATCGA-TTTGAATTATTTAGAATCTATATCAGTTTTCATTTTCAAACTTAGAAAGTCTTCTTTTATTTATAAGATCCAAGAAATTCCCGGTCCAAAACTTTTTTAATTTACTACtttt------gagttTCTTTTCATTGACAGAGACCCAAGTCATATATTAAAATGATACTGATacttc--------------------------------------cgtaaTGGTCGGCATAGCTTAATTGCGGAGGACTGAAAATCCTTGTGTCACCATT--------------------------------------------------------------------------------------------AGTAAAATGAGAA-TGATACTTCAGTAGATGATACCTCAGTAATGGTGGACATAGC--TTTTTTTTTGTG--CAGAGAACTGAAAATC------CTTGTGTCACCATTCGTAAAATAAGGATGATACTTCGGTAATGGCCGGGATAGCTCAgttg

AY257699 AATTGGATTGAGCCTTGGTATGGAAACCTACTAAGTGATAACTTTCAAATTCAGAGAAACCCTGGAATTAAcaatgggcAATCCTGAGCCAAATCCTGGTTTACGTGAACAAACCGGAGTTTAGAAAGCGAGAAAAAA-GGGATAGGTGCAGAGACTCAATGGAAGCTGTTCTAACAAATGGAGTTCACTACCTTGTGTTGATAAAGGAATCCTTCGATCAAAACTTCAAATCAAAAAG-GATGAAGGAGAAAAACCTATATTGTCTAAATATA------GGTAACACAAAA-CGATCTCAAAAATGACGACCTGAATCTCGATTTCTATTTTTTT--ATAAACAAAATCGAAATGATGTGAATCAATTCGAA-----GTTTAAGAAAtaatattcattGATCAAATGATTCTCTTCATAGTCTGATAGATCCGTGGTGGAActtattaaTCGGACGAGAATAAAGATAGAGTCCCATTTTAC-ATGTCAATACTGACAACAATGAAATTTATAGTAAGATGXXXGCTCTACT-CCCCAAAAAGGTCTGTTTGACACCTTACCTtttt-ttag-TTATTATCGA-TTTGAATTATTTAGAATCTATATCAGTTTTCATTTTCAAACTTAGAAAGTCTTCTTTTATTTATAAGATCCAAGAAATTCCCGGTCCAAAACTTTTTTAATTTACTACtttt------gagttTCTTTTCATTGACAGAGACCCAAGTCATATATTAAAATGATACTGATacttc--------------------------------------cgtaaTGGTCGGCATAGCTTAATTGCGGAGGACTGAAAATCCTTGTGTCACCATT--------------------------------------------------------------------------------------------AGTAAAATGAGAG-TGATACTTCAGTAGATGATACCTCAGTAATGGTGGACATAGC--TTTTTTTTTGTG--CAGAGGACTGAAAATC------CTTGTGTCACCATTCGTAAAATAAGGATGATACTTCGGTAATGGCCGGGATAGCTCAgttg

AY257700 AATTGGATTGAGCCTTGGTATGGAAACCTACTAAGTGATAACTTTCAAATTCAGAGAAACCCTGGAATTAAcaatgggcAATCCTGAGCCAAATCCTGGTTTACGTGAACAAACCGGAGTTTAGAAAGCGAGAAAAAA-GGGATAGGTGCAGAGACTCAATGGAAGCTGTTCTAACAAATGGAGTTCACTACCTTGTGTTGATAAAGGAATCCTTCGATCAAAACTTCAAATCAAAAAG-GATGAAGGAGAAAAACCTATATTGTCTAAATATA------GGTAACACAAAA-CGATCTCAAAAATGACGACCTGAATCTCGATTTCTATTTTTTT--ATAAACAAAATCGAAATGATGTGAATCAATTCGAA-----GTTTAAGAAAtaatattcattGATCAAATGATTCTCTTCATAGTCTGATAGATCCGTGGTGGAActtattaaTCGGACGAGAATAAAGATAGAGTCCCATTTTAC-ATGTCAATACTGACAACAATGAAATTTATAGTAAGATGXXXGCTCTACT-CCCCAAAAAGGTCTGTTTGACACCTTACCTtttt-ttag-TTATTATCGA-TTTGAATTATTTAGAATCTATATCAGTTTTCATTTTCAAACTTAGAAAGTCTTCTTTTATTTATAAGATCCAAGAAATTCCCGGTCCAAAACTTTTTTAATTTACTACtttt------gagttTCTTTTCATTGACAGAGACCCAAGTCATATATTAAAATGATACTGATacttc--------------------------------------cgtaaTGGTCGGCATAGCTTAATTGCGGAGGACTGAAAATCCTTGTGTCACCATT--------------------------------------------------------------------------------------------AGGAAAATGAGAA-TGATACTTCAGTAGATGATACCTCAGTAATGGTGGACATAGC--TTTTTTTTTGTG--CAGAGGACTGAAAATC------CTTGTGTCACCATTCGTAAAATAAGGATGATACTTCGGTAATGGCCGGGATAGCTCAgttg

AY257701 AATTGGATTGAGCCTTGGTATGGAAACCTACTAAGTGATAACTTTCAAATTCAGAGAAACCCTGGAATTAAcaatgggcAATCCTGAGCCAAATCCTGGTTTACGTGAACAAACCGGAGTTTAGAAAGCGAGAAAAAA-GGGATAGGTGCAGAGACTCAATGGAAGCTGTTCTAACAAATGGAGTTCACTACCTTGTGTTGATAAAGGAATCCTTCGATCAAAACTTCAAATCAAAAAG-GATGAAGGAGAAAAACCTATATTGTCTAAATATA------GGTAACACAAAA-CGATCTCAAAAATGACGACCTGAATCTCGATTTCTATTTTTTT--ATAAACAAAATCGAAATGATGTGAATCAATTCGAA-----GTTTAAGAAAtaatattcattGATCAAATGATTCTCTTCATAGTCTGATAGATCCGTGGTGGAActtattaaTCGGACGAGAATAAAGATAGAGTCCCATTTTAC-ATGTCAATACTGACAACAATGAAATTTATAGTAAGATGXXXGCTCTACT-CCCCAAAAAGGTCTGTTTGACACCTTACCTtttt-ttag-TTATTATCGA-TTTGAATTATTTAGAATCTATATCAGTTTTCATTTTCAAACTTAGAAAGTCTTCTTTTATTTATAAGATCCAAGAAATTCCCGGTCCAAAACTTTTTTAATTTACTACtttt------gagttTCTTTTCATTGACAGAGACCCAAGTCATATATTAAAATGATACTGATacttc--------------------------------------cgtaaTGGTCGGCATAGCTTAATTGCGGAGGACTGAAAATCCTTGTGTCACCATT--------------------------------------------------------------------------------------------AGTAAAATGAGAA-TGATACTTCAGTAGATGATACCTCAGTAATGGTGGACATAGC--TTTTTTTTTGTG--CAGAGGACTGAAAATC------CTTGTGTCCCCATTCGTAAAATAAGGATGATACTTCGGTAATGGCCGGGATAGCTCAGTNn

AY257702 AATTGGATTGAGCCTTGGTATGGAAACCTACTAAGTGATAACTTTCAAATTCAGAGAAACCCTGGAATTAAcaatgggcAATCCTGAGCCAAATCCTGGTTTACGTGAACAAACCGGAGTTTAGAAAGCGAGAAAAAA-GGGATAGGTGCAGAGACTCAATGGAAGCTGTTCTAACAAATGGAGTTCACTACCTTGTGTTGATAAAGGAATCCTTCGATCAAAACTTCAAATCAAAAAG-GATGAAGGAGAAAAACCTATATTGTCTAAATATA------GGTAACACAAAA-CGATCTCAAAAATGACGACCTGAATCTCGATTTCTATTTTTTT--ATAAACAAAATCGAAATGATGTGAATCAATTCGAA-----GTTTAAGAAAtaatattcattGATCAAATGATTCTCTTCATAGTCTGATAGATCCGTGGTGGAActtattaaTCGGACGAGAATAAAGATAGAGTCCCATTTTAC-ATGTCAATACTGACAACAATGAAATTTATAGTAAGATGXXXGCTCTACT-CCCCAAAAAGGTCTGTTTGACACCTTACCTtttt-ttag-TTATTATCGA-TTTGAATTATTTAGAATCTATATCAGTTTTCATTTTCAAACTTAGAAAGTCTTCTTTTATTTATAAGATCCAAGAAATTCCCGGTCCAAAACTTTTTTAATTTACTACtttt------gagttTCTTTTCATTGACAGAGACCCAAGTCATATATTAAAATGATACTGATacttc--------------------------------------cgtaaTGGTCGGCATAGCTTAATTGCGGAGGACTGAAAATCCTTGTGTCACCATT--------------------------------------------------------------------------------------------AGTAAAATGAGAA-TGATACTTCAGTAGATGATACCTCAGTAATGGTGGACATAGC--TTTTTTTTTGTG--CAGAGGACTGAAAATC------CTTGTGTCACCATTCGTAAAATAAGGATGATACTTCGGTAATGGCCGGGATAGCTCAGTTn

AY257703 NNNNNNNNNNNGCCTTGGTATGGAAACCTACTAAGTGATAACTTTCAAATTCAGAGAAACCCTGGAATTAAcaatgggcAATCCTGAGCCAAATCCTGGTTTACGTGAACAAACCGGAGTTTAGAAAGCGAGAAAAAA-GGGATAGGTGCAGAGACTCAATGGAAGCTGTTCTAACAAATGGAGTTCACTACCTTGTGTTGATAAAGGAATCCTTCGATCAAAACTTCAAATCAAAAAG-GATGAAGGAGAAAAACCTATATTGTCTAAATATA------GGTAACACAAAA-CGATCTCAAAAATGACGACCTGAATCTCGATTTCTATTTTTTT--ATAAACAAAATCGAAATGATGTGAATCAATTCGAA-----GTTTAAGAAAtaatattcattGATCAAATGATTCTCTTCATAGTCTGATAGATCCGTGGTGGAActtattaaTCGGACGAGAATAAAGATAGAGTCCCATTTTAC-ATGTCAATACTGACAACAATGAAATTTATAGTAAGATGXXXGCTCTACT-CCCCAAAAAGGTCTGTTTGACACCTTBCCTtttt-ttag-TTATTATCGA-TTTGAATTATTTAGAATCTATATCAGTTTTCATTTTCAAACTGAGAAAGTCTTCTTTTATTTATAAGATCCAAGAAATTCCCGGTCCAAAACTTTTTTAATTTACTACtttt------gagttTCTTTTCATTGACAGAGACCCAAGTCATATATTAAAATGATACTGATacttc--------------------------------------cgtaaTGGTCGGCATAGCTTAATTGCGGAGGACTGAAAATCCTTGTGTCACCATT--------------------------------------------------------------------------------------------AGTAAAATGAGAA-TGATACTTCAGTAGATGATACCTCAGTAATGGTGGACATAGC--TTTTTTTTTGTG--CAGAGGACTGAAAATC------CTTGTGTCACCATTCGTAAAATAAGGATGATACTTCGGTAATGGCCGGGATAGCTCAGTNn

AY257704 NNNNNNNNNNNNCCTTGGTATGGNAACCTACTAAGTGATAACTTTCAAATTCAGAGAAACCCTGGANTTAAcaatgggcAATCCTGAGCCAAATCCTGGTTTACGTGAACAAACCGGAGTTTAGAAAGCGAGAAAAAA-GGGATAGGTGCAGAGACTCAATGGAAGCTGTTCTAACAAATGGAGTTCACTACCTTGTGTTGATAAAGGAATCCTTCGATCAAAACTTCAAATCAAAAAG-GATGAAGGAGAAAAACCTATATTGTCTAAATATA------GGTAACACAAAA-CGATCTCAAAAATGACGACCTGAATCTCGATTTCTATTTTTTT--ATAAACAAAATCGAAATGATGTGAATCAATTCGAA-----GTTTAAGAAAtaatattcattGATCAAATGATTCTCTTCATAGTCTGATAGATCCGTGGTGGAActtattaaTCGGACGAGAATAAAGATAGAGTCCCATTTTAC-ATGTCAATACTGACAACAATGAAATTTATAGTAAGATGXXXGCTCTACT-CCCCAAAAAGGTCTGTTTGACACCTTACCTtttt-ttag-TTATTATCGA-TTTGAATTATTTAGAATCTATATCAGTTTTCATTTTCAAACTTAGAAAGTCTTCTTTTATTTATAAGATCCAAGAAATTCCCGGTCCAAAACTTTTTTAATTTACTACtttt------gagttTCTTTTCATTGACAGAGACCCAAGTCATATATTAAAATGATACTGATacttc--------------------------------------cgtaaTGGTCGGCATAGCTTAATTGCGGAGGACTGAAAATCCTTGTGTCACCATT--------------------------------------------------------------------------------------------------------------------------------------------------------------------------------------------------------CGTAAAATAAGGATGATACTTCGGTAATGGCCGGGATAGCTCAgttg

AY257705 AATTGGATTGAGCCTTGGTATGGAAACCTACTAAGTGATAACTTTCAAATTCAGAGAAACCCTGGAATTAAcaatgggcAATCCTGAGCCAAATCCTGGTTTACGTGAACAAACCGGAGTTTAGAAAGCGAGAAAAAA-GGGATAGGTGCAGAGACTCAATGGAAGCTGTTCTAACAAATGGAGTTCACTACCTTGTGTTGATAAAGGAATCCTTCGATCAAAACTTCAAATCAAAAAG-GATGAAGGAGAAAAACCTATATTGTCTAAATATA------GGTAACACAAAA-CGATCTCAAAAATGACGACCTGAATCTCGATTTCTATTTTTTT--ATAAACAAAATCGAAATGATGTGAATCAATTCGAA-----GTTTAAGAAAtaatattcattGATCAAATGATTCTCTTCATAGTCTGATAGATCCGTGGTGGAActtattaaTCGGACGAGAATAAAGATAGAGTCCCATTTTAC-ATGTCAATACTGACAACAATGAAATTTATAGTAAGATGXXXGCTCTACT-CCCCAAAAAGGTCTGTTTGACACCTTACCTtttt-ttag-TTATTATCGA-TTTGAATTATTTTGAATCTATATCAGTTTTCATTTTCAAACTTAGAAAGTCTTCTTTTATTTATAAGATCCAAGAAATTCCCGGTCCAAAACTTTTTTAATTTACTACtttt------gagttTCTTTTCATTGACAGAGACCCAAGTCATATATTAAAATGATACTGATacttc--------------------------------------cgtaaTGGTCGGCATAGCTTAATTGCGGAGGACTGAAAATCCTTGTGTCACCATT--------------------------------------------------------------------------------------------------------------------------------------------------------------------------------------------------------CGTAAAATAAGGATGATACTTCGGTAATGGCCGGGATAGCTCAgttg

AY257706 AATTGGATTGAGCCTTGGTATGGAAACCTACTAAGTGATAACTTTCAAATTCAGAGAAACCCTGGAATTAAcaatgggcAATCCTGAGCCAAATCCTGGTTTACGTGAACAAACCGGAGTTTAGAAAGCGAGAAAAAA-GGGATAGGTGCAGAGACTCAATGGAAGCTGTTCTAACAAATGGAGTTCACTACCTTGTGTTGATAAAGGAATCCTTCGATCAAAACTTCAAATCAAAAAG-GATGAAGGAGAAAAACCTATATTGTCTAAATATA------GGTAACACAAAA-CGATCTCAAAAATGACGACCTGAATCTCGATTTCTATTTTTTT--ATAAACAAAATCGAAATGATGTGAATCAATTCGAA-----GTTTAAGAAAtaatattcattGATCAAATGATTCTCTTCATAGTCTGATAGATCCGTGGTGGAActtattaaTCGGACGAGAATAAAGATAGAGTCCCATTTTAC-ATGTCAATACTGACAACAATGAAATTTATAGTAAGATGXXXGCTCTACT-CCCCAAAAAGGTCTGTTTGACACCTTATCTTTTT-TTCG-TTATTATCGA-TTTGAATTATTTAGAATCTATATCAGTTTTCATTTTCAAACTTAGAAAGTCTTCTTTTATTTATAAGATCCAAGAAATTCCCGGTCCAAAACTTTTTTAATTTACTACtttt------gagttTCTTTTCATTGACAGAGACCCAAGTCATATATTAAAATGATACTGATNCTTC--------------------------------------CGTAATGGTCGGCATAGCTTAATTGCGGAGGACTGAAAATCCTTGTGTCACCATT--------------------------------------------------------------------------------------------------------------------------------------------------------------------------------------------------------CGTAAAATAAGGATGATACTTCGGTAATGGCCGGGATAGCTCAGNNn

AY257707 AATTGGATTGAGCCTTGGTATGGAAACCTACTAAGTGATAACTTTCAAATTCAGAGAAACCCTGGAATTAAcaatgggcAATCCTGAGCCAAATCCTGGTTTACGTGAACAAACCGGAGTTTAGAAAGCGAGAAAAAA-GGGATAGGTGCAGAGACTCAATGGAAGCTGTTCTAACAAATGGAGTTCACTACCTTGTGTTGAGAAAGGAATCCTTCGATCAAAACTTCAAATCAAAAAG-GATGAAGGAGAAAAACCTATATTGTCTAAATATA------GGTAACACAAAA-CGATCTCAAAAATGACGACCTGAATCTCGATTTCTATTTTTTT--ATAAACAAAATCGAAATGATGTGAATCAATTCGAA-----GTTTAAGAAAtaatattcattGATCAAATGATTCTCTTCATAGTCTGATAGATCCGTGGTGGAActtattaaTCGGACGAGAATAAAGATAGAGTCCCATTTTAC-ATGTCAATACTGACAACAATGAAATTTATAGTAAGATGXXXGCTCTACT-CCCCAAAAAGGTCTGTTTGACACCTTACCTtttt-ttag-TTATTATCGA-TTTGAATTATTTAGAATCTATATCAGTTTTCATTTTCAAACTTAGAAAGTCTTCTTTTATTTATAAGATCCAAGAAATTCCCGGTCCAAAACTTTTTTAATTTACTACtttt------gagttTCTTTTCATTGACAGAGACCCAAGTCATATATTAAAATGATACTGATacttc--------------------------------------cgtaaTGGTCGGCATAGCTTAATTGCGGAGGACTGAAAATCCTTGTGTCACCATT--------------------------------------------------------------------------------------------------------------------------------------------------------------------------------------------------------CGTAAAATAAGGATGATACTTCGGTAATGGCCGGGATAGCTCAgttg

AY257708 AATTGGATTGAGCCTTGGTATGGAAACCTACTAAGTGATAACTTTCAAATTCAGAGAAACCCTGGAATTAAcaatgggcAATCCTGAGCCAAATCCTGGTTTACGTGAACAAACCGGAGTTTAGAAAGCGAGAAAAAA-GGGATAGGTGCAGAGACTCAATGGAAGCTGTTCTAACAAATGGAGTTCACTACCTTGTGTTGATAAAGGAATCCTTCGATCAAAACTTCAAATCAAAAAG-GATGAAGGAGAAAAACCTATATTGTCTAAATATA------GGTAACACAAAA-CGATCTCAAAAATGACGACCTGAATCTCGATTTCTATTTTTTT--ATAAACAAAATCGAAATGATGTGAATCAATTCGAA-----GTTTAAGAAAtaatattcattGATCAAATGATTCTCTTCATAGTCTGATAGATCCGTGGTGGAActtattaaTCGGACGAGAATAAAGATAGAGTCCCATTTTAC-ATGTCAATACTGACAACAATGAAATTTATAGTAAGATGXXXGCTCTACT-CCCCAAAAAGGTCTGTTTGACACCTTACCTtttt-ttag-TTATTATCGA-TTTGAATTATTTAGAATCTATATCAGTTTTCATTTTCAAACTTAGAAAGTCTTCTTTTATTTATAAGATCCAAGAAATTCCCGGTCCAAAACTTTTTTAATTTACTACtttt------gagttTATTTTCATTGACAGAGACCCAAGTCATATATTAAAATGATACTGATacttc--------------------------------------cgtaaTGGTCGGCATAGCTTAATTGCGGAGGACTGAAAATCCTTGTGTCACCATT--------------------------------------------------------------------------------------------------------------------------------------------------------------------------------------------------------CGTAAAATAAGGATGATACTTCGGTAATGGCCGGGATAGCTCAgttg

AY257709 AATTGGATTGAGCCTTGGTATGGTAACCTACTAAGTGATAACTTTCAAATTCAGAGAAACCCTGGAATTAAcaatgggcAATCCTGAGCCAAATCCTGGTTTACGTGAACAAACCGGAGTTTTGAAAGCGAGAAAAAA-GGGATAGGTGCAGAGACTCAATGGAAGCTGTTCTAACAAATGGAGTTCACTACCTTGTGTTGATAAAGGAATCCTTCGATCAAAACTTCAAATCAAAAAG-GATGAAGGAGAAAAACCTATATTGTCTAAATATA------GGTAACACAAAA-CGATCTCAAAAATGACGACCTGAATCTCGATTTCTATTTTTTT--ATAAACAAAATCGAAATGATGTGAATCAATTCGAA-----GTTTAAGAAAtaatattcattGATCAAATGATTCTCTTCATAGTCTGATAGATCCGTGGTGGAActtattaaTCGGACGAGAATAAAGATAGAGTCCCATTTTAC-ATGTCAATACTGACAACAATGAAATTTATAGTAAGATGXXXGCTCTACT-CCCCAAAAAGGTCTGTTTGACACCTTACCTtttt-ttag-TTATTATCGA-TTTGAATTATTTAGAATCTATATCAGTTTTCATTTTCAAACTTAGAAAGTCTTCTTTTATTTATAAGATCCAAGAAATTCCCGGTCCAAAACTTTTTTAATTTACTACtttt------gagttTCTTTTCATTGACAGAGACCCAAGTCATATATTAAAATGATACTGATacttc--------------------------------------cgtaaTGGTCGGCATAGCTTAATTGCGGAGGACTGAAAATCCTTGTGTCACCATT--------------------------------------------------------------------------------------------------------------------------------------------------------------------------------------------------------CGTAAAATAAGGATGATACTTCGGTAATGGCCGGGATAGCTCAgttg

AY257710 NNNNNNNNNNNNNNNNNNNNTGGNAACCTACTAAGTGATAACTTTCAAATTCAGAGAAACCCTGGAATTAAcaatgggcAATCCTGAGCCAAATCCTGGTTTACGTGAACAAACCGGAGTTTAGAAAGCGAGAAAAAA-GGGATAGGTGCAGAGACTCAATGGAAGCTGTTCTAACAAATGGAGTTCACTACCTTGTGTTGATAAAGGAATCCTTCGATCAAAACTTCAAATCAAAAAG-GATGAAGGAGAAAAACCTATATTGTCTAAATATA------GGTAACACAAAA-CGATCTCAAAAATGACGACCTGAATCTCGATTTCTATTTTTTT--ATAAACAAAATCGAAATGATGTGAATCAATTCGAA-----GTTTAAGAAAtaatattcattGATCAAATGATTCTCTTCATAGTCTGATAGATCCGTGGTGGAActtattaaTCGGACGAGAATAAAGATAGAGTCCCATTTTAC-ATGTCACTACTGACAACAATGAAATTTATAGTAAGATGXXXGCTCTACT-CCCCAAAAAGGTCTGTTTGACACCTTACCTtttt-ttag-TTATTATCGA-TTTGAATTATTTAGAATCTATATCAGTTTTCATTTTCAAACTTAGAAAGTCTTCTTTTATTTATAAGATCCAAGAAATTCCCGGTCCAAAACTTTTTTAATTTACTACtttt------gagttTCTTTTCATTGACAGAGACCCAAGTCATATATTAAAATGATACTGATacttc--------------------------------------cgtaaTGGTCGGCATAGCTTAATTGCGGAGGACTGAAAATCCTTGTGTCACCATT--------------------------------------------------------------------------------------------------------------------------------------------------------------------------------------------------------CGTAAAATAAGGATGATACTTCGGGAATGGCCGGGATAGCTCAgttg

AY257711 AATTGGATTGAGCCTTGGTATGGAAACCTACTAAGTGATAACTTTCAAATTCAGAGAAACCCTGGAATTAAcaatgggcAATCCTGAGCCAAATCCTGGTTTACGTGAACAAACCGGAGTTTAGAAAGCGAGAAAAAA-GGGATAGGTGCAGAGACTCAATGGAAGCTGTTCTAACAAATGGAGTTCACTACCTTGTGTTGATAAAGGAATCCTTCGATCAAAACTTCAAATCAAAAAG-GATGAAGGAGAAAAACCTATATTGTCTAAATATA------GGTAACACAAAA-CGATCTCAAAAATGACGACCTGAATCTCGATTTCTATTTTTTT--ATAAACAAAATCGAAATGATGTGAATCAATTCGAA-----GTTTAAGAAAtaatattcattGATCAAATGATTCTCTTCATAGTCTGATAGATCCGTGGTGGAActtattaaTCGGACGAGAATAAAGATAGAGTCCCATTTTAC-ATGTCACTACTGACAACAATGAAATTTATAGTAAGATGXXXGCTCTACT-CCCCAAAAAGGTCTGTTTGACATCTTACCTtttt-ttag-TTATTATCGA-TTTGAATTATTTAGAATCTATATCAGTTTTCATTTTCAAACTTAGAAAGTCTTCTTTTATTTATAAGATCCAAGAAATTCCCGGTCCAAAACTTTTTTAATTTACTACtttt------gagttTCTTTTCATTGACAGAGACCCAAGTCATATATTAAAATGATACTGATacttc--------------------------------------cgtaaTGGTCGGCATAGCTTAATTGCGGAGGACTGAAAATCCTTGTGTCACCATT--------------------------------------------------------------------------------------------------------------------------------------------------------------------------------------------------------CGTAAAATAAGGATGATACTTCGGGAATGGCCGGGATAGCTCAgttg

AY257712 AATTGGATTGAGCCTTGGTATGGAAACCTACTAAGTGATAACTTTCAAATTCAGAGAAACCCTGGAATTAAcaatgggcAATCCTGAGCCAAATCCTGGTTTACGTGAACAAACCGGAGTTTAGAAAGCGAGAAAAAA-GGGATAGGTGCAGAGACTCAATGGAAGCTGTTCTAACAAATGGAGTTCACTACCTTGTGTTGATAAAGGAATCCTTCGATCAAAACTTCAAATCAAAAAG-GATGAAGGAGAAAAACCTATATTGTCTAAATATA------GGTAACACAAAA-CGATCTCAAAAATGACGACCTGAATCTCGATTTCTATTTTTTT--ATAAACAAAATCGAAATGATGTGAATCAATTCGAA-----GTTTAAGAAAtaatattcattGATCAAATGATTCTCTTCATAGTCTAATAGATCCGTGGTGGAActtattaaTCGGACGAGAATAAAGATAGAGTCCCATTTTAC-ATGTCAATACTGACAACAATGAAATTTATAGTAAGATGXXXGCTCTACT-CCCCAAAAAGGTCTGTTTGACACCTTACCTtttt-ttag-TTATTATCGA-TTTGAATTATTTCGAATCTATATCAGTTTTCATTTTCAAACTTAGAAAGTCTTCTTTTATTTATAAGATCCAAGAAATTCCCGGTCCAAAACTTTTTTAATTTACTACtttt------gagttTCTTTTCATTGACAGAGACCCAAGTCATATATTAAAATGATACTGATacttc--------------------------------------cgtaaTGGTCGGCATAGCTTAATTGCGGAGGACTGAAAATCCTTGTGTCACCATT--------------------------------------------------------------------------------------------AGTAAAATGAGAA-TGATACTTCAGTAGATGATACCTCAGTAATGGTGGACATAGC--TTTTTTTTTTTG--CAGAGGACTGAAAATC------CTTGTGTCACCATTCGTAAAATGAGGATGATACTTCGGTAATGGCCGGGATAGCTCAgttg

AY257713 AATTGGATTGAGCCTTGGTATGGAAACCTACTAAGTGATAACTTTCAAATTCAGAGAAACCCTGGAATTAAcaatgggcAATCCTGAGCCAAATCCTGGTTTACGTGAACAAACCGGAGTTTAGAAAGCGAGAAAAAA-GGGATAGGTGCAGAGACTCAATGGAAGCTGTTCTAACAAATGGAGTTCACTACCTTGTGTTGATAAAGGAATCCTTCGATCAAAACTTCAAATCAAAAAG-GATGAAGGAGAAAAACCTATATTGTCTAAATATA------GGTAACACAAAA-CGATCTCAAAAATGACGACCTGAATCTCGATTTCTATTTTTTT--ATAAACAAAATCGAAATGATGTGAATCAATTCGAA-----GTTTAAGAAAtaatattcattGATCAAATGATTCTCTTCATAGTCTAATAGATCCGTGGTGGAActtattaaTCGGACGAGAATAAAGATAGAGTCCCATTTTAC-ATGTCAATACTGACAACAATGAAATTTATAGTAAGATGXXXNNNCTACT-CCCCAAAAAGGTCTGTTTGACACCTTACCTtttt-ttag-TTATTATCGA-TTTGAATTATTTCGAATCTATATCAGTTTTCATTTTCAAACTTAGAAAGTCTTCTTTTATTTATAAGATCCAAGAAATTCCCGGTCCAAAACTTTTTTAATTTACTACtttt------gagttTCTTTTCATTGACAGAGACCCAAGTCATATATTAAAATGAGACTGATacttc--------------------------------------cgtaaTGGTCGGCATAGCTTAATTGCGGAGGACTGAAAATCCTTGTGTCACCATT--------------------------------------------------------------------------------------------AGTAAAATGAGAA-TGATACTTCAGTAGATGATACCTCAGTAATGGTGGACATAGC--TTTTTTTTTTTG--CAGAGGACTGAAAATC------CTTGTGTCACCATTCGTAAAATGAGGATGATACTTCGGTAATGGCCGGGATAGCTCAGTTn

AY257714 AATTGGATTGAGCCTTGGTATGGAAACCTACTAAGTGATAACTTTCAAATTCAGAGAAACCCTGGAATTAAcaatgggcAATCCTGAGCCAAATCCTGGTTTACGTGAACAAACCGGAGTTTAGAAAGCGAGAAAAAA-GGGATAGGTGCAGAGACTCAATGGAAGCTGTTCTAACAAATGGAGTTCACTACCTTGTGTTGATAAAGGAATCCTTCGATCAAAACTTCAAATCAAAAAG-GATGAAGGAGAAAAACCTATATTGTCTAAATATA------GGTAACACAAAA-CGATCTCAAAAATGACGACCTGAATCTCGATTTCGATTTTTTT--ATAAACAAAATCGAAATGATGTGAATCAATTCGAA-----GTTTAAGAAAtaatattcattGATCAAATGATTCTCTTCATAGTCTAATAGATCCGTGGTGGAActtattaaTCGGACGAGAATAAAGATAGAGTCCCATTTTAC-ATGTCAATACTGACAACAATGAAATTTATAGTAAGATGXXXGCTCTACT-CCCCAAAAAGGTCTGTTTGACACCTTACCTtttt-ttag-TTATTATCGA-TTTGAATTATTTCGAATCTATATCAGTTTTCATTTTCAAACTTAGAAAGTCTTCTTTTATTTATAAGATCCAAGAAATTCCCGGTCCAAAACTTTTTTAATTTACTACtttt------gagttTCTTTTCATTGACAGAGACCCAAGTCATATATTAAAATGATACTGATacttc--------------------------------------cgtaaTGGTCGGCATAGCTTAATTGCGGAGGACTGAAAATCCTTGTGTCACCATT--------------------------------------------------------------------------------------------AGTAAAATGAGAA-TGATACTTCAGTAGATGATACCTCAGTAATGGTGGACATAGC--TTTTTTTTTTTG--CAGAGGACTGAAAATC------CTTGTGTCACCATTCGTAAAATGAGGATGATACTTCGGTAATGGCCGGGATAGCTCAGNNn

AY257715 AATTGGATTGAGCCTTGGTATGGAAACCTACTAAGTGATAACTTTCAAATTCAGAGAAACCCTGGAATTAAcaatgggcAATCCTGAGCCAAATCCTGGTTTACGTGAACAAACCGGAGTTTAGAAAGCGAGAAAAAA-GGGATAGGTGCAGAGACTCAATGGAAGCTGTTCTAACAAATGGAGTTCACTACCTTGTGTTGATAAAGGAATCCTTCGATCAAAACTTCAAATCAAAAAG-GATGAAGGAGAAAATCCTATATTGTCTAAATATA------GGTAACACAAAA-CGATCTCAAAAATGACGACCTGAATCTCGATTTCTATTTTTTT--ATAAACAAAATCGAAATGATGTGAATCAATTCGAA-----GTTTAAGAAAtaatattcattGATCAAATGATTCTCTTCATAGTCTAATAGATCCGTGGTGGAActtattaaTCGGACGAGAATAAAGATAGAGTCCCATTTTAC-ATGTCAATACTGACAACAATGAAATTTATAGTAAGATGXXXGCTCTACT-CCCCAAAAAGGTCTGTTTGACACCTTACCTtttt-ttag-TTATTATCGA-TTTGAATTATTTCGAATCTATATCAGTTTTCATTTTCAAACTTAGAAAGTCTTCTTTTATTTATAAGATCCAAGAAATTCCCGGTCCAAAACTTTTTTAATTTACTACtttt------gagttTCTTTTCATTGACAGAGACCCAAGTCATATATTAAAATGATACTGATacttc--------------------------------------cgtaaTGGTCGGCATAGCTTAATTGCGGAGGACTGAAAATCCTTGTGTCACCATT--------------------------------------------------------------------------------------------------------------------------------------------------------------------------------------------------------CGTAAAATGAGGATGATACTTCGGTAATGGCCGGGATAGCTCAgttg

AY257716 NNNNNNATTGAGCCTTGGTATGGAAACCTACTAAGTGATAACTTTCAAATTCAGAGAAACCCTGGAATTAAcaatgggcAATCCTGAGCCAAATCCTGGTTTACGTGAACAAACCGGAGTTTAGAAAGCGAGAAAAAA-GGGATAGGTGCAGAGACTCAATGGAAGCTGTTCTAACAAATGGAGTTCACTACCTTGTGTTGATAAAGGAATCCTTCGATCAAAACTTCAAATCAAAAAG-GATGAAGGAGAAAAACCTATATTGTCTAAATATA------GGTAACACAAAA-CGATCTCAAAAATGACGACCTGAATCTCGATTTCTATTTTTTT--ATAAACAAAATCGAAATGATGTGAATCAATTCGAA-----GTTTAAGAAAtaatattcattGATCAAATGATTCTCTTCATAGTCTAATAGATCCGTGGTGGAActtattaaTCGGACGAGAATAAAGATAGAGTCCCATTTTAC-ATGTCAATACTGACAACAATGAAATTTATAGTAAGATGXXXGCTCTACT-CCCCAAAAAGGTCTGTTTGACACCTTACCTtttt-ttag-TTATTATCGA-TTTGAATTATTTCGAATCTATATCAGTTTTCATTTTCAAACTTAGAAAGTCTTCTTTTATTTATAAGATCCAAGAAATTCCCGGTCCAAAACTTTTTTAATTTACTACtttt------gagttTCTTTTCATTGACAGAGACCCAAGTCATATATTAAAATGATACTGATacttc--------------------------------------cgtaaTGGTCGGCATAGCTTAATTGCGGAGGACTGAAAATCCTTGTGTCACCATT--------------------------------------------------------------------------------------------------------------------------------------------------------------------------------------------------------CGTAAAATGAGGATGATACTTCGGTAATGGCCGGGATAGCTCAgttg

DQ013045 AATTGGATTGAGCCTTGGTATGGAAACCTACTAAGTGATAACTTTCAAATTCAGAGAAACCCTGGAATTAAcaatgggcAATCCTGAGCCAAATCCTGGTTTACGTGAACAAACCGGAGTTTAGAAAGCGAGAAAAAA-GGGATAGGTGCAGAGACTCAATGGAAGCTGTTCTAACAAATGGAGTTCACTACCTTGTGTTGATAAAGGAATCCTTCGATCAAAACTTCAAATCAAAAAG-GATGAAGGAGAAAAACCTATATTGTCTAAATATA------GGTAACACAAAA-CGATCTCAAAAATGACGACCTGAATCTCGATTTCTATTTTTTT--ATAAACAAAATCGAAATGATGTGAATCAATTCGAA-----GTTTAAGAAAtaatattcattGATCAAATGATTCTCTTCATAGTCTGATAGATCCGTGGTGGAActtattaaTCGGACGAGAATAAAGATAGAGTCCCATTTTAC-ATGTCAATACTGACAACAATGAAATTTATAGTAAGATGXXXGCTCTACT-CCCCAAAAAGGTCTGTTTGACACCTTACCTtttt-ttag-TTATTATCGA-TTTGAATTATTTAGAATCTATATCAGTTTTCATTTTCAAACTTAGAAAGTCTTCTTTTATTTATAAGATCCAAGAAATTCCCGGTCCAAAACTTTTTTCATTTACTACtttt------gagttTCTTTTCATTGACAGAGACCCAAGTCATATATTAAAATGATACTGATacttc--------------------------------------cgtaaTGGTCGGCATAGCTTAATTGCGGAGGACTGAAAATCCTTGTGTCACCATT--------------------------------------------------------------------------------------------AGTAAAATGAGAA-TGATACTTCAGTAGATGATACCTCAGTAATGGTGGACATAGC--TTTTTTTTTTTG--CAGAGGACTGAAAATC------CTTGTGTCACCATTCGTAAAATGAGGATGATACTTCGGTAATGGCCGGGATAGCTCAgttg

DQ013046 AATTGGATTGAGCCTTGGTATGGAAACCTACTAAGTGATAACTTTCAAATTCAGAGAAACCCTGGAATTAAcaatgggcAATCCTGAGCCAAATCCTGGTTTACGTGAACAAACCAGAGTTTAGAAAGCGAGAAAAAA-GGGATAGGTGCAGAGACTCAATGGAAGCTGTTCTAACAAATGGAGTTCACTACCTTGTGTTGATAAAGGAATCCTTCGATCAAAACTTCAAATCAAAAAG-GATGAAGGAGAAAAACCTATATTGTCTAAATATA------GGTAACACAAAA-CGATCTCAAAAATGACGACCTGAATCTCGATTTCTATTTTTTT--ATAAACAAAATCGAAATGATGTGAATCAATTCGAA-----GTTTAAGAAAtaatattcattGATCAAATGATTCTCTTCATAGTCTGATAGATCCGTGGTGGAActtattaaTCGGACGAGAATAAAGATAGAGTCCCATTTTAC-ATGTCAATACTGACAACAATGAAATTTATAGTAAGATGXXXGCTCTACT-CCCCAAAAAGGTCTGTTTGACACCTTACCTtttt-ttag-TTATTATCGA-TTTGAATTATTTAGAATCTATATCAGTTTTCATTTTCAAACTTAGAAAGTCTTCTTTTATTTATAAGATCCAAGAAATTCCCGGTCCAAAACTTTTTTAATTTACTACtttt------gagttTCTTTTCATTGACAGAGACCCAAGTCATATAGTAAAATGATACTGATacttc--------------------------------------cgtaaTGGTCGGCATAGCTTAATTGCGGAGGACTGAAAATCCTTGTGTCACCATT--------------------------------------------------------------------------------------------------------------------------------------------------------------------------------------------------------CGTAAAATGAGGATGATACTTCGGTAATGGCCGGGATAGCTCAgttg

trnLF_DX AATTGGATTGAGCCTTGGTATGGAAACCTACTAAGTGATAACTTTCAAATTCAGAGAAACCCTGGAATTAAcaatgggcAATCCTGAGCCAAATCCTGGTTTACGTGAACAAACCGGAGTTTAGAAAGCGAGAAAAAA-GGGATAGGTGCAGAGACTCAATGGAAGCTGTTCTAACAAATGGAGTTCACTACCTTGTGTTGATAAAGGAATCCTTCGATCAAAACTTCAAATCAAAAAG-GATGAAGGAGAAAAACCTATATTGTCTAAATATA------GGTAACACAAAA-CGATCTCAAAAATGACGACCTGAATCTCGATTTCTATTTTTTT--ATAAACAAAATCGAAATGATGTGAATCAATTCGAA-----GTTTAAGAAAtaatattcattGATCAAATGATTCTCTTCATAGTCTGATAGATCCGTGGTGGAActtattaaTCGGACGAGAATAAAGATAGAGTCCCATTTTAC-ATGTCAATACTGACAACAATGAAATTTATAGTAAGATGXXXGCTCTACT-CCCCAAAAAGGTCTGTTTGACACCTTACCTtttt-ttag-TTATTATCGA-TTTGAATTATTTAGAATCTATATAAGTTTTCATTTTCAAACTTAGAAAGTCTTCTTTTATTTATAAGATCCAAGAAATTCCCGGTCCAAAACTTTTTTAATTTACTACtttt------gagttTCTTTTCATTGACAGAGACCCAAGTCATATATTAAAATGATACTGATacttc--------------------------------------cgtaaTGGTCGGCATAGCTTAATTGCGGAGGACTGAAAATCCTTGTGTCACCATT--------------------------------------------------------------------------------------------AGTAAAATGAGAA-TGATACTTCAGTAGATGATACCTCAGTAATGGTGGACATAGC---TTTTTTTTTTG--CAGAGGACTGAAAATC------CTTGTGTCACCATTCGTAAAATGAGGATGATACTTCGGTAATGGCCGGGATAGCTCAgttg

trnLF_EB AATTGGATTGAGCCTTGGTATGGAAACCTACTAAGTGATAACTTTCAAATTCAGAGAAACCCTGGAATTAAcaatgggcAATCCTGAGCCAAATCCTGGTTTACGTGAACAAACCGGAGTTTAGAAAGCGAGAAAAAA-GGGATAGGTGCAGAGACTCAATGGAAGCTGTTCTAACAAATGGAGTTCACTACCTTGTGTTGATAAAGGAATCCTTCGATCAAAACTTCAAATCAAAAAG-GATGAAGGAGAAAAACCTATATTGTCTAAATATA------GGTAACACAAAA-CGATCTCAAAAATGACGACCTGAATCTCGATTTCTATTTTTTT--ATAAACAAAATCGAAATGATGTGAATCAATTCGAA-----GTTTAAGAAAtaatattcattGATCAAATGATTCTCTTCATAGTCTGATAGATCCGTGGTGGAActtattaaTCGGACGAGAATAAAGATAGAGTCCCATTTTAC-ATGTCAATACTGACAACAATGAAATTTATAGTAAGATGXXXGCTCTACT-CCCCAAAAAGGTCTGTTTGACACCTTACCTTTT--ttAG-TTATTATCGA-TTTGAATTATTTAGAATCTATATCAGTTTTCATTTTCAAACTTAGAAAGTCTTCTTTGATTTATAAGATCCAAGAAATTCCCGGTCCAAAACTTTTTTAATTTACTACtttt------gagttTCTTTTCATTGACAGAGACCCAAGTCATATATTAAAATGATACTGATacttc--------------------------------------cgtaaTGGTCGGCATAGCTTAATTGCGGAGGACTGAAAATCCTTGTGTCACCATT--------------------------------------------------------------------------------------------AGTAAAATGAGAA-TGATACTTCAGTAGATGATACCTCAGTAATGGTGGACATAGC--TTTTTTTTTTTG--CAGAGGACTGAAAATC------CTTGTGTCAC--------AAATGAGGATGATACNNNNNNNNNNNNNNNNNNNNNNNNnnnn

trnLF_EC AATTGGATTGAGCCTTGGTATGGAAACCTACTAAGTGATAACTTTCAAATTCAGAGAAACCCTGGAATTAAcaatgggcAATCCTGAGCCAAATCCTGGTTTACGTGAACAAACCGGAGTTTAGAAAGCGAGAAAAAA-GGGATAGGTGCAGAGACTCAATGGAAGCTGTTCTAACAAATGGAGTTCACTACCTTGTGTTGATAAAGGAATCCTTCGATCAAAACTTCAAATCAAAAAG-GATGAAGGAGAAAAACCTATATTGTCTAAATATA------GGTAACACAAAA-CGATCTCAAAAATGACGACCTGAATCTCGATTTCTATTTTTTT--ATAAACAAAATCGAAATGATGTGAATCAATTCGAA-----GTTTAAGAAAtaatattcattGATCAAATGATTCTCTTCATAGTCTGATAGATCCGTGGTGGAActtattaaTCGGACGAGAATAAAGATAGAGTCCCATTTTAC-ATGTCAATACTGACAACAATGAAATTTATAGTAAGATGXXXGCTCTACT-CCCCAAAAAGGTCTGTTTGACACCTTACCTtttt-ttag-TTATTATCGA-TTTGAATTATTTAGAATCTATATCAGTTTTCATTTTCAAACTTAGAAAGTCTTCTTTGATTTATAAGATCCAAGAAATTCCCGGTCCAAAACTTTTTTAATTTACTACtttt------gagttTCTTTTCATTGACAGAGACCCAAGTCATATATTAAAATGATACTGATacttc--------------------------------------cgtaaTGGTCGGCATAGCTTAATTGCGGAGGACTGAAAATCCTTGTGTCACCATT--------------------------------------------------------------------------------------------AGTAAAATGAGAA-TGATACTTCAGTAGATGATACCTCAGTAATGGTGGACATAGC---TTTTTTTTTTG--CAGAGGACTGAAAATC------CTTGTGTCAC--------AAATGAGGATGATACTTCGGTAATGGCCGGGATAGCTCAgttg

trnLF_ED AATTGGATTGAGCCTTGGTATGGAAACCTACTAAGTGATAACTTTCAAATTCAGAGAAACCCTGGAATTAAcaatgggcAATCCTGAGCCAAATCCTGGTTTACGTGAACAAACCGGAGTTTAGAAAGCGAGAAAAAA-GGGATAGGTGCAGAGACTCAATGGAAGCTGTTCTAACAAATGGAGTTCACTACCTTGTGTTGATAAAGGAATCCTTCGATCAAAACTTCAAATCAAAAAG-GATGAAGGAGAAAAACCTATATTGTCTAAATATA------GGTAACACAAAA-CGATCTCAAAAATGACGACCTGAATCTCGATTTCTATTTTTTT--ATAAACAAAATCGAAATGATGTGAATCAATTCGAA-----GTTTAAGAAAtaatattcattGATCAAATGATTCTCTTCATAGTCTGATAGATCCGTGGTGGAActtattaaTCGGACGAGAATAAAGATAGAGTCCCATTTTAC-ATGTCAATACTGACAACAATGAAATTTATAGTAAGATGXXXGCTCTACT-CCCCAAAAAGGTCTGTTTGACACCTTACCTtttt-ttag-TTATTATCGA-TTTGAATTATTTAGAATCTATATCAGTTTTCATTTTCAAACTTAGAAAGTCTTCTTTGATTTATAAGATCCAAGAAATTCCCGGTCCAAAACTTTTTTAATTTACTACtttt------gagttTCTTTTCATTGACAGAGACCCAAGTCATATATTAAAATGATACTGATacttc--------------------------------------cgtaaTGGTCGGCATAGCTTAATTGCGGAGGACTGAAAATCCTTGTGTCACCATT--------------------------------------------------------------------------------------------AGTAAAATGAGAA-TGATACTTCAGTAGATGATACCTCAGTAATGGTGGACATAGC---TTTTTTTTTTG--CAGAGGACTGAAAATC------CTTGTGTCAC-------AAAATGAGGATGATACTTCGGTAATGGCCGGGATAGCTCAgttg

trnLF_EE AATTGGATTGAGCCTTGGTATGGAAACCTACTAAGTGATAACTTTCAAATTCAGAGAAACCCTGGAATTAAcaatgggcAATCCTGAGCCAAATCCTGGTTTACGTGAACAAACCGGAGTTTAGAAAGCGAGAAAAAA-GGGATAGGTGCAGAGACTCAATGGAAGCTGTTCTAACAAATGGAGTTCACTACCTTGTGTTGATAAAGGAATCCTTCGATCAAAACTTCAAATCAAAAAG-GATGAAGGAGAAAAACCTATATTGTCTAAATATA------GGTAACACAAAA-CGATCTCAAAAATGACGACCTGAATCTCGATTTCGATTTTTTT--ATAAACAAAATCGAAATGATGTGAATCAATTCGAA-----GTTTAAGAAAtaatattcattGATCAAATGATTCTCTTCATAGTCTGATAGATCCGTGGTGGAActtattaaTCGGACGAGAATAAAGATAGAGTCCCATTTTAC-ATGTCAATACTGACAACAATGAAATTTATAGTAAGATGXXXGCTCTACT-CCCCAAAAAGGTCTGTTTGACACCTTACCTtttt-ttag-TTATTATCGA-TTTGAATTATTTAGAATCTATATCAGTTTTCATTTTCAAACTTAGAAAGTCTTCTTTTATTTATAAGATCCAAGAAATTCCCGGTCCAAAACTTTTTTAATTTACTACtttt------gagttTCTTTTCATTGACAGAGACCCAAGTCATATATTAAAATGATACTGATacttc--------------------------------------cgtaaTGGTCGGCATAGCTTAATTGCGGAGGACTGAAAATCCTTGTGTCACCATT--------------------------------------------------------------------------------------------------------------------------------------------------------------------------------------------------------CGTAAAATAAGGATGATACTTCGGTAATGGCCGGGATAGCTCAgttg

trnLF_EL NNNNNNNNNNNNNNNNNNNNNNNNNNNNTACTAAGTGATAACTTTCAAATTCAGAGAAACCCTGGAATTAAcaatgggcAATCCTGAGCCAAATCCTGGTTTACGTGAACAAACCGGAGTTTAGAAAGCGAGAAAAAA-GGGATAGGTGCAGAGACTCAATGGAAGCTGTTCTAACAAATGGAGTTCACTACCTTGTGTTGATAAAGGAATCCTTCGATCAAAACTTCAAATCAAAAAG-GATGAAGGAGAAAAACCTATATTGTCTAAATATA------GGTAACACAAAA-CGATCTCAAAAATGACGACCTGAATCTCGATTTCTATTTTTTT--ATAAACAAAATCGAAATGATGTGAATCAATTCGAA-----GTTTAAGAAAtaatattcattGATCAAATGATTCTCTTCATAGTCTGATAGATCCGTGGTGGAActtattaaTCGGACGAGAATAAAGATAGAGTCCCATTTTAC-ATGTCAATACTGACAACAATGAAATTTATAGTAAGATGXXXNNNNNNNN-NNNNNNNNNNNNNNNNNNNNNCCCTTACCTTTT--tTAG-TTATTAGCGA-TTTGAATTATTTAGAATCTATATCAGTTTTCATTTTCAAACTTAGAAAGTCTTCTTTTATTTATAAGATCCAAGAAATTCCCGGTCCAAAACTTTTTTAATTTACTACtttt------gagttTCTTTTCATTGACAGAGACCCAAGTCATATATTAAAATGATACTGATacttc--------------------------------------cgtaaTGGTCGGCATAGCTTAATTGCGGAGGACTGAAAATCCTTGTGTCACCATT--------------------------------------------------------------------------------------------AGTAAAATGAGAA-TGATACTTCAGTAGATGATACCTCAGTAATGGTGGACATAGC--TTTTTTTTTTTG--CAGAGGACTGAAAATC------CTTGTGTCACCATTCGTAAAATGAGGATGATACTNNNNNNNNNNNNNNNNNNNNNNNnnnn

trnLF_EM AATTGGATTGAGCCTTGGTATGGAAACCTACTAAGTGATAACTTTCAAATTCAGAGAAACCCTGGAATTAAcaatgggcAATCCTGAGCCAAATCCTGGTTTACGTGAACAAACCGGAGTTTAGAAAGCGAGAAAAAA-GGGATAGGTGCAGAGACTCAATGGAAGCTGTTCTAACAAATGGAGTTCACTACCTTGTGTTGATAAAGGAATCCTTCGATCAAAACTTCAAATCAAAAAG-GATGAAGGAGAAAAACCTATATTGTCTAAATATA------GGTAACACAAAA-CGATCTCAAAAATGACGACCTGAATCTCGATTTCTATTTTTTT--ATAAACAAAATCGAAATGATGTGAATCAATTCGAA-----GTTTAAGAAAtaatattcattGATCAAATGATTCTCTTCATAGTCTGATAGATCCGTGGTGGAActtattaaTCGGACGAGAATAAAGATAGAGTCCCATTTTAC-ATGTCAATACTGACAACAATGAAATTTATAGTAAGATGXXXGCTCTACT-CCCCAAAAAGGTCTGTTTGACACCTTACCTtttt-ttag-TTATTAGCGA-TTTGAATTATTTAGAATCTATATCAGTTTTCATTTTCAAACTTAGAAAGTCTTCTTTTATTTATAAAATCCAAGAAATTCCCGGTCCAAAACTTTTTTAATTTACTACtttt------gagttTCTTTTCATTGACAGAGACCCAAGTCATATATTAAAATGATACTGATacttc--------------------------------------cgtaaTGGTCGGCATAGCTTAATTGCGGAGGACTGAAAATCCTTGTGTCACCATT--------------------------------------------------------------------------------------------------------------------------------------------------------------------------------------------------------CGTAAAATGAGGATGATACTTCGGTAATGGCCGGGATAGCTCAgttg

trnLF_EN AATTGGATTGAGCCTTGGTATGGAAACCTACTAAGTGATAACTTTCAAATTCAGAGAAACCCTGGAATTAAcaatgggcAATCCTGAGCCAAATCCTGGTTTACGTGAACAAACCGGAGTTTAGAAAGCGAGAAAAAA-GGGATAGGTGCAGAGACTCAATGGAAGCTGTTCTAACAAATGGAGTTCACTACCTTGTGTTGATAAAGGAATCCTTCGATCAAAACTTCAAATCAAAAAG-GATGAAGGAGAAAAACCTATATTGTCTAAATATA------GGTAACACAAAA-CGATCTCAAAAATGACGACCTGAATCTCGATTTCTATTTTTTT--ATAAACAAAATCGAAATGATGTGAATCAATTCGAA-----GTTTAAGAAAtaatattcattGATCAAATGATTCTCTTCATAGTCTGATAGATCCGTGGTGGAActtattaaTCGGACGAGAATAAAGATAGAGTCCCATTTTAC-ATGTCAATACTGACAACAATGAAATTTATAGTAAGATGXXXGCTCTACT-CCCCAAAAAGGTCTGTTTGACACCTTACCTtttt-ttag-TTATTAGCGA-TTTGAATTATTTAGAATCTATATCAGTTTTCATTTTCAAACTTAGAAAGTCTTCTTTTATTTATAAGATCCAAGAAATTCCCGGTCCAAAACTTTTTTAATTTACTACtttt------gagttTCTTTTCATTGACAGAGACCCAAGTCATATATTAAAATGATACTGATacttc--------------------------------------cgtaaTGGTCGGCATAGCTTAATTGCGGAGGACTGAAAATCCTTGTGTCACCATT--------------------------------------------------------------------------------------------------------------------------------------------------------------------------------------------------------CGTAAAATGAGGATGATACTTCGGTAATGGCCGGGATAGCTCAgttg

trnLF_EP AATTGGATTGAGCCTTGGTATGGAAACCTACTAAGTGATAACTTTCAAATTCAGAGAAACCCTGGAATTAAcaatgggcAATCCTGAGCCAAATCCTGGTTTACGTGAACAAACCGGAGTTTAGAAAGCGAGAAAAAA-GGGATAGGTGCAGAGACTCAATGGAAGCTGTTCTAACAAATGGAGTTCACTACCTTGTGTTGATAAAGGAATCCTTCGATCAAAACTTCAAATCAAAAAG-GATGAAGGAGAAAAACCTATATTGTCTAAATATA------GGTAACACAAAA-CGATCTCAAAAATGACGACCTGAATCTCGATTTCTATTTTTTT--ATAAACAAAATCGAAATGATGTGAATCAATTCGAA-----GTTTAAGAAAtaatattcattGATCAAATGATTCTCTTCATAGTCTGATAGATCCGTGGTGGAActtattaaTCGGACGAGAATAAAGATAGAGTCCCATTTTAC-ATGTCAATACTGACAACAATGAAATTTATAGTAAGATGXXXGCTCTACT-CCCCAAAAAGGTCTGTTTGACACCTTACCTtttt-ttag-TTATTATCGA-TTTGAATTATTTAGAATCTATATCAGTTTTCATTTTCAAACTTAGAAAGTCTTCTTTTATTTATAAGATCCAAGAAATTCCCGGTCCAAAACTTTTTTAATTTACTACtttt------gagttTCTTTTCATTGACAGAGACCCAAGTCATATATTAAAATGATACTGATacttc--------------------------------------cgtaaTGGTCGGCATAGCTTAATTGCGGAGGACTGAAAATCCTTGTGTCACCATT--------------------------------------------------------------------------------------------AGTAAAATGAGAA-TGATACTTCAGTAGATGATACCTCAGTAATGGTGGACATAGC---TTTTTTTTTTG--CAGAGGACTGAAAATC------CTTGTGTCACCATTCGTAAAATGAGGATGATACTTCGGTAATGGCCGGGATAGCTCAgttg

trnLF_FA AATTGGATTGAGCCTTGGTATGGAAACCTACTAAGTGATAACTTTCAAATTCAGAGAAACCCTGGAATTAAcaatgggcAATCCTGAGCCAAATCCTGGTTTACGTGAACAAACCGGAGTTTAGAAAGCGAGAAAAAA-GGGATAGGTGCAGAGACTCAATGGAAGCTGTTCTAACAAATGGAGTTCACTACCTTGTGTTGATAAAGGAATCCTTCGATCAAAACTTCAAATCAAAAAG-GATGAAGGAGAAAAACCTATATTGTCTAAATATA------GGTAACACAAAA-CGATCTCAAAAATGACGACCTGAATCTCGATTTCTATTTTTTTTTATAAACAAAATCGAAATGATGTGAATCAATTCGAA-----GTTTAAGAAAtaatattcattGATCAAATGATTCTCTTCATAGTCTGATAGATCCGTGGTGGAActtattaaTCGGACGAGAATAAAGATAGAGTCCCATTTTAC-ATGTCAATACTGACAACAATGAAATTTATAGTAAGATGXXXGCTCTACT-CCCCAAAAAGGTCTGTTTGACACCTTACCTtttt-ttag-TTATTATCGA-TTTGAATTATTTAGAATCTATATCAGTTTTCATTTTCAAACTTAGAAAGTCTTCTTTTATTTATAAGATCCAAGAAATTCCCGGTCCAAAACTTTTTTAATTTACTACtttt------gagttTCTTTTCATTGACAGAGACCCAAGTCATATATTAAAATGATACTGATACTTC--------------------------------------------------------------------------------------------------------------------------------------------------------------------------------------------------------------------------------------------------------------------------------------------------------------------------CG-TAATGGTNNNNNNNNNNNNnnnn

trnLF_FD NNNNNNNNNNNNNNNNNNNNNNNNNNCCTACTAAGTGATAACTTTCAAATTCAGAGAAACCCTGGAATTAAcaatgggcAATCCTGAGCCAAATCCTGGTTTACGTGAACAAACCGGAGTTTAGAAAGCGAGAAAAAA-GGGATAGGTGCAGAGACTCAATGGAAGCTGTTCTAACAAATGGAGTTCACTACCTTGTGTTGATAAAGGAATCCTTCGATCAAAACTTCAAATCAAAAAG-GATGAAGGAGAAAAACCTATATTGTCTAAATATA------GGTAACACAAAA-CGATCTCAAAAATGACGACCTGAATCTCGATTTCTATTTTTTT--ATAAACAAAATCGAAATGATGTGAATCAATTCGAA-----GTTTAAGAAAtaatattcattGATCAAATGATTCTCTTCATAGTCTGATAGATCCGTGGTGGAActtattaaTCGGACGAGAATAAAGATAGAGTCCCATTTTAC-ATGTCAATACTGACAACAATGAAATTTATAGTAAGATGXXXGCTCTACT-CCCCAAAAAGGTCTGTTTGACACCTTACCTtttt-ttag-TTATTATCGA-TTTGAATTATTTAGAATCTATATCAGTTTTCATTTTCAAACTGAGAAAGTCTTCTTTTATTTATAAGATCCAAGAAATTCCCGGTCCAAAACTTTTTTAATTTACTACtttt------gagttTCTTTTCATTGACAGAGACCCAAGTCATATATTAAAATGATACTGATacttc--------------------------------------cgtaaTGGTCGGCATAGCTTAATTGCGGAGGACTGAAAATCCTTGTGTCACCATT--------------------------------------------------------------------------------------------AGTAAAATGAGAA-TGATACTTCAGTAGATGATACCTCAGTAATGGTGGACATAGC----TTTTTTTTTGTGCAGAGGACTGAAAATC------CTTGTGTCACCATTCGTAAAATAAGGATGANNNNNNNNNNNNNNNNNNNNNNNNNNNnnnn

trnLF_FF AATTGGATTGAGCCTTGGTATGGAAACCTACTAAGTGATAACTTTCAAATTCAGAGAAACCCTGGAATTAAcaatgggcAATCCTGAGCCAAATCCTGGTTTACGTGAACAAACCGGAGTTTAGAAAGCGAGAAAAAA-GGGATAGGTGCAGAGACTCAATGGAAGCTGTTCTAACAAATGGAGTTCACTACCTTGTGTTGATAAAGGAATCCTTCGATCAAAACTTCAAATCAAAAAG-GATGAAGGAGAAAAACCTATATTGTCTAAATATA------GGTAACACAAAA-CGATCTCAAAAATGACGACCTGAATCTCGATTTCTATTTTTTT--ATAAACAAAATCGAAATGATGTGAATCAATTCGAA-----GTTTAAGAAAtaatattcattGATCAAATGATTCTCTTCATAGTCTGATAGATCCGTGGTGGAActtattaaTCGGACGAGAATAAAGATAGAGTCCCATTTTAC-ATGTCAATACTGACAACAATGAAATTTATAGTAAGATGXXXGCTCTACT-CCCCAAAAAGGTCTGTTTGACACCTTACCTtttt-ttag-TTATTATCGA-TTTGAATTATTTAGAATCTATATCAGTTTTCATTTTCAAACTTAGAAAGTCTTCTTTTATTTATAAGATCCAAGAAATTCCCGGTCCAAAACTTTTTTAATTTACTACtttt------gagttTCTTTTCATTGACAGAGACCCAAGTCATATATTAAAATGATACTGATacttc--------------------------------------cgtaaTGGTCGGCATAGCTTAATTGCGGAGGACTGAAAATCCTTGTGTCACCATT--------------------------------------------------------------------------------------------------------------------------------------------------------------------------------------------------------CGTAAAATGAGGATGATACTTCGGTAATGGCCGGGATAGCTCAgttg

trnLF_FI AATTGGATTGAGCCTTGGTATGGAAACCTACTAAGTGATAACTTTCAAATTCAGAGAAACCCTGGAATTAACAATGGGCAATCCTGAGCCAAATCCTGGTTTACGTGAACAAACCGGAGTTTAGAAAGCGAGAAAAAA-GGGATAGGTGCAGAGACTCAATGGAAGCTGTTCTAACAAATGGAGTTCACTACCTTGTGTTGATAAAGGAATCCTTCGATCAAAACTTCAAATCAAAAAG-GATGAAGGAGAAAAACCTATATTGTCTAAATATA------GGTAACACAAAA-CGATCTCAAAAATGACGACCTGAATCTCGATTTCTATTTTTTT--ATAAACAAAATCGAAATGATGTGAATCAATTCGAA-----GTTTAAGAAATAATATTCATTGATCAAATGATTCTCTTCATAGTCTGATAGATCCGTGGTGGAACTTATTAATCGTACGAGAATAAAGATAGAGTCCCATTTTAC-ATGTCAATACTGACAACAATGAAATTTATAGTAAGATGXXXGCTCTACT-CCCCAAAAAGGTCTGTTTGACACCTTACCTTTTT-TTAG-TTATTATCGA-TTTGAATTATTTAGAATCTATATCAGTTTTCATTTTCAAACTTAGAAAGTCTTCTTTTATTTATAAGATCCAAGAAATTCCCGGTCCAAAACTTTTTTAATTTACTACTTTT------GAGTTTCTTTTCATTGACAGAGACCCAAGTCATATATTAAAATGATACTGATACTTC--------------------------------------CGTAATGGTCGGCATAGCTTAATTGCGGAGGACTGAAAATCCTTGTGTCACCATT--------------------------------------------------------------------------------------------------------------------------------------------------------------------------------------------------------CGTAAAATAAGGATGATACTTCGGTAATGGCCGGGATAGCTCAgttg

trnLF_FK AATTGGATTGAGCCTTGGTATGGAAACCTACTAAGTGATAACTTTCAAATTCAGAGAAACCCTGGAATTAACAATGGGCAATCCTGAGCCAAATCCTGGTTTACGTGAACAAACCGGAGTTTAGAAAGCGAGAAAAAA-GGGATAGGTGCAGAGACTCAATGGAAGCTGTTCTAACAAATGGAGTTCACTACCTTGTGTTGATAAAGGAATCCTTCGATCAAAACTTCAAATCAAAAAG-GATGAAGGAGAAAAACCTATATTGTCTAAATATA------GGTAACACAAAA-CGATCTCAAAAATGACGACCTGAATCTCGATTTCTATTTTTTT--ATAAACAAAATCGAAATGATGTGAATCAATTCGAA-----GTTTAAGAAATAATATTCATTGATCAAATGATTCTCTTCATAGTCTAATAGATCCGCGGTGGAACTTATTAATCGGACGAGAATAAAGATAGAGTCCCATTTTAC-ATGTCAATACTGACAACAATGAAATTTATAGTAAGATGXXXGCTCTACT-CCCCAAAAAGGTCTGTTTGACACCTTACCTTTTT-TTAG-TTATTATCGA-TTTGAATTATTTCGAATCTATATCAGTTTTCATTTTCAAACTTAGAAAGTCTTCTTTTATTTATAAGATCCAAGAAATTCCCGGTCCAAAACTTTTTTAATTTACTACTTTT------GAGTTTCTTTTCATTGACAGAGACCCAAGTCATATATTAAAATGATACTGATACTTC--------------------------------------CGTAATGGTCGGCATAGCTTAATTGCGGAGGACTGAAAATCCTTGTGTCACCATT--------------------------------------------------------------------------------------------AGTAAAATGAGAA-TGATACTTCAGTAGATGATACCTCAGTAATGGTGGACATAGC--TTTTTTTTTTTG--CAGAGGACTGAAAATC------CTTGTGTCACCATTCGTAAAATGAGGATGANNNNNNNNNNNNNNNNNNNNNNNNNNNnnnn

trnLF_FN AATTGGATTGAGCCTTGGTATGGAAACCTACTAAGTGATAACTTTCAAATTCAGAGAAACCCTGGAATTAACAATGGGCAATCCTGAGCCAAATCCTGGTTTACGTGAACAAACCGGAGTTTAGAAAGCGAGAAAAAA-GGGATAGGTGCAGAGACTCAATGGAAGCTGTTCTAACAAATGGAGTTCACTACCTTGTGTTGATAAAGGAATCCTTCGATCAAAACTTCAAATCAAAAAG-GATGAAGGAGAAAAACCTATATTGTCTAAATATA------GGTAACACAAAA-CGATCTCAAAAATGACGACCTGAATCTCGATTTCTATTTTTTT--ATAAACAAAATCGAAATGATGTGAATCAATTCGAA-----GTTTAAGAAATAATATTCATTGATCAAATGATTCTCTTCATAGTCTGATAGATCCGTGGTGGAACTTATTAATCGGACGAGAATAAAGATAGAGTCCCATTTTAC-ATGTCAATACTGACAACAATGAAATTTATAGTAAGATGXXXGCTCTACT-CCCCAAAAAGGTCTGTTTGACACCTTACCTTTTT-TTAG-TTATTATCGA-TTTGAATTATTTAGAATCTATATAAGTTTTCATTTTCAAACTTAGAAAGTCTTCTTTTATTTATAAGATCCAAGAAATTCCCGGTCCAAAACTTTTTTAATTTACTACTTTT------GAGTTTCTTTTCATTGACAGAGACCCAAGTCATATATTAAAATGATACTGATACTTC--------------------------------------CGTAATGGTCGGCATAGCTTAATTGCGGAGGACTGAAAATCCTTGTGTCACCATT--------------------------------------------------------------------------------------------------------------------------------------------------------------------------------------------------------CGTAAAATGAGGATGATACTTCGGTAATGGCCGGGATAGCTCAgttg

trnLF_FO AATTGGATTGAGCCTTGGTATGGAAACCTACTAAGTGATAACTTTCAAATTCAGAGAAACCCTGGAATTAACAATGGGCAATCCTGAGCCAAATCCTGGTTTACGTGAACAAACCGGAGTTTAGAAAGCGAGAAAAAA-GGGATAGGTGCAGAGACTCAATGGAAGCTGTTCTAACAAATGGAGTTCACTACCTTGTGTTGATAAAGGAATCCTTCGATCAAAACTTCAAATCAAAAAG-GATGAAGGAGAAAAACCTATATTGTCTAAATATA------GGTAACACAAAA-CGATCTCAAAAATGACGACCTGAATCTCGATTTCTATTTTTTT--ATAAACAAAATCGAAATGATGTGAATCAATTCGAA-----GTTTAAGAAATAATATTCATTGATCAAATGATTCTCTTCATAGTCTGATAGATCCGTGGTGGAACTTATTAATCGGACGAGAATAAAGATAGAGTCCCATTTTAC-ATGTCAATACTGACAACAATGAAATTTATAGTAAGATGXXXGCTCTACT-CCCCAAAAAGGTCTGTTTGACNCCTTACCTTTTT-TTAG-TTATTATCGA-TTTGAATTATTTAGAATCTATATAAGTTTTCATTTTCAAACTTAGAAAGTCTTCTTTTATTTATAAGATCCAAGAAATTCCCGGTCCAAAACTTTTTTAATTTACTACTTTT------GAGTTTCTTTTCATTGACAGAGACCCAAGTCATATATTAAAATGATACTGATACTTC--------------------------------------CGTAATGGTCGGCATAGCTTAATTGCGGAGGACTGAAAATCCTTGTGTCACCATT--------------------------------------------------------------------------------------------AGTAAAATGAGAA-TGATACTTCAGTAGATGATACCTCAGTAATGGTGGACATAGC--TTTTTTTTTTTG--CAGAGGACTGAAAATC------CTTGTGTCACCATTCGTAAAATGAGGATGATACTNNNNNNNNNNNNNNNNNNNNNNNnnnn

trnLF_FZ AATTGGATTGAGCCTTGGTATGGAAACCTACTAAGTGATAACTTTCAAATTCAGAGAAACCCTGGAATTAACAATGGGCAATCCTGAGCCAAATCCTGGTTTACGTGAACAAACCGGAGTTTAGAAAGCGAGAAAAAA-GGGATAGGTGCAGAGACTCAATGGAAGCTGTTCTAACAAATGGAGTTCACTACCTTGTGTTGATAAAGGAATCCTTCGATCAAAACTTCAAATCAAAAAG-GATGAAGGAGAAAAACCTATATTGTCTAAATATA------GGTAACACAAAA-CGATCTCAAAAATGACGACCTGAATCTCGATTTCTATTTTTTT--ATAAACAAAATCGAAATGATGTGAATCAATTCGAA-----GTTTAAGAAATAATATTCATTGATCAAATGATTCTCTTCATAGTCTGATAGATCCGTGGTGGAACTTATTAATCGGACGAGAATAAAGATAGAGTCCCATTTTAC-ATGTCAATACTGACAACAATGAAATTTATAGTAAGATGXXXGCTCTACT-CCCCAAAAAGGTCTGTTTGACACCTTACCTTTTT-TTAG-TTATTATCGA-TTTGAATTATTTAGAATCTATATCAGTTTTCATTTTCAAACTTAGAAAGTCTTCTTTTATTTATAAGATCCAAGAAATTCCCGGTCCAAAACTTTTTTAATTTACTACTTTT------GAGTTTCTTTTCATTGACAGAGACCCAAGTCATATATTAAAATGATACTGATACTTC--------------------------------------CGTAATGGTCGGCATAGCTTAATTGCGGAGGACTGAAAATCCTTGTGTCACCATT--------------------------------------------------------------------------------------------AGTAAAATGAGAA-TGATACTTCAGTAGATGATACCTCAGTAATGGTGGACATAGC----TTTTTTTTTGTGCAGAGGACTGAAAATC------CTTGTGTCACCATTCGTAAAATAAGGATGATACTTCGGTAATGGCCGGGATAGCTCAgttg

trnLF_GB AATTGGATTGAGCCTTGGTATGGAAACCTACTAAGTGATAACTTTCAAATTCAGAGAAACCCTGGAATTAACAATGGGCAATCCTGAGCCAAATCCTGGGTTACGCGAACAAAACAGAGTTTAGAAAGC----------GGGATAGGTGCAGAGACTCAATGGAAGCTGTTCTAACAAATGGAGTTCAATCCCTTGTGTTGAATCAAACGA------------------------------------------------------------------------------------------------------------------------------------------------------------------------------------------------------TTCACTTCATAGTCTGATAGATCCTTGGTGGAACTTATTAATCGGACGAGAATAAAGATAGAGTCCCATTCTAC-ATGTCAATACTGACAACAATGAAATTTATAGTAAGATGXXXGCTCTACT-CCCCAAAAAGGTCTGTTTGACACCTTATCTTTTT-TTCG-TTATTATCGA-TTTGAATTATTTAGAATCTATATCAGTTTTCATTTTCAAACTTAGAAAGTCTTCTTTTATTTATAAGATCCAAGAAATTCCCGGTCCAAAACTTTTTTAATTTACTACTTTT------GAGTTTCTTTTCATTGACAGAGACCCAAGTCATATATTAAAATGATACTGATACTTC--------------------------------------CGTAATGGTCGGCATAGCTTAATTGCGGAGGACTGAAAATCCTTGTGTCACCATT--------------------------------------------------------------------------------------------------------------------------------------------------------------------------------------------------------CGTAAAATAAGGATGATACTTCGGTAATGGCCGGGATAGCTCAgttg

trnLF_GC AATTGGATTGAGCCTTGGTATGGAAACCTACTAAGTGATAACTTTCAAATTCAGAGAAACCCTGGAATTAACAATGGGCAATCCTGAGCCAAATCCTGGTTTACGTGAACAAACCGGAGTTTAGAAAGCGAGAAAAAA-GGGATAGGTGCAGAGACTCAATGGAAGCTGTTCTAACAAATGGAGTTCACTACCTTGTGTTGATAAAGGAATCCTTCGATCAAAACTTCAAATCAAAAAG-GATGAAGGAGAAAAACCTATATTGTCTAAATATA------GGTAACACAAAA-CGATCTCAAAAATGACGACCTGAATCTCGATTTCTATTTTTTT--ATAAACAAAATCGAAATGATGTGAATCAATTCGAA-----GTTTAAGAAATAATATTCATTGATCAAATGATTCTCTTCATAGTCTGATAGATCCGTGGTGGAACTTATTAATCGGACGAGAATAAAGATAGAGTCCCATTTTAC-ATGTCAATACTGACAACAATGAAATTTATAGTAAGATGXXXGCTCTACT-CCCCAAAAAGGTCTGTTTGACACCTTACCTTTTT-TTAG-TTATTATCGA-TTTGAATTATTTAGAATCTATATCAGTTTTCATTTTCAAACTTAGAAAGTCTTCTTTTATTTATAAGATCCAAGAAATTCCCGGTCCAAAACTTTTTTCATTTACTACTTTT------GAGTTTCTTTTCATTGACAGAGACCCAAGTCATATATTAAAATGATACTGATACTTC--------------------------------------CGTAATGGTCGGCATAGCTTAATTGCGGAGGACTGAAAATCCTTGTGTCACCATT--------------------------------------------------------------------------------------------AGGAAAATGAGAA-TGATACTTCAGTAGATGATACCTCAGTAATGGTGGACATAGC----TTTTTTTTTGTGCAGAGGACTGAAAATC------CTTGTGTCACCATTCGTAAAATAAGGATGATACTNNNNNNNNNNNNNNNNNNNNNNNnnnn

trnLF_HE AATTGGATTGAGCCTTGGTATGGAAACCTACTAAGTGATAACTTTCAAATTCAGAGAAACCCTGGAATTAAcaatgggcAATCCTGAGCCAAATCCTGGTTTACGTGAACAAACCGGAGTTTAGAAAGCGAGAAAAAA-GGGATAGGTGCAGAGACTCAATGGAAGCTGTTCTAACAAATGGAGTTCACTACCTTGTGTTGATAAAGGAATCCTTCGATCAAAACTTCAAATCAAAAAG-GATGAAGGAGAAAAACCTATATTGTCTAAATATA------GGTAACACAAAA-CGATCTCAAAAATGACGACCTGAATCTCGATTTCTATTTTTTTTTATAAACAAAATCGAAATGATGTGAATCAATTCGAA-----GTTTAAGAAAtaatattcattGATCAAATGATTCTCTTCATAGTCTGATAGATCCGTGGTGGAActtattaaTCGGACGAGAATAAAGATAGAGTCCCATTTTAC-ATGTCAATACTGACAACAATGAAATTTATAGTAAGATGXXXGCTCTACT-CCCCAAAAAGGTCTGTTTGACACCTTACCTTTTT-TTAG-TTATTATCGA-TTTGAATTATTTAGAATCTATATCAGTTTTCATTTTCAAACTTAGAAAGTCTTCTTTTATTTATAAGATCCAAGAAATTCCCGGTCCAAAACTTTTTTAATTTACTACtttt------gagttTCTTTTCATTGACAGAGACCCAAGTCATATATTAAAATGATACTGATacttc--------------------------------------cgtaaTGGTCGGCATAGCTTAATTGCGGAGGACTGAAAATCCTTGTGTCACCATT--------------------------------------------------------------------------------------------AGTAAAATGAGAA-TGATACTTCAGTAGATGATACCTCAGTAATGGTGGACATAGC--TTTTTTTTTTTG--CAGAGGACTGAAAATC------CTTGTGTCACCATTCGTAAAATGAGGATGNNNNNNNNNNNNNNNNNNNNNNNNNNNNnnnn

trnLF_HD AATTGGATTGAGCCTTGGTATGGAAACCTACTAAGTGATAACTTTCAAATTCAGAGAAACCCTGGAATTAAcaatgggcAATCCTGAGCCAAATCCTGGTTTACGTGAACAAACCGGAGTTTAGAAAGCGAGAAAAAA-GGGATAGGTGCAGAGACTCAATGGAAGCTGTTCTAACAAATGGAGTTCACTACCTTGTGTTGATAAAGGAATCCTTCGATCAAAACTTCAAATCAAAAAG-GATGAAGGAGAAAAACCTATATTGTCTAAATATA------GGTAACACAAAA-CGATCTCAAAAATGACGACCTGAATCTCGATTTCTATTTTTTT--ATAAACAAAATCGAAATGATGTGAATCAATTCGAA-----GTTTAAGAAAtaatattcattGATCAAATGATTCTCTTCATAGTCTGATAGATCCGTGGTGGAActtattaaTCGGACGAGAATAAAGATAGAGTCCCATTTTAC-ATGTCAATACTGACAACAATGAAATTTATAGTAAGATGXXXNNNNNNNN-NNNNNNNNNNNNNNNNNNNNNNNNNNNNCTTTTT-TTAGGTTATTAGCGA-TTTGAATTATTTAGAATCTATATCAGTTTTCATTTTCAAACTTAGAAAGTCTTCTTTTATTTATAAGATCCAAGAAATTCCCGGTCCAAAACTTTTTTAATTTACTACtttt------gagttTCTTTTCATTGACAGAGACCCAAGTCATATATTAAAATGATACTGATacttc--------------------------------------cgtaaTGGTCGGTATAGCTTAATTGCGGAGGACTGAAAATCCTTGTGTCACCATT--------------------------------------------------------------------------------------------AGTAAAATGAGAA-TGATACTTCAGTAGATGATACCTCAGTAATGGTGGACATAGC--TTTTTTTTTTTG--CAGAGGACTGAAAATC------CTTGTGTCACCATTCGTAAAATGAGGATNNNNNNNNNNNNNNNNNNNNNNNNNNNNNnnnn

trnLF_HO AATTGGATTGAGCCTTGGTATGGAAACCTACTAAGTGATAACTTTCAAATTCAGAGAAACCCTGGAATTAAcaatgggcAATCCTGAGCCAAATCCTGGTTTAYGTGAACAAACCGGAGTTTAGAAAGCGAGAAAAAA-GGGATAGGTGCAGAGACTCAATGGAAGCTGTTCTAACAAATGGAGTTCACTACCTTGTGTTGATAAAGGAATCCTTCGATCAAAACTTCAAATCAAAAAG-GATGAAGGAGAAAAACCTATATTGTCTAAATATA------GGTAACACAAAA-CGATCTCAAAAATGACGACCTGAATCTCGATTTCTATTTTTTT--ATAAACAAAATCGAAATGATGTGAATCAATTCGAA-----GTTTAAGAAAtaatattcattGATCAAATGATTCTCTTCATAGTCTGATAGATCCGTGGTGGAActtattaaTCGGACGAGAATAAAGATAGAGTCCCATTTTAC-ATGTCAATACTGACAACAATGAAATTTATAGTAAGATGXXXGCTCTACT-CCCCAAAAAGGTCTGTTTGACACCTTACCTtttt-ttag-TTATTAGCGA-TTTGAATTATTTAGAATCTATATCAGTTTTCATTTTCAAACTTAGAAAGTCTTCTTTTATTTATAAGATCCAAGAAATTCCCGGTCCAAAACTTTTTTAATTTACTACTTTT------GAGTTTCTTTTCATTGACAGAGACCCAAGTCATATATTAAAATGATACTGATacttc--------------------------------------cgtaaTGGTCGGCATAGCTTAATTGCGGAGGACTGAAAATCCTTGTGTCACCATT--------------------------------------------------------------------------------------------AGTAAAATGAGAA-TGATACTTCAGTAGATGATACCTCAGTAATGGTGGACATAGC-TTTTTTTTTTTTG--CAGAGGACTGAAAATC------CTTGTGTCACCATTCGTAAAATGAGGATGATACTTCGGTAATGGCCGGGATAGCTCAgttg

trnLF_HP AATTGGATTGAGCCTTGGTATGGAAACCTACTAAGTGATAACTTTCAAATTCAGAGAAACCCTGGAATTAAcaatgggcAATCCTGAGCCAAATCCTGGTTTACGTGAACAAACCGGAGTTTAGAAAGCGAGAAAAAA-GGGATAGGTGCAGAGACTCAATGGAAGCTGTTCTAACAAATGGAGTTCACTACCTTGTGTTGATAAAGGAATCCTTCGATCAAAACTTCAAATCAAAAAG-GATGAAGGAGAAAAACCTATATTGTCTAAATATA------GGTAACACAAAA-CGATCTCAAAAATGACGACCTGAATCTCGATTTCTATTTTTTT--ATAAACAAAATCGAAATGATGTGAATCAATTCGAA-----GTTTAAGAAAtaatattcattGATCAAATGATTCTCTTCATAGTCTGATAGATCCGTGGTGGAActtattaaTCGGACGAGAATAAAGATAGAGTCCCATTTTAC-ATGTCAATACTGACAACAATGAAATTTATAGTAAGATGXXXGCTCTACT-CCCCAAAAAGGTCTGTTTGACACCTTACCTtttt-ttag-TTATTAGCGA-TTTGAATTATTTAGAATCTATATCAGTTTTCATTTTCAAACTTAGAAAGTCTTCTTTTATTTATAAGATCCAAGAAATTCCCGGTCCAAAACTTTTTTAATTTACTACTTTT-GTTTTGAGTTTCTTTTCATTGACAGAGACCCAAGTCATATATTAAAATGATACTGATacttc--------------------------------------cgtaaTGGTCGGCATAGCTTAATTGCGGAGGACTGAAAATCCTTGTGTCACCATT--------------------------------------------------------------------------------------------AGTAAAATGAGAA-TGATACTTCAGTAGATGATACCTCAGTAATGGTGGACATAGC--TTTTTTTTTTTG--CAGAGGACTGAAAATC------CTTGTGTCACCATTCGTAAAATGAGGATGATACTTCGGTAATGGCCGGGATAGCTCAgttg

trnLF_HQ AATTGGATTGAGCCTTGGTATGGAAACCTACTAAGTGATAACTTTCAAATTCAGAGAAACCCTGGAATTAAcaatgggcAATCCTGAGCCAAATCCTGGTTTAYGTGAACAAACCGGAGTTTAGAAAGCGAGAAAAAA-GGGATAGGTGCAGAGACTCAATGGAAGCTGTTCTAACAAATGGAGTTCACWACCTTGTGTTGATAAAGGAATCCTTCGATCAAAACTTCAAATMAAAAAG-GATGAAGGAGAAAAACCTATATTGTCTAAATATA------GGTAACACAAAA-CGATCTCAAAAATGACGACCTGAATCTCGATTTCTATTTTTTT--ATAAACAAAATCGAAATGATGTGAATCAATTCGAA-----GTTTAAGAAAtaatattcattGATCAAATGATTCTCTTCATAGTCTGATAGATCCGTGGTGGAActtattaaTCGGACGAGAATAAAGATAGAGTCCCATTTTAC-ATGTCAATACTGACAACAATGAAATTTATAGTAAGATGXXXGCTCTACT-CCCCAAAAAGGTCTGTTTGACACCTTACCTtttt-ttag-TTATTAGCGA-TTTGAATTATTTAGAATCTATATCAGTTTTCATTTTCAAACTTAGAAAGTCTTCTTTTATTTATAAGATCCAAGAAATTCCCGGTCCAAAACTTTTTTAATTTACTACtttt------gagttTCTTTTCATTGACAGAGACCCAAGTCATATATTAAAATGATACTGATacttc--------------------------------------cgtaaTGGTCGGCATAGCTTAATTGCAGAGGACTGAAAATCCTTGTGTCACCATT--------------------------------------------------------------------------------------------AGTAAAATGAGAA-TGATACTTCAGTAGATGATACCTCAGTAATGGTGGACATAGC--TTTTTTTTTTTG--CAGAGGACTGAAAATC------CTTGTGTCACCATTCGTAAAATGAGGATGATACTTCGGTAATGGCCGGGATAGCTCAgttg

trnLF_HR NNNNNNNNNNNNNNNNNNNNNNNNNNNNNNNNAAGTGATAACTTTCAAATTCAGAGAAACCCTGGAATTAAcaatgggcAATCCTGAGCCAAATCCTGGTTTACGTGAACAAACCGGAGTTTAGAAAGCGAGAAAAAA-GGGATAGGTGCAGAGACTCAATGGAAGCTGTTCTAACAAATGGAGTTCACTACCTTGTGTTGATAAAGGAATCCTTCGATCAAAACTTCAAATCAAAAAG-GATGAAGGAGAAAAACCTATATTGTCTAAATATA------GGTAACACAAAA-CGATCTCAAAAATGACGACCTGAATCTCGATTTCTATTTTTTT--ATAAACAAAATCGAAATGATGTGAATCAATTCGAA-----GTTTAAGAAAtaatattcattGATCAAATGATTCTCTTCATAGTCTGATAGATCCGTGGTGGAActtattaaTCGGACGAGAATAAAGATAGAGTCCCATTTTAC-ATGTCAATACTGACAACAATGAAATTTANNNNNNNNNNXXXGCTCTACT-CCCCAAAAAGGTCTGT--GACACCTTACCTtttt-ttag-TTATTATCGA-TTTGAATTATTTAGAATCTATATCAGTTTTCATTTTCAAACTTAGAAAGTCTTCTTTGATTTATAAGATCCAAGAAATTCCCGGTCCAAAACTTTTTTAATTTACTACtttt------gagttTCTTTTCATTGACAGAGACCCAAGTCATATATTAAAATGATACTGATacttc--------------------------------------cgtaaTGGTCGGCATAGCTTAATTGCGGAGGACTGAAAATCCTTGTGTCACCATT--------------------------------------------------------------------------------------------AGTAAAATGAGAA-TGATACTTCAGTAGATGATACCTCAGTAATGGTGGACATAGC--TTTTTTTTTTTG--CAGAGGACTGAAAATC------CTTGTGTCAC--------AAATGAGGATGATACTTCGGTAATGGCCGGGATAGCTCAgttg

trnLF_HS AATTGGATTGAGCCTTGGTATGGAAACCTACTAAGTGATAACTTTCAAATTCAGAGAAACCCTGGAATTAAcaatgggcAATCCTGAGCCAAATCCTGGTTTACGTGAACAAACCGGAGTTTAGAAAGCGAGAAAAAA-GGGATAGGTGCAGAGACTCAATGGAAGCTGTTCTAACAAATGGAGTTCACTACCTTGTGTTGATAAAGGAATCCTTCGATCAAAACTTCAAATCAAAAAG-GATGAAGGAGAAAAACCTATATTGTCTAAATATA------GGTAACACAAAA-CGATCTCAAAAATGACGACCTGAATCTCGATTTCTATTTTTTT--ATAAACAAAATCGAAATGATGTGAATCAATTCGAA-----GTTTAAGAAAtaatattcattGATCAAATGATTCTCTTCATAGTCTGATAGATCCGTGGTGGAActtattaaTCGGACGAGAATAAAGATAGAGTCCCATTTTAC-ATGTCAATACTGACAACAATGAAATTTATAGTAAGATGXXXGCTCTACT-CCCCAAAAAGGTCTGTTTGACACCTTACCTtttt-ttag-TTATTATCGA-TTTGAATTATTTAGAATCTATATCAGTTTTCATTTTCAAACTTAGAAAGTCTTCTTTTATTTATAAGATCCAAGAAATTCCCGGTCCAAAACTTTTTTAATTTACTAAtttt------gagttTCTTTTCATTGACAGAGACCCAAGTCATATATTAAAATGATACTGATacttc--------------------------------------cgtaaTGGTCGGCATAGCTTAATTGCGGAGGACTGAAAATCCTTGTGTCACCATT--------------------------------------------------------------------------------------------AGGAAAATGAGAA-TGATACTTCAGTAGATGATACCTCAGTAATGGTGGACATAGC--TTTTTTTTTGTG--CAGAGGACTGAAAATC------CTTGTGTCACCATTCGTAAAATAAGGATGATACTTCGGTAATGGCCGGGATAGCTCAgttg

trnLF_HT NNNNNNNNNNNNCCTTGGTATGGAAACCTACTAAGTGAGAACTTTCAAATTCAGAGAAACCCTGGAATTAAcaatgggcAATCCTGAGCCAAATCCTGGTTTACGTGAACAAACCGGAGTTTAGAAAGCGAGAAAAAA-GGGATAGGTGCAGAGACTCAATGGAAGCTGTTCTAACAAATGGAGTTCACTACCTTGTGTTGATAAAGGAATCCTTCGATCAAAACTTCAAATCAAAAAG-GATGAAGGAGAAAAACCTATATTGTCTAAATATA------GGTAACACAAAA-CGATCTCAAAAATGACGACCTGAATCTCGATTTCTATTTTTTT--ATAAACAAAATCGAAATGATGTGAATCAATTCGAA-----GTTTAAGAAAtaatattcattGATCAAATGATTCTCTTCATAGTCTGATAGATCCGTGGTGGAActtattaaTCGGACGAGAATAAAGATAGAGTCCCATTTTAC-ATGTCAATACTGACAACAATGAAATTTATAGTAAGATGXXXGCTCTACT-CCCCAAAAAGGTCTGTTTGACACCTTACCTtttt-ttag-TTATTATCGA-TTTGAATTATTTAGAATCTATATCAGTTTTCATTTTCAAACTTAGAAAGTCTTCTTTTATTTATAAGATCCAAGAAATTCCCGGTCCAAAACTTTTTTAATTTACTACtttt------gagttTCTTTTCATTGACAGAGACCCAAGTCATATATTAAAATGATACTGATacttc--------------------------------------cgtaaTGGTCGGCATAGCTTAATTGCGGAGGACTGAAAATCCTTGTGTCACCATT--------------------------------------------------------------------------------------------AGGAAAATGAGAA-TGATACTTCAGTAGATGATACCTCAGTAATGGTGGACATAGC--TTTTTTTTTGTG--CAGAGGACTGAAAATC------CTTGTGTCACCATTCGTAAAATAAGGATGATACTTCGGTAATGGCCGGGATAGCTCAgttg

trnLF_HU AATTGGATTGAGCCTTGGTATGGAAACCTACTAAGTGATAACTTTCAAATTCAGAGAAACCCTGGAATTAAcaatgggcAATCCTGAGCCAAATCCTGGTTTACGTGAACAAACCGGAGTTTAGAAAGCGAGAAAAAA-GGGATAGGTGCAGAGACTCAATGGAAGCTGTTCTAACAAATGGAGTTCACTACCTTGTGTTGATAAAGGAATCCTTCGATCAAAACTTCAAATCAAAAAG-GATGAAGGAGAAAAACCTATATTGTCTAAATATA------GGTAACACAAAA-CGATCTCAAAAATGACGACCTGAATCTCGATTTCTATTTTTTT--ATAAACAAAATCGAAATGATGTGAATCAATTCGAA-----GTTTAAGAAAtaatattcattGATCAAATGATTCTCTTCATAGTCTGATAGATCCGTGGTGGAActtattaaTCGGACGAGAATAAAGATAGAGTCCCATTTTAC-ATGTCAATACTGACAACAATGAAATTTATAGTAAGATGXXXGCTCTACT-CCCCAAAAAGGTCTGTTTGACACCTTACCTtttt-ttag-TTATTATCGA-TTTGAATTATTTAGAATCTATATCAGTTTTCATTTTCAAACTTAGAAAGTCTTCTTTTATTTATAAGATCCAAGAAATTCCCGGTCCAAAACTTTTTGAATTTACTACtttt------gagttTCTTTTCATTGACAGAGACCCAAGTCATATATTAAAATGATACTGATacttc--------------------------------------cgtaaTGGTCGGCATAGCTTAATTGCGGAGGACTGAAAATCCTTGTGTCACCATT--------------------------------------------------------------------------------------------------------------------------------------------------------------------------------------------------------CGTAAAATAAGGATGATACTTCGGTAATGGCCGGGATAGCTCAgttg

trnLF_II AATTGGATTGAGCCTTGGTATGGAAACCTACTAAGTGATAACTTTCAAATTCAGAGAAACCCTGGAATTAAcaatgggcAATCCTGAGCCAAATCCTGGTTTACGTGAACAAACCGGAGTTTAGAAAGCGAGAAAAAA-GGGATAGGTGCAGAGACTCAATGGAAGCTGTTCTAACAAATGGAGTTCACTACCTTGTGTTGATAAAGGAATCCTTCGATCAAAACTTCAAATCAAAAAG-GATGAAGGAGAAAAACCTATATTGTCTAAATATA------GGTAACACAAAA-CGATCTCAAAAATGACGACCTGAATCTCGATTTCTATTTTTTT--ATAAACAAAATCGAAATGATGTGAATCAATTCGAA-----GTTTAAGAAAtaatattcattGATCAAATGATTCTCTTCATAGTCTCATAGATCCGTGGTGGAActtattaaTCGGACGAGAATAAAGATAGAGTCCCATTTTAC-ATGTCAATACTGACAACAATGAAATTTATAGTAAGATGXXXGCTCTACT-CCCCAAAAAGGTCTGTTTGACACCTTACCTtttt-ttag-TTATTATGGA-TTTGAATTATTTAGAATCTATATCAGTTTTCATTTTCAAACTTAGAAAGTCTTCTTTGATTTATAAGATCCAAGAAATTCCCGGTCCAAAACTTTTTTAATTTACTACtttt------gagttTCTTTTCATTGACAGAGACCCAAGTCATATATTAAAATGATACTGATacttc--------------------------------------cgtaaTGGTCGGCATAGCTTAATTGCGGAGGACTGAAAATCCTTGTGTCACCATT--------------------------------------------------------------------------------------------AGTAAAATGAGAA-TGATACTTCAGTAGATGATACCTCAGTAATGGTGGACATAGC--TTTTTTTTTTTG--CAGAGGACTGAAAATC------CTTGTGTCAC--------AAATGAGGATGATACTTCGGTAATGGCCGGGATAGCTCAgttg

trnLF_IW AATTGGATTGAGCCTTGGTATGGAAACCTACTAAGTGATAACTTTCAAATTCAGAGAAACCCTGGAATTAAcaatgggcAATCCTGAGCCAAATCCTGGTTTACGTGAACAAACCGGAGTTTAGAAAGCGAGAAAAAA-GGGATAGGTGCAGAGACTCAATGGAAGCTGTTCTAACAAATGGAGTTCACTACCTTGTGTTGATAAAGGAATCCTTCGATCAAAACTTCAAATCAAAAAG-GATGAAGGAGAAAAACCTATATTGTCTAAATATA------GGTAACACAAAA-CGATCTCAAAAATGACGACCTGAATCTCGATTTCTATTTTTTT--ATAAACAAAATCGAAATGATGTGAATCAATTCGAA-----GTTTAAGAAAtaatattcattGATCAAATGATTCTCTTCATAGTCTGATAGATCCGTGGTGGAActtattaaTCGGACGAGAATAAAGATAGAGTCCCATTTTAC-ATGTCAATACTGACAACAATGAAATTTATAGTAAGATGXXXNNNNNNNN-NNNNNNNNNNNNNNNNNNNACACATTACCTtttt-ttag-TTATTATCGA-TTTGAATTATTTAGAATCTATATCAGTTTTCATTTTCAAACTTAGAAAGTCTTCTTTTATTTATAAGATCCAAGAAATTCCCGGTCCAAAACTTTTTTAATTTACTACtttt------gagttTCTTTTCATTGACAGAGACCCAAGTCATATATTAAAATGATACTGATacttc--------------------------------------cgtaaTGGTCGGCATAGCTTAATTGCGGAGGACTGAAAATCCTTGTGTCACCATT--------------------------------------------------------------------------------------------AGTAAAATGAGAA-TGATACTTCAGTAGATGATACCTCAGTAATGGTGGACATAGC--TTTTTTTTTTTG--CAGAGGACTGAAAATC------CTTGTGTCACCATTCGTAAAATGAGGATGATACTTCGGTAATGGCCGGGATAGCTCAgttg

trnLF_IZ AATTGGATTGAGCCTTGGTATGGAAACCTACTAAGTGATAACTTTCAAATTCAGAGAAACCCTGGAATTAAcaatgggcAATCCTGAGCCAAATCCTGGTTTACGTGAACAAACCGGAGTTTAGAAAGCGAGAAAAAA-GGGATAGGTGCAGAGACTCAATGGAAGCTGTTCTAACAAATGGAGTTCACTACCTTGTGTTGATAAAGGAATCCTTCGATCAAAACTTCAAATCAAAAAG-GATGAAGGAGAAAAACCTATATTGTCTAAATATA------GGTAACACAAAA-CGATCTCAAAAATGACGACCTGAATCTCGATTTCTATTTTTTT--ATAAACAAAATCGAAATGATGTGAATCAATTCGAA-----GTTTAAGAAAtaatattcattGATCAAATGATTCTCTTCATAGTCTGATAGATCCGTGGTGGAActtattaaTCGGACGAGAATAAAGATAGAGTCCCATTTTAC-ATGTCAATACTGACAACAATGAAATTTATAGTAAGATGXXXGCTCTACT-CCCCAAAAAGGTCTGTTTGACACCTTACCTtttt-ttag-TTATTATCGA-TTTGAATTATTTAGAATCTATATCAGTTTTCATTTTCAAACTTAGAAAGTCTTCTTTTATTTATAAGATCCAAGAAATTCCCGGTCCAAAACTTTTTTAATTTACTACtttt------gagttTCTTTTCATTGACAGAGACCCAAGTCATATATTAAAATGATACTGATacttc--------------------------------------cgtaaTGGTCGGCATAGCTTAATTGCCGAGGACTGAAAATCCTTGTGTCACCATT--------------------------------------------------------------------------------------------AGTAAAATGAGAA-TGATACTTCAGTAGATGATACCTCAGTAATGGTGGACATAGC-------TTTTTTG--CAGAGGACTGAAAATC------CTTGTGTCACCATTCGTAAAATGAGGATGATACTTCGGTAATGGCCGGGATAGCTCAgttg

trnLF_KB AATTGGATTGAGCCTTGGTATGGAAACCTACTAAGTGATAACTTTCAAATTCAGAGAAACCCTGGAATTAAcaatgggcAATCCTGAGCCAAATCCTGGTTTACGTGAACAAACCGGAGTTTAGAAAGCGAGAAAAAA-GGGATAGGTGCAGAGACTCAATGGAAGCTGTTCTAACAAATGGAGTTCACTACCTTGTGTTGATAAAGGAATCCTTCGATCAAAACTTCAAATCAAAAAG-GATGAAGGAGAAAAACCTATATTGTCTAAATATA------GGTAACACAAAA-CGATCTCAAAAATGACGACCTGAATCTCGATTTCTATTTTTTT--ATAAACAAAATCGAAATGATGTGAATCAATTCGAA-----GTTTAAGAAAtaatattcattGATCAAATGATTCTCTTCATAGTCTGATAGATCCGTGGTGGAActtattaaTCGGACGAGAATAAAGATAGAGTCCCATTTTAC-ATGTCAATACTGACAACAATGAAATTTATAGTAAGATGXXXGCTGTACT-CCCCAAAAAGGTCTGTTTGACACCTTACCTtttt-ttag-TTATTATCGA-TTTGAATTATTTAGAATCTATATCAGTTTTCATTTTCAAACTTAGAAAGTCTTCTTTTATTTATAAGATCCAAGAAATTCCCGGTCCAAAACTTTTTTAATTTACTACtttt------gagttTCTTTTCATTGACAGAGACCCAAGTCATATATTAAAATGATACTGATacttc--------------------------------------cgtaaTGGTCGGCATAGCTTAATTGCGGAGGACTGAAAATCCTTGTGTCACCATT--------------------------------------------------------------------------------------------AGTAAAATGAGAA-TGATACTTCAGTAGATGATACCTCAGTAATGGTGGACATAGC--TTTTTTTTTTTG--CAGAGGACTGAAAATC------CTTGTGTCACCATTCGTAAAATGAGGATGATACTTCGGTAATNNNNNNNNNNNNNNNnnnn

trnLF_KE AATTGGATTGAGCCTTGGTATGGAAACCTACTAAGTGATAACTTTCAAATTCAGAGAAACCCTGGAATTAAcaatgggcAATCCTGAGCCAAATCCTGGTTTACGTGAACAAACCGGAGTTTAGAAAGCGAGAAAAAA-GGGATAGGTGCAGAGACTCAATGGAAGCTGTTCTAACAAATGGAGTTCACTACCTTGTGTTGATAAAGGAATCCTTCGATCAAAACTTCAAATCAAAAAG-GATGAAGGAGAAAAACCTATATTGTCTAAATATA------GGTAACACAAAA-CGATCTCAAAAATGACGACCTGAATCTCGATTTCTATTTTTTT--ATAAACAAAATCGAAATGATGTGAATCAATTCGAA-----GTTTAAGAAAtaatattcattGATCAAATGATTCTCTTCATAGTCTAATAGATCCGTGGTGGAActtattaaTCGGACGAGAATAAAGATAGAGTCCCATTTTAC-ATGTCAATACTGACAACAATGAAATTTATAGTAAGATGXXXNNTGTACT-CCCCAAAAAGGTCTGTTTGACACCTTACCTtttt-ttag-TTATTATCGA-TTTGAATTATTTCGAATCTATATCAGTTTTCATTTTCAAACTTAGAAAGTCTTCTTTTATTTATAAGATCCAAGAAATTCCCGGTCCAAAACTTTTTTAATTTACTACtttt------gagttTCTTTTCATTGACAGAGACCCAAGTCATATATTAAAATGATACTGATacttc--------------------------------------cgtaaTGGTCGGCATAGCTTAATTGCGGAGGACTGAAAATCCTTGTGTCACCATT--------------------------------------------------------------------------------------------AGTAAAATGAGAA-TGATACTTCAGTAGATGATACCTCAGTAATGGTGGACATAGC--TTTTTTTTTTTG--CAGAGGACTGAAAATC------CTTGTGTCACCATTCGTAAAATGAGGATGATACTTCGGTAATGGCCGGGATAGCTCAgttg

Arab1992 AATTGGATTGAGCCTTGGTATGGAAACCTACTAAGTGATAACTTTCAAATTCAGAGAAACCCTGGAATTAACAATGGGCAATCCTGAGCCAAATCCTGGTTTATGTGAACAAACCGGAGTTTAGAAAGCGAGAAAAAA-GGGATAGGTGCAGAGACTCAATGGAAGCTGTTCTAACAAATGGAGTTCACAACCTTGTGTTGATAAAGGAATCCTTCGATCAAAACTTAAAATAAAAAAG-GATGAAGGAGAAAAACCTATATTGTCTAAATATA------GGTAACACAAAA-CGATCTCAAAAATGACGACCTGAATCTCGATTTCTATTTTTTT--ATAAACAAAATCGAAATGATGTGAATCAATTCGAATCGAAGTTTAAGAAATAATATTCATTGATCAAATGATTCTCTTCATAGTCTGATAGATCCGTGGTGGAACTTATTAATCGGACGAGAATAAAGATAGAGTCCCATTTTAC-ATGTCAATACTGACAACAATGAAATTTATnnnnnnnnnXXXnnnnnnnnnnnnnnnnnnnnnnnnnnnnnnnnnnnnCCTTTTT-GTAG-TTATTATCCA-TTTGAATTATTTAGAATCTATATCAGTTTTCATTTTCAAACTTAGAAAGTCTTCTTTTATTTATAAGATCCAAAAAATTCCCGGTCCAAAACTTTTTTAATTTACTACTTTT------GAGTTTCTTTTCATTGACAGAGACCCAAGTCATATATTAAAATGATACTAATACTTC--------------------------------------CGTAATGGTCGGCATAGCTTAATTGCGGAGGACTGAAAATCCTTGTGTCACCATT--------------------------------------------------------------------------------------------------------------------------------------------------------------------------------------------------------CGTAAAATGAGGATGATACTTCGGTAATGGCCGGGATAGCTCAGTTG

AY257721 AATTGGATTGAGCCTTGGTATGGAAACCTACTAAGTGATAACTTTCAAATTCAGAGAAACCCTGGAATTAAcaatgggcAATCCTGAGCCAAATCCTGGTTTACGTGAACAAACCGTAGTTTAGAAAGCGAGAAAAAA-GGGATAGGTGCAGAGACTCAATGGAAGCTGTTCTAACAAATGGAGTTCACTACCTTGTGTTGATAAAGGAATCCTTCGATCAAAACTTCAAATCAAAAAG-GATGAAGGAGAAAAACCTATATTGTCTAAATATA------GGTAACACAAAA-CGATCGCAAAAATGACGACCTGAATCTCGATTTCTATTTTTTT--ATAAACAAAATCGAAATGATGTGAATCAATTCGAA-----GTTTAAGAAAtaatattcattGATCAAATGATTCTCTTCATAGTCTGATAGATCCGTGGTGGAActtattaaTCGGCCGAGAATAAAGATAGAGTCCCATTTTAC-ATGTCAATACTGACAACAATGAAATTTATAGTAAGATGXXXGCTCTACT-CCCCAAAAAGGTCTGTTTGACACCTTACCTtttt-ttag-TTATTATCCA-TTTGAATTATTTAGAATCTATATCAGTTTTCATTTTCAAACTTAGAAAGTCTTCTTTTATTTATAAGATCCAAGAAATTCCCGGTCCAAAACTTTTTTAATTTACTACtttt------gagttTCTGTTCATTGACAGAGACCCAAGTCATATATTAAAATGATACTGATacttc--------------------------------------cgtaaTGGTCGGCATAGCTTAATTGCGGAGGACTGAAAATCCTTGTGTCACCATT--------------------------------------------------------------------------------------------AGTAAAATGATAC-TGATACTTCAGTAGATGATACCTC--------------------------------------------------------------------------------------------GGTAATGGCCGGGATAGCTCAgttg

AY257722 AATTGGATTGAGCCTTGGTATGGAAACCTACTAAGTGATAACTTTCAAATTCAGAGAAACCCTGGAATTAAcaatgggcAATCCTGAGCCAAATCCTGGTTTACGTGAACAAACCGGAGTTTAGAAAGCGAGAAAAAA-GGGATAGGTGCAGAGACTCAATGGAAGCTGTTCTAACAAATGGAGTTCACTACCTTGTGTTGATAAAGGAATCCTTCGATCAAAACTTCAAATCAAAAAG-GATGAAGGAGAAAAACCTATATTGTCTAAATATA------GGTAACACAAAA-CGATCGAAAAAATGACGACCTGAATCTCGATTTCTATTTTTTT--ATAAACAAAATCGAAATGATGTGAATCAATTCGAA-----GTTTAAGAAAtaatattcattGATCAAATGATTCTCTTCATAGTCTGATAGATCCGTGGTGGAActtattaaTCGGCCGAGAATAAAGATAGAGTCCCATTTTAC-ATGTCAATACTGACAACAATGAAATTTATAGTAAGATGXXXGCTCTACT-CCCCAAAAAGGTCTGTTTGACACCTTACCTtttt-ttag-TTATTATCCA-TTTGAATTATTTAGAATCTATATCAGTTTTCATTTTCAAACTTAGAAAGTCTTCTTTTATTTATAAGATCCAAGAAATTCCCGGTCCAAAACTTTTTTAATTTACTACtttt------gagttTCTGTTCATTGACAGAGACCCAAGTCATATATTAAAATGATACTGATacttc--------------------------------------cgtaaTGGTCGGCATAGCTTAATTGCGGAGGACTGAAAATCCTTGTGTCACCATT--------------------------------------------------------------------------------------------AGTAAAATGATAC-TGATACTTCAGTAGATGATACCTCAGTAATGGTGGACATAGC-------TTTTTTG--CAGAGGACTGAAAATC------CTTGTGTCACCATTCGTAAAATGAGGATGATACTTCGGTAATGGCCGGGATAGCTCAgttg

AY257723 AATTGGATTGAGCCTTGGTATGGAAACCTACTAAGTGATAACTTTCAAATTCAGAGAAACCCTGGAATTAAcaatgggcAATCCTGAGCCAAATCCTGGTTTACGTGAACAAACCGGAGTTTAGAAAGCGAGAAAAAA-GGGATAGGTGCAGAGACTCAATGGAAGCTGTTCTAACAAATGGAGTTCACTACCTTGTGTTGATAAAGGAATCCTTCGATcaaaaCTTCAAATCAAAAAG-GATGAAGGAGAAAAACCTATATTGTCTAAATATA------GGTAACACAAAA-CGATCGCAAAAATGACGACCTGAATCTCGATTTCTATTTTTTT--ATAAACAAAATCGAAATGATGTGAATCAATTCGAA-----GTTTAAGAAAtaatattcattGATCAAATGATTCTCTTCATAGTCTGATAGATCCGTGGTGGAActtattaaTCGGCTGAGAATAAAGATAGAGTCCCATTTTAC-ATGTCAATACTGACAACAATGAAATTTATAGTAAGATGXXXGCTCTACT-CCCCAAAAAGGTCTGTTTGACACCTTACCTtttt-ttag-TTATTATCCA-TTTGAATTATTTAGAATCTATATCAGTTTTCATTTTCAAACTTAGAAAGTCTTCTTTTATTTATAAGATCCAAGAAATTCCCGGTCCAAAACTTTTTTAATTTACTACtttt------gagttTCTGTTCATTGACAGAGACCCAAGTCATATATTAAAATGATACTGATacttc--------------------------------------cgtaaTGGTCGGCATAGCTTAATTGCGGAGGACTGAAAATCCTTGTGTCACCATT--------------------------------------------------------------------------------------------AGTAAAATGATAC-TGATACTTCAGTAGATGATACCTCAGTAATGGTGGACATAGC-------TTTTTTG--CAGAGGACTGAAAATC------CTTGTGTCACCATTCGTAAAATGAGGATGATACTTCGGTAATGGCCGGGATAGCTCAgttg

AY257724 AATTGGATTGAGCCTTGGTATGGAAACCTACTAAGTGATAACTTTCAAATTCAGAGAAACCCTGGAATTAAcaatgggcAATCCTGAGCCAAATCCTGGTTTACGTGAACAAACCGGAGTTTAGAAAGCGAGAAAAAA-GGGATAGGTGCAGAGACTCAATGGAAGCTGTTCTAACAAATGGAGTTCACTACCTTGTGTTGATAAAGGAATCCTTCGATCAAAACTTCAAATCAAAAAG-GATGAAGGAGAAAAACCTATATTGTCTAAATATA------GGTAACACAAAA-CGATCGCAAAAATGACGACCTGAATCTCGATTTCTATTTTTTT--ATAAACAAAATCGAAATGATGTGAATCAATTCGAA-----GTTTAAGAAAtaatattcattGATCAAATGATTCTCTTCATAGTCTGATAGACCCGTGGTGGAActtattaaTCGGCCGAGAATAAAGATAGAGTCCCATTTTAC-ATGTCAATACTGACAACAATGAAATTTATAGTAAGATGXXXGCTCTACT-CCCCAAAAAGGTCTGTTTGACACCTTACCTtttt-ttag-TTATTATCCA-TTTGAATTATTTAGAATCTATATCAGTTTTCATTTTCAAACTTAGAAAGTCTTCTTTTATTTATAAGATCCAAGAAATTCCCGGTCCAAAACTTTTTTAATTTACTACtttt------gagttTCTGTTCATTGACAGAGACCCAAGTCATATATTAAAATGATACTGATacttc--------------------------------------cgtaaTGGTCGGCATAGCTTAATTGCGGAGGACTGAAAATCCTTGTGTCACCATT--------------------------------------------------------------------------------------------AGTAAAATGATAC-TGATACTTCAGTAGATGATACCTCAGTAATGGTGGACATAGC-------TTTTTTG--CAGAGGACTGAAAATC------CTTGTGTCACCATTCGTAAAATGAGGATGATACTTCGGTAATGGCCGGGATAGCTCAgttg

AY257725 AATTGGATTGAGCCTTGGTATGGAAACCTACTAAGTGATAACTTTCAAATTCAGAGAAACCCTGGAATTAAcaatgggcAATCCTGAGCCAAATCCTGGTTTACGTGAACAAACCGGAGTTTAGAAAGCGAGAAAAAA-GGGATAGGTGCAGAGACTCAATGGAAGCTGTTCTAACAAATGGAGTTCACTACCTTGTGTTGATAAAGGAATCCTTCGATCAAAACTTCAAATCAAAAAG-GATGAAGGAGAAAAACCTATATTGTCTAAATATA------GGTAACACAAAA-CGATCGCAAAAATGACGACCTGAATCTCGATTTCTATTTTTTT--ATAAACAAAATCGAAATGATGTGAATCAATTCGAA-----GTTTAAGAAAtaatattcattGATCAAATGATTCTCTTCATAGTCTGATAGATCCGTGGTGGAActtattaaTCGGCCGAGAATAAAGATAGAGTCCCATTTTAC-ATGTCAATACTGACAACAATGAAATTTATAGTAAGATGXXXGCTCTACT-CCCCAAAAAGGTCTGTTTGACACCTTACCTtttt-ttag-TTATTATCCA-TTTGAATTATTTAGAATCTATATCAGTTTTCATTTTCAAACTTAGAAAGTCTTCTTTTATTTATAAGATCCAAGAAATTCCCGGTCCAAAACTTTTTTAATTTACTACtttt------gagttTCTGTTCATTGACAGAGACCCAAGTCATATATTAAAATGATACTGATacttc--------------------------------------cgtaaTGGTCGGCATAGCTTAATTGCGGAGGACTGAAAATCCTTGTGTCACCATT--------------------------------------------------------------------------------------------AGTAAAATGATAC-TGATACTTCAGTAGATGATACCTCAGTAATGGTGGACATAGC-------TTTTTTG--CAGAGGACTGAAAATC------CTTGTGTCACCATTCGTAAAATGAGGATGATACTTCGGTAATGGCCGGGATAGCTCAgttg

AY257726 AATTGGATTGAGCCTTGGTATGGAAACCTACTAAGTGATAACTTTCAAATTCAGAGAAACCCTGGAATTAAcaatgggcAATCCTGAGCCAAATCCTGGTTTACGTGAACAAACCGGAGTTTAGAAAGCGAGAAAAAA-GGGATAGGTGCAGAGACTCAATGGAAGCTGTTCTAACAAATGGAGTTCACTACCTTGTGTTGATAAAGGAATCCTTCGATCAAAACTTCAAATCAAAAAG-GATGAAGGAGAAAAACCTATATTGTCTAAATATA------GGTAACACAAAA-CGATCGCAAAAATGACGACCTGAATCTCGATTTCTATTTTTTT--ATAAACAAAATCGAAATGATGTGAATCAATTCGAA-----GTTTAAGAAAtaatattcattGATCAAATGATTCTCTTCATAGTCTGATAGATCCGTGGTGGAActtattaaTCGGCCGAGAATAAAGATAGAGTCCCATTTTAC-ATGTCAATACTGACAACAATGAAATTTATAGTAAGATGXXXGCTCTACT-CCCCAAAAAGGTCTGTTTGACACCTTACCTtttt-ttag-TTATTATCCA-TTTGAATTATTTAGAATCTATATCAGTTTTCATTTTCAAACTTAGAAAGTCTTCTTTTATTTATAAGATCCAAGAAATTCCCGGTCCAAAACTTTTTTAATTTACTACtttt------gagttTCTGTTCATTGACAGAGACCCAAGTCATATATTAAAATGATACTGATacttc--------------------------------------cgtaaTGGTCGGCATAGCTTAATTGCGGAGGACTGAAAATCCTTGTGTCACCATT--------------------------------------------------------------------------------------------------------------------------------------------------------------------------------------------------------CGTAAAATGAGGATGATACTTCGGTAATGGCCGGGATAGCTCAgttg

AY257727 AATTGGATTGAGCCTTGGTATGGAAACCTACTAAGTGATAACTTTCAAATTCAGAGAAACCCTGGAATTAAcaatgggcAATCCTGAGCCAAATCCTGGTTTACGTGAACAAACCGGAGTTTAGAAAGCGAGAAAAAA-GGGATAGGTGCAGAGACTCAATGGAAGCTGTTCTAACAAATGGAGTTCACTACCTTGTGTTGATAAAGGAATCCTTCGATCAAAACTTCAAATCAAAAAG-GATGAAGGAGAAAAACCTATATTGTCTAAATATA------GGTAACACAAAA-CGATCGCAAAAATGACGACCTGAATCTCGATTTCTATTTTTTT--ATAAACAAAATCGAAATGATGTGAATCAATTCGAA-----GTTTAAGAAAtaatattcattGATCAAATGATTCTCTTCATAGTCTGATAGATCCGTGGTGGAActtattaaTCGGCCGAGAATAAAGATAGAGTCCCATTTTAC-ATGTCAATACTGACAACAATGAAATTTATAGTAAGATGXXXGCTCTACT-CCCCAAAAAGGTCTGTTTGACACCTTACCTtttt-ttag-TTATTATCCA-TTTGAATTATTTAGAATCTATATCAGTTTTCATTTTCAAACTTAGAAAGTCTTCTTTTATTTATAAGATCCAAGAAATTCCCGGTCCAAAACTTTTTTAATTTACTACtttt------gagttTCTGTTCATTGACAGAGACCCAAGTCATATATTAAAATGATACTGATacttc--------------------------------------cgtaaTGGTCGGCATAGCTTAATTGCGGAGGACTGAAAATCCTTGTGTCACCATT--------------------------------------------------------------------------------------------------------------------------------------------------------------------------------------------------------AGTAAAATGAGGATGATACTTCGGTAATGGCCGGGATAGCTCAgttg

AY257728 NNNNNNNNNNNNNNNNNNNNTGGAAACCTACTAAGTGATAACTTTCAAATTCAGAGAAACCCTGGAATTAAcaatgggcAATCCTGAGCCAAATCCTGGTTTACGTGAACAAACCGGAGTTTAGAAAGCGAGAAAAAA-GGGATAGGTGCAGAGACTCAATGGAAGCTGTTCTAACAAATGGAGTTCACTACCTTGTGTTGATAAAGGAATCCTTCGATCAAAACTTCAAATAAAAAAG-GATGAAGGAGAAAAACCTATATTGTCTAAATATA------GGTAACACAAAA-CGATCGCAAAAATGACGACCTGAATCTCGATTTCTATTTTTTT--ATAAACAAAATCGAAATGATGTGAATCAATTCGAA-----GTTTAAGAAAtaatattcattGATCAAATGATTCTCTTCATAGTCTGATAGATCCGTGGTGGAActtattaaTCGGCCGAGAATAAAGATAGAGTCCCATTTTAC-ATGTCAATACTGACAACAATGAAATTTATAGTAAGATGXXXGCTCTACT-CCCCAAAAAGGTCTGTTTGACACCTTACCTtttt-ttag-TTATTATCCA-TTTGAATTATTTAGAATCTATATCAGTTTTCATTTTCAAACTTAGAAAGTCTTCTTTTATTTATAAGATCCAAGAAATTCCCGGTCCAAAACTTTTTTAATTTACTACtttt------gagttTCTGTTCATTGACAGAGACCCAAGTCATATATTAAAATGATACTGATacttc--------------------------------------cgtaaTGGTCGGCATAGCTTAATTGCGGAGGACTGAAAATCCTTGTGTCACCACT--------------------------------------------------------------------------------------------AGTAAAATGATAC-TGATACTTCAGTAGATGATACCTCAGTAATGGTGGACATAGC-------TTTTTTG--CAGAGGACTGAAAATC------CTTGTGTCACCATTCGTAAAATGAGGATGATACTTCGGTAATGGCCGGGATAGCTCAgttg

AY257729 AATTGGATTGAGCCTTGGTATGGAAACCTACTAAGTGATAACTTTCAAATTCAGAGAAACCCTGGAATTAAcaatgggcAATCCTGAGCCAAATCCTGGTTTACGTGAACAAACCGGAGTTTAGAAAGCGAGAAAAAA-GGGATAGGTGCAGAGACTCAATGGAAGCTGTTCTAACAAATGGAGTTCACTACCTTGTGTTGATAAAGGAATCCTTCGATCAAAACTTCAAATAAAAAAG-GATGAAGGAGAAAAACCTATATTGTCTAAATATA------GGTAACACAAAA-CGATCGCAAAAATGACGACCTGAATCTCGATTTCTATTTTTTT--ATAAACAAAATCGAAATGATGTGAATCAATTCGAA-----GTTTAAGAAAtaatattcattGATCAAATGATTCTCTTCATAGTCTGATAGATCCGTGGTGGAActtattaaTCGGACGAGAATAAAGATAGAGTCCCATTTTAC-ATGTCAATACTGACAACAATGAAATTTATAGTAAGATGXXXGCTCTACT-CCCCAAAAAGGTCTGTTTGACACCTTACCTtttt-ttag-TTATTATCCA-TTTGAATTATTTAGAATCTATATCAGTTTTCATTTTCAAACTTAGAAAGTCTTCTTTTATTTATAAGATCCAAGAAATTCCCGGTCCAAAACTTTTTTAATTTACTACtttt------gagttTCTGTTCATTGACAGAGACCCAAGTCATATATTAAAATGATACTGATacttc--------------------------------------cgtaaTGGTCGGCATAGCTTAATTGCGGAGGACTGAAAATCCTTGTGTCACCATT--------------------------------------------------------------------------------------------AGTAAAATGATAC-TGATACTTCAGTAGATGATACCTCAGTAATGGTGGACATAGC-------TTTTTTG--CAGAGGACTGAAAATC------CTTGTGTCACCATTCGTAAAATGAGGATGATACTTCGGTAATGGCCGGGATAGCTCAgttg

AY257730 AATTGGATTGAGCCTTGGTATGGAAACCTACTAAGTGATAACTTTCAAATTCAGAGAAACCCTGGAATTAAcaatgggcAATCCTGAGCCAAATCCTGGTTTACGTGAACAAACCGGAGTTTAGAAAGCGAGAAAAAA-GGGATAGGTGCAGAGACTCAATGGAAGCTGTTCTAACAAATGGAGTTCACTACCTTGTGTTGATAAAGGAATCCTTCGATCAAAACTTCAAATAAAAAAG-GATGAAGGAGAAAAACCTATATTGTCTAAATATA------GGTAACACAAAA-CGATCGCAAAAATGACGACCTGAATCTCGATTTCTATTTTTTT--ATAAACAAAATCGAAATGATGTGAATCAATTCGAA-----GTTTAAGAAAtaatattcattGATCAAATGATTCTCTTCATAGTCTGATAGATCCGTGGTGGAActtattaaTCGGCCGAGAATAAAGATAGAGTCCCATTTTAC-ATGTCAATACTGACAACAATGAAATTTATAGTAAGATGXXXGCTCTACT-CCCCAAAAAGGTCTGTTTGACACCTTACCTtttt-ttag-TTATTATCCA-TTTGAATTATTTAGAATCTATATAAGTTTTCATTTTCAAACTTAGAAAGTCTTCTTTTATTTATAAGATCCAAGAAATTCCCGGTCCAAAACTTTTTTAATTTACTACtttt------gagttTCTGTTCATTGACAGAGACCCAAGTCATATATTAAAATGATACTGATacttc--------------------------------------cgtaaTGGTCGGCATAGCTTAATTGCGGAGGACTGAAAATCCTTGTGTCACCATT--------------------------------------------------------------------------------------------AGTAAAATGATAC-TGATACTTCAGTAGATGATACCTCAGTAATGGTGGACATAGC-------TTTTTTG--CAGAGGACTGAAAATC------CTTGTGTCACCATTCGTAAAATGAGGATGATACTTCGGTAATGGCCGGGATAGCTCAgttg

AY257731 AATTGGATTGAGCCTTGGTATGGAAACCTACTAAGTGATAACTTTCAAATTCAGAGAAACCCTGGAATTAAcaatgggcAATCCTGAGCCAAATCCTGGTTTACGTGAACAAACCGGAGTTTAGAAAGCGAGAAAAAA-GGGATAGGTGCAGAGACTCAATGGAAGCTGTTCTAACAAATGGAGTTCACTACCTTGTGTTGATAAAGGAATCCTTCGATCAAAACTTCAAATAAAAAAG-GATGAAGGAGAAAAACCTATATTGTCTAAATATA------GGTAACACAAAA-CGATCGCAAAAATGACGACCTGAATCTCGATTTCTATTTTTTT--ATAAACAAAATCGAAATGATGTGAATCAATTCGAA-----GTTTAAGAAAtaatattcattGATCAAATGATTCTCTTCATAGTCTGATAGATCCGTGGTGGAActtattaaTCGGCCGAGAATAAAGATAGAGTCCCATTTTAC-ATGTCAATACTGACAACAATGAAATTTATAGTAAGATGXXXGCTCTACT-CCCCAAAAAGGTCTGTTTGACACCTTACCTtttt-ttag-TTATTATCCA-TTTGAATTATTTAGAATCTATATCAGTTTTCATTTTCAAACTTAGAAAGTCTTCTTTTATTTATAAGATCCAAGAAATTCCCGGTCCAAAACTTTTTTAATTTACTACtttt------gagttTCTGTTCATTGACAGAGACCCAAGTCATATATTAAAATGATACTGATacttc--------------------------------------cgtaaTGGTCGGCATAGCTTAATTGCGGAGGACTGAAAATCCTTGTGTCACCATT--------------------------------------------------------------------------------------------AGTAAAATGATAC-TGATACTTCAGTAGATGATACCTCAGTAATGTTGGACATAGC-------TTTTTTG--CAGAGGACTGAAAATC------CTTGTGTCACCATTCGTAAAATGAGGATGATACTTCGGTAATGGCCGGGATAGCTCAgttg

AY257732 AATTGGATTGAGCCTTGGTATGGAAACCTACTAAGTGATAACTTTCAAATTCAGAGAAACCCTGGAATTAAcaatgggcAATCCTGAGCCAAATCCTGGTTTACGTGAACAAACCGGAGTTTAGAAAGCGAGAAAAAA-GGGATAGGTGCAGAGACTCAATGGAAGCTGTTCTAACAAATGGAGTTCACTACCTTGTGTTGATAAAGGAATCCTTCGATCAAAACTTCAAATAAAAAAG-GATGAAGGAGAAAAACCTATATTGTCTAAATATA------GGTAACACAAAA-CGATCGCAAAAATGACGACCTGAATCTCGATTTCTATTTTTTT--ATAAACAAAATCGAAATGATGTGAATCAATTCGAA-----GTTTAAGAAAtaatattcattGATCAAATGATTCTCTTCATAGTCTGATAGATCCGTGGTGGAActtattaaTCGGCCGAGAATAAAGATAGAGTCCCATTTTAC-ATGTCAATACTGACAACAATGAAATTTATAGTAAGATGXXXNNTCTACT-CCCCAAAAAGGTCTGTTTGACACCTTACCTtttt-ttag-TTATTATCCA-TTTGAATTATTTAGAATCTATATCAGTTTTCATTTTCAAACTTAGAAAGTCTTCTTTTATTTATAAGATCCAAGAAATTCCCGGTCCAAAACTTTTTTAATTTACTACtttt------gagttTCTGTTCATTGACAGAGACCCAAGTCATATATTAAAATGATACTGATacttc--------------------------------------cgtaaTGGTCGGCATAGCTTAATTGCGGAGGACTGAAAATCCTTGTGTCACCATT--------------------------------------------------------------------------------------------AGTAAAATGATAG-TGATACTTCAGTAGATGATACCTCAGTAATGGTGGACATAGC-------TTTTTTG--CAGAGGACTGAAAATC------CTTGTGTCACCATTCGTAAAATGAGGATGATACTTCGGTAATGGCCGGGATAGCTCAgttg

AY257733 AATTGGATTGAGCCTTGGTATGGAAACCTACTAAGTGATAACTTTCAAATTCAGAGAAACCCTGGAATTAAcaatgggcAATCCTGAGCCAAATCCTGGTTTACGTGAACAAACCGGAGTTTAGAAAGCGAGAAAAAA-GGGATAGGTGCAGAGACTCAATGGAAGCTGTTCTAACAAATGGAGTTCACTACCTTGTGTTGATAAAGGAATCCTTCGATCAAAACTTCAAATAACAAAG-GATGAAGGAGAAAAACCTATATTGTCTAAATATA------GGTAACACAAAA-CGATCGCAAAAATGACGACCTGAATCTCGATTTCTATTTTTTT--ATAAACAAAATCGAAATGATGTGAATCAATTCGAA-----GTTTAAGAAAtaatattcattGATCAAATGATTCTCTTCATAGTCTGATAGATCCGTGGTGGAActtattaaTCGGCCGAGAATAAAGATAGAGTCCCATTTTAC-ATGTCAATACTGACAACAATGAAATTTATAGTAAGATGXXXGCTCTACT-CCCCAAAAAGGTCTGTTTGACACCTTACCTtttt-ttag-TTATTATCCA-TTTGAATTATTTAGAATCTATATCAGTTTTCATTTTCAAACTTAGAAAGTCTTCTTTTATTTATAAGATCCAAGAAATTCCCGGTCCAAAACTTTTTTAATTTACTACtttt------gagttTCTGTTCATTGACAGAGACCCAAGTCATATATTAAAATGATACTGATacttc--------------------------------------cgtaaTGGTCGGCATAGCTTAATTGCGGAGGACTGAAAATCCTTGTGTCACCATT--------------------------------------------------------------------------------------------AGTAAAATGATAG-TGATACTTCAGTAGATGATACCTCAGTAATGGTGGACATAGC-------TTTTTTG--CAGAGGACTGAAAATC------CTTGTGTCACCATTCGTAAAATGAGGATGATACTTCGGTAATGGCCGGGATAGCTCAgttg

AY257734 AATTGGATTGAGCCTTGGTATGGAAACCTACTAAGTGATAACTTTCAAATTCAGAGAAACCCTGGAATTAAcaatgggcAATCCTGAGCCAAATCCTGGTTTACGTGAACAAACCGGAGTTTAGAAAGCGAGAAAAAA-GGGATAGGTGCAGAGACTCAATGGAAGCTGTTCTAACAAATGGAGTTCACTACCTTGTGTTGATAAAGGAATCCTTCGATCAAAACTTCAAATAAAAAAG-GATGAAGGAGAAAAACCTATATTGTCTAAATATA------GGTAACACAAAA-CGATCGCAAAAATGACGACCTGAATCTCGATTTCTATTTTTTT--ATAAACAAAATCGAAATGATGTGAATCAATTCGAA-----GTTTAAGAAAtaatattcattGATCAAATGATTCTCTTCATAGTCTGATAGATCCGTGGTGGAActtattaaTCGGCCGAGAATAAAGATAGAGTCCCATTTTAC-ATGTCAATACTGACAACAATGAAATTTATAGTAAGATGXXXGCTCTACT-CCCCAAAAAGGTCTGTTTGACACCTTACCTtttt-ttag-TTATTATCCA-TTTGAATTATTTAGAATCTATATCAGTTTTCATTTTCAAACTTAGAAAGTCTTCTTTTATTTATAAGATCCAAGAAATTCCCGGTCCAAAACTTTTTTAATTTACTACtttt------gagttTCTGTTCATTGACAGAGACCCAAGTCATATATTAAAATGATACTGATacttc--------------------------------------cgtaaTGATCGGCATAGCTTAATTGCGGAGGACTGAAAATCCTTGTGTCACCATT--------------------------------------------------------------------------------------------AGTAAAATGATAC-TGATACTTCAGTAGATGATACCTCAGTAATGGTGGACATAGC-------TTTTTTG--CAGAGGACTGAAAATC------CTTGTGTCACCATTCGTAAAATGAGGATGATACTTCGGTAATGGCCGGGATAGCTCAgttg

AY257735 AATTGGATTGAGCCTTGGTATGGAAACCTACTAAGTGATAACTTTCAAATTCAGAGAAACCCTGGAATTAAcaatgggcAATCCTGAGCCAAATCCTGGTTTACGTGAACAAACCGGAGTTTAGAAAGCGAGAAAAAA-GGGATAGGTGCAGAGACTCAATGGAAGCTGTTCTAACAAATGGAGTTCACTACCTTGTGTTGATAAAGGAATCCTTCGATCAAAACTTCAAATAAAAAAGTGATGAAGGAGAAAAACCTATATTGTCTAAATATA------GGTAACACAAAA-CGATCGCAAAAATGACGACCTGAATCTCGATTTCTATTTTTTT--ATAAACAAAATCGAAATGATGTGAATCAATTCGAA-----GTTTAAGAAAtaatattcattGATCAAATGATTCTCTTCATAGTCTGATAGATCCGTGGTGGAActtattaaTCGGCCGAGAATAAAGATAGAGTCCCATTTTAC-ATGTCAATACTGACAACAATGAAATTTATAGTAAGATGXXXGCTCTACT-CCCCAAAAAGGTCTGTTTGACACCTTACCTtttt-ttag-TTATTATCCA-TTTGAATTATTTAGAATCTATATCAGTTTTCATTTTCAAACTTAGAAAGTCTTCTTTTATTTATAAGATCCAAGAAATTCCCGGTCCAAAACTTTTTTAATTTACTACtttt------gagttTCTGTTCATTGACAGAGACCCAAGTCATATATTAAAATGATACTGATacttc--------------------------------------cgtaaTGGTCGGCATAGCTTAATTGCGGAGGACTGAAAATCCTTGTGTCACCATT--------------------------------------------------------------------------------------------AGTAAAATGATAC-TGATACTTCAGTAGATGATACCTCAGTAATGGTGGACATAGC-------TTTTTTG--CAGAGGACTGAAAATC------CTTGTGTCACCATTCGTAAAATGAGGATGATACTTCGGTAATGGCCGGGATAGCTCAgttg

AY257736 AATTGGATTGAGCCTTGGTATGGAAACCTACTAAGTGATAACTTTCAAATTCAGAGAAACCCTGGAATTAAcaatgggcAATCCTGAGCCAAATCCTGGTTTACGTGAACAAACCGGAGTTTAGAAAGCGAGAAAAAA-GGGATAGGTGCAGAGACTCAATGGAAGCTGTTCTAACAAATGGAGTTCACTACCTTGTGTTGATAAAGGAATCCTTCGATCAAAACTTCAAATAAAAAAG-GATGAAGGAGAAAAACCTATATTGTCTAAATATA------GGTAACACAAAA-CGATCGCAAAAATGACGACCTGAATCTCGATTTCTATTTTTTT--ATAAACAAAATCGAAATGATGTGAATCAATTCGAA-----GTTTAAGAAAtaatattcattGATCAAATGATTCTCTTCATAGTCTGATAGATCCGTGGTGGAActtattaaTCGGCCGAGAATAAAGATAGAGTCCCATTTTAC-ATGTCAATACTGACAACAATGAAATTTATAGTAAGATGXXXGCTCTACT-CCCCAAAAAGGTCTGTTTGACACCTTACCTtttt-ttag-TTATTATCCA-TTTGAATTATTTAGAATCTATATCAGTTTTCATTTTCAAACTTAGAAAGTCTTCTTTTATTTATAAGATCCAAGAAATTCCCGGTCCAAAACTTTTTTAATTTACTACtttt------gagttTCTGTTCATTGACAGAGACCCAAGTCATATATTAAAATGATACTGATacttc--------------------------------------cgtaaTGGTCGGCATAGCTTAATTGCGGAGGACTGAAAATCCTTGTGTCACCATT--------------------------------------------------------------------------------------------AGTAAAATGATAC-TGATACTTCAGTAGATGATACCTCAGTAATGGTGGACATAGC-------TTTTTTG--CAGAGGACTGAAAATC------CTTGTGTCACCATTCGTAAAATGAGGATGATACTTCGGTAATGGCCGGGATAGCTCAgttg

AY257737 AATTGGATTGAGCCTTGGTATGGAAACCTACTAAGTGATAACTTTCAAATTCAGAGAAACCCTGGAATTAAcaatgggcAATCCTGAGCCAAATCCTGGTTTACGTGAACAAACCGGAGTTTAGAAAGCGAGAAAAAA-GGGATAGGTGCAGAGACTCAATGGAAGCTGTTCTAACAAATGGAGTTCACTACCTTGTGTTGATAAAGGAATCCTTCGATCAAAACTTCAAATAAAAAAG-GATGAAGGAGAAAAACCTATATTGTCTAAATATA------GGTAACACAAAA-CGATCGCAAAAATGACGACCTGAATCTCGATTTCTATTTTTTT--ATAAACAAAATCGAAATGATGTGAATCAATTCGAA-----GTTTAAGAAAtaatattcattGATCAAATGATTCTCGTCATAGTCTGATAGATCCGTGGTGGAActtattaaTCGGCCGAGAATAAAGATAGAGTCCCATTTTAC-ATGTCAATACTGACAACAATGAAATTTATAGTAAGATGXXXGCTCTACT-CCCCAAAAAGGTCTGTTTGACACCTTACCTtttt-ttag-TTATTATCCA-TTTGAATTATTTAGAATCTATATCAGTTTTCATTTTCAAACTTAGAAAGTCTTCTTTTATTTATAAGATCCAAGAAATTCCCGGTCCAAAACTTTTTTAATTTACTACtttt------gagttTCTGTTCATTGACAGAGACCCAAGTCATATATTAAAATGATACTGATacttc--------------------------------------cgtaaTGGTCGGCATAGCTTAATTGCGGAGGACTGAAAATCCTTGTGTCACCATT--------------------------------------------------------------------------------------------AGTAAAATGATAC-TGATACTTCAGTAGATGATACCTCAGTAATGGTGGACATAGC-------TTTTTTG--CAGAGGACTGAAAATC------CTTGTGTCACCATTCGTAAAATGAGGATGATACTTCGGTAATGGCCGGGATAGCTCAgttg

AY257738 AATTGGATTGAGCCTTGGTATGGAAACCTACTAAGTGATAACTTTCAAATTCAGAGAAACCCTGGAATTAAcaatgggcAATCCTGAGCCAAATCCTGGTTTACGTGAACAAACCGGAGTTTAGAAAGCGAGAAAAAA-GGGATAGGTGCAGAGACTCAATGGAAGCTGTTCTAACAAATGGAGTTCACTACCTTGTGTTGATAAAGGAATCCTTCGATCAAAACTTCAAATAAAAAAG-GATGAAGGAGAAAAACCTATATTGTCTAAATATA------GGTAACACAAAA-CGATCGCAAAAATGACGACCTGAATCTCGATTTCTATTTTTTT--ATAAACAAAATCGAAATGATGTGAATCAATTCGAA-----GTTTAAGAAAtaatattcattGATCAAATGATTCTCTTCATAGTCTGATAGATCCGTGGTGGAActtattaaTCGGCCGAGAATAAAGATAGAGTCCCATTTTAC-ATGTCAATACTGACAACAATGAAATTTATAGTAAGATGXXXNNNNNNNN-NNNNNAAAAGGTCTGTTTGACACCTTACCTtttt-ttag-TTATTATCCA-TTTGAATTATTTAGAATCTATATCAGTTTTCATTTTCAAACTTAGAA-GTCTTCTTTTATTTATAAGATCCAAGAAATTCCCGGTCCAAAACTTTTTTAATTTACTACtttt------gagttTCTGTTCATTGACAGAGACCCAAGTCATATATTAAAATGATACTGATacttc--------------------------------------cgtaaTGGTCGGCATAGCTTAATTGCGGAGGACTGAAAATCCTTGTGTCACCATT--------------------------------------------------------------------------------------------AGTAAAATGATAC-TGATACTTCAGTAGATGATACCTCAGTAATGGTGGACATAGC-------TTTTTTG--CAGAGGACTGAAAATC------CTTGTGTCACCATTCGTAAAATGAGGATGATACTTCGGTAATGGCCGGGATAGCTCAgttg

AY257739 AATTGGATTGAGCCTTGGTATGGAAACCTACTAAGTGATAACTTTCAAATTCAGAGAAACCCTGGAATTAAcaatgggcAATCCTGAGCCAAATCCTGGTTTACGGGAACAAACCGGAGTTTAGAAAGCGAGAAAAAA-GGGATAGGTGCAGAGACTCAATGGAAGCTGTTCTAACAAATGGAGTTCACTACCTTGTGTTGATAAAGGAATCCTTCGATCAAAACTTCAAATAAAAAAG-GATGAAGGAGAAAAACCTATATTGTCTAAATATA------GGTAACACAAAA-CGATCGCAAAAATGACGACCTGAATCTCGATTTCTATTTTTTT--ATAAACAAAATCGAAATGATGTGAATCAATTCGAA-----GTTTAAGAAAtaatattcattGATCAAATGATTCTCTTCATAGTCTGATAGATCCGTGGTGGAActtattaaTCGGCCGAGAATAAAGATAGAGTCCCATTTTAC-ATGTCAATACTGACAACAATGAAATTTATAGTAAGATGXXXGCTCTACT-CCCCAAAAAGGTCTGTTTGACACCTTACCTtttt-ttag-TTATTATCCA-TTTGAATTATTTAGAATCTATATCAGTTTTCATTTTCAAACTTAGAAAGTCTTCTTTTATTTATAAGATCCAAGAAATTCCCGGTCCAAAACTTTTTTAATTTACTACtttt------gagttTCTGTTCATTGACAGAGACCCAAGTCATATATTAAAATGATACTGATacttc--------------------------------------cgtaaTGGTCGGCATAGCTTAATTGCGGAGGACTGAAAATCCTTGTGTCACCATT--------------------------------------------------------------------------------------------AGTAAAATGATAC-TGATACTTCAGTAGATGATACCTCAGTAATGGTGGACATAGC-------TTTTTTG--CAGAGGACTGAAAATC------CTTGTGTCACCATTCGTAAAATGAGGATGATACTTCGGTAATGGCCGGGATAGCTCAgttg

AY257740 AATTGGATTGAGCCTTGGTATGGAAACCTACTAAGTGATAACTTTCAAATTCAGAGAAACCCTGGAATTAAcaatgggcAATCCTGAGCCAAATCCTGGTTTACGTGAACAAACCGGAGTTTAGAAAGCGAGAAAAAA-GGGATAGGTGCAGAGACTCAATGGAAGCTGTTCTAACAAATGGAGTTCACTACCTTGTGTTGATAAAGGAATCCTTCGATCAAAACTTCAAATAAAAAAG-GATGAAGGAGAAAAACCTATATTGTCTAAATATA------GGTAACACAAAA-CGATCGCAAAAATGACGACCTGAATCTCGATTTCTATTTTTTT--ATAAACAAAATCGAAATGATGTGAATCAATTCGAA-----GTTTAAGAAAtaatattcattGATCAAATGATTCTCTTCATAGTCTGATAGATCCGTGGTGGAActtattaaTCGGCCGAGAATAAAGATAGAGTCCCATTTTAC-ATGTCAATACTGACAACAATGAAATTTATAGTAAGATGXXXGCTCTACT-CCCCAAAAAGGTCTGTTTGACACCTTACCTtttt-ttag-TTATTATCCA-TTTGAATTATTTAGAATCTATATCAGTTTTCATTTTCAAACTTAGAAGGTCTTCTTTTATTTATAAGATCCAAGAAATTCCCGGTCCAAAACTTTTTTAATTTACTACtttt------gagttTCTGTTCATTGACAGAGACCCAAGTCATATATTAAAATGATACTGATACTTCCGTACCCAAGTCATATATTAAAATGATACTGATACTTCCGTAATGGTCGGCATAGCTTAATTGCGGAGGACTGAAAATCCTTGTGTCACCATT--------------------------------------------------------------------------------------------AGTAAAATGATAC-TGATACTTCAGTAGATGATACCTCAGTAATGGTGGACATAGC-------TTTTTTG--CAGAGGACTGAAAATC------CTTGTGTCACCATTCGTAAAATGAGGATGATACTTCGGTAATGGCCGGGATAGCTCAgttg

AY257741 AATTGGATTGAGCCTTGGTATGGAAACCTACTAAGTGATAACTTTCAAATTCAGAGAAACCCTGGAATTAAcaatgggcAATCCTGAGCCAAATCCTGGTTTACGTGAACAAACCGGAGTTTAGAAAGCGAGAAAAAA-GGGATAGGTGCAGAGACTCAATGGAAGCTGTTCTAACAAATGGAGTTCACTACCTTGTGTTGATAAAGGAATCCTTCGATCAAAACTTCAAATAAAAAAG-GATGAAGGAGAAAAACCTATATTGTCTAAATATA------GGTAACACAAAA-CGATCGCAAAAATGACGACCTGAATCTCGATTTCTATTTTTTT--ATAAACAAAATCGAAATGATGTGAATCAATTCGAA-----GTTTAAGAAAtaatattcattGATCAAATGATTCTCTTCATAGTCTGATAGATCCGTGGTGGAActtattaaTCGGCCGAGAATAAAGATAGAGTCCCATTTTAC-ATGTCAATACTGACAACAATGAAATTTATAGTAAGATGXXXGCTCTACT-CCCCAAAAAGGTCTGTTTGACACCTTACCTtttt-ttag-TTATTATCCA-TTTGAATTATTTAGAATCTATATCAGTTTTCATTTTCAAACTTAGAAAGTCTTCTTTTATTTATAAGATCCAAGAAATTCCCGGTCCAAAACTTTTTTAATTTACTACtttt------gagttTCTGTTCATTGACAGAGACCCAAGTCATATATTAAAATGATACTGATACTTCCGTACCCAAGTCATATATTAAAATGATACTGATACTTCCGTAATGGTCGGCATAGCTTAATTGCGGAGGACTGAAAATCCTTGTGTCACCATT--------------------------------------------------------------------------------------------AGTAAAATGATAC-TGATACTTCAGTAGATGATACCTCAGTAATGGTGGACATAGC-------TTTTTTG--CAGAGGACTGAAAATC------CTTGTGTCACCATTCGTAAAATGAGGATGATACTTCGGTAATGGCCGGGATAGCTCAgttg

AY257742 AATTGGATTGAGCCTTGGTATGGAAACCTACTAAGTGATAACTTTCAAATTCAGAGAAACCCTGGAATTAAcaatgggcAATCCTGAGCCAAATCCTGGTTTACGTGAACAAACCGGAGTTTAGAAAGCGAGAAAAAA-GGGATAGGTGCAGAGACTCAATGGAAGCTGTTCTAACAAATGGAGTTCACTACCTTGTGTTGATAAAGGAATCCTTCGATCAAAACTTCAAATAAAAAAG-GATGAAGGAGAAAAACCTATATTGTCTAAATATA------GGTAACACAAAA-CGATCGCAAAAATGACGACCTGAATCTCGATTTCTATTTTTTT--ATAAACAAAATCGAAATGATGTGAATCAATTCGAA-----GTTTAAGAAAtaatattcattGATCAAATGATTCTCTTCATAGTCTGATAGATCCGTGGTGGAActtattaaTCGGCCGAGAATAAAGATAGAGTCCCATTTTAC-ATGTCAATACTGACAACAATGAAATTTATAGTAAGATGXXXGCTCTACT-CCCCAAAAAGGTCTGTTTGACACCTTACCTtttt-ttag-TTATTATCCA-TTTGAATTATTTAGAATCTATATCAGTTTTCATTTTCAAACTTAGAAAGTCTTCTTTTATTTATAAGATCCAAGAAATTCCCGGTCCAAAACTTTTTTAATTTACTACtttt------gagttTCTGTTCATTGACAGAGACCCAAGTCATATATTAAAATGATACTGATacttc--------------------------------------cgtaaTGGTCGGCATAGCTTAATTGCGGAGGACTGAAAATCCTTGTGTCACCATTAGTAAAATT-----------------------------------------------------------------------------------AGTAAAATGATAC-TGATACTTCAGTAGATGATACCTCAGTAATGGTGGACATAGC-------TTTTTTG--CAGAGGACTGAAAATC------CTTGTGTCACCATTCGTAAAATGAGGATGATACTTCGGTAATGGCCGGGATAGCTCAgttg

AY257743 AATTGGATTGAGCCTTGGTATGGAAACCTACTAAGTGATAACTTTCAAATTCAGAGAAACCCTGGAATTAAcaatgggcAATCCTGAGCCAAATCCTGGTTTACGTGAACAAACCGGAGTTTAGAAAGCGAGAAAAAA-GGGATAGGTGCAGAGACTCAATGGAAGCTGTTCTAACAAATGGAGTTCACTACCTTGTGTTGATAAAGGAATCCTTCGATCAAAACTTCAAATAAAAAAG-GATGAAGGAGAAAAACCTATATTGTCTAAATATA------GGTAACACAAAA-CGATCGCAAAAATGACGACCTGAATCTCGATTTCTATTTTTTT--ATAAACAAAATCGAAATGATGTGAATCAATTCGAA-----GTTTAAGAAAtaatattcattGATCAAATGATTCTCTTCATAGTCTGATAGATCCGTGGTGGAActtattaaTCGGCCGAGAATAAAGATAGAGTCCCATTTTAC-ATGTCAATACTGACAACAATGAAATTTATAGTAAGATGXXXGCTCTACT-CCCCAAAAAGGTCTGTTTGACACCTTACCTtttt-ttag-TTATTATCCA-TTTGAATTATTTAGAATCTATATCAGTTTTCATTTTCAAACTTAGAAAGTCTTCTTTTATTTATAAGATCCAAGAAATTCCCGGTCCAAAACTTTTTTAATTTACTACtttt------gagttTCTGTTCATTGACAGAGACCCAAGTCATATATTAAAATGATACTGATacttc--------------------------------------cgtaaTGGTCGGCATAGCTTAATTGCGGAGGACTGAAAATCCTTGTGTCACCATTAGTAAAATGATACTGATACTTCAGTAGATGATACCTCAGTAATGGTGGACATAGCTTTTTTGCAGAGGACTGAAAATCCTTGTGTCACCATTAGTAAAATGATAC-TGATACTTCAGTAGATGATACCTCAGTAATGGTGGACATAGC-------TTTTTTG--CAGAGGACTGAAAATC------CTTGTGTCACCATTCGTAAAATGAGGATGATACTTCGGTAATGGCCGGGATAGCTCAgttg

AY257744 AATTGGATTGAGCCTTGGTATGGAAACCTACTAAGTGATAACTTTCAAATTCAGAGAAACCCTGGAATTAAcaatgggcAATCCTGAGCCAAATCCTGGTTTACGTGAACAAACCGGAGTTTAGAAAGCGAGAAAAAA-GGGATAGGTGCAGAGACTCAATGGAAGCTGTTCTAACAAATGGAGTTCACTACCTTGTGTTGATAAAGGAATCCTTCGATCAAAACTTCAAATAAAAAAG-GATGAAGGATAAAAACCTATATTGTCTAAATATA------GGTAACACAAAA-CGATCGCAAAAATGACGACCTGAATCTCGATTTCTATTTTTTT--ATAAACAAAATCGAAATGATGTGAATCAATTCGAA-----GTTTAAGAAAtaatattcattGATCAAATGATTCTCTTCATAGTCTGATAGATCCGTGGTGGAActtattaaTCGGCCGAGAATAAAGATAGAGTCCCATTTTAC-ATGTCAATACTGACAACAATGAAATTTATAGTAAGATGXXXGCTCTACT-CCCCAAAAAGGTCTGTTTGACACCTTACCTtttt-ttag-TTATTATCCA-TTTGAATTATTTAGAATCTATATCAGTTTTCATTTTCAAACTTAGAAAGTCTTCTTTTATTTATAAGATCCAAGAAATTCCCGGTCCAAAACTTTTTTAATTTACTACtttt------gagttTCTGTTCATTGACAGAGACCCAAGTCATATATTAAAATGATACTGATacttc--------------------------------------cgtaaTGGTCGGCATAGCTTAATTGCGGAGGACTGAAAATCCTTGTGTCACCATT--------------------------------------------------------------------------------------------AGTAAAATGATAC-TGATACTTCAGTAGATGATACCTCAGTAATGGTGGACATAGC-------TTTTTTG--CAGAGGACTGAAAATC------CTTGTGTCACCATTAGTAAAATGATACTGATACTTCGGTAATGGCCGGGATAGCTCAgttg

AY257745 AATTGGATTGAGCCTTGGTATGGAAACCTACTAAGTGATAACTTTCAAATTCAGAGAAACCCTGGAATTAAcaatgggcAATCCTGAGCCAAATCCTGGTTTACGTGAACAAACCGGAGTTTAGAAAGCGAGAAAAAA-GGGATAGGTGCAGAGACTCAATGGAAGCTGTTCTAACAAATGGAGTTCACTACCTTGTGTTGATAAAGGAATCCTTCGATCAAAACTTCAAATAAAAAAG-GATGAAGGAGAAAAACCTATATTGTCTAAATATA------GGTAACACAAAA-CGATCGCAAAAATGACGACCTGAATCTCGATTTCTATTTTTTT--ATAAACAAAATCGAAATGATGTGAATCAATTCGAA-----GTTTAAGAAATAATATTTATTGATCAAATGATTCTCTTCATAGTCTGATAGATCCGTGGTGGAActtattaaTCGGCCGAGAATAAAGATAGAGTCCCATTTTAC-ATGTCAATACTGACAACAATGAAATTTATAGTAAGATGXXXGCTCTACT-CCCCAAAAAGGTCTGTTTGACACCTTACCTtttt-ttag-TTATTATCCA-TTTGAATTATTTAGAATCTATATCAGTTTTCATTTTCAAACTTAGAAAGTCTTCTTTTATTTATAAGATCCAAGAAATTCCCGGTCCAAAACTTTTTTAATTTACTACtttt------gagttTCTGTTCATTGACAGAGACCCAAGTCATATATTAAAATGATACTGATacttc--------------------------------------cgtaaTGGTCGGCATAGCTTAATTGCGGAGGACTGAAAATCCTTGTGTCACCATT--------------------------------------------------------------------------------------------AGTAAAATGATAC-TGATACTTCAGTAGATGATACCTCAGTAATGGTGGACATAGC-------TTTTTTG--CAGAGGACTGAAAATC------CTTGTGTCACCATTAGTAAAATGATACTGATACTTCGGTAATGGCCGGGATAGCTCAgttg

AY257746 AATTGGATTGAGCCTTGGTATGGAAACCTACTAAGTGATAACTTTCAAATTCAGAGAAACCCTGGAATTAAcaatgggcAATCCTGAGCCAAATCCTGGTTTACGTGAACAAACCGGAGTTTAGAAAGCGAGAAAAAA-GGGATAGGTGCAGAGACTCAATGGAAGCTGTTCTAACAAATGGAGTTCACTACCTTGTGTTGATAAAGGAATCCTTCGATCAAAACTTCAAATAAAAAAG-GATGAAGGAGAAAAACCTATATTGTCTAAATATA------GGTAACACAAAA-CGATCGCAAAAATGACGACCTGAATCTCGATTTCTATTTTTTT--ATAAACAAAATCGAAATGATGTGAATCAATTCGAA-----GTTTAAGAAAtaatattcattGATCAAATGATTCTCTTCATAGTCTGATAGATCCGTGGTGGAActtattaaTCGGCCGAGAATAAAGATAGAGTCCCATTTTAC-ATGTCAATACTGACAACAATGAAATTTATAGTAAGATGXXXGCTCTACT-CCCCAAAAAGGTCTGTTTGACACCTTACCTtttt-ttag-TTATTATCCA-TTTGAATTATTTAGAATCTATATCAGTTTTCATTTTCAAACTTAGAAAGTCTTCTTTTATTTATAAGATCCAAGAAATTCCCGGTCCAAAACTTTTTTAATTTACTACtttt------gagttTCTGTTCATTGACAGAGACCCAAGTCATATATTAAAATGATACTGATacttc--------------------------------------cgtaaTGGTCGGCATAGCTTAATTGCGGAGGACTGAAAATCCTTGTGTCACCATT--------------------------------------------------------------------------------------------------------------------------------------------------------------------------------------------------------AGTAAAATGATACTGATACTTCGGTAATGGCCGGGATAGCTCAgttg

AY257747 AATTGGATTGAGCCTTGGTATGGAAACCTACTAAGTGATAACTTTCAAATTCAGAGAAACCCTGGAATTAAcaatgggcAATCCTGAGCCAAATCCTGGTTTACGTGAACAAACCGGAGTTTAGAAAGCGAGAAAAAA-GGGATAGGTGCAGAGACTCAATGGAAGCTGTTCTAACAAATGGAGTTCACTACCTTGTGTTGATAAAGGAATCCTTCGATCAAAACTTCAAATAAAAAAG-GATGAAGGAGAAAAACCTATATTGTCTAAATATA------GGTAACACAAAA-CGATCGCAAAAATGACGACCTGAATCTCGATTTCTATTTTTTT--ATAAACAAAATCGAAATGATGTGAATCAATTCGAA-----GTTTAAGAAAtaatattcattGATCAAATGATTCTCTTCATAGTCTGATAGATCCGTGGTGGAActtattaaTCGGCCGAGAATAAAGATAGAGTCCCATTTTAC-ATGTCAATACTGACAACAATGAAATTTATAGTAAGATGXXXGCTCTACT-CCCCAAAAAGGTCTGTTTGACACCTTACCTtttt-ttag-TTATTATCCA-TTTGAATTATTTAGAATCTATATCAGTTTTCATTTTCAAACTTAGAAAGTCTTCTTTTATTTATAAGATCCAAGAAATTCCCGGTCCAAAACTTTTTTAATTTACTACtttt------gagttTCTGTTCATTGACAGAGACCCAAGTCATATATTAAAATGATACTGATacttc--------------------------------------cgtaaTGGTCGGCATAGCTTAATTGCGGAGGACTGAAAATCCTTGTGTCACCATT--------------------------------------------------------------------------------------------------------------------------------------------------------------------------------------------------------CGTAAAATGAGGATGATACTTCGGTAATGGCCGGGATAGCTCAgttg

AY257748 AATTGGATTGAGCCTTGGTATGGAAACCTACTAAGTGATAACTTTCAAATTCAGAGAAACCCTGGAATTAAcaatgggcAATCCTGAGCCAAATCCTGGTTTACGTGAACAAACCGGAGTTTAGAAAGCGAGAAAAAA-GGGATAGGTGCAGAGACTCAATGGAAGCTGTTCTAACAAATGGAGTTCACTACCTTGTGTTGATAAAGGAATCCTTCGATCAAAACTTCAAATAAAAAAG-GATGAAGGAGAAAAACCTATATTGTCTAAATATA------GGTAACACAAAA-CGATCGCAAAAATGACGACCTGAATCTCGATTTCTATTTTTTT--ATAAACAAAATCGAAATGATGTGAATCAATTCGAA-----GTTTAAGAAAtaatattcattGATCAAATGATTCTCTTCATAGTCTGATAGATCCGTGGTGGAActtattaaTCGGCCGAGAATAAAGATAGAGTCCCATTTTAC-ATGTCAATACTGACAACAATGAAATTTATAGTAAGATGXXXGCTCTACT-CCCCAAAAAGGTCTGTTTGACACCTTACCTtttt-ttag-TTATTATCCA-TTTGAATTATTTAGAATCTATATCAGTTTTCATTTTCAAACTTAGAAAGTCTTCTTTTATTTATAAGATCCAAGAAATTCCCGGTCCAAAACTTTTTTAATTTACTACtttt------gagttTCTGTTCATTGACAGAGACCCAAGTCATATATTAAAATGATACTGATACTTC----------------------------------------------------------------------------------------------------------------------------------------------------------------------------------------------------------------CGTAGATGATACCTCAGTAATGGTGGACATAGC-------TTTTTTG--CAGAGGACTGAAAATC------CTTGTGTCACCATTCGTAAAATGAGGATGATACTTCGGTAATGGCCGGGATAGCTCAgttg

AY257749 AATTGGATTGAGCCTTGGTATGGAAACCTACTAAGTGATAACTTTCAAATTCAGAGAAACCCTGGAATTAAcaatgggcAATCCTGAGCCAAATCCTGGTTTACGTGAACAAACCGGAGTTTAGAAAGCGAGAAAAAA-GGGATAGGTGCAGAGACTCAATGGAAGCTGTTCTAACAAATGGAGTTCACTACCTTGTGTTGATAAAGGAATCCTTCGATCAAAACTTCAAATAAAAAAG-GATGAAGGAGAAAAACCTATATTGTCTAAATATA------GGTAACACAAAA-CGATCGCAAAAATGACGACCTGAATCTCGATTTCTATTTTTTT--ATAAACAAAATCGAAATGATGTTAATCAATTCGAA-----GTTTAAGAAAtaatattcattGATCAAATGATTCTCTTCATAGTCTGATAGATCCGTGGTGGAActtattaaTCGGCCGAGAATAAAGATAGAGTCCCATTTTAC-ATGTCAATACTGACAACAATGAAATTTATAGTAAGATGXXXGCTCTACT-CCCCAAAAAGGTCTGTTTGACACCTTACCTtttt-ttag-TTATTATCCA-TTTGAATTATTTAGAATCTATATCAGTTTTCATTTTCAAACTTAGAAAGTCTTCTTTTATTTATAAGATCCAAGAAATTCCCGGTCCAAAACTTTTTTAATTTACTACtttt------gagttTCTGTTCATTGACAGAGACCCAAGTCATATATTAAAATGATACTGATacttc--------------------------------------cgtaaTGGTCGGCATAGCTTAATTGCGGAGGACTGAAAATCCTTGTGTCACCATT--------------------------------------------------------------------------------------------AGTAAAATGATAC-TGATACTTCAGTAGATGATACCTCAGTAATGGTGGACATAGC-------TTTTTTG--CAGAGGACTGAAAATC------CTTGTGTCACCATTCGTAAAATGAGGATGATACTTCGGTAATGGCCGGGATAGCTCAgttg

AY257750 AATTGGATTGAGCCTTGGTATGGAAACCTACTAAGTGATAACTTTCAAATTCAGAGAAACCCTGGAATTAAcaatgggcAATCCTGAGCCAAATCCTGGTTTACGTGAACAAACCGGAGTTTAGAAAGCGAGAAAAAA-GGGATAGGTGCAGAGACTCAATGGAAGCTGTTCTAACAAATGGAGTTCACTACCTTGTGTTGATAAAGGAATCCTTCGATCAAAACTTCAAATAAAAAAG-GATGAAGGAGAAAAACCTATATTGTCTAAATATA------GGTAACACAAAA-CGATCGCAAAAATGACGACCTGAATCTCGATTTCTATTTTTTT--ATAAACAAAATCGAAATGATGTTAATCAATTCGAA-----GTTTAAGAAAtaatattcattGATCAAATGATTCTCTTCATAGTCTGATAGATCCGTGGTGGAActtattaaTCGGCCGAGAATAAAGATAGAGTCCCATTTTAC-ATGTCAATACTGACAACAATGAAATTTATAGTAAGATGXXXGCTCTACT-CCCCAAAAAGGTCTGTTTGACACCTTACCTtttt-ttag-TTATTATCCA-TTTGAATTATTTAGAATCTATATCAGTTTTCATTTTCAAACTTAGAAAGTCTTCTTTTATTTATAAGATCCAAGAAATTCCCGGTCCAAAACTTTTTTAATTTACTACtttt------gagttTCTGTTCATTGACAGAGACCCAAGTCATATATTAAAATGATACTGATacttc--------------------------------------cgtaaTGGTCGGCATAGCTTAATTGCGGAGGACTGAAAATCCTTGTGTCAACATT--------------------------------------------------------------------------------------------AGTAAAATGATAC-TGATACTTCAGTAGATGATACCTCAGTAATGGTGGACATAGC-------TTTTTTG--CAGAGGACTGAAAATC------CTTGTGTCACCATTCGTAAAATGAGGATGATACTTCGGTAATGGCCGGGATAGCTCAgttg

AY257751 AATTGGATTGAGCCTTGGTATGGAAACCTACTAAGTGATAACTTTCAAATTCAGAGAAACCCTGGAATTAAcaatgggcAATCCTGAGCCAAATCCTGGTTTACGTGAACAAACCGGAGTTTAGAAAGCGAGAAAAAA-GGGATAGGTGCAGAGACTCAATGGAAGCTGTTCTAACAAATGGAGTTCACTACCTTGTGTTGATAAAGGAATCCTTCGATCAAAACTTCAAATAAAAAAG-GATGAAGGAGAAAAACCTATATTGTCTAAATATA------GGTAACACAAAA-CGATCGCAAAAATGACGACCTGAATCTCGATTTCTATTTTTTT--ATAAACAAAATCGAAATGATGTGAATCAATTCGAA-----GTTTAAGAAAtaatattcattGATCAAATGATTCTCTTCATAGTCTGATAGATCCGTGGTGTAActtattaaTCGGCCGAGAATAAAGATAGAGTCCCATTTTAC-ATGTCAATACTGACAACAATGAAATTTATAGTAAGATGXXXGCTCTACT-CCCCAAAAAGGTCTGTTTGACACCTTACCTtttt-ttag-TTATTATCCA-TTTGAATTATTTAGAATCTATATCAGTTTTCATTTTCAAACTTAGAAAGTCTTCTTTTATTTATAAGATCCAAGAAATTCCCGGTCCAAAACTTTTTTAATTTACTACtttt------gagttTCTGTTCATTGACAGAGACCCAAGTCATATATTAAAATGATACTGATacttc--------------------------------------cgtaaTGGTCGGCATAGCTTAATTGCGGAGGACTGAAAATCCTTGTGTCACCATT--------------------------------------------------------------------------------------------AGTAAAATGATAC-TGATACTTCAGTAGATGATACCTCAGTAATGGTGGACATAGC-------TTTTTTG--CAGAGGACTGAAAATC------CTTGTGTCACCATTCGTAAAATGAGGATGATACTTCGGTAATGGCCGGGATAGCTCAgttg

AY257752 AATTGGATTGAGCCTTGGTATGGAAACCTACTAAGTGATAACTTTCAAATTCAGAGAAACCCTGGAATTAAcaatgggcAATCCTGAGCCAAATCCTGGTTTACGTGAACAAACCGGAGTTTAGAAAGCGAGAAAAAA-GGGATAGGTGCAGAGACTCAATGGAAGCTGTTCTAACAAATGGAGTTCACTACCTTGTGTTGATAAAGGAATCCTTCGATCAAAACTTCAAATAAAAAAG-GATGAAGGAGAAAAACCTATATTGTCTAAATATA------GGTAACACAAAA-CGATCGCAAAAATGACGACCTGAATCTCGATTTCTATTTTTTT--ATAAACAAAATCGAAATGATGTGAATCAATTCGAA-----GTTTAAGAAAtaatattcattGATCAAATGATTCTCTTCATAGTCTGATAGATCCGTGGTGTAActtattaaTCGGCCGAGAATAAAGATAGAGTCCCATTTTAC-ATGTCAATACTGACAACAATGAAATTTATAGTAAGATGXXXGCTCTACT-CCCCAAAAAGGTCTGTTTGACACCTTACCTtttt-ttag-TTATTATCCA-TTTGAATTATTTAGAATTTATATCAGTTTTCATTTTCAAACTTAGAAAGTCTTCTTTTATTTATAAGATCCAAGAAATTCCCGGTCCAAAACTTTTTTAATTTACTACtttt------gagttTCTGTTCATTGACAGAGACCCAAGTCATATATTAAAATGATACTGATacttc--------------------------------------cgtaaTGGTCGGCATAGCTTAATTGCGGAGGACTGAAAATCCTTGTGTCACCATT--------------------------------------------------------------------------------------------AGTAAAATGATAC-TGATACTTCAGTAGATGATACCTCAGTAATGGTGGACATAGC-------TTTTTTG--CAGAGGACTGAAAATC------CTTGTGTCACCATTCGTAAAATGAGGATGATACTTCGGTAATGGCCGGGATAGCTCAgttg

AY257753 AATTGGATTGAGCCTTGGTATGGAAACCTACTAAGTGATAACTTTCAAATTCAGAGAAACCCTGGAATTAAcaatgggcAATCCTGAGCCAAATCCTGGTTTACGTGAACAAACCGGAGTTTAGAAAGCGAGAAAAAA-GGGATAGGTGCAGAGACTCAATGGAAGCTGTTCTAACAAATGGAGTTCACTACCTTGTGTTGATAAAGGAATCCTTCGATCAAAACTTCAAATCAAAAAG-GATGAAGGAGAAAAACCTATATTGTCTAAATATA------GGTAACACAAAA-CGATCGCAAAAATGACGACCTGAATCTCGATTTCTATTTTTTT--ATAAACAAAATCGAAATGATGTGAATCAATTCGAA-----GTTTAAGAAAtaatattcattGATCAAATGATTCTCTTCATAGTCTGATAGATCCGTGGTGGAActtattaaTCGGCCGAGAATAAAGATAGAGTCCCATTTTAC-ATGTCAATACTGACAACAATGAAATTTATAGTAAGATGXXXGCTCTACT-CCCCAAAAAGGTCTGTTTGACACCTTACCTtttt-ttag-TTATTATCCA-T----------------------------------------------------------------------------------------AACTTTTTTAATTTACTACtttt------gagttTCTGTTCATTGACAGAGACCCAAGTCATATATTAAAATGATACTGATacttc--------------------------------------cgtaaTGGTCGGCATAGCTTAATTGCGGAGGACTGAAAATCCTTGTGTCACCATT--------------------------------------------------------------------------------------------AGTAAAATGATAC-TGATACTTCAGTAGATGATACCTCAGTAATGGTGGACATAGC-------TTTTTTG--CAGAGGACTGAAAATC------CTTGTGTCACCATTCGTAAAATGAGGATGATACTTCGGTAATGGCCGGGATAGCTCAgttg

AY257754 AATTGGATTGAGCCTTGGTATGGAAACCTACTAAGTGATAACTTTCAAATTCAGAGAAACCCTGGAATTAAcaatgggcAATCCTGAGCCAAATCCTGGTTTACGTGAACAAACCGGAGTTTAGAAAGCGAGAAAAAA-GGGATAGGTGCAGAGACTCAATGGAAGCTGTTCTAACAAATGGAGTTCACTACCTTGTGTTGATAAAGGAATCCTTCGATCAAAACTTCAAATCAAAAAG-GATGAAGGAGAAAAACCTATATTATCTAAATATA------GGTAACACAAAA-CGATCGCAAAAATGACGACCTGAATCTCGATTTCTATTTTTTT--ATAAACAAAATCGAAATGATGTGAATCAATTCGAA-----GTTTAAGAAAtaatattcattGATCAAATGATTCTCTTCATAGTCTGATAGATCCGTGGTGGAActtattaaTCGGCCGAGAATAAAGATAGAGTCCCATTTTAC-ATGTCAATACTGACAACAATGAAATTTATAGTAAGATGXXXGCTCTACT-CCCCAAAAAGGTCTGTTTGACACCTTACCTtttt-ttag-TTATTATCCA-T----------------------------------------------------------------------------------------AACTTTTTTAATTTACTACtttt------gagttTCTGTTCATTGACAGAGACCCAAGTCATATATTAAAATGATACTGATacttc--------------------------------------cgtaaTGGTCGGCATAGCTTAATTGCGGAGGACTGAAAATCCTTGTGTCACCATT--------------------------------------------------------------------------------------------AGTAAAATGATAC-TGATACTTCAGTAGATGATACCTCAGTAATGGTGGACATAGC-------TTTTTTG--CAGAGGACTGAAAATC------CTTGTGTCACCATTCGTAAAATGAGGATGATACTTCGGTAATGGCCGGGATAGCTCAgttg

AY257755 AATTGGATTGAGCCTTGGTATGGAAACCTACTAAGTGATAACTTTCAAATTCAGAGAAACCCTGGAATTAAcaatgggcAATCCTGAGCCAAATCCTGGTTTACGTGAACAAACCGGAGTTTAGAAAGCGAGAAAAAA-GGGATAGGTGCAGAGACTCAATGGAAGCTGTTCTAACAAATGGAGTTCACTACCTTGTGTTGATAAAGGAATCCTTCGATCAAAACTTCAAATCAAAAAG-GATGAAGGAGAAAAACCTATATTGTCTAAATATA------GGTAACACAAAA-CGATCGCAAAAATGACGACCTGAATCTCGATTTCTATTTTTTT--ATAAACAAAATCGAAATGATGTGAATCAATTCGAA-----GTTTAAGAAAtaatattcattGATCAAATGATTCTCTTCATAGTCTGATAGATCCGTGGTGGAActtattaaTCGGCCGAGAATAAAGATAGAGTCCCATTTTAC-ATGTCAATACTGACAACAATGAAATTTATAGTAAGATGXXXGCTCTACT-CCCCAAAAAGGTCTATTTGACACCTTACCTtttt-ttag-TTATTATCCA-T----------------------------------------------------------------------------------------AACTTTTTTAATTTACTACtttt------gagttTCTGTTCATTGACAGAGACCCAAGTCATATATTAAAATGATACTGATacttc--------------------------------------cgtaaTGGTCGGCATAGCTTAATTGCGGAGGACTGAAAATCCTTGTGTCACCATT--------------------------------------------------------------------------------------------AGTAAAATGATAC-TGATACTTCAGTAGATGATACCTCAGTAATGGTGGACATAGC-------TTTTTTG--CAGAGGACTGAAAATC------CTTGTGTCACCATTCGTAAAATGAGGATGATACTTCGGTAATGGCCGGGATAGCTCAgttg

AY257759 AATTGGATTGAGCCTTGGTATGGAAACCTACTAAGTGATAACTTTCAAATTCAGAGAAACCCTGGAATTAAcaatgggcAATCCTGAGCCAAATCCTAGTTTACGTGAACAAACCGGAGTTTAGAAAGCGAGAAAAAA-GGGATAGGTGCAGAGACTCAATGGAAGCTGTTCTAACAAATGGAGTTCACTACCTTGTGTTGATAAAGGAATCCTTCGATCAAAACTTCAAATCAAAAAG-GTTGAAGGAGAAAAACCTATATTGTCTAAATATA------GGTAACACAAAA-CGATCTCAAAAATGACGACCTGAATCTCGATTTCTATTTTTTT--ATAAACAAAATCGAAATGATGTGAATCAATTCGAA-----GTTTAAGAAAtaatattcattGATCAAATGATTCTCTTCATAGTCTGATAGATCCGTGGTGGAActtattaaTCGGCCGAGAATAAAGATAGAGTCCCATTTTAC-ATGTCAATACTGACAACAATGAAATTTATAGTAAGATGXXXGCTCTACT-CCCCAAAAAGGTCTGTTTGACACCTTACCTtttt-ttag-TTATTATCCA-TTTGAATTATTTAGAATCTATATCAGTTTTCATTTTCAAACTTAGAAAGTCTTCTTTTATTTATAAGATCCAAGAAATTCCCGGTCCAAAACTTTTTTAATTTACTACtttt------gagttTCTTTTCATTGACAGAGACCCAAGTCATATATTAAAATGATACTGATacttc--------------------------------------cgtaaTGGTCGGCATAGCTTAATTGCGGAGGACTGAAAATCCTTGTGTCACCATT--------------------------------------------------------------------------------------------AGTAAAA-------TGATACTTCAGTAGATGATACCTCAGTAATGGTGGACATAGC-------TTTTTTG--CAGAGGACTGAAAATC------CTTGTGTCACCATTCGTAAAATGAGGATGATACTTCGGTAATGGCCGGGATAGCTCAgttg

DQ013047 AATTGGATTGAGCCTTGGTATGGAAACCTACTAAGTGATAACTTTCAAATTCAGAGAAACCCTGGAATTAAcaatgggcAATCCTGAGCCAAATCCTGGTTTACGTGAACAAACCGGAGTTTAGAAAGCGAGAAAAAA-GGGATAGGTGCAGAGACTCAATGGAAGCTGTTCTAACAAATGGAGTTCACTACCTTGTGTTGATAAAGGAATCCTTCGATCAAAACTTCAAATCAAAAAG-GATGAAGGAGAAAAACCTATATTGTCTAAATATA------GGTAACACAAAA-CGATCGCAAAAATGACGACCTGAATCTCGATTTCTATTTTTTT--ATAAACAAAATCGAAATGATGTGAATCAATTCGAA-----GTTTAAGAAAtaatattcattGATCAAATGATTCTCTTCATAGTCTGATAGATCCGTGGTGGAActtattaaTCGGCCGAGAATAAAGATAGAGTCCCATTTTAC-ATGTCAATACTGACAACAATGAAATTTATAGTAAGATGXXXGCTCTACT-CCCCAAAAAGGTCTGTTTGACACCTTACCTTTTT-KTAG-TTATTATCCA-TTTGAATTATTTAGAATCTATATCAGTTTTCATTTTCAAACTTAGAAAGTCTTCTTTTATTTATAAGATCCAARAAATTCCCGGTCCAAAACTTTTTTAATTTACTACtttt------gagttTCTKTTCATTGACAGAGACCCAAGTCATATATTAAAATGATACTGATacttc--------------------------------------cgtaaTGGTCGGCATAGCTTAATTGCGGAGGACTGAAAATCCTTGTGTCACCATT--------------------------------------------------------------------------------------------AGTAAAATGATAM-TGATACTTCAGTAGATGATACCTCAGTAATGGTGGACATAGC-------TTTTTTG--CAGAGGACTGAAAATC------CTTGTGTCACCATTCGTAAAATGAGGATGATACTTCGGTAATGGCCGGGATAGCTCAgttg

DQ013048 AATTGGATTGAGCCTTGGTATGGAAACCTACTAAGTGATAACTTTCAAATTCAGAGAAACCCTGGAATTAAcaatgggcAATCCTGAGCCAAATCCTGGTTTACGTGAACAAACCGGAGTTTAGAAAGCGAGAAAAAA-GGGATAGGTGCAGAGACTCAATGGAAGCTGTTCTAACAAATGGAGTTCACTACCTTGTGTTGATAAAGGAATCCTTCGATCAAA----------AAAAAG-GATGAAGGAGAAAAACCTATATTGTCTAAATATA------GGTAACACAAAA-CGATCGCAAAAATGACGACCTGAATCTCGATTTCTATTTTTTT--ATAAACAAAATCGAAATGATGTGAATCAATTCGAA-----GTTTAAGAAAtaatattcattGATCAAATGATTCTCTTCATAGTCTGATAGATCCGTGGTGGAActtattaaTCGGCCGAGAATAAAGATAGAGTCCCATTTTAC-ATGTCAATACTGACAACAATGAAATTTATAGTAAGATGXXXGCTCTACT-CCCCAAAAAGGTCTGTTTGACACCTTACCTtttt-ttag-TTATTATCCA-TTTGAATTATTTAGAATCTATATCAGTTTTCATTTTCAAACTTAGAAAGTCTTCTTTTATTTATAAGATCCAAGAAATTCCCGGTCCAAAACTTTTTTAATTTACTACtttt------gagttTCTGTTCATTGACAGAGACCCAAGTCATATATTAAAATGATACTGATacttc--------------------------------------cgtaaTGGTCGGCATAGCTTAATTGCGGAGGACTGAAAATCCTTGTGTCACCATT--------------------------------------------------------------------------------------------AGTAAAATGATAC-TGATACTTCAGTAGATGATACCTCAGTAATGGTGGACATAGC-------TTTTTTG--CAGAGGACTGAAAATC------CTTGTGTCACCATTCGTAAAATGAGGATGATACTTCGGTAATGGCCGGGATAGCTCAgttg

DQ013049 AATTGGATTGAGCCTTGGTATGGAAACCTACTAAGTGATAACTTTCAAATTCAGAGAAACCCTGGAATTAAcaatgggcAATCCTGAGCCAAATCCTGGTTTACGTGAACAAACCGGAGTTTAGAAAGCGAGAAAAAA-GGGATAGGTGCAGAGACTCAATGGAAGCTGTTCTAACAAATGGAGTTCACTACCTTGTGTTGATAAAGGAATCCTTCGATCAAAACTTCGAATAAAAAAG-GATGAAGGAGAAAAACCTATATTGTCTAAATATA------GGTAACACAAAA-CGATCGCAAAAATGACGACCTGAATCTCGATTTCTATTTTTTT--ATAAACAAAATCGAAATGATGTTAATCAATTCGAA-----GTTTAAGAAAtaatattcattGATCAAATGATTCTCTTCATAGTCTGATAGATCCGTGGTGGAActtattaaTCGGCCGAGAATAAAGATAGAGTCCCATTTTAC-ATGTCAATACTGACAACAATGAAATTTATAGTAAGATGXXXGCTCTACT-CCCCAAAAAGGTCTGTTTGACACCTTACCTtttt-ttag-TTATTATCCA-TTTGAATTATTTAGAATCTATATCAGTTTTCATTTTCAAACTTAGAAAGTCTTCTTTTATTTATAAGATCCAAGAAATTCCCGGTCCAAAACTTTTTTAATTTACTACtttt------gagttTCTGTTCATTGACAGAGACCCAAGTCATATATTAAAATGATACTGATacttc--------------------------------------cgtaaTGGTCGGCATAGCTTAATTGCGGAGGACTGAAAATCCTTGTGTCACCATT--------------------------------------------------------------------------------------------AGTAAAATGATAC-TGATACTTCAGTAGATGATACCTCAGTAATGGTGGACATAGC-------TTTTTTG--CAGAGGACTGAAAATC------CTTGTGTCACCATTCGTAAAATGAGGATGATACTTCGGTAATGGCCGGGATAGCTCAgttg

DQ013050 AATTGGATTGAGCCTTGGTATGGAAACCTACTAAGTGATAACTTTCAAATTCAGAGAAACCCTGGAATTAAcaatgggcAATCCTGAGCCAAATCCTGGTTTACGTGAACAAACCGGAGTTTAGAAAGCGAGAAAAAA-GGGATAGGTGCAGAGACTCAATGGAAGCTGTTCTAACAAATGGAGTTCACTACCTTGTGTTGATAAAGGAATCCTTCGATCAAAACTTCAAATAAAAAAG-GATGAAGGAGAAAAACCTATATTGTCTAAAGATA------GGTAACACAAAA-CGATCGCAAAAATGACGACCTGAATCTCGATTTCTATTTTTTT--ATAAACAAAATCGAAATGATGTTAATCAATTCGAA-----GTTTAAGAAAtaatattcattGATCAAATGATTCTCTTCATAGTCTGATAGATCCGTGGTGGAActtattaaTCGGCCGAGAATAAAGATAGAGTCCCATTTTAC-ATGTCAATACTGACAACAATGAAATTTATAGTAAGATGXXXGCTCTACT-CCCCAAAAAGGTCTGTTTGACACCTTACCTtttt-ttag-TTATTATCCA-TTTGAATTATTTAGAATCTATATCAGTTTTCATTTTCAAACTTAGAAAGTCTTCTTTTATTTATAAGATCCAAGAAATTCCCGGTCCAAAACTTTTTTAATTTACTACtttt------gagttTCTGTTCATTGACAGAGACCCAAGTCATATATTAAAATGATACTGATacttc--------------------------------------cgtaaTGGTCGGCATAGCTTAATTGCGGAGGACTGAAAATCCTTGTGTCACCATT--------------------------------------------------------------------------------------------AGTAAAATGATAC-TGATACTTCAGTAGATGATACCTCAGTAATGGTGGACATAGC-------TTTTTTG--CAGAGGACTGAAAATC------CTTGTGTCACCATTCGTAAAATGAGGATGATACTTCGGTAATGGCCGGGATAGCTCAgttg

DQ013051 AATTGGATTGAGCCTTGGTATGGAAACCTACTAAGTGATAACTTTCAAATTCAGAGAAACCCTGGAATTAAcaatgggcAATCCTGAGCCAAATCCTGGTTTACGTGAACAAACCGGAGTTTAGAAAGCGAGAAAAAA-GGGATAGGTGCAGAGACTCAATGGAAGCTGTTCTAACAAATGGAGTTCACTACCTTGTGTTGATAAAGGAATCCTTCGATCAAAACTTCAAATCAAAAAG-GATGAAGGAGAAAAACCTATATTGTCTAAATATA------GGTAACACAAAA-CGATCGCAAAAATGACGACCTGAATCTCGATTTCTATTTTTTT--ATAAACAAAATCGAAATGATGTGAATCAATTCGAA-----GTTTAAGAAAtaatattcattGATCAAATGATTCTCTTCATAGTCTGATAGATCCGTGGTGGAActtattaaTCGGCCGAGAATAAAGATAGAGTCCCATTTTAC-ATGTCAATACTGACAACAATGAAATTTATAGTAAGATGXXXGCTCTACT-CCCCAAAAAGGTCTGTTTGACACCTTACCTtttt-ttag-TTATTATCCA-TTTGAATTATTTAGAATCTATATCAGTTTTCATTTTCAAACTTAGAAAGTCTTCTTTTATTTATAAGATCCAAGAAATTCCCGGTCCAAAACTTTTTTAATTTACTACtttt------gagttTCTGTTCATTGACAGAGACCCAAGTCATATATTAAAATGATACTGATacttc--------------------------------------cgtaaTGGTCGGCATAGCTTAATTGCGGAGGACTGAAAATCCTTGTTT-ACCATT--------------------------------------------------------------------------------------------AGTAAAATGATAC-TGATACTTCAGTAGATGATACCTCAGTAATGGTGGACATAGCG------TTTTTTG--CAGAGGACTGAAAATC------CTTGTGTCACCATTCGTAAAATGAGGATGATACTTCGGTAATGGCCGGGATAGCTCAgttg

trnLF_DV AATTGGATTGAGCCTTNGTATGGAAACCTACTAAGTGATAACTTTCAAATTCAGAGAAACCCTGGAATTAAcaatgggcAATCCTGAGCCAAATCCTGGTTTACGTGAACAAACCGGAGTTTAGAAAGCGAGAAAAAA-GGGATAGGTGCAGAGACTCAATGGAAGCTGTTCTAACAAATGGAGTTAACTACCTTGTGTTGATAAAGGAATCCTTCGATCAAAACTTCAAATCAAAAAG-GATGAAGGAGAAAAACCTATATTGTCTAAATATA------GGTAACACAAAA-CGATCGAAAAAATGACGACCTGAATCTCGATTTCTATTTTTTT--ATAAACAAAATCGAAATGATGTGAATCAATTCGAA-----GTTTAAGAAAtaatattcattGATCAAATGATTCTCTTCATAGTCTGATAGATCCGTGGTGGAActtattaaTCGGCCGAGAATAAAGATAGAGTCCCATTTTAC-ATGTCAATACTGACAACAATGAAATTTATAGTAAGATGXXXGCTCTACT-CCCCAAAAAGGTCTGTTTGACACCTTACCTtttt-ttag-TTATTATCCA-TTTGAATTATTTAGAATCTATATCAGTTTTCATTTTCAAACTTAGAAAGTCTTCTTTTATTTATAAGATCCAAGAAATTCCCGGTCCAAAACTTTTTTAATTTACTACtttt------gagttTCTGTTCATTGACAGAGACCCAAGTCATATATTAAAATGATACTGATacttc--------------------------------------cgtaaTGGTCGGCATAGCTTAATTGCGGAGGACTGAAAATCCTTGTGTCACCATT--------------------------------------------------------------------------------------------AGTAAAATGATAC-TGATACTTCAGTAGATGATACCTCAGTAATGGTGGACATAGC-------TTTTTTG--CAGAGGACTGAAAATC------CTTGTGTCACCATTCGTAAAATGAGGATGATACTTCGGTAATGGCCGGGATAGCTCAgttg

trnLF_FH AATTGGATTGAGCCTTGGTATGGAAACCTACTAAGTGATAACTTTCAAATTCAGAGAAACCCTGGAATTAAcaatgggcAATCCTGAGCCAAATCCTGGTTTACGTGAACAAACCGGAGTTTAGAAAGCGAGAAAAAA-GGGATAGGTGCAGAGACTCAATGGAAGCTGTTCTAACAAATGGAGTTCACTACCTTGTGTTGATAAAGGAATCCTTCGATCAAAACTTCAAATCAAAAAG-GATGAAGGAGAAAAACCTATATTGTCTAAATATA------GGTAACACAAAA-CGATCGCAAAAATGACGACCTGAATCTCGATTTCTATTTTTTT--ATAAACAAAATCGAAATGATGTGAATCAATTCGAA-----GTTTAAGAAAtaatattcattGATCAAATGATTCTCTTCATAGTCTGATAGATCCGTGGTGGAActtattaaTCGGCCGAGAATAAAGATAGAGTCCCATTTTAC-ATGTCAATACTGACAACAATGAAATTTATAGTAAGATGXXXGCTCTACT-CCCCAAAAAGGTCTGTTTGACACCTTACCTtttt-ttagCTTATTATCCA-TTTGAATTATTTAGAATCTATATCAGTTTTCATTTTCAAACTTAGAAAGTCTTCTTTTATTTATAAGATCCAAGAAATTCCCGGTCCAAAACTTTTTTAATTTACTACtttt------gagttTCTGTTCATTGACAGAGACCCAAGTCATATATTAAAATGATACTGATacttc--------------------------------------cgtaaTGGTCGGCATAGCTTAATTGCGGAGGACTGAAAATCCTTGTGTCACCATT--------------------------------------------------------------------------------------------AGTAAAATGATAC-TGATACTTCAGTAGATGATACCTCAGTAATGGTGGACATAGC-------TTTTTTG--CAGAGGACTGAAAATC------CTTGTGTCACCATTCGTAAAATGAGGATGATACTTCGGTAATGGCCGGGATAGCTCAgttg

trnLF_FT AATTGGATTGAGCCTTGGTATGGAAACCTACTAAGTGATAACTTTCAAATTCAGAGAAACCCTGGAATTAAcaatgggcAATCCTGAGCCAAATCCTGGTTTACGTGAACAAACCGGAGTTTAGAAAGCGAGAAAAAA-GGGATAGGTGCAGAGACTCAATGGAAGCTGTTCTAACAAATGGAGTTCACTACCTTGTGTTGATAAAGGAATCCTTCGATCAAAACTTCAAATAAAAAAG-GATGAAGGAGAAAAACCTATATTGTCTAAATATA------GGTAACACAAAA-CGATCGCAAAAATGACGACCTGAATCTCGATTTCTATTTTTTT--ATAAACAAAATCGAAATGATGTGAATCAATTCGAA-----GTTTAAGAAAtaatattcattGATCAAATGATTCTCTTCATAGTCTGATAGATCCGTGGTGGAActtattaaTCGGCCGAGAATAAAGATAGAGTCCCATTTTAC-ATGTCAATACTGACAACAATGAAATTTATAGTAAGATGXXXGCTCTACT-CCCCAAAAAGGTCTGTTTGACACCTTACCTtttt-ttag-TTATTATCCA-TTTGAATTATTTAGAATCTATATCAGTTTTCATTTTCAAACTTAGAAAGTCTTCTTTTATTTATAAGATCCAAGAAATTCCCGGTCCAAAACTTTTTTAATTTACTACtttt------gagttTCTGTTCATTGACAGAGACCCAAGTCATATATTAAAATGATACTGATacttc--------------------------------------cgtaaTGGTCGGCATAGCTTAATTGCGGAGGACTGAAAATCCTTGTGTCACCATT--------------------------------------------------------------------------------------------AGTAAAATGATAC-TGATACTTCAGTAGATGATACCTCAGTAATGGTGGACATAGC-------TTTTTTG--CAGAGGACTGAAAATC------CTTGTGTCACCATTAGTAAAATGATACTGATACTTCGGTAATGGCCGGGATAGCTCAgttg

trnLF_HY AATTGGATTGAGCCTTGGTATGGAAACCTACTAAGTGATAACTTTCAAATTCAGAGAAACCCTGGAATTAAcaatgggcAATCCTGAGCCAAATCCTGGTTTACGTGAACAAACCGGAGTTTAGAAAGCGAGAAAAAA-GGGATAGGTGCAGAGACTCAATGGAAGCTGTTCTAACAAATGGAGTTCACTACCTTGTGTTGATAAAGGAATCCTTCGATCAAAACTTCAAATAAAAAAG-GATGAAGGAGAAAAACCTATATTGTCTAAATATA------GGTAACACAAAA-CGATCGCAAAAATGACGACCTGAATCTCGATTTCTATTTTTTT--ATAAACAAAATCGAAATGATGTGAATCAATTCGAA-----GTTTAAGAAAtaatattcattGATCAAATGATTCTCTTCATAGTCTGATAGATCCGTGGTGGACcttattaaTCGGCCGAGAATAAAGATAGAGTCCCATTTTAC-ATGTCAATACTGACAACAATGAAATTTATAGTAAGATGXXXNNNNNNNN-NNCCAAAAAGGTCTGTTTGACACCTTACCTtttt-ttag-TTATTATCCA-TTTGAATTATTTAGAATCTATATCAGTTTTCATTTTCAAACTTAGAAAGTCTTCTTTTATTTATAAGATCCAAGAAATTCCCGGTCCAAAACTTTTTTAATTTACTACtttt------gagttTCTGTTCATTGACAGAGACCCAAGTCATATATTAAAATGATACTGATacttc--------------------------------------cgtaaTGGTCGGCATAGCTTAATTGCGGAGGACTGAAAATCCTTGTGTCACCATT--------------------------------------------------------------------------------------------AGTAAAATGATAC-TGATACTTCAGTAGATGATACCTCAGTAATGGTGGACATAGC-------TTTTTTG--CAGAGGACTGAAAATC------CTTGTGTCACCATTCGTAAAATGAGGATGATACTTCGGTAATGGCCGGGATAGCTCAgttg

trnLF_IC AATTGGATTGAGCCTTGGTATGGAAACCTACTAAGTGATATCTTTCAAATTCAGAGAAACCCTGGAATTAAcaatgggcAATCCTGAGCCAAATCCTGGTTTACGTGAACAAACCGGAGTTTAGAAAGCGAGAAAAAA-GGGATAGGTGCAGAGACTCAATGGAAGCTGTTCTAACAAATGGAGTTCACTACCTTGTGTTGATAAAGGAATCCTTCGATCAAAACTTCAAATAAAAAAG-GATGAAGGAGAAAAACCTATATTGTCTAAATATA------GGTAACACAAAA-CGATCGCAAAAATGACGACCTGAATCTCGATTTCTATTTTTTT--ATAAACAAAATCGAAATGATGTGAATCAATTCGAA-----GTTTAAGAAAtaatattcattGATCAAATGATTCTCTTCATAGTCTGATAGATCCGTGGTGGAActtattaaTCGGCCGAGAATAAAGATAGAGTCCCATTTTAC-ATGTCAATACTGACAACAATGAAATTTATAGTAAGATGXXXGCTCTACT-CCCCAAAAAGGTCTGTTTGACACCTTACCTtttt-ttag-TTATTATCCA-TTTGAATTATTTAGAATCTATATCAGTTTTCATTTTCAAACTTAGAAAGTCTTCTTTTATTTATAAGATCCAAGAAATTCCCGGTCCAAAACTTTTTTAATTTACTACtttt------gagttTCTGTTCATTGACAGAGACCCAAGTCATATATTAAAATGATACTGATacttc--------------------------------------cgtaaTGGTCGGCATAGCTTAATTGCGGAGGACTGAAAATCCTTGTGTCACCATT--------------------------------------------------------------------------------------------AGTAAAATGATAC-TGATACTTCAGTAGATGATACCTCAGTAATGGTGGACATAGC-------TTTTTTG--CAGAGGACTGAAAATC------CTTGTGTCACCATTCGTAAAATGAGGATGATACTTCGGTAATGGCCGGGATAGCTCAgttg

trnLF_ID AATTGGATTGAGCCTTGGTATGGAAACCTACTAAGTGATAACTTTCAAATTCAGAGAAACCCTGGAATTAAcaatgggcAATCCTGAGCCAAATCCTGGTTTACGTGAACAAACCGGAGTTTAGAAAGCGAGAAAAAA-GGGATAGGTGCAGAGACTCAATGGAAGCTGTTCTAACAAATGGAGTTCACTACCTTGTGTTGATAAAGGAATCCTTCGATCAAAACTTCAAATAAAAAAG-GATGAAGGAGAAAAACCTATATTGTCTAAATATA------GGTAACACAAAA-CGATCGCAAAAATGACGACCTGAATCTCGATTTCTATTTTTTT--ATAAACAAAATCGAAATGATGTGAATCAATTCGAA-----GTTTAAGAAAtaatattcattGATCAAATGATTCTCTTCATAGTCTGATAGATCCGTGGTGGAActtattaaTCGGCCGAGAATAAAGATAGAGTCCCATTTTAC-ATGTCAATACTGACAACAATGAAATTTATAGTAAGATGXXXGCTCTACT-CCCCAAAAAGGTCTGTTTGACACCTTACCTtttt-ttag-TTATTATCCA-TTTGAATTATTTAGAATCTATATCAGTTTTCATTTTCAAACTTAGAAAGTCTTCTTTTATTTATAAGATCCAAGAAATTCCCGGTCCAAAACTTTTTTAATTTACTACtttt------gagttTCTGTTCATTGACAGAGACCCAAGTCATATATTAAAATGATACTGATACTTC--------------------------------------CGGAATGGTCGGCATAGCTTAATTGCGGAGGACTGAAAATCCTTGTGTCACCATT--------------------------------------------------------------------------------------------AGTAAAATGATAC-TGATACTTCAGTAGATGATACCTCAGTAATGGTGGACATAGC-------TTTTTTG--CAGAGGACTGAAAATC------CTTGTGTCACCATTCGTAAAATGAGGATGATACTTCGGTAATGGCCGGGATAGCTCAgttg

trnLF_IE AATTGGATTGAGCCTTGGTATGGAAACCTACTAAGTGATAACTTTCAAATTCAGAGAAACCCTGGAATTAAcaatgggcAATCCTGAGCCAAATCCTGGTTTACGTGAACAAACCGGAGTTTAGAAAGCGAGAAAAAA-GGGATAGGTGCAGAGACTCAATGGAAGCTGTTCTAACAAATGGAGTTCACTACCTTGTGTTGATAAAGGAATCCTTCGATCAAAACTTCAAATCAAAAAG-GATGAAGGAGAAAAACCTATATTGTCTAAATATA------GGTAACACAAAA-CGATCGCAAAAATGACGACCTGAATCTCGATTTCTATTTTTTT--ATAAACAAAATCGAAATGATGTGAATCAATTCGAA-----GTTTAAGAAAtaatattcattGATCAAATGATTCTCTTCATAGTCTGATAGATCCGTGGTGGAActtattaaTCGGCCGAGAATAAAGATAGAGTCCCATTTTAC-ATGTCAATACTGACAACAATGAAATTTATAGTAAGATGXXXGCTCTACT-CCCCAAAAAGGTCTGTTTGACACCTTACCTtttt-ttag-TTATTATCCA-TTTGAATTATTTAGAATCTATATCAGTTTTCATTTTCAAACTTAGAAAGTCTTCTTTTATTTATAAGATCCAAGAAATTCCCGGTCCAAAACTTTTTTAATTTACTACtttt------gagttTCTGTTCATTGACAGAGACCCAAGTCATATATTAAAATGATACTGATacttc--------------------------------------cgtaaTGGTCGGCATAGCTTAATTGCGGAGGACTGAAAATCCTTGTGTCACCATTAGTAAAATGATACTGATACTTCAGTAGATGATACCTCAGTAATGGTGGACATAGCTTTTTTGCAGAGGACTGAAAATCCTTGTGTCACCATTAGTAAAATGATAC-TGATACTTCAGTAGATGATACCTCAGTAATGGTGGACATAGC-------TTTTTTG--CAGAGGACTGAAAATC------CTTGTGTCACCATTCGTAAAATGAGGATGATACTTCGGTAATGGCCGGGATAGCTCAgttg

AY257761 AATTGGATTGAGCCTTGGTATGGAAACCTACTAAGTGATAACTTTCAAATTCAGAGAAACCCTGGAATTAAcaatgggcAATCCTGAGCCAAATCCTGGTTTATGTGAACAAACCGGAGTTTAGAAAGCGAGAAAAAA-GGGATAGGTGCAGAGACTCAATGGAAGCTGTTCTAACAAATGGAGTTCACTACCTTGTGTTGATAAAGGAATCCTTCGATCAAAACTTCAAATAAAAAAG-GATGAAGGAGAAAAACCTATATTGTCTAAATATA------GGTAACACAAAA-CGATCTCAAAAATGACGACCTGAATCTCGATTTCTATTTTTTT--ATAAACAAAATCGAAATGATGTGAATCAATTCGAA-----GTTTAAGAAAtaatattcattGATCAAATGATTCTCTTCATAGTCTGATAGATCCGTGGTGGAActtattaaTCGGACGAGAATAAAGATAGAGTCCCATTTTAC-ATGTCAATACTGACAACAATGAAATTTATAGTAAGATGXXXGCTCTACT-CCCCAAAAAGGTCTGTTTGACACCTTACCTtttt-gtag-TTATTATCCA-TTTGAATTATTTAGAATCTATATCAGTTTTCATTTTCAAACTTAGAAAGTCTTCTTTTATTTATAAGATCCAAAAAATTCCCGGTCCAAAACTTTTTTAATTTACTACtttt------gagttTCTTTTCATTGACAGAGACCCAAGTCATATATTAAAATGATACTGATacttc--------------------------------------cgtaaTGGTCGGCATAGCTTAATTGCGGAGGACTGAAAATCCTTGTGTCACCATT--------------------------------------------------------------------------------------------AGTAAAATGATAA-TGATACTTCAGTAGATGATACCTCAGTAATGGTGGACATAGC-------TTTTTTG--CAGAGGACTGAAAATC------CTTGTGTCACCATTCGTAAAATGAGGATGATACTTCGGTAATGGCCGGGATAGCTCAGNNn

AY257762 AATTGGATTGAGCCTTGGTATGGAAACCTACTAAGTGATAACTTTCAAATTCAGAGAAACCCTGGAATTAAcaatgggcAATCCTGAGCCAAATCCTGGTTTATGTGAACAAACCGGAGTTTAGAAAGCGAGAAAAAA-GGGATAGGTGCAGAGACTCAATGGAAGCTGTTCTAACAAATGGAGTTCACTACCTTGTGTTGATAAAGGAATCCTTCGATCAAAACTTCAAATAAAAAAG-GATGAAGGAGAAAAACCTATATTGTATAAATATA------GGTAACACAAAA-CGATCTCAAAAATGACGACCTGAATCTCGATTTCTATTTTTTT--ATAAACAAAATCGAAATGATGTGAATCAATTCGAA-----GTTTAAGAAAtaatattcattGATCAAATGATTCTCTTCATAGTCTGATAGATCCGTGGTGGAActtattaaTCGGACGAGAATAAAGATAGAGTCCCATTTTAC-ATGTCAATACTGACAACAATGAAATTTATAGTAAGATGXXXGCTCTACT-CCCCAAAAAGGTCTGTTTGACACCTTACCTtttt-gtag-TTATTATCCA-TTTGAATTATTTAGAATCTATATCAGTTTTCATTTTCAAACTTAGAAAGTCTTCTTTTATTTATAAGATCCAAAAAATTCCCGGTCCAAAACTTTTTTAATTTACTACtttt------gagttTCTTTTCATTGACAGAGACCCAAGTCATATATTAAAATGATACTGATacttc--------------------------------------cgtaaTGGTCGGCATAGCTTAATTGCGGAGGACTGAAAATCCTTGTGTCACCATT--------------------------------------------------------------------------------------------AGTAAAATGATAA-TGATACTTCAGTAGATGATACCTCAGTAATGGTGGACATAGC-------TTTTTTG--CAGAGGACTGAAAATC------CTTGTGTCACCATTCGTAAAATGAGGATGATACTTCGGTAATGGCCGGGATAGCTCAgttg

AY257763 NNNNNNNNTGAGCCTTGGTATGGAAACCTACTAAGTGATAACTTTCAAATTCAGAGAAACCCTGGAATTAAcaatgggcAATCCTGAGCCAAATCCTGGTTTATGTGAACAAACCGGAGTTTAGAAAGCGAGAAAAAA-GGGATAGGTGCAGAGACTCAATGGAAGCTGTTCTAACAAATGGAGTTCACTACCTTGTGTTGATAAAGGAATCCTTCGATCAAAACTTCAAATAAAAAAG-GATGAAGGAGAAAAACCTATATTGTCTAAATATA------GGTAACACAAAA-CGATCTCAAAAATGACGACCTGAATCTCGATTTCTATTTTTTT--ATAAACAAAATCGAAATGATGTGAATCAATTCGAA-----GTTTAAGAAAtaatattcattGATCAAATGATTCTCTTCATAGTCTGATAGATCCGTGGTGGAActtattaaTCGGACGAGAATAAAGATAGAGTCCCATTTTAC-ATGTCAATACTGACAACAATGAAATTTATAGTAAGATGXXXGCTCTACT-CCCCAAAAAGGTCTGTTTGACACCTTACCTtttt-gtag-TTATTATCCA-TTTGAATTATTTAGAATCTATATCAGTTTTCATTTTCAAACTTAGAAAGTCTTCTTTTATTTATAAGATCCAAAAAATTCCCGGTCCAAAACTTTTTTAATTTACTACtttt------gagttTCTTTTCATTGACAGAGACCCAAGTCATATATTAAAATGATACTGATacttc--------------------------------------cgtaaTGGTCGGCATAGCTTAATTGCGGAGGACTGAAAATCCTTGTGTCACCATT--------------------------------------------------------------------------------------------------------------------------------------------------------------------------------------------------------CGTAAAATGAGGATGATACTTCGGTAATGGCCGGGATAGCTCAgttg

AY257764 NNNNNNNTTGANCCTTGGTATGGNAACCTACTAAGTGATAACTTTCAAATTCAGAGAAACCCTGGAATTAAcaatgggcAATCCTGAGCCAAATCCTGGTTTATGTGAACAAACCGGAGTTTAGAAAGCGAGAAAAAA-GGGATAGGTGCAGAGACTCAATGGAAGCTGTTCTAACAAATGGAGTTCACTACCTTGTGTTGATAAAGGAATCCTTCGATCAAAACTTCAAATAAAAAAG-GATGAAGGAGAAAAACCTATATTGTCTAAATATA------GGTAACACAAAA-CGATCTCAAAAATGACGACCTGAATCTCGATTTCTATTTTTTT--ATAAACAAAATCGAAATGATGTGAATCAATTCGAA-----GTTTAAGAAAtaatattcattGATCAAATGATTCTCTTCATAGTCTGATAGATCCGTGGTGGAActtattaaTCGGACGAGAATAAAGATAGAGTCCCATTTTAC-ATGTCAATACTGACAACAATGAAATTTATAGTAAGATGXXXGCTCTACT-CCCCAAAAAGGTCTGTTTGACACCTTACCTtttt-gtag-TTATTATCCA-TTTGAATTATTTAGAATCTACATCAGTTTTCATTTTCAAACTTAGAAAGTCTTCTTTTATTTATAAGATCCAAAAAATTCCCGGTCCAAAACTTTTTTAATTTACTACtttt------gagttTCTTTTCATTGACAGAGACCCAAGTCATATATTAAAATGATACTGATacttc--------------------------------------cgtaaTGGTCGGCATAGCTTAATTGCGGAGGACTGAAAATCCTTGTGTCACCATT--------------------------------------------------------------------------------------------AGTAAAATGATAA-TGATACTTCAGTAGATGATACCTCAGTAATGGTGGACATAGC-------TTTTTTG--CAGAGGACTGAAAATC------CTTGTGTCACCATTCGTAAAATGAGGATGATACTTCGGTAATGGCCGGGATAGCTCAgttg

AY257765 AATTGGATTGAGCCTTGGTATGGAAACCTACTAAGTGATAACTTTCAAATTCAGAGAAACCCTGGAATTAAcaatgggcAATCCTGAGCCAAATCCTGGTTTATGTGAACAAACCGGAGTTTAGAAAGCGAGAAAAAA-GGGATAGGTGCAGAGACTCAATGGAAGCTGTTCTAACAAATGGAGTTCACTACCTTGTGTTGATAAAGGAATCCTTCGATCAAAACTTCAAATAAAAAAG-GATGAAGGAGAAAAACCTATATTGTCTAAATATA------GGTAACACAAAA-CGATCTCAAAAATGACGACCTGAATCTCGATTTCTATTTTTTT--ATAAACAAAATCGAAATGATGTGAATCAATTCGAA-----GTTTAAGAAAtaatattcattGATCAAATGATTCTCTTCATAGTCTGATAGATCCGTGGTGGAActtattaaTCGGACGAGAATAAAGATAGAGTCCCATTTTAC-ATGTCAATACTGACAACAATGAAATTTATAGTAAGATGXXXNNNNNNCT-CCCCAAAAAGGTCTGTTTGACACCTTACCTtttt-gtag-TTATTATCCA-TTTGAATTATTTAGAATCTACATCAGTTTTCATTTTCAAACTTAGAAAGTCTTCTTTTATTTATAAGATCCAAAAAATTCCCGGTCCAAAACTTTTTTAATTTACTACtttt------gagttTCTTTTCATTGACAGAGACCCAAGTCATATATTAAAATGATACTGATacttc--------------------------------------cgtaaTGGTCGACATAGCTTAATTGCGGAGGACTGAAAATCCTTGTGTCACCATT--------------------------------------------------------------------------------------------ATTAAAATGATAA-TGATACTTCAGTAGATGATACCTCAGTAATGGTGGACATAGC-------TTTTTTG--CAGAGGACTGAAAATC------CTTGTGTCACCATTCGTAAAATGAGGATGATACTTCGGTAATGGCCGGGATAGCTCAgttg

AY257766 AATTGGATTGAGCCTTGGTATGGAAACCTACTAAGTGATAACTTTCAAATTCAGAGAAACCCTGGAATTAAcaatgggcAATCCTGAGCCAAATCCTGGTTTATGTGAACAAACCGGAGTTTAGAAAGCGAGAAAAAA-GGGATAGGTGCAGAGACTCAATGGAAGCTGTTCTAACAAATGGAGTTCACTACCTTGTGTTGATAAAGGAATCCTTCGATCAAAACTTCAAATAAAAAAG-GATGAAGGAGAAAAACCTATATTGTCTAAATATA------GGTAACACAAAA-CGATCTCAAAAATGACGACCTGAATCTCGATTTCTATTTTTTT--ATAAACAAAATCGAAATGATGTGAATCAATTCGAA-----GTTTAAGAAAtaatattcattGATCAAATGATTCTCTTCATAGTCTGATAGATCCGTGGTGGAActtattaaTCGGACGAGAATAAAGATAGAGTCCCATTTTAC-ATGTCAATACTGACAACAATGAAATTTATAGTAAGATGXXXGCTCTACT-CCCCAAAAAGGTCTGTTTGACACCTTACCTtttt-gtag-TTATTATCCA-TTTGAATTATTTAGAATCTACATCAGTTTTCATTTTCAAACTTAGAAAGTCTTCTTTTATTTATAAGATCCAAAAAATTCCCGGTCCAAAACTTTTTTAATTTACTACtttt------gagttTCTTTTCATTGACAGAGACCCAAGTCATATATTAAAATGATACTGATacttc--------------------------------------cgtaaTGGTCGGCATAGCTTAATTGCGGAGGACTGAAAATCCTTGTGTCACCATT--------------------------------------------------------------------------------------------ATTAAAATGATAA-TGATACTTCAGTAGATGATACCTCAGTAATGGTGGACATAGC-------TTTTTTG--CAGAGGACTGAAAATC------CTTGTGTCACCATTCGTAAAATGAGGATGATACTTCGGTAATGGCCGGGATAGCTCAgttg

AY257767 AATTGGATTGAGCCTTGGTATGGAAACCTACTAAGTGATAACTTTCAAATTCAGAGAAACCCTGGAATTAAcaatgggcAATCCTGAGCCAAATCCTGGTTTATGTGAACAAACCGGAGTTTAGAAAGCGAGAAAAAA-GGGATAGGTGCAGAGACTCAATGGAAGCTGTTCTAACAAATGGAGTTCACTACCTTGTGTTGATAAAGGAATCCTTCGATCAAAACTTCAAATAAAAAAG-GATGAAGGAGAAAAACCTATATTGTCTAAATATA------GGTAACACAAAA-CGATCTCAAAAATGACGACCTGAATCTCGATTTCTATTTTTTT--ATAAACAAAATCGAAATGATGTGAATCAATTCGAA-----GTTTAAGAAAtaatattcattGATCAAATGATTCTCTTCATAGTCTGATAGATCCGTGGTGGAActtattaaTCGGACGAGAATAAAGATAGAGTCCCATTTTAC-ATGTCAATACTGACAACAATGAAATTTATAGTAAGATGXXXGCTCTACT-CCCCAAAAAGGTCTGTTTGACACCTTACCTtttt-gtag-TTATTATCCA-TTTGAATTATTTAGAATCTACATCAGTTTTCATTTTCAAACTTAGAAAGTCTTCTTTTATTTATAAGATCCAAAAAATTCCCGGTCCAAAACTTTTTTAATTTACTACtttt------gagttTCTTTTCATTGACAGAGACCCAAGTCATATATTAAAATGATACTGATacttc--------------------------------------cgtaaTGGTCGGCATAGCTTAATTGCGGAGGACTGAAAATCCTTGTGTCACCATT--------------------------------------------------------------------------------------------AGTAAAATGATAA-TGATACTTCAGTAGATGATACCTTAGTAATGGTGGACATAGC-------TTTTTTG--CAGAGGACTGAAAATC------CTTGTGTCACCATTCGTAAAATGAGGATGATACTTCGGTAATGGCCGGGATAGCTCAgttg

AY257768 AATTGGATTGAGCCTTGGTATGGAAACCTACTAAGTGATAACTTTCAAATTCAGAGAAACCCTGGAATTAAcaatgggcAATCCTGAGCCAAATCCTGGTTTATGTGAACAAACCGGAGTTTAGAAAGCGAGAAAAAA-GGGATAGGTGCAGAGACTCAATGGAAGCTGTTCTAACAAATGGAGTTCACTACCTTGTGTTGATAAAGGAATCCTTCGATCAAAACTTCAAATAAAAAAG-GATGAAGGAGAAAAACCTATATTGTCTAAATATA------GGTAACACAAAA-CGATCTCAAAAATGACGACCTGAATCTCGATTTCTATTTTTTT--ATAAACAAAATCGAAATGATGTGAATCAATTCGAA-----GTTTAAGAAAtaatattcattGATCAAATGATTCTCTTCATAGTCTGATAGATCCGTGGTGGAActtattaaTCGGACGAGAATAAAGATAGAGTCCCATTTTAC-ATGTCAATACTGACAACAATGAAATTTATAGTAAGATGXXXGCTCTACT-CCCCAAAAAGGTCTGTTTGACACCTTACCTtttt-gtag-TTATTATCCA-TTTGAATTATTTAGAATCTACATCAGTTTTCATTTTCAAACTTAGAAAGTCTTCTTTTATTTATAAGATCCAAAAAATTCCCGGTCCAAAACTTTTTTAATTTACTACtttt------gagttTCTTTTCATTGACAGAGACCCAAGTCATATATTAAAATGATACTGATacttc--------------------------------------cgtaaTGGTCGGCATAGCTTAATTGCGGAGGACTGAAAATCCTTGTGTCACCATT--------------------------------------------------------------------------------------------AGTAAAATGATAA-TGATACTTCAGTAGATGATACCTCAGTAATGGTGGACATAGC-------TTTTTTG--CGGAGGACTGAAAATC------CTTGTGTCACCATTCGTAAAATGAGGATGATACTTCGGTAATGGCCGGGATAGCTCAgttg

AY257769 AATTGGATTGAGCCTTGGTATGGAAACCTACTAAGTGATAACTTTCAAATTCAGAGAAACCCTGGAATTAAcaatgggcAATCCTGAGCCAAATCCTGGTTTATGTGAACAAACCGGAGTTTAGAAAGCGAGAAAAAA-GGGATAGGTGCAGAGACTCAATGGAAGCTGTTCTAACAAATGGAGTTCACTACCTTGTGTTGATAAAGGAATCCTTCGATCAAAACTTCAAATAAAAAAG-GATGAAGGAGAAAAACCTATATTGTCTAAATATA------GGTAACACAAAA-CGATCTCAAAAATGACGACCTGAATCTCGATTTCTATTTTTTT--ATAAACAAAATCGAAATGATGTGAATCAATTCGAA-----GTTTAAGAAAtaatattcattGATCAAATGATTCTCTTCATAGTCTGATAGATCCGTGGTGGAActtattaaTCGGACGAGAATAAAGATAGAGTCCCATTTTAC-ATGTCAATACTGACAACAATGAAATTTATAGTAAGATGXXXGCTCTACT-CCCCAAAAAGGTCTGTTTGACACCTTACCTtttt-gtag-TTATTATCCA-TTTGAATTATTTAGAATCTACATCAGTTTTCATTTTCAAACTTAGAAAGTCTTCTTTTATTTATAAGATCCAAAAAATTCCCGGTCAAAAACTTTTTTAATTTACTACtttt------gagttTCTTTTCATTGACAGAGACCCAAGTCATATATTAAAATGATACTGATacttc--------------------------------------cgtaaTGGTCGGCATAGCTTAATTGCGGAGGACTGAAAATCCTTGTGTCACCATT--------------------------------------------------------------------------------------------AGTAAAATGATAA-TGATACTTCAGTAGATGATACCTCAGTAATGGTGGACATAGC-------TTTTTTG--CAGAGGACTGAAAATC------CTTGTGTCACCATTCGTAAAATGAGGATGATACTTCGGTAATGGCCGGGATAGCTCAgttg

AY257770 AATTGGATTGAGCCTTGGTATGGAAACCTACTAAGTGATAACTTTCAAATTCAGAGAAACCCTGGAATTAAcaatgggcAATCCTGAGCCAAATCCTGGTTTATGTGAACAAACCGGAGTTTAGAAAGCGAGAAAAAA-GGGATAGGTGCAGAGACTCAATGGAAGCTGTTCTAACAAATGGAGTTCACTACCTTGTGTTGATAAAGGAATCCTTCGATCAAAACTTCAAATAAAAAAG-GATGAAGGAGAAAAACCTATATTGTCTAAATATA------GGTAACACAAAA-CGATCTCAAAAATGACGACCTGAATCTCGATTTCTATTTTTTT--ATAAACAAAATCGAAATGATGTGAATCAATTCGAA-----GTTTAAGAAAtaatattcattGATCAAATGATTCTCTTCATAGTCTGATAGATCCGTGGTGGAActtattaaTCGGACGAGAATAAAGATAGAGTCCCATTTTAC-ATGTCAATACTGACAACAATGAAATTTATAGTAAGATGXXXGCTCTACT-CCCCAAAAAGGTCTGTTTGACACCTTACCTtttt-gtag-TTATTATCCA-TTTGAATTATTTAGAATCTACATCAGTTTTCATTTTCAAACTTAGAAAGTCTTCTTTTATTTATAAGATCCAAAAAATTCCCGGTCCAAAACTTTTTTAATTTACTACtttt------gagttTCTTTTCATTGACAGAGACCCAAGTCATATATTAAAATGATACTGATacttc--------------------------------------cgtaaTGGTCGGCATAGCTTAATTGCGGAGGACTGAAAATCCTTGTGTCACCATTAGTAAAATGATAATGATACTTCAGTAGATGATACCTCAGTAATGGTGGACATAGCTTTTTTGCAGAGGACTGAAAATCCTTGTGTCACCATTAGTAAAATGATAA-TGATACTTCAGTAGATGATACCTCAGTAATGGTGGACATAGC-------TTTTTTG--CAGAGGACTGAAAATC------CTTGTGTCACCATTCGTAAAATGAGGATGATACTTCGGTAATGGCCGGGATAGCTCAgttg

AY257771 NNNNNNNNNNNNNNNNNNNNNNNNNNNNNNNNNNNNNNNNNCTTTCAAATTCAGAGAAACCCTGGAATTAAcaatgggcAATCCTGAGCCAAATCCTGGTTTATGTGAACAAACCGGAGTTTAGAAAGCGAGAAAAAA-GGGATAGGTGCAGAGACTCAATGGAAGCTGTTCTAACAAATGGAGTTCACTACCTTGTGTTGATAAAGGAATCCTTCGATCAAAACTTCAAATAAAAAAG-GATGAAGGAGAAAAACCTATATTGTCTAAATATA------GGTAACACAAAA-CGATCTCAAAAATGACGACCTGAATCTCGATTTCTATTTTTTT--ATAAACAAAATCGAAATGATGTGAATCAATTCGAA-----GTTTAAGAAAtaatattcattGATCAAATGATTCTCTTCATAGTCTGATAGATCCGTGGTGGAActtattaaTCGGACGAGAATAAAGATAGAGTCCCATTTTAC-ATGTCAATACTGACAACAATGAAATTTATAGTAAGATGXXXGCTCTACT-CCCCAAAAAGGTCTGTTTGACACCTTACCTtttt-gtag-TTATTATCCA-TTTGAATTATTTAGAATCTACATCAGTTTTCATTTTCAAACTTAGAAAGTCTTCTTTTATTTATAAGATCCAAAAAATTCCCGGTCCAAAACTTTTTTAATTTACTACtttt------gagttTCTTTTCATTGACAGAGACCCAAGTCATATATTAAAATGATACTGATacttc--------------------------------------cgtaaTGGTCGGCATAGCTTAATTGCGGAGGACTGAAAATCCTTGTGTCACCATT--------------------------------------------------------------------------------------------------------------------------------------------------------------------------------------------------------CGTAAAATGAGGATGATACTTCGGTAATGGCCGGGATAGCTCAgttg

AY257772 AATTGGATTGAGCCTTGGTATGGAAACCTACTAAGTGATAACTTTCAAATTCAGAGAAACCCTGGAATTAAcaatgggcAATCCTGAGCCAAATCCTGGTTTATGTGAACAAACCGGAGTTTAGAAAGCGAGAAAAAA-GGGATAGGTGCAGAGACTCAATGGAAGCTGTTCTAACAAATGGAGTTCACTACCTTGTGTTGATAAAGGAATCCTTCGATCAAAACTTCAAATAAAAAAG-GATGAAGGAGAAAAACCTATATTGTCTAAATATA------GGTAACACAAAA-CGATCTCAAAAATGACGACCTGAATCTCGATTTCTATTTTTTT--ATAAACAAAATCGAAATGATGTGAATCAATTCGAA-----GTTTAAGAAAtaatattcattGATCAAATGATTCTCTTCATAGTCTGATAGATCCGTGGTGGAActtattaaTCGGACGAGAATAAAGATAGAGTCCCATTTTAC-ATGTCAATACTGACAACAATGAAATTTATAGTAAGATGXXXNNNNNNNN-NNNNNNNNNNNTCTGTTTGACACCTTACCTtttt-gtag-TTATTATCCA-TTTGAATTATTTAGAATCTACATCAGTTTTCATTTTCAAACTTAGAAAGTCTTCTTTTATTTATAAGATCCAAAAAATTCCCGGTCCAAAACTTTTTAAATTTACTACtttt------gagttTCTTTTCATTGACAGAGACCCAAGTCATATATTAAAATGATACTGATacttc--------------------------------------cgtaaTGGTCGGCATAGCTTAATTGCGGAGGACTGAAAATCCTTGTGTCACCATT--------------------------------------------------------------------------------------------AGTAAAATGATAA-TGATACTTCAGTAGATGATACCTCAGTAATGGTGGACATAGC-------TTTTTTG--CAGAGGACTGAAAATC------CTTGTGTCACCATTCGTAAAATGAGGATGATACTTCGGTAATGGCCGGGATAGCTCAgttg

AY257773 AATTGGATTGAGCCTTGGTATGGAAACCTACTAAGTGATAACTTTCAAATTCAGAGAAACCCTGGAATTAAcaatgggcAATCCTGAGCCAAATCCTGGTTTATGTGAACAAACCGGAGTTTAGAAAGCGAGAAAAAA-GGGATAGGTGCAGAGACTCAATGGAAGCTGTTCTAACAAATGGAGTTCACTACCTTGTGTTGATAAAGGAATCCTTCGATCAAAACTTCAAATAAAAAAG-GATGAAGGAGAAAAACCTATATTGTCTAAATATA------GGTAACACAAAA-CGATCTCAAAAATGACGACCTGAATCTCGATTTCTATTTTTTT--ATAAACAAAATCGAAATGATGTGAATCAATTCGAA-----GTTTAAGAAAtaatattcattGATCAAATGATTCTCTTCATAGTCTGATAGATCCGTGGTGGAActtattaaTCGGACGAGAATAAAGATAGAGTCCCATTTTAC-ATGTCAATACTGACAACAATGAAATTTATAGTAAGATGXXXGCTCTACT-CCCCAAAAAGGTCTGTTTGACACCTTACCTtttt-gtag-TTATTATGCA-TTTGAATTATTTAGAATCTACATCAGTTTTCATTTTCAAACTTAGAAAGTCTTCTTTTATTTATAAGATCCAAAAAATTCCCGGTCCAAAACTTTTTAAATTTACTACtttt------gagttTCTTTTCATTGACAGAGACCCAAGTCATATATTAAAATGATACTGATacttc--------------------------------------cgtaaTGGTCGGCATAGCTTAATTGCGGAGGACTGAAAATCCTTGTGTCACCATT--------------------------------------------------------------------------------------------AGTAAAATGATAA-TGATACTTCAGTAGATGATACCTCAGTAATGGTGGACATAGC-------TTTTTTG--CAGAGGACTGAAAATC------CTTGTGTCACCATTCGTAAAATGAGGATGATACTTCGGTAATGGCCGGGATAGCTCAgttg

AY257774 AATTGGATTGAGCCTTGGTATGGAAACCTACTAAGTGATAACTTTCAAATTCAGAGAAACCCTGGAATTAAcaatgggcAATCCTGAGCCAAATCCTGGTTTATGTGAACAAACCGGAGTTTAGAAAGCGAGAAAAAA-GGGATAGGTGCAGAGACTCAATGGAAGCTGTTCTAACAAATGGAGTTCACTACCTTGTGTTGATAAAGGAATTCTTCGATCAAAACTTCAAATAAAAAAG-GATGAAGGAGAAAAACCTATATTGTCTAAATATA------GGTAACACAAAA-CGATCTCAAAAATGACGACCTGAATCTCGATTTCTATTTTTTT--ATAAACAAAATCGAAATGATGTGAATCAATTCGAA-----GTTTAAGAAAtaatattcattGATCAAATGATTCTCTTCATAGTCTGATAGATCCGTGGTGGAActtattaaTCGGACGAGAATAAAGATAGAGTCCCATTTTAC-ATGTCAATACTGACAACAATGAAATTTATAGTAAGATGXXXGCTCTACT-CCCCAAAAAGGTCTGTTTGACACCTTACCTtttt-gtag-TTATTATCCA-TTTGAATTATTTAGAATCTACATCAGTTTTCATTTTCAAACTTAGAAAGTCTTCTTTTATTTATAAGATCCAAAAAATTCCCGGTCCAAAACTTTTTAAATTTACTACtttt------gagttTCTTTTCATTGACAGAGACCCAAGTCATATATTAAAATGATACTGATacttc--------------------------------------cgtaaTGGTCGGCATAGCTTAATTGCGGAGGACTGAAAATCCTTGTGTCACCATT--------------------------------------------------------------------------------------------AGTAAAATGATAA-TGATACTTCAGTAGATGATACCTCAGTAATGGTGGACATAGC-------TTTTTTG--CAGAGGACTGAAAATC------CTTGTGTCACCATTCGTAAAATGAGGATGATACTTCGGTAATGGCCGGGATAGCTCAgttg

AY257775 AATTGGATTGAGCCTTGGTATGGAAACCTACTAAGTGATAACTTTCAAATTCAGAGAAACCCTGGAATTAAcaatgggcAATCCTGAGCCAAATCCTGGTTTATGTGAACAAACCGGAGTTTAGAAAGCGAGAAAAAA-GGGATAGGTGCAGAGACTCAATGGAAGCTGTTCTAACAAATGGAGTTCACTACCTTGTGTTGATAAAGGAATCCTTCGATCAAAACTTCAAATAAAAAAG-GATGAAGGAGAAAAACCTATATTGTCTAAATATA------GGTAACACAAAA-CGATCTCAAAAATGACGACCTGAATCTAGATTTCTATTTTTTT--ATAAACAAAATCGAAATGATGTGAATCAATTCGAA-----GTTTAAGAAAtaatattcattGATCAAATGATTCTCTTCATAGTCTGATAGATCCGTGGTGGAActtattaaTCGGACGAGAATAAAGATAGAGTCCCATTTTAC-ATGTCAATACTGACAACAATGAAATTTATAGTAAGATGXXXGCTCTACT-CCCCAAAAAGGTCTGTTTGACACCTTACCTtttt-gtag-TTATTATCCA-TTTGAATTATTTAGAATCTACATCAGTTTTCATTTTCAAACTTAGAAAGTCTTCTTTTATTTATAAGATCCAAAAAATTCCCGGTCCAAAACTTTTTTAATTTACTACtttt------gagttTCTTTTCATTGACAGAGACCCAAGTCATATATTAAAATGATACTGATacttc--------------------------------------cgtaaTGGTCGGCATAGCTTAATTGCGGAGGACTGAAAATCCTTGTGTCACCATT--------------------------------------------------------------------------------------------AGTAAAATGATAA-TGATACTTCAATAGATGATACCTCAGTAATGGTGGACATAGC-------TTTTTTG--CAGAGGACTGAAAATC------CTTGTGTCACCATTCGTAAAATGAGGATGATACTTCGGTAATGGCCGGGATAGCTCAgttg

AY257776 AATTGGATTGAGCCTTGGTATGGAAACCTACTAAGTGATAACTTTCAAATTCAGAGAAACCCTGGAATTAAcaatgggcAATCCTGAGCCAAATCCTGGTTTATGTGAACAAACCGGAGTTTAGAAAGCGAGAAAAAA-GGGATAGGTGCAGAGACTCAATGGAAGCTGTTCTAACAAATGGAGTTCACTACCTTGTGTTGATAAAGGAATCCTTCGATCAAAACTTCAAATAAAAAAG-GATGAAGGAGAAAAACCTATATTGTCTAAATATA------GGTAACACAAAA-CGATCTCAAAAATGACGACCTGAATCTAGATTTCTATTTTTTT--ATAAACAAAATCGAAATGATGTGAATCAATTCGAA-----GTTTAAGAAAtaatattcattGATCAAATGATTCTCTTCATAGTCTGATAGATCCGTGGTGGAActtattaaTCGGACGAGAATAAAGATAGAGTCCCATTTTAC-ATGTCAATACTGACAACAATGAAATTTATAGTAAGATGXXXGCTCTACT-CCCCAAAAAGGTCTGTTTGACACCTTACCTtttt-gtag-TTATTATCCA-TTTGAATTATTTAGAATCTACATCAGTTTTCATTTTCAAACTTAGAAAGTCTTCTTTTATTTATAAGATCCAAAAAATTCCCGGTCCAAAACTTTTTTAATTTACTACtttt------gagttTCTTTTCATTGACAGAGACCCAAGTCATATATTAAAATGATACTGATacttc--------------------------------------cgtaaTGGTCGGCATAGCTTAATTGCGGAGGACTGAAAATCCTTGTGTCACCATT--------------------------------------------------------------------------------------------AGTAAAATGATAA-TGATACTTCAGTAGATGATACCTCAGTAATGGTGGACATAGC-------TTTTTTG--CAGAGGACTGAAAATC------CTTGTGTCACCATTCGTAAAATGAGGATGATACTTCGGTAATGGCCGGGATAGCTCAgttg

AY257777 AATTGGATTGAGCCTTGGTATGGAAACCTACTAAGTGATAACTTTCAAATTCAGAGAAACCCTGGAATTAAcaatgggcAATCCTGAGCCAAATCCTGGTTTATGTGAACAAACCGGAGTTTAGAAAGCGAGAAAAAA-GGGATAGGTGCAGAGACTCAATGCAAGCTGTTCTAACAAATGGAGTTCACAACCTTGTGTTGATAAAGGAATCCTTCGATCAAAACTTCAAATAAAAAAG-GATGAAGGAGAAAAACCTATATTGTCTAAATATA------GGTAACACAAAA-CGATCTCAAAAATGACGACCTGAATCTCGATTTCTATTTTTTT--ATAAACAAAATCGAAATGATGTGAATCAATTCGAA-----GTTTAAGAAAtaatattcattGATCAAATGATTCTCTTCATAGTCTGATAGATCCGTGGTGGAActtattaaTCGGACGAGAATAAAGATAGAGTCCCATTTTAC-ATGTCAATACTGACAACAATGAAACTTATAGTAAGATGXXXGCTCTACT-CCCCAAAAAGGTCTGTTTGACACCTTACCTtttt-gtag-TTATTATCCA-TTTGAATTATTTAGAATCTATATCAGTTTTCATTTTCAAACTTAGAAAGTCTTCTTTTATTTATAAGATCCAAAAAATTCCCGGTCCAAAACTTTTTTAATTTACTACtttt------gagttTCTTTTCATTGACAGAGACCCAAGTCATATATTAAAATGATACTGATacttc--------------------------------------cgtaaTGGTCGGCATAGCTTAATTGCGGAGGACTGAAAATCCTTGTGTCACCATT--------------------------------------------------------------------------------------------AGTAAAATGATAA-TGATACTTCAGTAGATGATACCTCAGTAATGGTGGACATAGC-------TTTTTTG--CAGAGGACTGAAAATC------CTTGTGTCACCATTCGTAAAATGAGGATGATACTTCGGTAATGGCCGGGATAGCTCAgttg

AY257778 AATTGGATTGAGCCTTGGTATGGAAACCTACTAAGTGATAACTTTCAAATTCAGAGAAACCCTGGAATTAAcaatgggcAATCCTGAGCCAAATCCTGGTTTATGTGAACAAACCGGAGTTTAGAAAGCGAGAAAAAA-GGGATAGGTGCAGAGACTCAATGGAAGCTGTTCTAACAAATGGAGTTCACAACCTTGTGTTGATAAAGGAATCCTTCGATCAAAACTTCAAATAAAAAAG-GATGAAGGAGAAAAACCTATATTGTCTAAATATA------GGTAACACAAAA-CGATCTCAAAAATGACGACCTGAATCTCGATTTCTATTTTTTT--ATAAACAAAATCGAAATGATGTGAATCAATTCGAA-----GTTTAAGAAAtaatattcattGATCAAATGATTCTCTTCATAGTCTGATAGATCCGTGGTGGAActtattaaTCGGACGAGAATAAAGATAGAGTCCCATTTTAC-ATGTCAATACTGACAACAATGAAATTTATAGTAAGANNXXXGCTCTACT-CCCCAAAAAGGTCTGTTTGACACCTTACCTtttt-gtag-TTATTATCCA-TTTGAATTATTTAGAATCTATATCAGTTTTCATTTTCAAACTTAGAAAGTCTTCTTTTATTTATAAGATCCAAAAAATTCCCGGTCCAAAACTTTTTTAATTTACTACtttt------gagttTCTTTTCATTGACAGAGACCCAAGTCATATATTAAAATGATACTGATacttc--------------------------------------cgtaaTGGTCGGCATAGCTTAATTGCGGAGGACTGAAAATCCTTGTGTCACCATT--------------------------------------------------------------------------------------------AGTAAAATGATAA-TGATACTTCAGTAGATGATACCTCAGTAATGGTGGACATAGC-------TTTTTTG--CAGAGGACTGAAAATC------CTTGTGTCACCATTCGTAAAATGAGGATGATACTTCGGTAATGGCCGGGATAGCTCAgttg

AY257779 AATTGGATTGAGCCTTGGTATGGAAACCTACTAAGTGATAACTTTCAAATTCAGAGAAACCCTGGAATTAAcaatgggcAATCCTGAGCCAAATCCTGGTTTATGTGAACAAACCGGAGTTTAGAAAGCGAGAAAAAA-GGGATAGGTGCAGAGACTCAATGGAAGCTGTTCTAACAAATGGAGTTCACAACCTTGTGTTGATAAAGGAATCCTTCGATCAAAACTTCAAATAAAAAAG-GATGAAGGAGAAAAACCTATATTGTCTAAATATA------GGTAACACAAAA-CGATCTCAAAAATGACGACCTGAATCTCGATTTCTATTTTTTT--ATAAACAAAATCGAAATGATGTGAATCAATTCGAA-----GTTTAAGAAAtaatattcattGATCAAATGATTCTCTTCATAGTCTGATAGATCCGTGGTGGAActtattaaTCGGACGAGAATAAAGATAGAGTCCCATTTTAC-ATGTCAATACTGACAACAATGAAATTTATAGTAAGATGXXXNNNNNNNT-CCCCAAAAAGGTCTGTTTGACACCTTACCTtttt-gtag-TTATTATCCA-TTTGAATTATTTAGAATCTATATCAGTTTTCATTTTCAAACTTAGAAAGTCTTCTTTTATTTATAAGATCCAAAAAATTCCCGGTCCAAAACTTTTTTAATTTACTACtttt------gagttTCTTTTCATTGACAGAGACCCAAGTCATATATTAAAATGATACTGATacttc--------------------------------------cgtaaTGGTCGGCATAGCTTAATTGCGGAGGACTGAAAATCCTTGTGTCACCATT--------------------------------------------------------------------------------------------ATTAAAATGATAA-TGATACTTCAGTAGATGATACCTCAGTAATGGTGGACATAGC-------TTTTTTG--CAGAGGACTGAAAATC------CTTGTGTCACCATTCGTAAAATGAGGATGATACTTCGGTAATGGCCGGGATAGCTCAgttg

AY257780 NNNNNNNNNNNNNNNNNNNNNNGAAACCTACTAAGTGATAACTTTCAAATTCAGAGAAACCCTGGAATTAAcaatgggcAATCCTGAGCCAAATCCTGGTTTATGTGAACAAACCGGAGTTTAGAAAGCGAGAAAAAA-GGGATAGGTGCAGAGACTCAATGGAAGCTGTTCTAACAAATGGAGTTCACAACCTTGTGTTGATAAAGGAATCCTTCGATCAAAACTTCAAATAAAAAAG-GATGAAGGAGAAAAACCTATATTGTCTAAATATA------GGTAACACAAAA-CGATCTCAAAAATGACGACCTGAATCTCGATTTCTATTTTTTT--ATAAACAAAATCGAAATGATGTGAATCAATTCGAA-----GTTTAAGAAAtaatattcattGATCAAATGATTCTCTTCATAGTCTGATAGATCCGTGGTGGAActtattaaTCGGACGAGAATAAAGATAGAGTCCCATTTTAC-ATGTCAATACTGACAACAATGAAATTTATAGTAAGATGXXXGCTCTACT-CCCCAAAAAGGTCTGTTTGACACCTTACCTtttt-gtag-TTATTATCCA-TTTGAATTATTTAGAATCTATATCAGTTTTCATTTTCAAACTTAGAAAGTCTTCTTTTATTTATAAGATCCAAAAAATTCCCGGTCCAAAACTTTTTTAATTTACTACtttt------gagttTCTTTTCATTGACAGAGACCCAAGTCATATATTAAAATGATACTGATacttc--------------------------------------cgtaaTGGTCGGCATAGCTTAATTGCGGAAGACTGAAAATCCTTGTGTCACCATT--------------------------------------------------------------------------------------------AGTAAAATGATAA-TGATACTTCAGTAGATGATACCTCAGTAATGGTGGACATAGC-------TTTTTTG--CAGAGGACTGAAAATC------CTTGTGTCACCATTCGTAAAATGAGGATGATACTTCGGTAATGGCCGGGATAGCTCAgttg

AY257781 AATTGGNTTGAGCCTTGGTATGGNAACCTACTAAGTGATAACTTTCAAATTCAGAGAAACCCTGGAATTAAcaatgggcAATCCTGAGCCAAATCCTGGTTTATGTGAACAAACCAGAGTTTAGAAAGCGAGAAAAAA-GGGATAGGTGCAGAGACTCAATGGAAGCTGTTCTAACAAATGGAGTTCACAACCTTGTGTTGATAAAGGAATCCTTCGATCAAAACTTCAAATAAAAAAG-GATGAAGGAGAAAAACCTATATTGTCTAAATATA------GGTAACACAAAA-CGATCTCAAAAATGACGACCTGAATCTCGATTTCTATTTTTTT--ATAAACAAAATCGAAATGATGTGAATCAATTCGAA-----GTTTAAGAAAtaatattcattGATCAAATGATTCTCTTCATAGTCTGATAGATCCGTGGTGGAActtattaaTCGGACGAGAATAAAGATAGAGTCCCATTTTAC-ATGTCAATACTGACAACAATGAAATTTATAGTAAGATGXXXGCTCTACT-CCCCAAAAAGGTCTGTTTGACACCTTACCTtttt-gtag-TTATTATCCA-TTTGAATTATTTAGAATCTATATCAGTTTTCATTTTCAAACTTAGAAAGTCTTCTTTTATTTATAAGATCCAAAAAATTCCCGGTCCAAAACTTTTTTAATTTACTACtttt------gagttTCTTTTCATTGACAGAGACCCAAGTCATATATTAAAATGATACTGATacttc--------------------------------------cgtaaTGGTCGGCATAGCTTAATTGCGGAGGACTGAAAATCCTTGTGTCACCATT--------------------------------------------------------------------------------------------AGTAAAATGATAA-TGATACTTCAGTAGATGATACCTCAGTAATGGTGGACATAGC-------TTTTTTG--CAGAGGACTGAAAATC------CTTGTGTCACCATTCGTAAAATGAGGATGATACTTCGGTAATGGCCGGGATAGCTCAgttg

AY257782 NNNNNNNNNNAGCCTTGGTATGGNAACCTACTAAGTGATAACTTTCAAATTCAGAGAAACCCTGGAATTAAcaatgggcAATCCTGAGCCAAATCCTGGTTTATGTGAACAAACCGGAGTTTAGAAAGCGAGAAAAAA-GGGATAGGTGCAGAGACTCAATGGAAGCTGTTCTAACAAATGGAGTTCACAACCTTGTGTTGATAAAGGAATCCTTCGAT-----CTTCAAATAAAAAAG-GATGAAGGAGAAAAACCTATATTGTCTAAATATA------GGTAACACAAAA-CGATCTCAAAAATGACGACCTGAATCTCGATTTCTATTTTTTT--ATAAACAAAATCGAAATGATGTGAATCAATTCGAA-----GTTTAAGAAAtaatattcattGATCAAATGATTCTGTTCATAGTCTGATAGATCCGTGGTGGAActtattaaTCGGACGAGAATAAAGATAGAGTCCCATTTTAC-ATGTCAATACTGACAACAATGAAATTTATAGTAAGATGXXXGCTCTACT-CCCCAAAAAGGTCTGTTTGACACCTTACCTtttt-gtag-TTATTATCCA-TTTGAATTATTTAGAATCTATATCAGTTTTCATTTTCAAACTTAGAAAGTCTTCTTTTATTTATAAGATCCAAAAAATTCCCGGTCCAAAACTTTTTTAATTTACTACtttt------gagttTCTTTTCATTGACAGAGACCCAAGTCATATATTAAAATGATACTGATacttc--------------------------------------cgtaaTGGTCGGCATAGCTTAATTGCGGAGGACTGAAAATCCTTGTGTCACCATT--------------------------------------------------------------------------------------------AGTAAAATGATAA-TGATACTTCAGTAGATGATACCTCAGTAATGGTGGACATAGC-------TTTTTTG--CAGAGGACTGAAAATC------CTTGTGTCACCATTCGTAAAATGAGGATGATACTTCGGTAATGGCCGGGATAGCTCAgttg

AY257783 AATTGGATTGAGCCTTGGTATGGAAACCTACTAAGTGATAACTTTCAAATTCAGAGAAACCCTGGAATTAAcaatgggcAATCCTGAGCCAAATCCTGGTTTATGTGAACAAACCGGAGTTTAGAAAGCGAGAAAAAA-GGGATAGGTGCAGAGACTCAATGGAAGCTGTTCTAACAAATGGAGTTCACAACCTTGTGTTGATAAAGGAATCCTTCGATCAAAACTTCAAATAAAAAAG-GATGAAGGAGAAAAACCTATATTGTCTAAATATA------GGTAACACAAAA-CGATCTCAAAAATGACGACCTGAATCTCGATTTCTATTTTTTT--ATAAACAAAATCGAAATGATGTGAATCAATTCGAA-----GTTTAAGAAAtaatattcattGATCAAATGATTCTCTTCATAGTCTGATAGATCCGTGGTGAAActtattaaTCGGACGAGAATAAAGATAGAGTCCCATTTTAC-ATGTCAATACTGACAACAATGAAATTTATAGTAAGATGXXXGCTCTACT-CCCCAAAAAGGTCTGTTTGACACCTTACCTtttt-gtag-TTATTATCCA-TTTGAATTATTTAGAATCTATATCAGTTTTCATTTTCAAACTTAGAAAGTCTTCTTTTATTTATAAGATCCAAAAAATTCCCGGTCCAAAACTTTTTTAATTTACTACtttt------gagttTCTTTTCATTGACAGAGACCCAAGTCATATATTAAAATGATACTGATacttc--------------------------------------cgtaaTGGTCGGCATAGCTTAATTGCGGAGGACTGAAAATCCTTGTGTCACCATT--------------------------------------------------------------------------------------------AGTAAAATGATAA-TGATACTTCAGTAGATGATACCTCAGTAATGGTGGACATAGC-------TTTTTTG--CAGAGGACTGAAAATC------CTTGTGTCACCATTCGTAAAATGAGGATGATACTTCGGTAATGGCCGGGATAGCTCAgttg

AY257784 AATTGGATTGAGCCTTGGTATGGAAACCTACTAAGTGATAACTTTCAAATTCAGAGAAACCCTGGAATTAAcaatgggcAATCCTGAGCCAAATCCTGGTTTATGTGAACAAACCGGAGTTTAGAAAGCGAGAAAAAA-GGGATAGGTGCAGAGACTCAATGGAAGCTGTTCTAACAAATGGAGTTCACAACCTTGTGTTGATAAAGGAATCCTTCGATCAAAACTTCAAATAAAAAAG-GATGAAGGAGAAAAACCTATATTGTCTAAATATA------GGTAACACAAAA-CGATCTCAAAAATGACGACCTGAATCTCGATTTCTATTTTTTT--ATAAACAAAATCGAAATGATGTGAATCAATTCGAA-----GTTTAAGAAAtaatattcattGATCAAATGATTCTCTTCATAGTCTGATAGATCCGTGGTGGAActtattaaTCGGACGAGAATAAAGATAGAGTCCCATTTTAC-ATGTCAATACTGACAACAATGAAATTTATAGTAAGATGXXXGCTCTACT-CCCCAAAAAGGTCTGTTTGACACCTTACCTtttt-gtag-TTATTATCCA-TTTGAATTATTTAGAATCTATATCAGTTTTCATTTTCAAACTTAGAAAGTCTTCTTTTATTTATAAGATCCAAAAAATTCCCGGTCCAAAACTTTTTTAATTTACTACtttt------gagttTCTTTTCATTGACAGAGACCCAAGTCATATATTAAAATGATACTGATacttc--------------------------------------cgtaaTGGTCGGCATAGCTTAATTGCGGAGGACTGAAAATCCTTGTGTCACCATT--------------------------------------------------------------------------------------------AGTAAAATGATAA-TGATACTTCAGTAGATGATACCTCAGTAATGGTGGACATAGC-------TTTTTTG--CAGAGGACTTAAAATC------CTTGTGTCACCATTCGTAAAATGAGGATGATACTTCGGTAATGGCCGGGATAGCTCAgttg

AY257785 AATTGGATTGAGCCTTGGTATGGAAACCTACTAAGTGATAACTTTCAAATTCAGAGAAACCCTGGAATTAAcaatgggcAATCCTGAGCCAAATCCTGGTTTATGTGAACAAACCGGAGTTTAGAAAGCGAGAAAAAA-GGGATAGGTGCAGAGACTCAATGGAAGCTGTTCTAACAAATGGAGTTCACAACCTTGTGTTGAGAAAGGAATCCTTCGATCAAAACTTCAAATAAAAAAG-GATGAAGGAGAAAAACCTATATTGTCTAAATATA------GGTAACACAAAA-CGATCTCAAAAATGACGACCTGAATCTCGATTTCTATTTTTTT--ATAAACAAAATCGAAATGATGTGAATCAATTCGAA-----GTTTAAGAAAtaatattcattGATCAAATGATTCTCTTCATAGTCTGATAGATCCGTGGTGGAACTTCTTAATCGGACGAGAATAAAGATAGAGTCCCATTTTAC-ATGTCAATACTGACAACAATGAAATTTATAGTAAGATGXXXGCTCTACT-CCCCAAAAAGGTCTGTTTGACACCTTACCTtttt-gtag-TTATTATCCA-TTTGAATTATTTAGAATCTATATCAGTTTTCATTTTCAAACTTAGAAAGTCTTCTTTTATTTATAAGATCCAAAAAATTCCCGGTCCAAAACTTTTTTAATTTACTACtttt------gagttTCTTTTCATTGACAGAGACCCAAGTCATATATTAAAATGATACTGATacttc--------------------------------------cgtaaTGGTCGGCATAGCTTAATTGCGGAGGACTGAAAATCCTTGTGTCACCATT--------------------------------------------------------------------------------------------AGTAAAATGATAA-TGATACTTCAGTAGATGATACCTCAGTAATGGTGGACATAGC-------TTTTTTG--CAGAGGACTGAAAATC------CTTGTGTCACCATTCGTAAAATGAGGATGATACTTCGGTAATGGCCGGGATAGCTCAgttg

AY257786 AATTGGATTGAGCCTTGGTATGGWAACCTACTAAGTGATAACTTTCAAATTCAGAGAAACCCTGGAATTAAcaatgggcAATCCTGAGCCAAATCCTGGTTTATGTGAACAAACCGGAGTTTAGAAAGCGAGAAAAAA-GGGATAGGTGCAGAGACTCAATGGAAGCTGTTCTAACAAATGGAGTTCACAACCTTGTGTTGATAAAGGAATCCTTCGATCAAAACTTCAAATAAAAAAG-GATGAAGGAGAAAAACCTATATTGTCTAAATATA------GGTAACACAAAA-CGATCTCAAAAATGACGACCTGAATCTCGATTTCTATTTTTTT--ATAAACAAAATCGAAATGATGTGAATCAATTCGAA-----GTTTAAGAAAtaatattcattGATCAAATGATTCTCTTCATAGTCTGATAGATCCGTGGTGGAActtattaaTCGGACGAGAATAAAGATAGAGTCCCATTTTAC-ATGTCAATACTGACAACAATGAAATTTATAGTAAGATGXXXGCTCTACT-CCCCAAAAAGGTCTGTTTGACACCTTACCTtttt-gtag-TTATTATCCA-TTTGAATTATTTAGAATCTATATCAGTTTTCATTTTCAAACTTAGAAAGTCTTCTTTTATTTATAAGATCCAAAAAATTCCCGGTCCAAAACTTTTTTAATTTACTACtttt------gagttTCTTTTCATTGACAGAGACCCAAGTCATATATTAAAATGATACTGATacttc--------------------------------------cgtaaTGGTCGGCATAGCTTAATTGCGGAGGACTGAAAATCCTTGTGTCACCATT--------------------------------------------------------------------------------------------AGTAAAATGATAA-TGATACTTCAGTAGATGATACCTCAGTAATGGTGGACATAGC-------TTTTTTG--CAGAGGACTTAAAATC------CTTGTGTCACCATTCGTAAAATGAGGATGATACTTCGGTAAGGGCCGGGATAGCTCAgttg

AY257787 AATTGGATTGAGCCTTGGTATGGAAACCTACTAAGTGATAACTTTCAAATTCAGAGAAACCCTGGAATTAAcaatgggcAATCCTGAGCCAAATCCTGGTTTATGTGAACAAACCGGAGTTTAGAAAGCGAGAAAAAA-GGGATAGGTGCAGAGACTCAATGGAAGCTGTTCTAACAAATGGAGTTCACAACCTTGTGTTGATAAAGGAATCCTTCGATCAAAACTTCAAATAAAAAAG-GATGAAGGAGAAAAACCTATATTGTCTAAATATA------GGTAACACAAAA-CGATCTCAAAAATGACGACCTGAATCTCGATTTCGATTTTTTT--ATAAACAAAATCGAAATGATGTGAATCAATTCGAA-----GTTTAAGAAAtaatattcattGATCAAATGATTCTCTTCATAGTCTGATAGATCCGTGGTGGAActtattaaTCGGACGAGAATAAAGATAGAGTCCCATTTTAC-ATGTCAATACTGACAACAATGAAATTTATAGTAAGATGXXXGCTCTACT-CCCCAAAAAGGTCTGTTTGACACCTTACCTtttt-gtag-TTATTATCCA-TTTGAATTATTTAGAATCTATATCAGTTTTCATTTTCAAACTTAGAAAGTCTTCTTTTATTTATAAGATCCAAAAAATTCCCGGTCCAAAACTTTTTTAATTTACTACtttt------gagttTCTTTTCATTGACAGAGACCCAAGTCATATATTAAAATGATACTGATacttc--------------------------------------cgtaaTGGTCGGCATAGCTTAATTGCGGAGGACTGAAAATCCTTGTGTCACCATT--------------------------------------------------------------------------------------------AGTAAAATGATAA-TGATACTTCAGTAGATGATACCTCAGTAATGGTGGACATAGC-------TTTTTTG--CAGAGGACTGAAAATC------CTTGTGTCACCATTCGTAAAATGAGGATGATACTTCGGTAATGGCCGGGATAGCTCAgttg

AY257788 AATTGGATTGAGCCTTGGTATGGAAACCTACTAAGTGATAACTTTCAAATTCAGAGAAACCCTGGAATTAAcaatgggcAATCCTGAGCCAAATCCTGGTTTATGTGAACAAACCGGAGTTTAGAAAGCGAGAAAAAA-GGGATAGGTGCAGAGACTCAATGGAAGCTGTTCTAACAAATGGAGTTCACAACCTTGTGTTGATAAAGGAATCCTTCGATCAAAACTTCAAATAAAAAAG-GATGAAGGAGAAAAACCTATATTGTCTAAATATA------GGTAACACAAAA-CGATCTCAAAAATAACGACCTGAATCTCGATTTCTATTTTTTT--ATAAACAAAATCGAAATGATGTGAATCAATTCGAA-----GTTTAAGAAAtaatattcattGATCAAATGATTCTCTTCATAGTCTGATAGATCCGTGGTGGAActtattaaTCGGACGAGAATAAAGATAGAGTCCCATTTTAC-ATGTCAATACTGACAACCATGAAATTTATAGTAAGATGXXXGCTCTACT-CCCCAAAAAGGTCTGTTTGACACCTTACCTtttt-gtag-TTATTATCCA-TTTGAATTATTTAGAATCTATATCAGTTTTCATTTTCAAACTTAGAAAGTCTTCTTTTATTTATAAGATCCAAAAAATTCCCGGTCCAAAACTTTTTTAATTTACTACtttt------gagttTCTTTTCATTGACAGAGACCCAAGTCATATATTAAAATGATACTGATacttc--------------------------------------cgtaaTGGTCGGCATAGCTTAATTGCGGAGGACTGAAAATCCTTGTGTCACCATT--------------------------------------------------------------------------------------------AGTAAAATGATAA-TGATACTTCAGTAGATGATACCTCAGTAATGGTGGACATAGC-------TTTTTTG--CAGAGGACTGAAAATC------CTTGTGTCACCATTCGTAAAATGAGGATGATACTTCGGTAATGGCCGGGATAGCTCAgttg

AY257789 AATTGGATTGAGCCTTGGTATGGAAACCTACTAAGTGATAACTTTCAAATTCAGAGAAACCCTGGAATTAAcaatgggcAATCCTGAGCCAAATCCTGGTTTATGTGAACAAACCGGAGTTTAGAAAGCGAGAAAAAA-GGGATAGGTGCAGAGACTCAATGGAAGCTGTTCTAACAAATGGAGTTCACAACCTTGTGTTGATAAAGGAATCCTTCGATCAAAACTTCAAATAAAAAAG-GATGAAGGATAAAAACCTATATTGTCTAAATATA------GGTAACACAAAA-CGATCTCAAAAATGACGACCTGAATCTCGATTTCTATTTTTTT--ATAAACAAAATCGAAATGATGTGAATCAATTCGAA-----GTTTAAGAAAtaatattcattGATCAAATGATTCTCTTCATAGTCTGATAGATCCGTGGTGGAActtattaaTCGGACGAGAATAAAGATAGAGTCCCATTTTAC-ATGTCAATACTGACAACAATGAAATTTATAGTAAGATGXXXGCTCTACT-CCCCAAAAAGGTCTGTTTGACACCTTATCTtttt-gtag-TTATTATCCA-TTTGAATTATTTAGAATCTATATCAGTTTTCATTTTCAAACTTAGAAAGTCTTCTTTTATTTATAAGATCCAAAAAATTCCCGGTCCAAAACTTTTTTAATTTACTACtttt------gagttTCTTTTCATTGACAGAGACCCAAGTCATATATTAAAATGATACTGATacttc--------------------------------------cgtaaTGGTCGGCATAGCTTAATTGCGGAGGACTGAAAATCCTTGTGTCACCATT--------------------------------------------------------------------------------------------AGTAAAATGATAA-TGATACTTCAGTAGATGATACCTCAGTAATGGTGGACATAGC-------TTTTTTG--CAGAGGACTGAAAATC------CTTGTGTCACCATTCGTAAAATGAGGATGATACTTCGGTAATGGCCGGGATAGCTCAgttg

AY257790 AATTGGATTGAGCCTTGGTATGGAAACCTACTAAGTGATAACTTTCAAATTCAGAGAAACCCTGGAATTAAcaatgggcAATCCTGAGCCAAATCCTGGTTTATGTGAACAAACCGGAGTTTAGAAAGCGAGAAAAAA-GGGATAGGTGCAGAGACTCAATGGAAGCTGTTCTAACAAATGGAGTTCACAACCTTGTGTTGATAAAGGAATCCTTCGATCAAAACTTCAAATAAAAAAG-GATGAAGGAGAAAAACCTATATTGTCTAAATATA------GGTAACACAAAA-CGATCTCAAAAATGACGACCTGAATCTCGATTTCTATTTTTTT--ATAAACAAAATCGAAATGATGTGAATCAATTCGAA-----GTTTAAGAAAtaatattcattGATCAAATGATTCTCTTCATAGTCTGATAGATCCGTGGTGGAActtattaaTCGGACGAGAATAAAGATAGAGTCCCATTTTAC-ATGTCAATACTGACAACAATGAAATTTATAGTAAGATGXXXGCTCTACT-CCCCAAAAAGGTCTGTTTGACACCTTACCTtttt-gtag-TTATTATCCA-TTTGAATTATTTAGAATCTATATCAGTTTTCATTTTCAAACTTAGAAAGTCTTCTTTTATTTATAAGATCCAAAAAATTCCCGGTCCAAAACTTTTTTAATTTACTACtttt------gagttTCTTTTCATTGACAGAGACCCAAGTCATATATTAAAATGATACTGATacttc--------------------------------------cgtaaTGGTCGGCATAGCTTAATTGCGGAGGACTGAAAATCCTTGTGTCACCATT--------------------------------------------------------------------------------------------AGTAAAATGATAA-TGATACTTCAGTAGATGATACCTCAGTAATGGTGGACATAGC-------TTTTTTG--CAGAGGACTGAAAATC------CTTGTGTCCTCATTCGTAAAATGAGGATGATACTTCGGTAATGGCCGGGATAGCTCAgttg

AY257791 AATTGGATTGAGCCTTGGTATGGAAACCTACTAAGTGATAACTTTCAAATTCAGAGAAACCCTGGAATTAAcaatgggcAATCCTGAGCCAAATCCTGGTTTATGTGAACAAACCGGAGTTTAGAAAGCGAGAAAAAA-GGGATAGGTGCAGAGACTCAATGGAAGCTGTTCTAACAAATGGAGTTCACAACCTTGTGTTGATAAAGGAATCCTTCGATCAAAACTTCAAATAAAAAAG-GATGAAGGAGAAAAACCTATATTGTCTAAATATA------GGTAACACAAAA-CGATCTCAAAAATGACGACCTGAATCTCGATTTCTATTTTTTT--ATAAACAAAATCGAAATGATGTGAATCAATTCGAA-----GTTTAAGAAAtaatattcattGATCAAATGATTCTCTTCATAGTCTGATAGATCCGTGGTGGAActtattaaTCGGACGAGAATAAAGATAGAGTCCCATTTTAC-ATGTCAATACTGACAACAATGAAATTTATAGTAAGATGXXXGCTCTACT-CCCCAAAAAGGTCTGTTTGACACCTTACCTtttt-gtag-TTATTATCCA-TTTGAATTATTTAGAATCTATATCAGTTTTCATTTTCAAACTTAGAAAGTCTTCTTTTATTTATAAGATCCAAAAAATTCCCGGTCCAAAACTTTTTTAATTTACTACtttt------gagttTCTTTTCATTGACAGAGACCCAAGTCATATATTAAAATGATACTGATacttc--------------------------------------cgtaaTGGTCGGCATAGCTTAATTGCGGAGGACTGAAAATCCTTGTGTCACCATTAGTAAAATGATAATGATACTTCAGTAGATGATACCTCAGTAATGGTGGACATAGCTTTTTTGCAGAGGACTGAAAATCCTTGTGTCACCATTAGTAAAATGATAA-TGATACTTCAGTAGATGATACCTCAGTAATGGTGGACATAGC-------TTTTTTG--CAGAGGACTTAAAATC------CTTGTGTCACCATTCGTAAAATGAGGATGATACTTCGGTAATGGCCGGGATAGCTCAGTTn

AY257792 AATTGGATTGAGCCTTGGTATGGAAACCTACTAAGTGATAACTTTCAAATTCAGAGAAACCCTGGAATTAAcaatgggcAATCCTGAGCCAAATCCTGGTTTATGTGAACAAACCGGAGTTTAGAAAGCGAGAAAAAA-GGGATAGGTGCAGAGACTCAATGGAAGCTGTTCTAACAAATGGAGTTCACAACCTTGTGTTGATAAAGGAATCCTTCGATCAAAACTTCAAATAAAAAAG-GATGAAGGAGAAAAACCTATATTGTCTAAATATA------GGTAACACAAAA-CGATCTCAAAAATGACGACCTGAATCTCGATTTCTATTTTTTT--ATAAACAAAATCGAAATGATGTGAATCAATTCGAA-----GTTTAAGAAAtaatattcattGATCAAATGATTCTCTTCATAGTCTGATAGATCCGTGGTGGAActtattaaTCGGACGAGAATAAAGATAGAGTCCCATTTTAC-ATGTCAATACTGACAACAATGAAATTTATAGTAAGATGXXXGCTCTACT-CCCCAAAAAGGTCTGTTTGACACCTTACCTtttt-gtag-TTATTATCCA-TTTGAATTATTTAGAATCTATATCAGTTTTCATTTTCAAACTTAGAAAGTCTTCTTTTATTTATAAGATCCAAAAAATTCCCGGTCCAAAACTTTTTTAATTTACTACTTTT------AAGTTTCTTTTCATTGACAGAGACCCAAGTCATATATTAAAATGATACTGATacttc--------------------------------------cgtaaTGGTCGGCATAGCTTAATTGCGGAGGACTGAAAATCCTTGTGTCACCATT--------------------------------------------------------------------------------------------------------------------------------------------------------------------------------------------------------CGTAAAATGAGGATGATACTTCGGTAATGGCCGGGATAGCTCAgttg

AY257793 AATTGGATTGAGCCTTGGTATGGAAACCTACTAAGTGATAACTTTCAAATTCAGAGAAACCCTGGAATTAAcaatgggcAATCCTGAGCCAAATCCTGGTTTATGTGAACAAACCGGAGTTTAGAAAGCGAGAAAAAA-GGGATAGGTGCAGAGACTCAATGGAAGCTGTTCTAACAAATGGAGTTCACAACCTTGTGTTGATAAAGGAATCCTTCGATCAAAACTTAAAATAAAAAAG-GATGAAGGAGAAAAACCTATATTGTCTAAATATA------GGTAACACAAAA-CGATCTCAAAAATGACGACCTGAATCTCGATTTCTATTTTTTT--ATAAACAAAATCGAAATGATGTGAATCAATTCGAA-----GTTTAAGAAAtaatattcattGATCAAATGATTCTCTTCATAGTCTGATAGATCCGTGGTGGAActtattaaTCGGACGAGAATAAAGATAGAGTCCCATTTTAC-ATGTCAATACTGACAACAATGAAATTTATAGTAAGATGXXXGCTCTACT-CCCCAAAAAGGTCTGTTTGACACCTTACCTtttt-gtag-TTATTATCCA-TTTGAATTATTTAGAATCTATATCAGTTTTCATTTTCAAACTTAGAAAGTCTTCTTTTATTTATAAGATCCAAAAAATTCCCGGTCCAAAACTTTTTTAATTTACTACtttt------gagttTCTTTTCATTGACAGAGACCCAAGTCATATATTAAAATGATACTGATacttc--------------------------------------cgtaaTGGTCGGCATAGCTTAATTGCGGAGGACTGAAAATCCTTGTGTCACCATT--------------------------------------------------------------------------------------------------------------------------------------------------------------------------------------------------------CGTAAAATGAGGATGATACTTCGGTAATGGCCGGGATAGCTCAgttg

AY257794 AATTGGATTGAGCCTTGGTATGGAAACCTACTAAGTGATAACTTTCAAATTCAGAGAAACCCTGGAATTAAcaatgggcAATCCTGAGCCAAATCCTGGTTTATGTGAACAAACCGGAGTTTAGAAAGCGAGAAAAAA-GGGATAGGTGCAGAGACTCAATGGAAGCTGTTCTAACAAATGGAGTTCACAACCTTGTGTTGATAAAGGAATCCTTCGATCAAAACTTCAAATAAAAAAG-GATGAAGGAGAAAAACCTATATTGTCTAAATATA------GGTAACACAAAA-CGATCTCAAAAATGACGACCTGAATCTCGATTTCTATTTTTTT--ATAAACAAAATCGAAATGATGTGAATCAATTCGAA-----GTTTAAGAAAtaatattcattGATCAAATGATTCTCTTCATAGTCTGATAGATCCGTGGTGGAActtattaaTCGGACGAGAATAAAGATAGAGTCCCATTTTAC-ATGTCAATACTGACAACAATGAAATTTATAGTAAGATGXXXGCTCTACT-CCCCAAAAAGGTCTGTTTGACACCTTACCTtttt-gtag-TTATTATCCA-TTTGAATTATTTAGAATCTATATCAGTTTTCATTTTCAAACTTAGAAAGTCTTCTTTTATTTATAAGATCCAAAAAATTCCCGGTCCAAAACTTTTTTAATTTACTACtttt------gagttTCTTTTCATTGACAGAGACCCAAGTCATATATTAAAATGATACTGATacttc--------------------------------------cgtaaTGGTCGGCATAGCTTAATTGCGGAGGACTGAAAATCCTTGTGTCACCATT--------------------------------------------------------------------------------------------------------------------------------------------------------------------------------------------------------CGTAAAATGAGGATGATACTTCGGTAATGGCCGGGATAGCTCAgttg

DQ013052 AATTGGATTGAGCCTTGGTATGGAAACCTACTAAGTGATAACTTTCAAATTCAGAGAAACCCTGGAATTAAcaatgggcAATCCTGAGCCAAATCCTGGTTTATGTGAACAAACCGGAGTTTARAAAGCGAGAAAAAA-GGGATAGGTGCAGAGACTCAATGGAAGCTGTTCTAACAAATGGAGTTCACTACCTTGTGTTGATAAAGGAATCCTTCGATCAAAACTTCAAAGAAAAAAG-GATGAAGGAGAAAAACCTATATTGTCTAAATATA------GGTAACACAAAA-CGATCTCAAAAATGACGACCTGAATCTCGATTTCTATTTTTTT--ATAAACAAAATCGAAATGATGTGAATCAATTCGAA-----GTTTAAGAAAtaatattcattGATCAAATGATTCTCTTCATAGTCTGATAGATCCGTGGTGGAActtattaaTCGGACGAGAATAAAGATAGAGTCCCATTTTAC-ATGTCAATACTGACNACAAYGAAAKTNATAGTAAGATGXXXGCTCTACT-CCCCAAAAAGGTCTGTTTGACACCTTACCTtttt-gtag-TTATTATCCA-TTTGAATTATTTAGAATCTACATCAGTTTTCATTTTCAAACTTAGAAAGTCTTCTTTTATTTATAAGATCCAAAAAATTCCCGGTCCAAAACTTTTTTAATTTACTACtttt------gagttTCTTTTCATTGACAGAGACCCAAGTCATATATTAAAATGATACTGATACTTC----------------------------------------------------------------------------------------------------------------------------------------------------------------------------------------------------------------AGTAGATGATACCTCAGTAATGGTGGACATAGC-------TTTTTTG--CAGAGGACTGAAAATC------CTTGTGTCACCATTCGTAAAATGAGGATGATACTTCGGTAATGGCCGGGATAGCTCAgttg

DQ013053 AATTGGATTGAGCCTTGGTATGGAAACCTACTAAGTGATAACTTTCAAATTCAGAGAAACCCTGGAATTAAcaatgggcAATCCTGAGCCAAATCCTGGTTTATGTGAACAAACCGGAGTTTAGAAAGCGAGAAAAAA-GGGATAGGTGCAGAGACTCAATGGAAGCTGTTCTAACAAATGGAGTTCACTACCTTGTGTTGATAAAGGAATCCTTCGATCAAAACTTCGAATAAAAAAG-GATGAAGGAGAAAAACCTATATTGTCTAAATATA------GGTAACACAAAA-CGATCTCAAAAATGACGACCTGAATCTCGATTTCTATTTTTTT--ATAAACAAAATCGAAATGATGTGAATCAATTCGAA-----GTTTAAGAAAtaatattcattGATCAAATGATTCTCTTCATAGTCTGATAGATCCGTGGTGGAActtattaaTCGGACGAGAATAAAGATAGAGTCCCATTTTAC-ATGTCAATACTGACAACAATGAAATTTATAGTAAGATGXXXGCTCTACT-CCCCAAAAAGGTCTGTTTGACACCTTACCTtttt-gtag-TTATTATCCA-TTTGAATTATTTAGAATCTACATCAGTTTTCATTTTCANACTTAGAAAGTCTTCTTTTATTTATAAGATCCAAAAAATTCCCGGTCCAAAACTTTTTTAATTTACTACtttt------gagttTCTTTTCATTGACAGAGACCCAAGTCATATATTAAAATGATACTGATacttc--------------------------------------cgtaaTGGTCGGCATAGCTTAATTGCGGAGGACTGAAAATCCTTGTGTCACCATT--------------------------------------------------------------------------------------------AGTAAAATGATAA-TGATACTTCAGTAGATGATACCTCAGTAATGGTGGACATAGC-------TTTTTTG--CAGAGGACTGAAAATC------CTTGTGTCACCATTCGTAAAATGAGGATGATACCTCGGTAATGGCCGGGATAGCTCAgttg

DQ013054 AATTGGATTGAGCCTTGGTATGGAAACCTACTAAGTGATAACTTTCAAATTCAGAGAAACCCTGGAATTAAcaatgggcAATCCTGAGCCAAATCCTGGTTTATGTGAACAAACCGGAGTTTAGAAAGCGAGARAAAA-GGGATAGGTGCAGAGACTCAATGGAAGCTGYTCTAACAAATGGAGTTCMCAACCTTGTGTKGATAAAGSAATCCTTCGATCAAAACTTCRAATAAAMAAG-GATGAAGGAGAAAAACCTATATTGTCTAAATATA------GGTMACRCAANA-CGATCTCAAAAATGACGACCTGAATCTCGATTTCTATTTTTTT--ATRAACAAAATCGAAATGATGTGARTCAATTCGAA-----GTKTRAGAAAtaatattcattGATCAAATGATTCTCTTCATAGTCTGATAGATCCGTGGTGGAActtattaaTCGGACGAGAATAAAGATAGAGTCCCATTYTAC-ATGTCAATACTGACAACAATGAAATTTATAGTAAGATGXXXGCTCTACT-CCCCAAAAAGGTCTGTTTGACACCTTACCTtttt-gtag-TTATTATCCA-TTTGAATTATTTAGAATCTATATCAGTTTTCATTTTCAAACTTAGAAAGTCTTCTTTTATTTATAAGATCCAAAAAATTCCCGGTCCAAAACTTTTTTAATTTACTACtttt------gagttTCTTTTCATTGACAGAGACCCAAGTCATATATTAAAATGATACTGATacttc--------------------------------------cgtaaTGGTCGGCATAGCTTAATTGCGGAGGACTGAAAATCCTTGTGTCACCATT--------------------------------------------------------------------------------------------AGTAAAATGATAA-TGATACTTCAGTAGATGATACCTCAGTAATGGTGGACATAGC-------TTTTTTG--CAGAGGACTGAAAATC------CTTGTGTCACCATTCGTAAAATGAGGATGATACTTCGGTAATGGCCGGGATAGCTCAgttg

DQ013055 AATTGGATTGAGCCTTGGTATGGAAACCTACTAAGTGATAACTTTCAAATTCAGAGAAACCCTGGAATTAAcaatgggcAATCCTGAGCCAAATCCTGGTTTATGTGAACAAACCGGAGTTTAGAAAGCGAGAAAAAA-GGGATAGGTGCAGAGACTCAATGGAAGCTGTTCTAACAAATGGAGTTCACAACCTTGTGTTGATAAAGGAATCCTTCGATCAAAACTTCAAATAAAAAAG-GATGAAGGAGAAAAACCTATATTGTCTAAATATA------GGTAACACAAAA-CGATCTCAAAAATGACGACCTGAATCTCGATTTCTATTTTTTT--ATAAACAAAATCGAAATGATGTGAATCAATTCGAA-----GTTTAAGAAAtaatattcattGATCAAATGATTCTCTTCATAGTCTGATAGATCCGTGGTGGAActtattaaTCGGACGAGAATAAAGATAGAGTCCCATTTTAC-ATGTCAATACTGACAACAATGAAATTTATAGTAAGATGXXXGCTCTACT-CCCCAAAAAGGTCTGTTTGACACCTTACCTtttt-gtag-TTATTATCCA-TTTGAATTATTTAGAATCTATATCAGTTTTCATTTTCAAACTTAGAAAGTCTTCTTTTATTTATAAGATCCAAAAAATTCCCGGTCCAAAACTTTTTTAATTTACTACtttt------gagttTCTTTTCATTGACAGAGACCCAAGTCATATATTAAAATGATACTGATacttc--------------------------------------cgtaaTGGTCGGCATAGCTTAATTGCGGAGGACTGAAAATCCTTGTGTCACCATTAGTAAAATGATAATGATACTTCAGTAGATGATACCTCAGTAATGGTGGACATAGCTTTTTTGCAGAGGACTGAAAATCCTTGTGTCACCATTAGTAAAATGATAA-TGATACTTCAGTAGATGATACCTCAGTAATGGTGGACATAGC-------TTTTTTG--CAGAGGACTTAAAATC------CTTGTGTCACCATTCGTAAAATGAGGATGATACTTCGGTAATGGCCGGGATAGCTCAgttg

DQ013056 AATTGGATTGAGCCTTGGTATGGAAACCTACTAAGTGATAACTTTCAAATTCAGAGAAACCCTGGAATTAAcaatgggcAATCCTGAGCCAAATCCTGGTTTATGTGAACAAACCGGAGTTTAGAAAGCGAGAAAAAA-GGGATAGGTGCAGAGACTCAATGGAAGCTGTTCTAACAAATGGAGTTCACAACCTTGTGTTGATAAAGGAATCCTTCGATCAAAACTTCAAATAAAAAAG-GATGAAGGAGAAAAACCTATATTGTCTAAATATA------GGTAACACAAAA-CGATCTCAAAAATGACGACCTGAATCTCGATTTCTATTTTTTT--ATAAACAAAATCGAAATGATGTGAATCAATTCGAA-----GTTTAAGAAAtaatattcattGATCAAATGATTCTCTTCATAGTCTGATAGATCCGTGGTGGAActtattaaTCGGACGAGAATAAAGATAGAGTCCCATTTTAC-ATGTCAATACTGACAACAATGAAATTTATAGTAAGATGXXXGCTCTACT-CCCCAAAAAGGTCTGTTTGACACCTTACCTtttt-gtag-TTATTATCCA-TTTGAATTATTTAGAATCTATATCAGTTTTCATTTTCAAACTTATAAAGTCTTCTTTTATTTATAAGATCCAAAAAATTCCCGGTCCAAAACTTTTTTAATTTACTACtttt------gagttTCTTTTCATTGACAGAGACCCAAGTCATATATTAAAATGATACTGATacttc--------------------------------------cgtaaTGGTCGGCATAGCTTAATTGCGGAGGACTGAAAATCCTTGTGTCACCATT--------------------------------------------------------------------------------------------AGTAAAATGATAA-TGATACTTCAGTAGATGATACCTCAGTAATGGTGGACATAGC-------TTTTTTG--CAGAGGACTGAAAATC------CTTGTGTCACCATTCGTAAAATGAGGATGATACTTCGGTAATGGCCGGGATAGCTCAgttg

DQ013057 AATTGGATTGAGCCTTGGTATGGAAACCTACTAAGTGATAACTTTCAAATTCAGAGAAACCCTGGAATTAAcaatgggcAATCCTGAGCCAAATCCTGGTTTATGTGAACAAACCGGAGTTTAGAAAGCGAGAAAAAA-GGGATAGGTGCAGAGACTCAATGGAAGCTGTTCTAACAAATGGAGTTCACAACCTTGTGTTGATAAAGGAATCCTTCGATCAAAACTTCAAATAAAAAAG-GATGAAGGAGAAAAACCTATATTGTCTAAATATA------GGTAACACAAAA-CGATCTCAAAAATGACGACCTGAATCTCGATTTCTATTTTTTT--ATAAACAAAATCGAAATGATGTGAATCAATTCGAA-----GTTTAAGAAAtaatattcattGATCAAATGATTCTCTTCATAGTCTGATAGATCCGTGGTGGAACTTCTTAATCGGACGAGAATAAAGATAGAGTCCCATTTTAC-ATGTCAATACTGACAACAATGAAATTTATAGTAAGATGXXXGCTCTACT-CCCCAAAAAGGTCTGTTTGACACCTTACCTtttt-gtag-TTATTATCCA-TTTGAATTATTTAGAATCTATATCAGTTTTCATTTTCAAACTTAGAAAGTCTTCTTTTATTTATAAGATCCAAAAAATTCCCGGTCCAAAACTTTTTTAATTTACTACtttt------gagttTCTTTTCATTGACAGAGACCCAAGTCATATATTAAAATGATACTGATacttc--------------------------------------cgtaaTGGTCGGCATAGCTTAATTGCGGAGGACTGAAAATCCTTGTGTCACCATT--------------------------------------------------------------------------------------------AGTAAAATGATAA-TGATACTTCAGTAGATGATACCTCAGTAATGGTGGACATAGC-------TTTTTTG--CAGAGGACTGAAAATC------CTTGTGTCACCATTCGTAAAATGAGGATGATACTTCGGTAATGGCCGGGATAGCTCAgttg

DQ013058 AATTGGATTGAGCCTTGGTATGGAAACCTACTAAGTGATAACTTTCAAATTCAGAGAAACCCTGGAATTAAcaatgggcAATCCTGAGCCAAATCCTGGTTTATGTGAACAAACCGGAGTTTAGAAAGCGAGAAAAAA-GGGATAGGTGCAGAGACTCAATGGAAGCTGTTCTAACAAATGGAGTTCACAACCTTGTGTTGATAAAGGAATCCTTCGATCAAAACTTCAAATAAAAAAG-GATGAAGGAGAAAAACCTATATTGTCTAAATATA------GGTAACACAAAA-CGATCTCAAAAATGACGACCTGAATCTCGATTTCTATTTTTTT--ATAAACAAAATCGAAATGATGTGAATCAATTCGAA-----GTTTAAGAAAtaatattcattGATCAAATGATTCTCTTCATAGTCTGATAGATCCGTGGTGGAACTTCTTAATCGGACGAGAATAAAGATAGAGTCCCATTTTAC-ATGTCAATACTGACAACAATGAAATTTATAGTAAGATGXXXGCTCTACT-CCCCAAAAAGGTCTGTTTGACACCTTACCTtttt-gtag-TTATTATCCA-TTTGAATTATTTAGAATCTATATCAGTTTTCATTTTCAAACTTAGAAAGTCTTCTTTTATTTATAAGATCCAAAAAATTCCCGGTCCAAAACTTTTTTAATTTACTACtttt------gagttTCTTTTCATTGACAGAGACCCAAGTCATATATTAAAATGATACTGATacttc--------------------------------------cgtaaTGGTCGGCATAGCTTAATTGCGGAGGACTGAAAATCCTTGTGTCACCATT--------------------------------------------------------------------------------------------AGTAAAATGATAA-TGATACTTCAGTAGATGATACCTCAGTAATGGTGGACATAGC-------TTTTTTG--CAGAGGACTGAAAATC------CTTGTGTCACCATTCGTAAAATGAGGATGANACCTCGGTAATGGCCGGGATAGCTCAgttg

DQ013059 AATTGGATTGAGCCTTGGTATGGAAACCTACTAAGTGATAACTTTCAAATTCAGAGAAACCCTGGAATTAAcaatgggcAATCCTGAGCCAAATCCTGGTTTATGTGAACAAACCGGAGTTTAGAAAGCGAGAAAAAA-GGGATAGGTGCAGAGACTCAATGGAAGCTGTTCTAACAAATGGAGGTCACAACCTTGTGTTGATAAAGGAATCCTTCGATCAAAACTTCAAATAAAAAAG-GATGAAGGAGAAAAACCTATATTGTCTAAATATA------GGTAACACAAAA-CGATCTCAAAAATGACGACCTGAATCTCGATTTCTATTTTTTT--ATAAACAAAATCGAAATGATGTGAATCAATTCGAA-----GTTTAAGAAAtaatattcattGATCAAATGATTCTCTTCATAGTCTGATAGATCCGTGGTGGAActtattaaTCGGACGAGAATAAAGATAGAGTCCCATTTTAC-ATGTCAATACTGACAATAATGAAATTTATAGTAAGATGXXXGCTCTACT-CCCCAAAAAGGTCTGTTTGACACCTTACCTtttt-gtag-TTATTATCCA-TTTGAATTATTTAGAATCTATATCAGTTTTCATTTTCAAACTTAGAAAGTCTTCTTTTATTTATAAGATCCAAAAAATTCCCGGTCCAAAACTTTTTTAATTTACTACtttt------gagttTCTTTTCATTGACAGAGACCCAAGTCATATATTAAAATGATACTGATacttc--------------------------------------cgtaaTGGTCGGCATAGCTTAATTGCGGAGGACTGAAAATCCTTGTGTCACCATT--------------------------------------------------------------------------------------------AGTAAAATGATAA-TGATACTTCAGTAGATGATACCTCAGTAATGGTGGACATAGC-------TTTTTTG--CAGAGGACTGAAAATC------CTTGTGTCACCATTCGTAAAATGAGGATGATACTTCGGTAATGGCCGGGATAGCTCAgttg

DQ013060 AATTGGATTGAGCCTTGGTATGGAAACCTACTAAGTGATAACTTTCAAATTCAGAGAAACCCTGGAATTAAcaatgggcAATCCTGAGCCAAATCCTGGTTTATGTGAACAAACCGGAGTTTAGAAAGCGAGAAAAAA-GGGATAGGTGCAGAGACTCAATGGAAGCTGTTCTAACAAATGGAGTTCACAACCTTGTGTTGATAAAGGAATCCTTCGATCAAAACTTCAAATAAAAAAG-GATGAAGGAGAAAAACCTATATTGTCTAAATATA------GGTAACACAAAA-CGATCTCAAAAATGACGACCTGAATCTCGATTTCTATTTTTTT--ATAAACAAAATCGAAATGATGTGAATCAATTCGAA-----GTTTAAGAAAtaatattcattGATCAAATGATTCTCTTCATAGTCTGATAGATCCGTGGTGGAActtattaaTCGGACGAGAATAAAGATAGAGTCCCATTTTAC-ATGTCAATACTGACAACAATGAAATTTATAGTAAGATGXXXGCTCTACT-CCCCAAAAAGGTCTGTTTGACACCTTACCTtttt-gtag-TTATTATCCA-TTTGAATTATTTAGAATCTATATCAGTTTTCATTTTCAAACTTAGAAAGTCTTCTTTTATTTATAAGATCAAAAAAATTCCCGGTCCAAAACTTTTTTAATTTACTACtttt------gagttTCTTTTCATTGACAGAGACCCAAGTCATATATTAAAATGATACTGATacttc--------------------------------------cgtaaTGGTCGGCATAGCTTAATTGCGGAGGACTGAAAATCCTTGTGTCACCATT--------------------------------------------------------------------------------------------AGTAAAATGATAA-TGATACTTCAGTAGATGATACCTCAGTAATGGTGGACATAGC-------TTTTTTG--CAGAGGACTGAAAATC------CTTGTGTCACCATTAGTAAAATGAGGATGATACTTCGGTAATGGCCGGGATAGCTCAgttg

trnLF_DW AATTGGATTGAGCCTTGGTATGGAAACCTACTAAGTGATAACTTTCAAATTCAGAGAAACCCTGGAATTAAcaatgggcAATCCTGAGCCAAATCCTGGTTTATGTGAACAAACCGGAGTTTAGAAAGCGAGAAAAAA-GGGATAGGTGCAGAGACTCAATGGAAGCTGTTCTAACAAATGGAGTTCACAACCTTGTGTTGATAAAGGAATCCTTCGATCAAAACTTCAAATAAAAAAG-GATGAAGGAGAAAAACCTATATTGTCTAAATATA------GGTAACACAAAA-CGATCTCAAAAATGACGACCTGAATCTCGATTTCTATTTTTTT--ATAAACAAAATCGAAATGATGTGAATCAATTCGAA-----GTTTAAGAAAtaatattcattGATCAAATGATTCTCTTCATAGTCTGATAGATCCGTGGTGGAActtattaaTCGGACGAGAATAAAGATAGAGTCCCATTTTAC-ATGTCAATACTGACAACAATGAAATTTATAGTAAGATGXXXGCTCTACT-CCCCAAAAAGGTCTGTTTGACACCTTACCTtttt-ttag-TTATTATCGA-TTTGAATTATTTAGAATCTATATAAGTTTTCATTTTCAAACTTAGAAAGTCTTCTTTTATTTATAAGATCCAAGAAATTCCCGGTCCAAAACTTTTTTAATTTACTACtttt------gagttTCTTTTCATTGACAGAGACCCAAGTCATATATTAAAATGATACTGATacttc--------------------------------------cgtaaTGGTCGGCATAGCTTAATTGCGGAGGACTGAAAATCCTTGTGTCACCATT--------------------------------------------------------------------------------------------------------------------------------------------------------------------------------------------------------CGTAAAATGAGGATGATACTTCGGTAATGGCCGGGATAGCTCAgttg

trnLF_DY AATTGGATTGAGCCTTGGTATGGAAACCTACTAAGTGATAACTTTCAAATTCAGAGAAACCCTGGAATTAAcaatgggcAATCCTGAGCCAAATCCTGGTTTACGTGAACAAACCGGAGTTTAGAAAGCGAGAAAAAA-GGGATAGGTGCAGAGACTCAATGGAAGCTGTTCTAACAAATGGAGTTCACTACCTTGTGTTGATAAAGGAATCCTTCGATCAAAACTTCAAATCAAAAAG-GATGAAGGAGAAAAACCTATATTGTCTAAATATA------GGTAACACAAAA-CGATCTCAAAAATGACGACCTGAATCTCGATTTCTATTTTTTT--ATAAACAAAATCGAAATGATGTGAATCAATTCGAA-----GTTTAAGAAAtaatattcattGATCAAATGATTCTCTTCATAGTCTGATAGATCCGTGGTGGAActtattaaTCGGACGAGAATAAAGATAGAGTCCCATTTTAC-ATGTCAATACTGACAACAATGAAATTTATAGTAAGATGXXXGCTCTACT-CCCCAAAAAGGTCTGTTTGACACCTTACCTtttt-gtag-TTATTATCCA-TTTGAATTATTTAGAATCTATATCAGTTTTCATTTTCAAACTTAGAAAGTCTTCTTTTATTTATAAGATCCAAAAAATTCCCGGTCCAAAACTTTTTTAATTTACTACtttt------gagttTCTTTTCATTGACAGAGACCCAAGTCATATATTAAAATGATACTGATacttc--------------------------------------cgtaaTGGTCGGCATAGCTTAATTGCGGAGGACTGAAAATCCTTGTGTCACCATT--------------------------------------------------------------------------------------------AGTAAAATGATAA-TGATACTTCAGTAGATGATACCTCAGTAATGGTGGACATAGC-------TTTTTTG--CAGAGGACTGAAAATC------CTTGTGTCACCATTCGTAAAATGAGGATGATACTTCGGTAATGGCCGGGATAGCTCAgttg

trnLF_DZ AATTGGATTGAGCCTTGGTATGGAAACCTACTAAGTGATAACTTTCAAATTCAGAGAAACCCTGGAATTAAcaatgggcAATCCTGAGCCAAATCCTGGTTTATGTGAACAAACCGGAGTTTAGAAAGCGAGAAAAAA-GGGATAGGTGCAGAGACTCAATGGAAGCTGTTCTAACAAATGGAGTTCACTACCTTGTGTTGATAAAGGAATCCTTCGATCAAAACTTCAAATAAAAAAG-GATGAAGGAGAAAAACCTATATTGTCTAAATATA------GGTAACACAAAA-CGATCTCAAAAATGACGACCTGAATCTCGATTTCTATTTTTTT--ATAAAAAAAATCGAAATGATGTGAATCAATTCGAA-----GTTTAAGAAAtaatattcattGATCAAATGATTCTCTTCATAGTCTGATAGATCCGTGGTGGAActtattaaTCGGACGAGAATAAAGATAGAGTCCCATTTTAC-ATGTCAATACTGACAACAATGAAATTTATAGTAAGATGXXXGCTCTACT-CCCCAAAAAGGTCTGTTTGACACCTTACCTtttt-gtag-TTATTATCCA-TTTGAATTATTTAGAATCTACATCAGTTTTCATTTTCAAACTTAGAAAGTCTTCTTTTATTTATAAGATCCAAAAAATTCCCGGTCCAAAACTTTTTTAATTTACTACtttt------gagttTCTTTTCATTGACAGAGACCCAAGTCATATATTAAAATGATACTGATacttc--------------------------------------cgtaaTGGTCGGCATAGCTTAATTGCGGAGGACTGAAAATCCTTGTGTCACCATT--------------------------------------------------------------------------------------------ATTAAAATGATAA-TGATACTTCAGTAGATGATACCTCAGTAATGGTGGACATAGC------TTTTTTT---CAGAGGACTGAAAATC------CTTGTGTCACCATTCGTAAAATGAGGATGATACTTCGGTAATGGCCGGGATAGCTCAgttg

trnLF_EH AATTGGATTGAGCCTTGGTATGGAAACCTACTAAGTGATAACTTTCAAATTCAGAGAAACCCTGGAATTAAcaatgggcAATCCTGAGCCAAATCCTGGTTTATGTGAACAAACCGGAGTTTAGAAAGCGAGAAAAAA-GGGATAGGTGCAGAGACTCAATGGAAGCTGTTCTAACAAATGGAGTTCACTACCTTGTGTTGATAAAGGAATCCTTCGATCAAAACTTCAAATAAAAAAG-GATGAAGGAGAAAAACCTATATTGTCTAAATATA------GGTAACACAAAA-CGATCTCAAAAATGACGACCTGAATCTCGATTTCGATTTTTTT--ATAAACAAAATCGAAATGATGTGAATCAATTCGAA-----GTTTAAGAAAtaatattcattGATCAAATGATTCTCTTCATAGTCTGATAGATCCGTGGTGGAActtattaaTCGGACGAGAATAAAGATAGAGTCCCATTTTAC-ATGTCAATACTGACAACAATGAAATTTATAGTAAGATGXXXGCTCTACT-CCCCAAAAAGGTCTGTTTGACACCTTACCTtttt-gtag-TTATTATCCA-TTTGAATTATTTAGAATCTATATCAGTTTTCATTTTCAAACTTAGAAAGTCTTCTTTTATTTATAAGATCCAAAAAATTCCCGGTCCAAAACTTTTTTAATTTACTACtttt------gagttTCTTTTCATTGACAGAGACCCAAGTCATATATTAAAATGATACTGATacttc--------------------------------------cgtaaTGGTCGGCATAGCTTAATTGCGGAGGACTGAAAATCCTTGTGTCACCATT--------------------------------------------------------------------------------------------AGTAAAATGATAA-TGATACTTCAGTAGATGATACCTCAGTAATGGTGGACATAGC-------TTTTTTG--CAGAGGACTGAAAATC------CTTGTGTCACCATTCGTAAAATGAGGATGATACTTCGGTAATGGCCGGGATAGCTCAgttg

trnLF_EQ AATTGGATTGAGCCTTGGTATGGAAACCTACTAAGTGATAACTTTCAAATTCAGAGAAACCCTGGAATTAAcaatgggcAATCCTGAGCCAAATCCTGGTTTATGTGAACAAACCGGAGTTTAGAAAGCGAGAAAAAA-GGGATAGGTGCAGAGACTCAATGGAAGCTGTTCTAACAAATGGAGTTCACAACCTTGTGTTGATAAAGGAATCCTTCGATCAAAACTTCAAATAAAAAAG-GATGAAGGAGAAAAACCTATATTGTCTAAATATA------GGTAACACAAAA-CGATCTCAAAAATGACGACCTGAATCTCGATTTCTATTTTTTT--ATAAACAAAATCGAAATGATGTGAATCAATTCGAA-----GTTTAAGAAAtaatattcattGATCAAATGATTCTCTTCATAGTCTGATAGATCCGTGGTGGAActtattaaTCGGACGAGAATAAAGATAGAGTCCCATTTTAC-ATGTCAATACTGACAACAATGAAATTTATAGTAAGATGXXXGCTCTACT-CCCCAAAAAGGTCTGTTTGACACCTTACCTtttt-gtag-TTATTATCCA-TTTGAATTATTTAGAATCTATATCAGTTTTCATTTTCAAACTTAGAAAGTCTTCTTTTATTTATAAGATCCAAAAAATTCCCGGTCCAAAACTTTTTTAATTTACTACTTTT-GTTTTGAGTTTCTTTTCATTGACAGAGACCCAAGTCATATATTAAAATGATACTGATacttc--------------------------------------cgtaaTGGTCGGCATAGCTTAATTGCGGAGGACTGAAAATCCTTGTGTCACCATT--------------------------------------------------------------------------------------------ATTAAAATGATAA-TGATACTTCAGTAGATGATACCTCAGTAATGGTGGACATAGC-------TTTTTTG--CAGAGGACTGAAAATC------CTTGTGTCACCATTCGTAAAATGAGGATGATACTTCGGTAATGGCCGGGATAGCTCAgttg

trnLF_ER AATTGGATTGAGCCTTGGTATGGAAACCTACTAAGTGATAACTTTCAAATTCAGAGAAACCCTGGAATTAAcaatgggcAATCCTGAGCCAAATCCTGGTTTATGTGAACAAACCGGAGTTTAGAAAGCGAGAAAAAA-GGGATAGGTGCAGAGACTCAATGGAAGCTGTTCTAACAAATGGAGTTCACTACCTTGTGTTGATAAAGGAATCCTTCGATCAAAACTTCAAAGAAAAAAG-GATGAAGGAGAAAAACCTATATTGTCTAAATATA------GGTAACACAAAA-CGATCTCAAAAATGACGACCTGAATCTCGATTTCTATTTTTTT--ATAAACAAAATCGAAATGATGTGAATCAATTCGAA-----GTTTAAGAAAtaatattcattGATCAAATGATTCTCTTCATAGTCTGATAGATCCGTGGTGGAActtattaaTCGGACGAGAATAAAGATAGAGTCCCATTTTAC-ATGTCAATACTGACAACAATGAAATTTATAGTAAGATGXXXGCTCTACT-CCCCAAAAAGGTCTGTTTGACACCTTACCTtttt-gtag-TTATTATCCA-TTTGAATTATTTAGAATCTACATCAGTTTTCATTTTCAAACTTAGAAAGTCTTCTTTTATTTATAAGATCCAAAAAATTCCCGGTCCAAAACTTTTTTAATTTACTACtttt------gagttTCTTTTCATTGACAGAGACCCAAGTCATATATTAAAATGATACTGATacttc--------------------------------------cgtaaTGGTCGGCATAGCTTAATTGCGGAGGACTGAAAATCCTTGTGTCACCATT--------------------------------------------------------------------------------------------AGTAAAATGATAA-TGATACTTCAGTAGATGATACCTCAGTAATGGTGGACATAGC-------TTTTTTG--CAGAGGACTGAAAATC------CTTGTGTCACCATTCGTAAAATGAGGATGATACTTCGGTAATGGCCGGGATAGCTCAgttg

trnLF_ES AATTGGATTGAGCCTTGGTATGGAAACCTACTAAGTGATAACTTTCAAATTCAGAGAAACCCTGGAATTAAcaatgggcAATCCTGAGCCAAATCCTGGTTTATGTGAACAAACCGGAGTTTAGAAAGCGAGAAAAAA-GGGATAGGTGCAGAGACTCAATGGAAGCTGTTCTAACAAATGGAGTTCACAACCTTGTGTTGATAAAGGAATCCTTCAATCAAAACTTCAAATAAAAAAG-GATGAAGGAGAAAAACCTATATTGTCTAAATATAAATATAGGTAACACAAAA-CGATCTCAAAAATGACGACCTGAATCTCGATTTCTATTTTTTT--ATAAACAAAATCGAAATGATGTGAATCAATTCGAA-----GTTTAAGAAAtaatattcattGATCAAATGATTCTCTTCATAGTCTGATAGATCCGTGGTGGAActtattaaTCGGACGAGAATAAAGATAGAGTCCCATTTTAC-ATGTCAATACTGACAACAATGAAATTTATAGTAAGATGXXXGCTCTACT-CCCCAAAAAGGTCTGTTTGACACCTTACCTtttt-gtag-TTATTATCCA-TTTGAATTATTTAGAATCTATATCAGTTTTCATTTTCAAACTTAGAAAGTCTTCTTTTATTTATAAGATCCAAAAAATTCCCGGTCCAAAACTTTTTTAATTTACTACtttt------gagttTCTTTTCATTGACAGAGACCCAAGTCATATATTAAAATGATACTGATacttc--------------------------------------cgtaaTGGTCGGCATAGCTTAATTGCGGAGGACTGAAAATCCTTGTGTCACCATT--------------------------------------------------------------------------------------------------------------------------------------------------------------------------------------------------------CGTAAAATGAGGATGATACTTCGGTAATGGCCGGGATAGCTCAgttg

trnLF_ET AATTGGATTGAGCCTTGGTATGGAAACCTACTAAGTGATAACTTTCAAATTCAGAGAAACCCTGGAATTAAcaatgggcAATCCTGAGCCAAATCCTGGTTTATGTGAACAAACCGGAGTTTAGAAAGCGAGAAAAAA-GGGATAGGTGCAGAGACTCAATGGAAGCTGTTCTAACAAATGGAGTTCACAACCTTGTGTTGATAAAGGAATCCTTCGATCAAAACTTCAAATAAAAAAG-GATGAAGGAGAAAAACCTATATTGTCTAAATATA------GGTAACACAAAA-CGATCTCAAAAATGACGACCTGAATCTCGATTTCTATTTTTTT--ATAAACAAAATCGAAATGATGTGAATCAATTTGAA-----GTTTAAGAAAtaatattcattGATCAAATGATTCTCTTCATAGTCTGATAGATCCGTGGTGGAActtattaaTCGGACGAGAATAAAGATAGAGTCCCATTTTAC-ATGTCAATACTGACAACAATGAAATTTATAGTAAGATGXXXGCTCTACT-CCCCAAAAAGGTCTGTTTGACACCTTACCTtttt-gtag-TTATTATCCA-TTTGAATTATTTAGAATCTATATCAGTTTTCATTTTCAAACTTAGAAAGTCTTCTTTTATTTATAAGATCCCAAAAATTCCCGGTCCAAAACTTTTTTAATTTACTACtttt------gagttTCTTTTCATTGACAGAGACCCAAGTCATATATTAAAATGATACTGATacttc--------------------------------------cgtaaTGGTCGGCATAGCTTAATTGCGGAGGACTGAAAATCCTTGTGTCACCATT--------------------------------------------------------------------------------------------------------------------------------------------------------------------------------------------------------CGTAAAATGAGGATGATACTTCGGTAATGGCCGGGATAGCTCAgttg

trnLF_EU AATTGGATTGAGCCTTGGTATGGAAACCTACTAAGTGATAACTTTCAAATTCAGAGAAACCCTGGAATTAACAATGGGCAATCCTGAGCCAAATCCTGGTTTATGTGAACAAACCGGAGTTTAGAAAGCGAGAAAAAA-GGGATAGGTGCAGAGACTCAATGGAAGCTGTTCTAACAAATGGAGTTCACAACCTTGTGTTGATAAAGGAATCCTTCGATCAAAACTTCAAATAAAAAAG-GATGAAGGAGAAAAACCTATATTGTCTAAATATA------GGTAACACAAAA-CGATCTCAAAAATGACGACCTGAATCTCGATTTCTATTTTTTT--ATAAACAAAATCGAAATGATGTGAATCAATTCGAA-----GTTTAAGAAATAATATTCATTGATCAAATGATTCTCTTCATAGTCTGATAGATCCGTGGTGGAACTTATTAATCGGACGAGAATAAAGATAGAGTCCCATTTTAC-ATGTCAATACTGACAACAATGAAATTTATAGTAAGATGXXXGCTCTACT-CCCCAAAAAGGTCTGTTTGACACCTTACCTTTTT-GTAG-TTATTATCCA-TTTGAATTATTTAGAATCTATATCAGTTTTCATTTTCAAACTTATAAAGTCTTCTTTTATTTATAAGATCCAAAAAATTCCCGGTCCAAAACTTTTTTAATTTACTACTTTT------GAGTTTCTTTTCATTGACAGAGACCCAAGTCATATATTAAAATGATACTGATACTTC--------------------------------------CGTAATGGTCGGCATAGCTTAATTGCGGAGGACTGAAAATCCTTGTGTCACCATT--------------------------------------------------------------------------------------------AGTAAAATGATAA-TGATACTTCAGTAGATGATACCTCAGTAATGGTGGACATAGC-------TTTTTTG--CAGAGGACTGAAAATC------CTTGTGTCACCATTCGTAAAATGAGGATGATACTTCGGTAATGGCCGGGATAGCTCAgttg

trnLF_EV AATTGGATTGAGCCTTGGTATGGAAACCTACCAAGTGATAACTTTCAAATTCAGAGAAACCCTGGAATTAAcaatgggcAATCCTGAGCCAAATCCTGGTTTATGTGAACAAACCGGAGTTTAGAAAGCGAGAAAAAA-GGGATAGGTGCAGAGACTCAATGGAAGCTGTTCTAACAAATGGAGTTCACAACCTTGTGTTGATAAAGGAATCCTTCGATCAAAACTTCAAATAAAAAAG-GATGAAGGAGAAAAACCTATATTGTCTAAATATA------GGTAACACAAAA-CGATCTCAAAAATGACGACCTGAATCTCGATTTCTATTTTTTT--ATAAACAAAATCGAAATGATGTGAATCAATTCGAA-----GTTTAAGAAAtaatattcattGATCAAATGATTCTCTTCATAGTCTGATAGATCCGTGGTGGAActtattaaTCGGACGAGAATAAAGATAGAGTCCCATTTTAC-ATGTCAATACTGACAACAATGAAATTTATAGTAAGATGXXXGCTCTACT-CCCCAAAAAGGTCTGTTTGACACCTTACCTtttt-gtag-TTATTATCCA-TTTGAATTATTTAGAATCTATATCAGTTTTCATTTTCAAACTTAGAAAGTCTTCTTTTATTTATAAGATCCAAAAAATTCCCCGTCCAAAACTTTTTTAATTTACTACtttt------gagttTCTTTTCATTGACAGAGACCCAAGTCATATATTAAAATGATACTGATacttc--------------------------------------cgtaaTGGTCGGCATAGCTTAATTGCGGAGGACTGAAAATCCTTGTGTCACCATT--------------------------------------------------------------------------------------------AGTAAAATGATAA-TGATACTTCAGTAGATGATACCTCAGTAATGGTGGACATAGC-------TTTTTTG--CAGAGGACTGAAAATC------CTTGTGTCACCATTCGTAAAATGAGGATGATACTNNNNNNNNNNNNNNNNNNNNNNNnnnn

trnLF_EW AATTGGATTGAGCCTTGGTATGGAAACCTACTAAGTGATAACTTTCAAATTCAGAGAAACCCTGGAATTAAcaatgggcAATCCTGAGCCAAATCCTGGTTTATGTGAACAAACCGGAGTTTAGAAAGCGAGAAAAAA-GGGATAGGTGCAGAGACTCAATGGAAGCTGTTCTAACAAATGGAGTTCACAACCTTGTGTTGATAAAGGAATCCTTCGATCAAAACTTCAAATAAAAAAG-GATGAAGGAGAAAAACCTATATTGTCTAAATATA------GGTAACACAAAA-CGATCTCAAAAATGACGACCTGAATCTCGATTTCTATTTTTTT--ATAAACAAAATCGAAATGATGTGAATCAATTCGAA-----GTTTAAGAAAtaatattcattGATCAAATGATTCTCTTCATAGTCTGATAGATCCGTGGTGGAActtattaaTCGGACGAGAATAAAGATAGAGTCCCATTTTAC-ATGTCAATACTGACAACAATGAAATTTATAGTAAGATGXXXGCTCTACT-CCCCAAAAAGGTCTGTTTGACACCTTACCTtttt-gtag-TTATTATCCA-TTTGAATTTTTTAGAATCTATATCAGTTTTCATTTTCAAACTTAGAAAGTCTTCTTTTATTTATAAGATCCAAAAAATTCCCGGTCCAAAACTTTTTTAATTTACTACtttt------gagttTCTTTTCATTGACAGAGACCCAAGTCATATATTAAAATGATACTGATacttc--------------------------------------cgtaaTGGTCGGCATAGCTTAATTGCGGAGGACTGAAAATCCTTGTGTCACCATT--------------------------------------------------------------------------------------------AGTAAAATGATAA-TGATACTTCAGTAGATGATACCTCAGTAATGGTGGACATAGC-------TTTTTTG--CAGAGGACTGAAAATC------CTTGTGTCACCATTCGTAAAATGAGGATGANNNNNNNNNNNNNNNNNNNNNNNNNNNnnnn

trnLF_EX AATTGGATTGAGCCTTGGTATGGAAACCTACTAAGTGATAACTTTCAAATTCAGAGAAACCCTGGAATTAAcaatgggcAATCCTGAGCCAAATCCTGGTTTATGTGAACAAACCGGAGTTTAGAAAGCGAGAAAAAA-GGGATAGGTGCAGAGACTCAATGGAAGCTGTTCTAACAAATGGAGTTCACTACCTTGTGTTGATAAAGGAATCCTTCGATCAAAACTTCAAAGAAAAAAG-GATGAAGGAGAAAAACCTATATTGTCTAAATATA------GGTAACACAAAA-CGATCTCAAAAATGACGACCTGAATCTCGATTTCTATTTTTTT--ATAAACAAAATCGAAATGATGTGAATCAATTCGAA-----GTTTAAGAAAtaatattcattGATCAAATGATTCTCTTCATAGTCTGATAGATCCGTGGTGGAActtattaaTCGGACGAGAATAAAGATAGAGTCCCATTTTAC-ATGTCAATACTGACAACAATGAAATTTATAGTAAGATGXXXGCTCTACT-CCCCAAAAAGGTCTGTTTGACACCTTACCTtttt-gtag-TTATTATCCA-TTTGAATTATTTAGAATCTACATCAGTTTTCATTTTCAAACTTAGAAAGTCTTCTTTTATTTATAAGATCCAAAAAATTCCCGGTCCAAAACTTTTTTAATTTACTACtttt------gagttTCTTTTCATTGACAGAGACCCAAGTCATAT---------------------------------------------------------------------------------------------------------------------------------------------------------------------------------------------------------------ATTAAAATGATAC-TGATACTTCAGTAGATGATACCTCAGTAATGGTGGACATAGC-------TTTTTTG--CAGAGGACTGAAAATC------CTTGTGTCACCATTCGTAAAATGAGGATGATACTTCGGTAATGGCCGGGATAGCTCAgttg

trnLF_EY AATTGGATTGAGCCTTGGTATGGAAACCTACTAAGTGATAACTTTCAAATTCAGAGAAACCCTGGAATTAAcaatgggcAATCCTGAGCCAAATCCTGGTTTATGTGAACAAACCGGAGTTTAGAAAGCGAGAAAAAA-GGGATAGGTGCAGAGACTCAATGGAAGCTGTTCTAACAAATGGAGTTCACAACCTTGTGTTGATAAAGGAATCCTTCGATCAAAACTTCAAATAAAAAAG-GATGAAGGAGAAAAACCTATATTGTCTAAATATA------GGTAACACAAAA-CGATCTCAAAAATGACGACCTGAATCTCGATTTCTATTTTTTT--ATAAACAAAATCGAAATGATGTGAATCAATTCGAA-----GTTTAAGAAAtaatattcattGATCAAATGATTCTGTTCATAGTCTGATAGATCCGTGGTGGAActtattaaTCGGACGAGAATAAAGATAGAGTCCCATTTTAC-ATGTCAATACTGACAACAATGAAATTTATAGTAAGATGXXXGCTCTACT-CCCCAAAAAGGTCTGTTTGACACCTTACCTtttt-gtag-TTATTATCCA-TTTGAATTATTTAGAATCTATATCAGTTTTCATTTTCAAACTTAGAAAGTCTTCTTTTATTTATAAGATCCAAAAAATTCCCGGTCCAAAACTTTTTTAATTTACTACtttt------gagttTCTTTTCATTGACAGAGACCCAAGTCATATATTAAAATGATACTGATacttc--------------------------------------cgtaaTGGTCGGCATAGCTTAATTGCGGAGGACTGAAAATCCTTGTGTCACCATT--------------------------------------------------------------------------------------------AGTAAAATGATAA-TGATACTTCAGTAGATGATACCTCAGTAATGGTGGACATAGC-------TTTTTTG--CAGAGGACTGAAAATC------CTTGTGTCACCATTCGTAAAATGAGGATGATACTNNNNNNNNNNNNNNNNNNNNNNNnnnn

trnLF_FB AATTGGATTGAGCCTTGGTATGGAAACCTACTAAGTGATAACTTTCAAATTCAGAGAAACCCTGGAATTAAcaatgggcAATCCTGAGCCAAATCCTGGTTTATGTGAACAAACCGGAGTTTAGAAAGCGAGAAAAAAAGGGATAGGTGCAGAGACTCAATGGAAGCTGTTCTAACAAATGGAGTTCACAACCTTGTGTTGATAAAGGAATCCTTCGATCAAAACTTCAAATAAAAAAG-GATGAAGGAGAAAAACCTATATTGTCTAAATATA------GGTAACACAAAA-CGATCTCAAAAATGACGACCTGAATCTCGATTTCTATTTTTTT--ATAAACAAAATCGAAATGATGTGAATCAATTCGAA-----GTTTAAGAAAtaatattcattGATCAAATGATTCTCTTCATAGTCTGATAGATCCGTGGTGGAActtattaaTCGGACGAGAATAAAGATAGAGTCCCATTTTAC-ATGTCAATACTGACAACAATGAAATTTATAGTAAGATGXXXGCTCTACT-CCCCAAAAAGGTCTGTTTGACACCTTACCTtttt-gtag-TTATTATCCA-TTTGAATTATTTAGAATCTATATCAGTTTTCATTTTCAAACTTAGAAAGTCTTCTTTTATTTATAAGATCCAAAAAATTCCCGGTCCAAAACTTTTTTAATTTACTACtttt------gagttTCTTTTCATTGACAGAGACCCAAGTCATATATTAAAATGATACTGATacttc--------------------------------------cgtaaTGGTCGGCATAGCTTAATTGCGGAGGACTGAAAATCCTTGTGTCACCATT--------------------------------------------------------------------------------------------AGTAAAATGATAA-TGATACTTCAGTAGATGATACCTCAGTAATGGTGGACATAGC-------TTTTTTG--CAGAGGACTGAAAATC------CTTGTGTCACCATTCGTAAAANNNNNNNNNNNNNNNNNNNNNNNNNNNNNNNNNNNNnnnn

trnLF_FC AATTGGATTGAGCCTTGGTATGGAAACCTACTAAGTGATAACTTTCAAATTCAGAGAAACCCTGGAATTAAcaatgggcAATCCTGAGCCAAATCCTGGTTTATGTGAACAAACCGGAGTTTAGAAAGCGAGAAAAAA-GGGATAGGTGCAGAGACTCAATGGAAGCTGTTCTAACAAATGGAGTTCACAACCTTGTGTTGATAAAGGAATCCTTCGATCAAAACTTCAAATAAAAAAG-GATGAAGGAGAAAAACCTATATTGTCTAAATATA------GGTAACACAAAA-CGATCTCAAAAATGACGACCTGAATCTCGATTTCTATTTTTTT--ATAAACAAAATCGAAATGATGTGAATCAATTCGAA-----GTTTAAGAAAtaatattcattGATCAAATGATTCTCTTCATAGTCTGATAGATCCGTGGTGGAActtattaaTCGGACGAGAATAAAGATAGAGTCCCATTTTAC-ATGTCAATACTGACAACAATGAAATTTAGAGTAAGATGXXXGCTCTACT-CCCCAAAAAGGTCTGTTTGACACCTTACCTtttt-gtag-TTATTATCCA-TTTGAATTATTTAGAATCTATATCAGTTTTCATTTTCAAACTTAGAAAGTCTTCTTTTATTTATAAGATCCAAAAAATTCCCGGTCCAAAACTTTTTTAATTTACTACtttt------gagttTCTTTTCATTGACAGAGACCCAAGTCATATATTAAAATGATACTGATacttc--------------------------------------cgtaaTGGTCGGCATAGCTTAATTGCGGAGGACTGAAAATCCTTGTGTCACCATT--------------------------------------------------------------------------------------------AGTAAAATGATAA-TGATACTTCAGTAGATGATACCTCAGTAATGGTGGACATAGC-------TTTTTTG--CAGAGGACTGAAAATC------CTTGTGTCACCATTCGTAAAATGAGGATGATACNNNNNNNNNNNNNNNNNNNNNNNNnnnn

trnLF_FE AATTGGATTGAGCCTTGGTANGGAAACCTACTAAGTGATAACTTTCAAATTCAGAGAAACCCTGGAATTAAcaatgggcAATCCTGAGCCAAATCCTGGTTTATGTGAACAAACCGGAGTTTAGAAAGCGAGAAAAAA-GGGATAGGTGCAGAGACTCAATGGAAGCTGTTCTAACAAATGGAGTTCACAACCTTGTGTTGATAAAGGAATCCTTCGATCAAAACTTCAAATAAAAAAG-GATGAAGGAGAAAAACCTATATTGTCTAAATATA------GGTAACACAAAA-CGATCTCAAAAATGACGACCTGAATCTCTATTTCTATTTTTTT--ATAAACAAAATCGAAATGATGTGAATCAATTCGAA-----GTTTAAGAAAtaatattcattGATCAAATGATTCTCTTCATAGTCTGATAGATCCGTGGTGGAActtattaaTCGGACGAGAATAAAGATAGAGTCCCATTTTAC-ATGTCAATACTGACAACAATGAAATTTATAGTAAGATGXXXGCTCTACT-CCCCAAAAAGGTCTGTTTGACACCTTACCTtttt-gtag-TTATTATCCA-TTTGAATTATTTAGAATCTATATCAGTTTTCATTTTCAAACTTAGAAAGTCTTCTTTTATTTATAAGATCCAAAAAATTCCCGGTCCAAAACTTTTTTAATTTACTACtttt------gagttTCTTTTCATTGACAGAGACCCAAGTCATATATTAAAATGATACTGATacttc--------------------------------------cgtaaTGGTCGGCATAGCTTAATTGCGGAGGACTGAAAATCCTTGTGTCACCATT--------------------------------------------------------------------------------------------AGTAAAATGATAA-TGATACTTCAGTAGATGATACCTCAGTAATGGTGGACATAGC-------TTTTTTG--CAGAGGACTGAAAATC------CTTGTGTCACCATTCGTAAAATGAGGNNNNNNNNNNNNNNNNNNNNNNNNNNNNNNNnnnn

trnLF_FM AATTGGATTGAGCCTTGGTATGGAAACCTACTAAGTGATAACTTTCAAATTCAGAGAAACCCTGGAATTAAcaatgggcAATCCTGAGCCAAATCCTGGTTTATGTGAACAAACCGGAGTTTAGAAAGCGAGAAAAAA-GGGATAGGTGCAGAGACTCAATGGAAGCTGTTCTAACAAATGGAGTTCACAACCTTGTGTTGATAAAGGAATCCTTCGATCAAAACTTCAAATAAAAAAG-AATGAAGGAGAAAAACCTATATTGTCTAAATATA------GGTAACACAAAA-CGATCTCAAAAATGACGACCTGAATCTCGATTTCTATTTTTTT--ATAAACAAAATCGAAATGATGTGAATCAATTCGAA-----GTTTAAGAAAtaatattcattGATCAAATGATTCTCTTCATAGTCTGATAGATCCGTGGTGGAActtattaaTCGGACGAGAATAAAGATAGAGTCCCATTTTAC-ATGTCAATACTGACAACAATGAAATTTATAGTAAGATGXXXGCTCTACT-CCCCAAAAAGGTCTGTTTGACACCTTACCTtttt-gtag-TTATTATCCA-TTTGAATTATTTAGAATCTATATCAGTTTTCATTTTCAAACTTAGAAAGTCTTCTTTTATTTATAAGATCCAAAAAATTCCCGGTCCAAAACTTTTTTAATTTACTACtttt------gagttTCTTTTCATTGACAGAGACCCAAGTCATATATTAAAATGATACTGATacttc--------------------------------------cgtaaTGGTCGGCATAGCTTAATTGCGTAGGACTGAAAATCCTTGTGTCACCATT--------------------------------------------------------------------------------------------AGTAAAATGATAA-TGATACTTCAGTAGATGATACCTCAGTAATGGTGGACATAGC-------TTTTTTG--CAGAGGACTGAAAATC------CTTGTGTCACCATTCGTAAAATGAGGATGATACTTCGGTAATGGCCGGGATAGCTCAgttg

trnLF_FQ AATTGGATTGAGCCTTGGTATGGAAACCTACTAAGTGATAACTTTCAAATTCAGAGAAACCCTGGAATTAAcaatgggcAATCCTGAGCCAAATCCTGGTTTATGTGAACAAACCGGAGTTTAGAAAGCGAGAAAAAA-GGGATAGGTGCAGAGACTCAATGGAAGCTGTTCTAACAAATGGAGTTCATAACCTTGTGTTGATAAAGGAATCCTTCGATCAAAACTTCAAATAAAAAAG-GATGAAGGAGAAAAACCTATATTGTCTAAATATA------GGTAACACAAAA-CGATCTCAAAAATGACGACCTGAATCTCTATTTCTATTTTTTT--ATAAACAAAATCGAAATGATGTGAATCAATTCGAA-----GTTTAAGAAAtaatattcattGATCAAATGATTCTCTTCATAGTCTGATAGATCCGTGGTGGAActtattaaTCGGACGAGAATAAAGATAGAGTCCCATTTTAC-ATGTCAATACTGACAACAATGAAATTTATAGTAAGATGXXXGCTCTACT-CCCCAAAAAGGTCTGTTTGACACCTTACCTtttt-gtag-TTATTATCCA-TTTGAATTATTTAGAATCTATATCAGTTTTCATTTTCAAACTTAGAAAGTCTTCTTTTATTTATAAGATCCAAAAAATTCCCGGTCCAAAACTTTTTTAATTTACTACtttt------gagttTCTTTTCATTGACAGAGACCCAAGTCATATATTAAAATGATACTGATacttc--------------------------------------cgtaaTGGTCGGCATAGCTTAATTGCGGAGGACTGAAAATCCTTGTGTCACCATT--------------------------------------------------------------------------------------------AGTAAAATGATAA-TGATACTTCAGTAGATGATACCTCAGTAATGGTGGACATAGC-------TTTTTTG--CAGAGGACTGAAAATC------CTTGTGTCACCATTCGTAAAATGAGGATGATNNNNNNNNNNNNNNNNNNNNNNNNNNnnnn

trnLF_FS AATTGGATTGAGCCTTGGTATGGAAACCTACTAAGTGATAACTTTCAAATTCAGAGAAACCCTGGAATTAAcaatgggcAATCCTGAGCCAAATCCTGGTTTATGTGAACAAACCGGAGTTTAGAAAGCGAGAAAAAAAGGGATAGGTGCAGAGACTCAATGGAAGCTGTTCTAACAAATGGAGTTCACAACCTTGTGTTGATAAAGGAATCCTTCGATCAAAACTTCAAATAAAAAAG-GATGAAGGAGAAAAACCTATATTGTCTAAATATA------GGTAACACAAAA-CGATCTCAAAAATGACGACCTGAATCTCGATTTCGATTTTTTT--ATAAACAAAATCGAAATGATGTGAATCAATTCGAA-----GTTTAAGAAAtaatattcattGATCAAATGATTCTCTTCATAGTCTGATAGATCCGTGGTGGAActtattaaTCGGACGAGAATAAAGATAGAGTCCCATTTTAC-ATGTCAATACTGACAACAATGAAATTTATAGTAAGATGXXXGCTCTACT-CCCCAAAAAGGTCTGTTTGACACCTTACCTtttt-gtag-TTATTATCCA-TTTGAATTATTTAGAATCTATATCAGTTTTCATTTTCAAACTTAGAAAGTCTTCTTTTATTTATAAGATCCAAAAAATTCCCGGTCCAAAACTTTTTTAATTTACTACtttt------gagttTCTTTTCATTGACAGAGACCCAAGTCATATATTAAAATGATACTGATacttc--------------------------------------cgtaaTGGTCGGCATAGCTTAATTGCGGAGGACTGAAAATCCTTGTGTCACCATT--------------------------------------------------------------------------------------------AGTAAAATGATAA-TGATACTTCAGTAGATGATACCTCAGTAATGGTGGACATAGC-------TTTTTTG--CAGAGGACTGAAAATC------CTTGTGTCACCATTCGTAAAATGAGGCTGATACTTCGGTAATGGCCGGGATAGCTCAgttg

trnLF_HF AATTGGATTGAGCCTTGGTATGGAAACCTACTAAGTGATAACTTTCAAATTCAGAGAAACCCTGGAATTAAcaatgggcAATCCTGAGCCAAATCCTGGTTTATGTGAACAAACCGGAGTTTAGAAAGCGAGAAAAAA-GGGATAGGTGCAGAGACTCAATGGAAGCTGTTCTAACAAATGGAGTTCACAACCTTGTGTTGATAAAGGAATCCTTCGATCAAAACTTCAAATAAAAAAG-GATGAAGGAGAAAAACCTATATTGTCTAAATATA------GGTAACACAAAA-CGATCTCAAAAATGACGACCTGAATCTCGATTTCTATTTTTTT--ATAAACAAAATCGAAATGATGTGAATCAATTCGAA-----GTTTAAGAAAtaatattcattGATCAAATGATTCTCTTCATAGTCTGATAGATCCGTGGTGGAActtattaaTCGGACGAGAATAAAGATAGAGTCCCATTTTAC-ATGTCAATACTGACAACAATGAAATTTATAGTAAGATGXXXNCTCTACT-CCCCAAAAAGGTCTGTTTGACACCTTACCTTTTT-GTAG-TTATTATCCA-TTTGAATTATTTAGAATCTATATCAGTTTTCATTTTCAAACTTAGAAAGTCTTCTTTTATTTATAAGATCCAAAAAATTCCCGGTCCAAAACTTTTTTAATTTACTACtttt------gagttTCTTTTCATTGACAGAGACCCAAGTCATATAGTAAAATGATACTGATacttc--------------------------------------cgtaaTGGTCGGCATAGCTTAATTGCGGAGGACTGAAAATCCTTGTGTCACCATT--------------------------------------------------------------------------------------------AGTAAAATGATAA-TGATACTTCAGTAGATGATACCTCAGTAATGGTGGACATAGC-------TTTTTTG--CAGAGGACTGAAAATC------CTTGTGTCACCATTCGTAAAATGAGGATGATACTTCGGTAATGGCCGGGATAGCTCAgttg

trnLF_HG NNNNNNNNNNNNNNNNNNNNNNNNNNNNNNNNNNNNNATAACTTTCAAATTCAGAGAAACCCTGGAATTAAcaatgggcAATCCTGAGCCAAATCCTGGTTTATGTGAACAAACCGGAGTTTAGAAAGCGAGAAAAAA-GGGATAGGTGCAGAGACTCAATGGAAGCTGTTCTAACAAATGGAGTTCACAACCTTGTGTTGATAAAGGAATCCTTCGATCAAAACTTCAAATAAAAAAG-GATGAAGGAGAAAAACCTATATTGTCTAAATATA------GGTAACACAAAA-CGATCTCAAAAATGACGACCTGAATCTCGATTTCTATTTTTTT--ATAAACAAAATCGAAATGATGTGAATCAATTCGAA-----GTTTAAGAAAtaatattcattGATCAAATGATTCTCTTCATAGTCTGATAGATCCGTGGTGGAActtattaaTCGGACGAGAATAAAGATAGAGTCCCATTTTAC-ATGTCAATACTGACAACAATGAAATTTATAGTAAGATGXXXGNNNNNNN-NNNNNNNNNNNNNNNNNNNNNNNNNNNNNTTTTT-GTAG-TTATTATCCA-TTTGAATTATTTAGAATCTATATCAGTTTTCATTTTCAAACTTAGAAAGTCTTCTTTTATTTATAAGATCCAAAAAATTCCCGGTCCAAAACTTTTTTAATTTACTACtttt------gagttTCTTTTCATTGACAGAGACCCAAGTCATATATTAAAATGATACTGATacttc--------------------------------------cgtaaTGGTCGGCATAGCTTAATTGCGGAGGACTGAAAATCCTTGTGTCACCATT--------------------------------------------------------------------------------------------ATTAAAATGATAA-TGATACTTCAGTAGATGATACCTCAGTAATGGTGGACATAGC-------TTTTTTG--CAGAGGACTGAAAATC------CTTGTGTCACCATTCGTAAAATGAGGANNNNNNNNNNNNNNNNNNNNNNNNNNNNNNnnnn

trnLF_HH AATTGGATTGAGCCTTGGTATGGAAACCTACTAAGTGATAACTTTCAAATTCAGAGAAACCCTGGAATTAAcaatgggcAATCCTGAGCCAAATCCTGGTTTATGTGAACAAACCGGAGTTTAGAAAGCGAGAAAAAA-GGGATAGGTGCAGAGACTCAATGGAAGCTGTTCTAACAAATGGAGTTCACAACCTTGTGTTGATAAAGGAATCCTTCGATCAAAACTTCAAATAAAAAAG-GATGAAGGAGAAAAACCTATATTGTCTAAATATA------GGTAACACAAAA-CGATCTCAAAAATGACGACCTGAATCTCGATTTCTATTTTTTT--ATAAACAAAATCGAAATGATGTGAATCAATTCGAA-----GTTTAAGAAAtaatattcattGATCAAATGATTCTCTTCATAGTCTGATAGATCCGTGGTGGAActtattaaTCGGACGAGAATAAAGATAGAGTCCCATTTTAC-ATGTCAATACTGACAACAATGAAATTTATAGTAAGATGXXXGCTCTACT-CCCCAAAAAGGTCTGTTTGACACCTTACCTtttt-gtag-TTATTATCCA-TTTGAATTATTTAGAATCTATATCAGTTTTCATTTTCAAACTTAGAAAGTCTTCTTTTATTTATAAGATCCAAAAAATTCCCGGTCCAAAACTTTTTTAATTTACTACtttt------gagttTCTTTTCATTGACAGAGACCCAAGTCATATATTAAAATGATACTGATacttc--------------------------------------cgtaaTGGTCGGCATAGCTTAATTGCGGAGGACTGAAAATCCTTGTGTCACCATT--------------------------------------------------------------------------------------------AGTAAAATGATAA-TGATACTTCAGTAGATTATACCTCAGTAATGGTGGACATAGC-------TTTTTTG--CAGAGGACTGAAAATC------CTTGTGTCACCATTCGTAAAATGAGGATGATACTTCGGTAATGGCCGGGATAGCTCAgttg

trnLF_HI AATTGGATTGAGCCTTGGTATGGAAACCTACTAAGTGATAACTTTCAAATTCAGAGAAACCCTGGAATTAAcaatgggcAATCCTGAGCCAAATCCTGGTTTATGTGAACAAACCGGAGTTTAGAAAGCGAGAAAAAA-GGGATAGGTGCAGAGACTCAATGGAAGCTGTTCTAACAAATGGAGTTCACAACCTTGTGTTGATAAAGGAATCCTTCGATCAAAACTTCAAATAAAAAAG-GATGAAGGAGAAAAACCTATATTGTCTAAATATA------GGTAACACAAAA-CGATCTCAAAAATGACGACCTGAATCTCGATTTCTATTTTTTT--ATAAACAAAATCGAAATGATGTGAATCAATTCGAA-----GTTTAAGAAAtaatattcattGATCAAATGATTCTCTTCATAGTCTGATAGATCCGTGGTGGAActtattaaTCGGACGAGAATAAAGATAGAGTCCCATTTTAC-ATGTCAATACTGACAACAATGAAATTTATAGTAAGATGXXXGCTCTACT-CCCCAAAAAGGTCTGTTTGACACCTTACCTtttt-gtag-TTATTATCCA-TTTGAATTATTTAGAATCTATATCAGTTTTCATTTTCAAACTTAGAAAGTCTTCTTTTATTTATAAGATCCAAAAAATTCCCGGTCCAAAACTTTTTTAATTTACTACtttt------gagttTCTTTTCATTGACAGAGACCCAAGTCATATATTAAAATGATACTGATacttc--------------------------------------cgtaaTGGTCGGCATAGCTTAATTGCGGAGGACTGAAAATCCTTGTGTCACCAAA--------------------------------------------------------------------------------------------ATGATAATGATA-------CTTCAGTAGATGATACCTCAGTAATGGTGGACATAGC-------TTTTTTG--CAGAGGACTGAAAATC------CTTGTGTCACCATTCGTAAAATGAGGATGATACTTCGGTAATGGCCGGGATAGCTCAgttg

trnLF_HK AATTGGATTGAGCCTTGGTATGGAAACCTACTAAGTGATAACTTTCAAATTCAGAGAAACCCTGGAATTAAcaatgggcAATCCTGAGCCAAATCCTGGTTTATGTGAACAAACCGGAGTTTAGAAAGCGAGAAAAAA-GGGATAGGTGCAGAGACTCAATGGAAGCTGTTCTAACAAATGGAGTTCACAACCTTGTGTTGATAAAGGAATCCTTCGATCAAAACTTCAAATAAAAAAG-GATGAAGGAGAAAAACCTATATTGTCTAAATATA------GGTAACACAAAA-CGATCTCAAAAATGACGACCTGAATCTCGATTTCTTTTTTTTT--ATAAACAAAATCGAAATGATGTGAATCAATTCGAA-----GTTTAAGAAAtaatattcattGATCAAATGATTCTCTTCATAGTCTGATAGATCCGTGGTGGAActtattaaTCGGACGAGAATAAAGATAGAGTCCCATTTTAC-ATGTCAATACTGACAACAATGAAATTTATAGTAAGATGXXXGCTCTACT-CCCCAAAAAGGTCTGTTTGACACCTTACCTtttt-gtag-TTATTATCCATTTTGAATTATTTAGAATCTATATCAGTTTTCATTTTCAAACTTAGAAAGTCTTCTTTTATTTATAAGATCAAAAAAATTCCCGGTCCAAAACTTTTTTAATTTACTACtttt------gagttTCTTTTCATTGACAGAGACCCAAGTCATATATTAAAATGATACTGATacttc--------------------------------------cgtaaTGGTCGGCATAGCTTAATTGCGGAGGACTGAAAATCCTTGTGTCACCATT--------------------------------------------------------------------------------------------AGTAAAATGATAA-TGATACTTCAGTAGATGATACCTCAGTAATGGTGGACATAGC-------TTTTTTG--CAGAGGACTGAAAATC------CTTGTGTCACCATTCGTAAAATGAGGATGATACTTCGGTAATGGCCGGGATAGCTCAgttg

trnLF_HV AATTGGATTGAGCCTTGGTATGGAAACCTACTAAGTGATAACTTTCAAATTCAGAGAAACCCTGGAATTAAcaatgggcAATCCTGAGCCAAATCCTGGTTTATGTGAACAAACCGGAGTTTAGAAAGCGAGAAAAAA-GGGATAGGTGCAGAGACTCAATGGAAGCTGTTCTAACAAATGGAGTTCACAACCTTGTGTTGATAAAGGAATCCTTCGATCAAAACTTCAAATAAAAAAG-GATGAAGGAGAAAAACCTATATTGTCTAAATATA------GGTAACACAAAA-CGATCTCAAAAATGACGACCTGAATCTCGATTTCTTTTTTTTT--ATAAACAAAATCGAAATGATGTGAATCAATTCGAA-----GTTTAAGAAAtaatattcattGATCAAATGATTCTCTTCATAGTCTGATAGATCCGTGGTGGAActtattaaTCGGACGAGAATAAAGATAGAGTCCCATTTTAC-ATGTCAATACTGACAACAATGAAATTTATAGTAAGATGXXXGCTCTACT-CCCCAAAAAGGTCTGTTTGACACCTTACCTtttt-gtag-TTATTATCCA-TTTGAATTATTTAGAATCTATATCAGTTTTCATTTTCAAACTTAGAAAGTCTTCTTTTATTTATAAGATCAAAAAAATTCCCGGTCCAAAACTTTTTTAATTTACTACtttt------gagttTCTTTTCATTGACAGAGACCCAAGTCATATATTAAAATGATACTGATacttc--------------------------------------cgtaaTGGTCGGCATAGCTTAATTGCGGAGGACTGAAAATCCTTGTGTCACCATT--------------------------------------------------------------------------------------------AGTAAAATGATAA-TGATACTTCAGTAGATGATACCTCAGTAATGGTGGACATAGC-------TTTTTTG--CAGAGGACTGAAAATC------CTTGTGTCACCATTCGTAAAATGAGGATGATACTTCGGTAATGGCCGGGATAGCTCAgttg

trnLF_HW AATTGGATTGAGCCTTGGTATGGAAACCTACTAAGTGATAACTTTCAAATTCAGAGAAACCCTGGAATTAAcaatgggcAATCCTGAGCCAAATCCTGGTTTACGTGAACAAACCGGAGTTTAGAAAGCGAGAAAAAA-GGGATAGGTGCAGAGACTCAATGGAAGCTGTTCTAACAAATGGAGTTCACTACCTTGTGTTGATAAAGGAATCCTTCGATCAAAACTTCAAATCAAAAAG-GATGAAGGAGAAAAACCTATATTGTCTAAATATA------GGTAACACAAAA-CGATCTCAAAAATGACGACCTGAATCTCGATTTCTATTTTTTT--ATAAACAAAATCGAAATGATGTGAATCAATTCGAA-----GTTTAAGAAAtaatattcattGATCAAATGATTCTCTTCATAGTCTGATAGATCCGTGGTGGAActtattaaTCGGACGAGAATAAAGATAGAGTCCCATTTTAC-ATGTCAATACTGACAACAATGAAATTTATAGTAAGATGXXXNNNNTACT-CCCCAAAAAGGTCTGTTTGACACCTTACCTtttt-gtag-TTATTATCCA-TTTGAATTATTTAGAATCTATATCAGTTTTCATTTTCAAACTTAGAAAGTCTTCTTTTATTTATAAGATCCAAGAAATTCCCGGTCCAAAACTTTTTTAATTTACTGCtttt------gagttTCTTTTCATTGACAGAGACCCAAGTCATATATTAAAATGATACTGATacttc--------------------------------------cgtaaTGGTCGGCATAGCTTAATTGTGGAGGACTGAAAATCCTTGTGTCACCATT--------------------------------------------------------------------------------------------AGTAAAATGATAC-TGATACTTCAGTAGATGATACCTCAGTAATGGTGGACATAGC-------TTTTTTG--CAGAGGACTGAAAATCAAAATCCTTGTGTCACCATTCGTAAAATGAGGATGATACTTCGGTAATGGCCGGGATAGCTCAgttg

trnLF_IF NNNNNNNNNNNGCCTTGGTATGGAAACCTACTAAGTGATAACTTTCAAATTCAGAGAAACCCTGGAATTAAcaatgggcAATCCTGAGCCAAATCCTGGTTTATGTGAACAAACCGGAGTTTAGAAAGCGAGAAAAAA-GGGATAGGTGCAGAGACTCAATGGAAGCTGTTCTAACAAATGGAGTTCATAACCTTGTGTTGATAAAGGAATCCTTCGATCAAAACTTCAAATAAAAAAG-GATGAAGGAGAAAAACCTATATTGTCTAAATATA------GGTAACACAAAA-CGATCTCAAAAATGACGACCTGAATCTCTATTTCTATTTTTTT--ATAAACAAAATCGAAATGATGTGAATCAATTCGAA-----GTTTAAGAAAtaatattcattGATCAAATGATTCTCTTCATAGTCTGATAGATCCGTGGTGGACcttattaaTCGGACGAGAATAAAGATAGAGTCCCATTTTAC-ATGTCAATACTGACAACAATGAAATTTATAGTAAGATGXXXGCTCTACT-CCCCAAAAAGGTCTGTTTGACACCTTACCTtttt-gtag-TTATTATCCA-TTTGAATTATTTAGAATCTATATCAGTTTTCATTTTCAAACTTAGAAAGTCTTCTTTTATTTATAAGATCCAAAAAATTCCCGGTCCAAAACTTTTTTAATTTACTACtttt------gagttTCTTTTCATTGACAGAGACCCAAGTCATATATTAAAATGATACTGATacttc--------------------------------------cgtaaTGGTCGGCATAGCTTAATTGCGGAGGACTGAAAATCCTTGTGTCACCATT--------------------------------------------------------------------------------------------AGTAAAATGATAA-TGATACTTCAGTAGATGATACCTCAGTAATGGTGGACATAGC-------TTTTTTG--CAGAGGACTGAAAATC------CTTGTGTCACCATTCGTAAAATGAGGATGATACTTCGGTAATGGCCGGGATAGCTCAgttg

trnLF_IG AATTGGATTGAGCCTTGGTATGGAAACCTACTAAGTGATAACTTTCAAATTCAGAGAAACCCTGGAATTAAcaatgggcAATCCTGAGCCAAATCCTGGTTTATGTGAACAAACCGGAGTTTAGAAAGCGAGAAAAAA-GGGATAGGTGCAGAGACTCAATGGAAGCTGTTCTAACAAATGGAGTTCACAACCTTGTGTTGATAAAGGAATCCTTCGATCAAAACTTCAAATAAAAAAG-GATGAAGGAGAAAAACCTATATTGTCTAAATATA------GGTAACACAAAA-CGATCTCAAAAATGACGACCTGAATCTCGATTTCTATTTTTTT--ATAAACAAAATCGAAATGATGTGAATCAATTCGAA-----GTTTAAGAAAtaatattcattGATCAAATGATTCTCTTCATAGTCTGATAGATCCGTGGTGGAActtattaaTCGGACGAGAATAAAGATAGAGTCCCATTTTAC-ATGTCAATACTGACAACAATGAAATTTATAGTAAGATGXXXGCTCTACT-CCCCAAAAAGGTCTGTTTGACACCTTACCTtttt-gtag-TTATTATCCA-TTTGAATTATTTAGAATCTATATCAGTTTTCATTTTCAAACTTAGAAAGTCTTCTTTTATTTATAAGATCCAAAAAATTCCCGGTCCAAAACTTTTTTAATTTACTACtttt------gagttTCTTTTCATTGACAGAGACCCAAGTCATATATTAAAATGATACTGATacttc--------------------------------------cgtaaTGGTCGGCATAGCTTAATTGCGGAGGACTGAAAATCCTTGCGTCACCATT--------------------------------------------------------------------------------------------AGTAAAATGATAA-TGATACTTCAGTAGATGATACCTCAGTAATGGTGGACATAGC-------TTTTTTG--CAGAGGACTGAAAATC------CTTGTGTCACCATTCGTAAAATGAGGATGATACTTCGGTAATGGCCGGGATAGCTCAgttg

trnLF_IH AATTGGATTGAGCCTTGGTATGGAAACCTACTAAGTGATAACTTTCAAATTCAGAGAAACCCTGGAATTAAcaatgggcAATCCTGAGCCAAATCCTGGTTTACGTGAACAAACCGGAGTTTAGAAAGCGAGAAAAAA-GGGATAGGTGCAGAGACTCAATGGAAGCTGTTCTAACAAATGGAGTTCACTACCTTGTGTTGATAAAGGAATCCTTCGATCAAAACTTCAAATCAAAAAG-GATGAAGGAGAAAAACCTATATTGTCTAAATATA------GGTAACACAAAA-CGATCTCAAAAATGACGACCTGAATCTCGATTTCTATTTTTTT--ATAAACAAAATCGAAATGATGTGAATCAATTCGAA-----GTTTAAGAAAtaatattcattGATCAAATGATTCTCTTCATAGTCTGATAGATCCGTGGTGGAActtattaaTCGGACGAGAATAAAGATAGAGTCCCATTTTAC-ATGTCAATACTGACAACAATGAAATTTATAGTAAGATGXXXGCTCTACT-CCCCAAAAAGGTCTGTTTGACACCTTACCTtttt-gtag-TTATTATCCA-TTTGAATTATTTAGAATCTATATCAGTTTTCATTTTCAAACTTAGAAAGTCTTCTTTTATTTATAAGATCCAAAAAATTCCCGGTCCAAACCTTTTTTAATTTACTACtttt------gagttTCTTTTCATTGACAGAGACCCAAGTCATATATTAAAATGATACTGATacttc--------------------------------------cgtaaTGGTCGGCATAGCTTAATTGCGGAGGACTGAAAATCCTTGTGTCACCATT--------------------------------------------------------------------------------------------------------------------------------------------------------------------------------------------------------CGTAAAATGAGGCTGATACTTCGGTAATGGCCGGGATAGCTCAgttg

trnLF_IK NNNNNNNNNNNNNNNNNNNNNNNNNNNCTACTAAGTGATAACTTTCAAATTCAGAGAAACCCTGGAATTAAcaatgggcAATCCTGAGCCAAATCCTGGTTTACGTGAACAAACCGGAGTTTAGAAAGCGAGAAAAAA-GGGATAGGTGCAGAGACTCAATGGAAGCTGTTCTAACAAATGGAGTTCACTACCTTGTGTTGATAAAGGAATCCTTCGATCAAAACTTCAAATCAAAAAG-GATGAAGGAGAAAAACCTATATTGTCTAAATATA------GGTAACACAAAA-CGATCGCAAAAATGACGACCTGAATCTCGATTTCTATTTTTTT--ATAAACAAAATCGAAATGATGTGAATCAATTCGAA-----GTTTAAGAAAtaatattcattGATCAAATGATTCTCTTCATAGTCTGATAGATCCGTGGTGGAActtattaaTCGGCCGAGAATAAAGATAGAGTCCCATTTTAC-ATGTCAATACTGACAACAATGAAATTTATAGTAAGATGXXXGCTCTACT-CCCCAAAAAGGTCTGTTTGACACCTTACCTtttt-gtag-TTATTATCCA-TTTGAATTATTTAGAATCTATATCAGTTTTCATTTTCAAACTTAGAAAGTCTTCTTTTATTTATAAGATCAAAAAAATTCCCGGTCCAAAACTTTTTTAATTTACTACtttt------gagttTCTTTTCATTGACAGAGACCCAAGTCATATATTAAAATGATACTGATacttc--------------------------------------cgtaaTGGTCGGCATAGCTTAATTGCGGAGGACTGAAAATCCTTGTGTCACCATT--------------------------------------------------------------------------------------------AGTAAAATGATAA-TGATACTTCAGTAGATGATACCTCAGTAATGGTGGACATAGC-------TTTTTTG--CAGAGGACTGAAAATC------CTTGTGTCACCATTAGTAAAATGAGGATGATACTTCGGTAATGGCCGGGATAGCTCAgttg

trnLF_IL AATTGGATTGAGCCTTGGTATGGAAACCTACTAAGTGATAACTTTCAAATTCAGAGAAACCCTGGAATTAAcaatgggcAATCCTGAGCCAAATCCTGGTTTACGTGAACAAACCGGAGTTTAGAAAGCGAGAAAAAA-GGGATAGGTGCAGAGACTCAATGGAAGCTGTTCTAACAAATGGAGTTCACTACCTTGTGTTGATAAAGGAATCCTTCGATCAAAACTTCAAATCAAAAAG-GATGAAGGAGAAAAACCTATATTGTCTAAATATA------GGTAACACAAAA-CGATCGCAAAAATGACGACCTGAATCTCGATTTCTATTTTTTT--ATAAACAAAATCGAAATGATGTGAATCAATTCGAA-----GTTTAAGAAAtaatattcattGATCAAATGATTCTCTTCATAGTCTGATAGATCCGTGGTGGAActtattaaTCGGCCGAGAATAAAGATAGAGTCCCATTTTAC-ATGTCAATACTGACAACAATGAAATTTATAGTAAGATGXXXGCTCTACT-CCCCAAAAAGGTCTGTTTGACACCTTACCTtttt-gtag-TTATTATCCA-TTTGAATTATTTAGAATCTATATCAGTTTTCATTTTCAAACTTAGAAAGTCTTCTTTTATTTATAAGATCCAAAAAATTCCCGGTCCAAAACTTTTTTAATTTACTACtttt------gagttTCTTTTCATTGACAGAGACCCAAGTCATATATTAAAATGATACTGATacttc--------------------------------------cgtaaTGGTCGGCATAGCTTAATTGCGGAGGACTGAAAATCCTTGTGTCACCATT--------------------------------------------------------------------------------------------AGTAAAATGATAA-TGATACTTCAGTAGATGATACCTCAGTAATGGTGGACATAGC-------TTTTTTG--CAGAGGACTGAAAATC------CTTGTGTCACCATTCGTAAAATGAGGATGATACTTCGGTAATGGCCGGGATAGCTCAgttg

trnLF_IM AATTGGATTGAGCCTTGGTATGGAAACCTACTAAGTGATAACTTTCAAATTCAGAGAAACCCTGGAATTAAcaatgggcAATCCTGAGCCAAATCCTGGTTTACGTGAACAAACCGGAGTTTAGAAAGCGAGAAAAAA-GGGATAGGTGCAGAGACTCAATGGAAGCTGTTCTAACAAATGGAGTTCACTACCTTGTGTTGATAAAGGAATCCTTCGATCAAAACTTCAAATCAAAAAG-GATGAAGGAGAAAAACCTATATTGTCTAAATATA------GGTAACACAAAA-CGATCGCAAAAATGACGACCTGAATCTCGATTTCTATTTTTTT--ATAAACAAAATCGAAATGATGTGAATCAATTCGAA-----GTTTAAGAAAtaatattcattGATCAAATGATTCTCTTCATAGTCTGATAGATCCGTGGTGGAActtattaaTCGGCCGAGAATAAAGATAGAGTCCCATTTTAC-ATGTCAATACTGACAACAATGAAATTTATAGTAAGATGXXXGCTCTACT-CCCCAAAAAGGTCTGTTTGACACCTTACCTtttt-gtag-TTATTATCCA-TTTGAATTATTTAGAATCTATATCAGTTTTCATTTTCAAACTTAGAAAGTCTTCTTTTATTTATAAGATCCAAAAAATTCCCGGTCCAAAACTTTTTTAATTTACTACtttt------gagttTCTTTTCATTGACAGAGACCCAAGTCATATATTAAAATGATACTGATacttc--------------------------------------cgtaaTGGTCGGCATAGCTTAATTGCGGAGGACTGAAAATCCTTGTGTCACCATT--------------------------------------------------------------------------------------------ATTAAAATGATAA-TGATACTTCAGTAGATGATACCTCAGTAATGGTGGACATAGC-------TTTTTTG--CAGAGGACTGAAAATC------CTTGTGTCACCATTCGTAAAATGAGGATGATACTTCGGTAATGGCCGGGATAGCTCAgttg

trnLF_IO AATTGGATTGAGCCTTGGTATGGAAACCTACTAAGTGATAACTTTCAAATTCAGAGAAACCCTGGAATTAAcaatgggcAATCCTGAGCCAAATCCTGGTTTACGTGAACAAACCGGAGTTTAGAAAGCGAGAAAAAA-GGGATAGGTGCAGAGACTCAATGGAAGCTGTTCTAACAAATGGAGTTCACTACCTTGTGTTGATAAAGGAATCCTTCGATCAAAACTTCAAATCAAAAAG-GATGAAGGAGAAAAACCTATATTGTCTAAATATA------GGTAACACAAAA-CGATCTCAAAAATGACGACCTGAATCTCGATTTCTATTTTTTT--ATAAACAAAATCGAAATGATGTGAATCAATTCGAA-----GTTTAAGAAAtaatattcattGATCAAATGATTCTCTTCATAGTCTAATAGATCCGTGGTGGAActtattaaTCGGACGAGAATAAAGATAGAGTCCCATTTTAC-ATGTCAATACTGACAACAATGAAATTTATAGTAAGATGXXXGCTCTACT-CCCCAAAAAGGTCTGTTTGACACCTTACCTtttt-gtag-TTATTATCCA-TTTGAATTATTTAGAATCTATATCAGTTTTCATTTTCAAACTTAGAAAGTCTTCTTTTATTTATAAGATCCAAAAAATTCCCGGTCCAAAACTTTTTTAATTTACTACtttt------gagttTCTTTTCATTGACAGAGACCCAAGTCATATATTAAAATGATACTGATacttc--------------------------------------cgtaaTGGTCGGCATAGCTTAATTGCGGAGGACTGAAAATCCTTGTGTCACCATT--------------------------------------------------------------------------------------------AGTAAAATGATAA-TGATACTTCAGTAGATGATACCTCAGTAATGGTGGACATAGC-------TTTTTTG--CAGAGGACTGAAAATC------CTTGTGTCACCATTCGTAAAATGAGGATGATACTTCGGTAATGGCCGGGATAGCTCAgttg

trnLF_IP AATTGGATTGAGCCTTGGTATGGAAACCTACTAAGTGATAACTTTCAAATTCAGAGAAACCCTGGAATTAAcaatgggcAATCCTGAGCCAAATCCTGGTTTACGTGAACAAACCGGAGTTTAGAAAGCGAGAAAAAA-GGGATAGGTGCAGAGACTCAATGGAAGCTGTTCTAACAAATGGAGTTCACTACCTTGTGTTGATAAAGGAATCCTTCGATCAAAACTTCAAATAAAAAAG-GATGAAGGAGAAAAACCTATATTGTCTAAATATA------GGTAACACAAAA-CGATCGCAAAAATGACGACCTGAATCTCGATTTCTATTTTTTT--ATAAACAAAATCGAAATGATGTGAATCAATTCGAA-----GTTTAAGAAAtaatattcattGATCAAATGATTCTCTTCATAGTCTGATAGATCCGTGGTGGAActtattaaTCGGCCGAGAATAAAGATAGAGTCCCATTTTAC-ATGTCAATACTGACAACAATGAAATTTATAGTAAGATGXXXGCTCTACT-CCCCAAAAAGGTCTGTTTGACACCTTACCTtttt-gtag-TTATTATCCA-TTTGAATTATTTAGAATCTATATCAGTTTTCATTTTCAAACTTAGAAAGTCTTCTTTTATTTATAAGATCCAAAAAATTCCCGGTCCAAAACTTTTTTAATTTACTACtttt------gagttTCTTTTCATTGACAGAGACCCAAGTCATATATTAAAATGATACTGATacttc--------------------------------------cgtaaTGGTCGGCATAGCTTAATTGCGGAGGACTGAAAATCCTTGTGTCACCATT--------------------------------------------------------------------------------------------AGTAAAATGATAA-TGATACTTCAGTAGATGATACCTCAGTAATGGTGGACATAGC-------TTTTTTG--CAGAGGACTGAAAATC------CTTGTGTCACCATTCGTAAAATGAGGATGATACTTCGGTAATGGCCGGGATAGCTCAgttg

trnLF_IU AATTGGATTGAGCCTTGGTATGGAAACCTACTAAGTGATAACTTTCAAATTCAGAGAAACCCTGGAATTAAcaatgggcAATCCTGAGCCAAATCCTGGTTTATGTGAACAAACCGGAGTTTAGAAAGCGAGAAAAAA-GGGATAGGTGCAGAGACTCAATGGAAGCTGTTCTAACAAATGGAGTTCACAACCTTGTGTTGATAAAGGAATCCTTCGATCAAAACTTCAAATAAAAAAG-GATGAAGGAGAAAAACCTATATTGTCTAAATATA------GGTAACACAAAA-CGATCTCAAAAATGACGACCTGAATCTCGATTTCTATTTTTTT--ATAAACAAAATCGAAATGATGTGAATCAATTCGAA-----GTTTAAGAAAtaatattcattGATCAAATGATTCTCTTCATAGTCTGATAGATCCGTGGTGGAActtattaaTCGGACGAGAATAAAGATAGAGTCCCATTTTAC-ATGTCAATACTGACAACAATGAAATTTATAGTAAGATGXXXGCTCTACT-CCCCAAAAAGGTCTGTTTGACACCTTACCTtttt-gtag-TTATTATCCA-TTTGAATTATTTAGAATCTATATCAGTTTTCATTTTCAAACTTAGAAAGTCTTCTTTTATTTATAAGATCCAAAAAATTCCCGGTCCAAAACTTTTTTAATTTACTACtttt------gagttTCTTTTCATTGACAGAGACCCAAGTCATATATTAAAATGATACTGATacttc--------------------------------------cgtaaTGGTCGGCATAGCTTAATTGCGGAGGACTGAAAATCCTTGTGTCACC--------------------------------------------------------------------------------------------------AAAATGATAA-TGATACTTCAGTAGATGATACCTCAGTAATGGTGGACATAGC-------TTTTTTG--CAGAGGACTGAAAATC------CTTGTGTCACCATTCGTAAAATGAGGATGATACTTCGGTAATGGCCGGGATAGCTCAgttg

trnLF_IY AATTGGATTGAGCCTTGGTATGGAAACCTACTAAGTGATAACTTTCAAATTCAGAGAAACCCTGGAATTAAcaatgggcAATCCTGAGCCAAATCCTGGTTTATGTGAACAAACCGGAGTTTAGAAAGCGAGAAAAAA-GGGATAGGTGCAGAGACTCAATGGAAGCTGTTCTAACAAATGGAGTTCACAACCTTGTGTTGATAAAGGAATCCTTCGATCAAAACTTCAAATAAAAAAG-GATGAAGGAGAAAAACCTATATTGTCTAAATATA------GGTAACACAAAA-CGATCTCAAAAATGACGACCTGAATCTCGATTTCTATTTTTTT--ATAAACAAAATCGAAATGATGTGAATCAATTCGAA-----GTTTAAGAAAtaatattcattGATCAAATGATTCTCTTCATAGTCTGATAGATCCGTGGTGGAActtattaaTCGGACGAGAATAAAGATAGAGTCCCATTTTAC-ATGTCAATACTGACAACAATGAAATTTATAGTAANNNNXXXGCTCTACT-CCCCAAAAAGGTCTGTTTGACACCTTACCTtttt-gtag-TTATTATCCA-TTTGAATTATTTAGAATCTATATCAGTTTTCATTTTCAAACTTAGAAAGTCTTCTTTTATTTATAAGATCCAAAAAATTCCCGGTCCAAAACTTTTTTAATTTACTACtttt------gagttTCTTTTCATTGACAGAGACCCAAGTCATATATTAAAATGATACTGATTCTTC--------------------------------------CGTAATGGTCGGCATAGCTTAATTGCGGAGGACTGAAAATCCTTGTGTCACCATT--------------------------------------------------------------------------------------------AGTAAAATGATAA-TGATACTTCAGTAGATGATACCTCAGTAATGGTGGACATAGC-------TTTTTTG--CAGAGGACTGAAAATC------CTTGTGTCACCATTCGTAAAATGAGGATGATACTTCGGTAATGGCCGGGATAGCTCAgttg

trnLF_GA AATTGGATTGAGCCTTGGTATGGAAACCTACTAAGTGATAACTTTCAAATTCAGAGAAACCCTGGAATTAACAATGGGCAATCCTGAGCCAAATCCTGGTTTATGTGAACAAACCGGAGTTTAGAAAGCGAGAAAAAA-GGGATAGGTGCAGAGACTCAATGGAAGCTGTTCTAACAAATGGAGTTCACWACCTTGTGTTGATAAAGGAATCCTTCGATCAAAACTTCAAATAAAAAAG-GATGAAGGAGAAAAACCTATATTGTCTAAATATA------GGTAACACAAAA-CGATCTCAAAAATGACGACCTGAATCTCGATTTCTATTTTTTT--ATAAACAAAATCGAAATGATGTGAATCAATTCGAA-----GTTTAAGAAATAATATTCATTGATCAAATGATTCTCTTCATAGTCTGATAGATCCGTGGTGGAACTTATTAATCGGACGAGAATAAAGATAGAGTCCCATTTTAC-ATGTCAATACTGACAACAATGAAATTTATAGTAAGATGXXXGCTCTACT-CCCCAAAAAGGTCTGTTTGACACCTTACCTTTTT-GTAGGTTATTATCCA-TTTGAATTATTTAGAATCTATATCAGTTTTCATTTTCAAACTTAGAAAGTCTTCTTTTATTTATAAGATCCAAAAAATTCCCGGTCCAAAACTTTTTTAATTTACTACTTTT------GAGTTTCTTTTCATTGACAGAGACCCAAGTCATATATTAAAATGATACTGATACTTC--------------------------------------CGTAATGGTCGGCATAGCTTAATTGCGGAGGACTGAAAATCCTTGTGTCACCATT--------------------------------------------------------------------------------------------AGTAAAATGATAA-TGATACTTCAGTAGATGATACCTCAGTAATGGTGGACATAGC-------TTTTTTG--CAGAGGACTGAAAATC------CTTGTGTCACCATTCGTAAAATGAGGATGATACTTCGGTAATGGCCGGGATAGCTCAgttg

trnLF_GD AATTGGATTGAGCCTTGGTATGGAAACCTACTAAGTGATAACTTTCAAATTCAGAGAAACCCTGGAATTAACAATGGGCAATCCTGAGCCAAATCCTGGTTTATGTGAACAAACCGGAGTTTAGAAAGCGAGAAAAAA-GGGATAGGTGCAGAGACTCAATGGAAGCTGTTCTAACAAATGGAGTTCACAACCTTGTGTTGATAAAGGAATCCTTCGATCAAAACTTCAAATAAAAAAG-GATGAAGGAGAAAAACCTATATTGTCTAAATATA------GGTAACACAAAA-CGATCTCAAAAATGACGACCTGAATCTCGATTTCTATTTTTTT--ATAAACAAAATCGAAATGATGTGAATCAATTCGAA-----GTTTAAGAAATAATATTCATTGATCAAATGATTCTCTTCATAGTCTGATAGATCCGTGGTGGAACTTATTAATCGGACGAGAATAAAGATAGAGTCCCATTTTAC-ATGTCAATACTGACAACAATGAAATTTATAGTAAGATGXXXGCTCTACT-CCCCAAAAAGGTCTGTTTGACACCTTACCTTTTT-GTAG-TTATTATCCA-TTTGAATTATTTAGAATCTATATCAGTTTTCATTTTCAAACTTAGAAAGTCTTCTTTTATTTATAAGATCCAAAAAATTCCCGGTCCAAAACTTTTTTAATTTACTACTTTT-GTTTTGAGTTTCTTTTCATTGACAGAGACCCAAGTCATATATTAAAATGATACTGATACTTC--------------------------------------CGTAATGGTCGGCATAGCTTAATTGCGGAGGACTGAAAATCCTTGTGTCACCATT--------------------------------------------------------------------------------------------ATTAAAATGATAA-TGATACTTCAGTAGATGATACCTCAGTAATGGTGGACATAGC-------TTTTTTG--CAGAGGACTGAAAATC------CTTGTGTCACCATTCGTAAAATGAGGATGATACTTCGGTAATGGCCGGGATAGCTCAgttg

trnLF_GI AATTGGATTGAGCCTTGGTATGGAAACCTACTAAGTGATAACTTTCAAATTCAGAGAAACCCTGGAATTAAcaatgggcAATCCTGAGCCAAATCCTGGTTTATGTGAACAAACCGGAGTTTAGAAAGCGAGAAAAAA-GGGATAGGTGCAGAGACTCAATGGAAGCTGTTCTAACAAATGGAGTTCACAACCTTGTGTTGATAAAGGAATCCTTCGATCAAAACTTCAAATAAAAAAG-GATGAAGGAGAAAAACCTATATTGTCTAAATATA------GGTAACACAAAA-CGATCTCAAAAATAACGACCTGAATCTCGATTTCTATTTTTTT--ATAAACAAAATCGAAATGATGTGAATCAATTCGAA-----GTTTAAGAAAtaatattcattGATCAAATGATTCTCTTCATAGTCTGATAGATCCGTGGTGGAActtattaaTCGGACGAGAATAAAGATAGAGTCCCATTTTAC-ATGTCAATACTGACAACAATGAAATTTATAGTAAGATGXXXGCTCTACT-CCCCAAAAAGGTCTGTTTGACACCTTACCTtttt-gtag-TTATTATCCA-TTTGAATTATTTAGAATCTATATCAGTTTTCATTTTCAAACTTAGAAAGTCTTCTTTTATTTATAAGATCCAAAAAATTCCCGGTCCAAAACTTTTTTAATTTACTACtttt------gagttTCTTTTCATTGACAGAGACCCAAGTCATATATTAAAATGATACTGATacttc--------------------------------------cgtaaTGGTCGGCATAGCTTAATTGCGGAGGACTGAAAATCCTTGTGTCACCATT--------------------------------------------------------------------------------------------AGTAAAATGATAA-TGATACTTCAGTAGATGATACCTCAGTAATGGTGGACATAGC-------TTTTTTG--CAGAGGACTGAAAATC------CTTGTGTCACCATTCGTAAAATGAGGATGATACTTCGGTAATGGCCGGGATAGCTCAgttg

trnLF_GK AATTGGATTGAGCCTTGGTATGGAAACCTACTAAGTGATAACTTTCAAATTCAGAGAAACCCTGGAATTAAcaatgggcAATCCTGAGCCAAATCCTGGTTTATGTGAACAAACCGGAGTTTAGAAAGCGAGAAAAAA-GGGATAGGTGCAGAGACTCAATGGAAGCTGTTCTAACAAATGGAGTTCACAACCTTGTGTTGATAAAGGAATCCTTCGATCAAAACTTCAAATAAAAAAG-GATGAAGGAGAAAAACCTATATTGTCTAAATATA------GGTAACACAAAA-CGATCTCAAAAATAACGACCTGAATCTCGATTTCTATTTTTTT--ATAAACAAAATCGAAATGATGTGAATCAATTCGAA-----GTTTAAGAAAtaatattcattGATCAAATGATTCTCTTCATAGTCTGATAGATCCGTGGTGGAActtattaaTCGGACGAGAATAAAGATAGAGTCCCATTTTAC-ATGTCAATACTGACAACAATGAAATTTATAGTAAGATGXXXGCTCTACT-CCCCAAAAAGGTCTGTTTGACACCTTACCTtttt-gtag-TTATTATCCA-TTTGAATTATTTAGAATCTATATCAGTTTTCATTTTCAAACTTAGAAAGTCTTCTTTTATTTATAAGATCCAAAAAATTCCCGGTCCAAAACTTTTTTAATTTACTACtttt------gagttTCTTTTCATTGACAGAGACCCAAGTCATATATTAAAATGATACTGATacttc--------------------------------------cgtaaTGGTCGGCATAGCTTAATTGCGGAGGACTGAAAATCCTTGTGTCACCATT--------------------------------------------------------------------------------------------AGTAAAATGATAA-TGATACTTCAGTAGATGATACCTCAGTAATGGTGGACATAGC-------TTTTTTT--CAGAGGACTGAAAATC------CTTGTGTCACCATTCGTAAAATGAGGATGATACTTCGGTAATGGCCGGGATAGCTCAgttg

trnLF_GL AATTGGATTGAGCCTTGGTATGGAAACCTACTAAGTGATAACTTTCAAATTCAGAGAAACCCTGGAATTAAcaatgggcAATCCTGAGCCAAATCCTGGTTTACGTGAACAAACCGGAGTTTAGAAAGCGAGAAAAAA--GGATAGGTGCAGAGACTCAATGGAAGCTGTTCTAACAAATGGAGTTCACTACCTTGTGTTGATAAAGGAATCCTTCGATCAAAACTTCAAATCAAAAAG-GATGAAGGAGAAAAACCTATATTGTCTAAATATA------GGTAACACAAAA-CGATCTCAAAAATGACGACCTGAATCTCGATTTCTATTTTTTT--ATAAACAAAATCGAAATGATGTGAATCAATTCGAA-----GTTTAAGAAAtaatattcattGATCAAATGATTCTCTTCATAGTCTGATAGATCCGTGGTGGAActtattaaTCGGACGAGAATAAAGATAGAGTCCCATTTTAC-ATGTCAATACTGACAACAATGAAATTTATAGTAAGATGXXXGCTCTACTCCCCCAAAAAGGTCTGTTTGACACCTTACCTtttt-gtag-TTATTATCCA-TTTGAATTATTTAGAATCTATATCAGTTTTCATTTTCAAACTTAGAAAGTCTTCTTTTATTTATAAGATCCAAAAAATTCCCGGTCCAAAACTTTTTTAATTTACTACtttt------gagttTCTTTTCATTGACAGAGACCCAAGTCATATATTAAAATGATACTGATacttc--------------------------------------cgtaaTGGTCGGCATAGCTTAATTGCGGAGGACTGAAAATCCTTGTGTCACCATT--------------------------------------------------------------------------------------------AGTAAAATGATAA-TGATACTTCAGTAGATGATACCTCAGTAATGGTGGACATAGC-------TTTTTTG--CAGAGGACTGAAAATC------CTTGTGTCACCATTCGTAAAATGAGGATGATACTTCGGTAATGGCCGGGATAGCTCAgttg

trnLF_GN AATTGGATTGAGCCTTGGTATGGAAACCTACTAAGTGATAACTTTCAAATTCAGAGAAACCCTGGAATTAAcaatgggcAATCCTGAGCCAAATCCTGGTTTATGTGAACAAACCGGAGTTTAGAAAGCGAGAAAAAA-GGGATAGGTGCAGAGACTCAATGGAAGCTGTTCTAACAAATGGAGTTCACTACCTTGTGTTGATAAAGGAATCCTTCGATCAAAACTTCAAATAAAAAAG-GATGAAGGAGAAAAACCTATATTGTCTAAATATA------GGTAACACAAAA-CGATCTCAAAAATGACGACCTGAATCTCGATTTCTATTTTTTT--ATAAACAAAATCGAAATGATGTGAATCAATTCGAA-----GTTTAAGAAAtaatattcattGATCAAATGATTCTCTTCATAGTCTGATAGATCCGTGGTGGAActtattaaTCGGACGAGAATAAAGATAGAGTCCCATTTTAC-ATGTCAATACTGACAACAATGAAATTTATAGTAAGATGXXXGCTCTACT-CCCCAAAAAGGTCTGTTTGACACCTTACCTtttt-gtag-TTATTATCCA-TTTGAATTATTTAGAATCTATATCAGTTTTCATTTTCAAACTTAGAAAGTCTTCTTTTATTTATAAGATCCAAAAAATTCCCGGTCCAAAACTTTTTTAATTTACTACTTTT------GAGTTTCTTTTCATTGACAGAGACCCAAGCCATATATTAAAATGATACTGATacttc--------------------------------------cgtaaTGGTCGGCATAGCTTAATTGCGGAGGACTGAAAATCCTTGTGTCACCATT--------------------------------------------------------------------------------------------------------------------------------------------------------------------------------------------------------CGTAAAATGAGGATGATACTTCGGTAATGGCCGGGATAGCTCAgttg

trnLF_GO NNNNNNNNNNNNNNNNNNNNNNNNNNNNNNNNNAGTGATAACTTTCAAATTCAGAGAAACCCTGGAATTAACAATGGGCAATCCTGAGCCAAATCCTGGTTTATGTGAACAAACCGGAGTTTAGAAAGCGAGAAAAAA-GGGATAGGTGCAGAGACTCAATGGAAGCTGTTCTAACAAATGGAGTTCACTACCTTGTGTTGATAAAGGAATCCTTCGATCAAAACTTCAAATAAAAAAG-GATGAAGGAGAAAAACCTATATTGTCTAAATATA------GGTAACACAAAA-CGATCTCAAAAATGACGACCTGAATCTCGATTTCTATTTTTTT--ATAAACAAAATCGAAATGATGTGAATCAATTCGAA-----GTTTAAGAAATAATATTCATTGATCAAATGATTCTCTTCATAGTCTGATAGATCCGTGGTGGAACTTATTAATCGGACGAGAATAAAGATAGAGTCCCATTTTAC-ATGTCAATACTGACAACAATGAAATTTATAGTAAGATGXXXGCTCTACT-CCCAAAAAAGGTCTGTTTGACACCTTACCTTTTT-GTAG-TTATTATCCA-TTTGAATTATTTAGAATCTATATCAGTTTTCATTTTCAAACTTAGAAAGTCTTCTTTTATTTATAAGATCCAAAAAATTCCCGGTCCAAAACTTTTTTAATTTACTACTTTT------GAGTTTCTTTTCATTGACAGAGACCCAAGTCATATATTAAAATGATACTGATACTTC--------------------------------------CGTAATGGTCGGCATAGCTTAATTGCGGAGGACTGAAAATCCTTGTGTCACCATT--------------------------------------------------------------------------------------------------------------------------------------------------------------------------------------------------------CGTAAAATGAGGATGANNNNNNNNNNNNNNNNNNNNNNNNNNNnnnn

trnLF_GP AATTGGATTGAGCCTTGGTATGGAAACCTACTAAGTGATAACTTTCAAATTCAGAGAAACCCTGGAATTAACAATGGGCAATCCTGAGCCAAATCCTGGTTTATGTGAACAAACCGGAGTTTAGAAAGCGAGAAAAAA-GGGATAGGTGCAGAGACTCAATGGAAGCTGTTCTAACAAATGGAGTTCACAACCTTGTGTTGATAAAGGAATCCTTCGATCAAAACTTCAAATAAAAAAG-AATGAAGGAGAAAAACCTATATTGTCTAAATATA------GGTAACACAAAA-CGATCTCAAAAATGACGACCTGAATCTCGATTTCTATTTTTTT--ATAAACAAAATCGAAATGATGTGAATCAATTCGAA-----GTTTAAGAAATAATATTCATTGATCAAATGATTCTCTTCATAGTCTGATAGATCCGTGGTGGAACTTATTAATCGGACGAGAATAAAGATAGAGTCCCATTTTAC-ATGTCAATACTGACAACAATGAAATTTATAGTAAGATGXXXGCTCTACT-CCCCAAAAAGGTCTGTTTGACACCTTACCTTTTT-GTAG-TTATTATCCA-TTTGAATTATTTAGAATCTATATCAGTTTTCATTTTCAAACTTAGAAAGTCTTCTTTTATTTATAAGATCCAAAAAATTCCCGGTCCAAAACTTTTTTAATTTACTACTTTT------GAGTTTCTTTTCATTGACAGAGACCCAAGTCATATATTAAAATGATACTGATACTTC--------------------------------------CGTAATGGTCGGCATAGCTTAATTGCGGAGGACTGAAAATCCTTGTGTCACCATT--------------------------------------------------------------------------------------------AGTAAAATGATAA-TGATACTTCAGTAGATGATACCTCAGTAATGGTGGACATAGC-------TTTTTTG--CAGAGGACTGAAAATC------CTTGTGTCACCATTCGTAAAATGAGGATGATACTTCGGTAATGGCCGGGATAGCTCAgttg

trnLF_GQ AATTGGATTGAGCCTTGGTATGGAAACCTACTAAGTGATAACTTTCAAATTCAGAGAAACCCTGGAATTAACAATGGGCAATCCTGAGCCAAATCCTGGTTTATGTGAACAAACCGGAGTTTAGAAAGCGAGAAAAAA-GGGATAGGTGCAGAGACTCAATGGAAGCTGTTCTAACAAATGGAGTTCACAACCTTGTGTTGATAAAGGAATCCTTCGATCAAAACTTCAAATAAAAAAG-GATGAAGGAGAAAAACCTATATTGTCTAAATATA------GGTAACACAAAA-CGATCTCAAAAATGACGACCTGAATCTCGATTTCTATTTTTTT--ATAAACAAAATCGAAATGATGTGAATCAATTTGAA-----GTTTAAGAAATAATATTCATTGATCAAATGATTCTCTTCATAGTCTGATAGATCCGTGGTGGAACTTATTAATCGGACGAGAATAAAGATAGAGTCCCATTTTAC-ATGTCAATACTGACAACAATGAAATTTATAGTAAGATGXXXGCTCTACT-CCCCAAAAAGGTCTGTTTGACACCTTACCTTTTT-GTAG-TTATTATCCA-TTTGAATTATTTAGAATCTATATCAGTTTTCATTTTCAAACTTAGAAAGTCTTCTTTTATTTATAAGATCCAAAAAATTCCCGGTCCAAAACTTTTTTAATTTACTACTTTT------GAGTTTCTTTTCATTGACAGAGACCCAAGTCATATATTAAAATGATACTGATACTTC--------------------------------------CGTAATGGTCGGCATAGCTTAATTGCGGAGGACTGAAAATCCTTGTGTCACCATT--------------------------------------------------------------------------------------------------------------------------------------------------------------------------------------------------------CGTAAAATGAGGATGATACTTCGGTAATGGCCGGGATAGCTCAgttg

trnLF_GR AATTGGATTGAGCCTTGGTATGGAAACCTACTAAGTGATAACTTTCAAATTCAGAGAAACCCTGGAATTAACAATGGGCAATCCTGAGCCAAATCCTGGTTTATGTGAACAAACCGGAGTTTAGAAAGCGAGAAAAA--GGGATAGGTGCAGAGACTCAATGGAAGCTGTTCTAACAAATGGAGTTCACAACCTTGTGTTGATAAAGGAATCCTTCGATCAAAACTTCAAATAAAAAAG-GATGAAGGAGAAAAACCTATATTGTCTAAATATA------GGTAACACAAAA-CGATCTCAAAAATGACGACCTGAATCTCGATTTCTATTTTTTT--ATAAACAAAATCGAAATGATGTGAATCAATTCGAA-----GTTTAAGAAATAATATTCATTGATCAAATGATTCTCTTCATAGTCTGATAGATCCGTGGTGGAACTTATTAATCGGACGAGAATAAAGATAGAGTCCCATTTTAC-ATGTCAATACTGACAACAATGAAATTTATAGTAAGATGXXXGCTCTACT-CCCCAAAAAGGTCTGTTTGACACCTTACCTTTTT-GTAG-TTATTATCCA-TTTGAATTATTTAGAATCTATATCAGTTTTCATTTTCAAACTTAGAAAGTCTTCTTTTATTTATAAGATCCAAAAAATTCCCGGTCCAAAACTTTTTTAATTTACTACTTTT------GAGTTTCTTTTCATTGACAGAGACCCAAGTCATATATTAAAATGATACTGATACTTC--------------------------------------CGTAATGGTCGGCATAGCTTAATTGCGGAGGACTGAAAATCCTTGTGTCACCATT--------------------------------------------------------------------------------------------AGTAAAATGATAA-TGATACTTCAGTAGATGATACCTCAGTAATGGTGGACATAGC-------TTTTTTG--CAGAGGACTGAAAATC------CTTGTGTCACCATTCGTAAAATGAGGATGATACTTCGGTAATGGCCGGGATAGCTCAgttg

trnLF_GS AATTGGATTGAGCCTTGGTATGGAAACCTACTAAGTGATAACTTTCAAATTCAGAGAAACCCTGGAATTAACAATGGGCAATCCTGAGCCAAATCCTGGTTTATGTGAACAAACCGGAGTTTAGAAAGCGAGAAAAAA-GGGATAGGTGCAGAGACTCAATGGAAGCTGTTCTAACAAATGGAGTTCACAACCTTGTGTTGATAAAGGAATCCTTCGATCAAAACTTCAAATAAAAAAG-GATGAAGGAGAAAAACCTATATTGTCTAAATATA------GGTAACACAAAA-CGATCTCAAAAATGACGACCTGAATCTCGATTTCTATTTTTTT--ATAAACAAAATCGAAATGATGTGAATCAATTCGAA-----GTTTAAGAAATAATATTCATTGATCAAATGATTCTCTTCATAGTCTGATAGATCCGTGGTGGAACTTATTAATCGGACGAGAATAAAGATAGAGTCCCATTTTAC-ATGTCAATACTGACAACAATGAAATTTATAGTAAGATGXXXGCTCTACT-CCCCAAAAAGGTCTGTTTGACACCTTACCTTTTT-GTAG-TTATTATCCA-TTTGAATTATTTAGAATCTATATCAATTTTCATTTTCAAACTTAGAAAGTCTTCTTTTATTTATAAGATCCAAAAAATTCCCGGTCCAAAACTTTTTTAATTTACTACTTTT------GAGTTTCTTTTCATTGACAGAGACCCAAGTCATATATTAAAATGATACTGATACTTC--------------------------------------CGTAATGGTCGGCATAGCTTAATTGCGGAGGACTGAAAATCCTTGTGTCACCATT--------------------------------------------------------------------------------------------AGTAAAATGATAA-TGATACTTCAGTAGATGATACCTCAGTAATGGTGGACATAGC-------TTTTTTG--CAGAGGACTGAAAATC------CTTGTGTCACCATTCGTAAAATGAGGATGATACTTCGGTAATGGCCGGGATAGCTCAgttg

trnLF_GT AATTGGATTGAGCCTTGGTATGGAAACCTACTAAGTGATAACTTTCAAATTCAGAGAAACCCTGGAATTAAcaatgggcAATCCTGAGCCAAATCCTGGTTTATGTGAACAAACCGGAGTTTAGAAAGCGAGAAAAAA-GGGATAGGTGCAGAGACTCAATGGAAGCTGTTCTAACAAATGGAGTTCACTACCTTGTGTTGATAAAGGAATTCTTCGATCAAAACTTCAAATAAAAAAG-GATGAAGGAGAAAAACCTATATTGTCTAAATATA------GGTAACACAAAA-CGATCTCAAAAATGACGACCTGAATCTCGATTTCTATTTTTTT--ATAAACAAAATCGAAATGATGTGAATCAATTCGAA-----GTTTAAGAAAtaatattcattGATCAAATGATTCTCTTCATAGTCTGATAGATCCGTGGTGGAActtattaaTCGGACGAGAATAAAGATAGAGTCCCATTTTAC-ATGTCAATACTGACAACAATGAAATTTATAGTAAGATGXXXGCTCTACT-CCCCAAAAAGGTCTGTTTGACACCTTACCTtttt-gtag-TTATTATCCA-TTTGAATTATTTAGAATCTACATCAGTTTTCATTTTCAAACTTAGAAAGTCTTCTTTTATTTATAAGATCCAAAAAATTCCCGGTCCAAAACTTTTTTAATTTACTACTTTT------GAGTTTCTTTTCATTGACAGAGACCCAAGTCATATATTAAAATGATACTGATacttc--------------------------------------cgtaaTGGTCGGCATAGCTTAATTGCGGAGGACTGAAAATCCTTGTGTCACCATT--------------------------------------------------------------------------------------------AGTAAAATGATAA-TGATACTTCAGTAGATGATACCTCAGTAATGGTGGACATAGC-------TTTTTTG--CAGAGGACTGAAAATC------CTTGTGTCACCATTCGTAAAATGAGGATGATACTTCGGTAATGGCCGGGATAGCTCAgttg

trnLF_GU AATTGGATTGAGCCTTGGTATGGAAACCTACTAAGTGATAACTTTCAAATTCAGAGAAACCCTGGAATTAAcaatgggcAATCCTGAGCCAAATCCTGGTTTATGTGAACAAACCGGAGTTTAGAAAGCGAGAAAAAA-GGGATAGGTGCAGAGACTCAATGGAAGCTGTTCTAACAAATGGAGTTCACAACCTTGTGTTGATAAAGGAATCCTTCGATCAAAACTTCAAATAAAAAAG-GATGAAGGAGAAAAACCTATATTGTCTAAATATA------GGTAACACAAAA-CGATCTCAAAAATGACGACCTGAATCTCGATTTCTATTTTTTT--ATAAACAAAATCGAAATGATGTGAATCAATTTGAA-----GTTTAAGAAAtaatattcattGATCAAATGATTCTCTTCATAGTCTGATAGATCCGTGGTGGAActtattaaTCGGACGAGAATAAAGATAGAGTCCCATTTTAC-ATGTCAATACTGACAACAATGAAATTTATAGTAAGATGXXXGCTCTACT-CCCCAAAAAGGTCTGTTTGACACCTTACCTTTTT-GTAG-TTATTATCCA-TTTGAATTATTTAGAATCTATATAAGTTTTCATTTTCAAACTTAGAAAGTCTTCTTTTATTTATAAGATCCAAAAAATTCCCGGTCCAAAACTTTTTTAATTTACTACTTTT------GAGTTTCTTTTCATTGACAGAGACCCAAGTCATATATTAAAATGATACTGATacttc--------------------------------------cgtaaTGGTCGGCATAGCTTAATTGCGGAGGACTGAAAATCCTTGTGTCACCATT--------------------------------------------------------------------------------------------------------------------------------------------------------------------------------------------------------CGTAAAATGAGGATGATACTTCGGTAATGGCCGGGATAGCTCAgttg

trnLF_GV AATTGGATTGAGCCTTGGTATGGAAACCTACTAAGTGATAACTTTCAAATTCAGAGAAACCCTGGAATTAAcaatgggcAATCCTGAGCCAAATCCTGGTTTATGTGAACAAACCGGAGTTTAGAAAGCGAGAAAAAA-GGGATAGGTGCAGAGACTCAATGGAAGCTGTTCTAACAAATGGAGTTCACAACCTTGTGTTGATAAAGGAATCCTTCGATCAAAACTTCAAATAAAAAAG-GATGAAGGAGAAAAACCTATATTGTCTAAATATAAATATAGGTAACACAAAA-CGATCTCAAAAATGACGACCTGAATCTCGATTTCTATTTTTTT--ATAAACAAAATCGAAATGATGTGAATCAATTCGAA-----GTTTAAGAAAtaatattcattGATCAAATGATTCTCTTCATAGTCTGATAGATCCGTGGTGGAActtattaaTCGGACGAGAATAAAGATAGAGTCCCATTTTAC-ATGTCAATACTGACAACAATGAAATTTATAGTAAGATGXXXGCTCTACT-CCCCAAAAAGGTCTGTTTGACACCTTACCTTTTT-GTAG-TTATTATCCA-TTTGAATTATTTAGAATCTATATCAGTTTTCATTTTCAAACTTAGAAAGTCTTCTTTTATTTATAAGATCCAAAAAATTCCCGGTCCAAAACTTTTTTAATTTACTACTTTT------GAGTTTCTTTTCATTGACAGAGACCCAAGTCATATATTAAAATGATACTGATacttc--------------------------------------cgtaaTGGTCGGCATAGCTTAATTGCGGAGGACTGAAAATCCTTGTGTCACCATT--------------------------------------------------------------------------------------------AGTAAAATGATAA-TGATACTTCAGTAGATGATACCTCAGTAATGGTGGACATAGC-------TTTTTTG--CAGAGGACTGAAAATC------CTTGTGTCACCATTCGTAAAATGAGGATGATACTTCGGTAATGGCCGGGATAGCTCAgttg

trnLF_GW AATTGGATTGAGCCTTGGTATGGAAACCTACTAAGTGATAACTTTCAAATTCAGAGAAACCCTGGAATTAAcaatgggcAATCCTGAGCCAAATCCTGGTTTATGTGAACAAACCGGAGTTTAGAAAGCGAGAAAAAA-GGGATAGGTGCAGAGACTCAATGGAAGCTGTTCTAACAAATGGAGTTCACTACCTTGTGTTGATAAAGGAATCCTTCGATCAAAACTTCAAATAAAAAAG-GATGAAGGAGAAAAACCTATATTGTCTAAATATA------GGTAACACAAAA-CGATCTCAAAAATGACGACCTGAATCTCGATTTCTATTTTTTT--ATAAACAAAATCGAAATGATGTGAATCAATTCGAA-----GTTTAAGAAAtaatattcattGATCAAATGATTCTCTTCATAGTCTGATAGATCCGTGGTGGAActtattaaTCGGACGAGAATAAAGATAGAGTCCCATTTTAC-ATGTCAATACTGACAACAATGAAATTTATAGTAAGATGXXXGCTCTACT-CCCCAAAAAGGTCTGTTTGACACCTTACCTTTTT-GTAG-TTATTATCCA-TTTGAATTATTTAGAATCTATATCAGTTTTCATTTTCAAACTTAGAAAGTCTTCTTTTATTTATAAGATCCAAAAAATTCCCGGTCCAAAACTTTTTTAATTTACTACTTTT------GAGTTTCTTTTCATTGACAGAGACCCAAGTCATATATTAAAATGATACTGATAC-------------------------------------------------------------------------------------------------------------------------------------------------------------------------------------------------------------------------------CTCAGTAATGGTGGACATAGC-------TTTTTTG--CAGAGGACTGAAAATC------CTTGTGTCACCATTCGTAAAATGAGGATGATACTTCGGTAATGGCCGGGATAGCTCAgttg

trnLF_GX AATTGGATTGAGCCTTGGTATGGAAACCTACTAAGTGATAACTTTCAAATTCAGAGAAACCCTGGAATTAAcaatgggcAATCCTGAGCCAAATCCTGGTTTATGTGAACAAACCGGAGTTTAGAAAGCGAGAAAAAA-GGGATAGGTGCAGAGACTCAATGGAAGCTGTTCTAACAAATGGAGTTCACAACCTTGTGTTGATAAAGGAATCCTTCAATCAAAACTTCAAATAAAAAAG-GATGAAGGAGAAAAACCTATATTGTCTAAATATA------GGTAACACAAAA-CGATCTCAAAAATGACGACCTGAATCTCTATTTCTATTTTTTT--ATAAACAAAATCGAAATGATGTGAATCAATTCGAA-----GTTTAAGAAAtaatattcattGATCAAATGATTCTCTTCATAGTCTGATAGATCCGTGGTGGAActtattaaTCGGACGAGAATAAAGATAGAGTCCCATTTTAC-ATGTCAATACTGACAACAATGAAATTTATAGTAAGATGXXXGCTCTACT-CCCCAAAAAGGTCTGTTTGACACCTTACCTTTTT-GTAG-TTATTATCCA-TTTGAATTATTTAGAATCTATATCAGTTTTCATTTTCAAACTTAGAAAGTCTTCTTTTATTTATAAGATCCAAAAAATTCCCGGTCCAAAACTTTTTTAATTTACTACTTTT------GAGTTTCTTTTCATTGACAGAGACCCAAGTCATATATTAAAATGATACTGATacttc--------------------------------------cgtaaTGGTCGGCATAGCTTAATTGCGGAGGACTGAAAATCCTTGTGTCACCATT--------------------------------------------------------------------------------------------AGTAAAATGATAA-TGATACTTCAGTAGATGATACCTCAGTAATGGTGGACATAGC-------TTTTTTG--CAGAGGACTGAAAATC------CTTGTGTCACCATTCGTAAAATGAGGATGATACTTCGGTAATGGCCGGGATAGCTCAgttg

trnLF_GY NNNNNNNNNNNNNNNNNNNNNNNNNNNNNNNNNNNNNNNNNNNNNNAAATTCAGAGAAACCCTGGAATTAAcaatgggcAATCCTGAGCCAAATCCTGGTTTATGTGAACAAACCGGAGTTTAGAAAGCGAGAAAAAA-GGGATAGGTGCAGAGACTCAATGGAAGCTGTTCTAACAAATGGAGTTCACTACCTTGTGTTGATAAAGGAATCCTTCGATCAAAACTTCAAAGAAAAAAG-GATGAAGGAGAAAAACCTATATTGTCTAAATATA------GGTAACACAAAA-CGATCTCAAAAATGACGACCTGAATCTCGATTTCTATTTTTTT--ATAAACAAAATCGAAATGATGTGAATCAATTCGAA-----GTTTAAGAAAtaatattcattGATCAAATGATTCTCTTCATAGTCTGATAGATCCGTGGTGGAActtattaaTCGGACGAGAATAAAGATAGAGTCCCATTTTAC-ATGTCAATACTGACAACAATGAAATTTATAGTAAGATGXXXNCTCTACT-CCCCAAAAAGGTCTGTTTGACACCTTACCTTTTT-GTAG-TTATTATCCA-TTTGAATTATTTAGAATCTACATCAGTTTTCATTTTCAAACTTAGAAAGTCTTCTTTTATTTATAAGATCCAAAAAATTCCCGGTCCAAAACTTTTTTAATTTACTACTTTT------GAGTTTCTTTTCATTGACAGAGACCCAAGTCATATATTAAAATGATACTGATacttc--------------------------------------cgtaaTGGTCGGCATAGCTTAATTGCGGAGGACTGAAAATCCTTGTGTCACCATT--------------------------------------------------------------------------------------------AGTAAAATGATAA-TGATACTTCAGTAGATGATACCTCAGTAATGGTGGACATAGC-------TTTTTTG--CAGAGGACTTAAAATC------CTTGTGTCACCATTCGTAAAATGAGGATGATACTTCGGTAATGGCCGGGATAGCTCAgttg

trnLF_GZ NNNNNNNNNNNNNNNNNNNNNNNNNNCCTACTAAGTGATAACTTTCAAATTCAGAGAAACCCTGGAATTAACAATGGGCAATCCTGAGCCAAATCCTGGTTTATGTGAACAAACCGGAGTTTAGAAAGCGAGAAAAAA-GGGATAGGTGCAGAGACTCAATGGAAGCTGTTCTAACAAATGGAGTTCACTACCTTGTGTTGATAAAGGAATCCTTCGATCAAAACTTCAAAGAAAAAAG-GATGAAGGAGAAAAACCTATATTGTCTAAATATA------GGTAACACAAAA-CGATCTCAAAAATGACGACCTGAATCTCGATTTCTATTTTTTT--ATAAACAAAATCGAAATGATGTGAATCAATTCGAA-----GTTTAAGAAATAATATTCATTGATCAAATGATTCTCTTCATAGTCTGATAGATCCGTGGTGGAACTTATTAATCGGACGAGAATAAAGATAGAGTCCCATTTTAC-ATGTCAATACTGACAACAATGAAATTTATAGTAAGATGXXXGCTCTACT-CCCCAAAAAGGTCTGTTTGACACCTTACCTTTTT-GTAG-TTATTATCCA-TTTGAATTATTTAGAATCTACATCAGTTTTCATTTTCAAACTTAGAAAGTCTTCTTTTATTTATAAGATCCAAAAAATTCCCGGTCCAAAACTTTTTTAATTTACTACTTTT------GAGTTTCTTTTCATTGACAGAGACCCAAGTCATATATTAAAATGATACTGATACTTC--------------------------------------CGTAATGGTCGGCATAGCTTAATTGCGGAGGACTGAAAATCCTTGTGTCACCATT--------------------------------------------------------------------------------------------AGTAAAATGATAA-TGATACTTCAGTAGATGATACCTCAGTAATGGTGGACATAGC-------TTTTTTG--CAGAGGACTGAAAATC------CTTGTGTCACCATTCGTAAAATGAGGATGATACTTCGGTAATGGCCGGGATAGCTCAgttg

trnLF_HA AATTGGATTGAGCCTTGGTATGGAAACCTACTAAGTGATAACTTTCAAATTCAGAGAAACCCTGGAATTAAcaatgggcAATCCTGAGCCAAATCCTGGTTTATGTGAACAAACCGGAGTTTAGAAAGCGAGAAAAAA-GGGATAGGTGCAGAGACTCAATGGAAGCTGTTCTAACAAATGGAGTTCACAACCTTGTGTTGATAAAGGAATCCTTCGATCAAAACTTCAAATAAAAAAG-GATGAAGGAGAAAAACCTATATTGTCTAAATATA------GGTAACACAAAA-CGATCTCAAAAATGACGACCTGAATCTCGATTTCTATTTTTTT--ATAAACAAAATCGAAATGATGTGAATCAATTCGAA-----GTTTAAGAAAtaatattcattGATCAAATGATTCTCTTCATAGTCTGATAGATCCGTGGTGGAActtattaaTCGGACGAGAATAAAGATAGAGTCCCATTTTAC-ATGTCAATACTGACAACAATGAAATTTATAGTAAGATGXXXGCTCTACT-CCCCAAAAAGGTCTGTTTGACACCTTACCTTTTT-GTAG-TTATTATCCA-TTTGAATTATTTAGAATCTATATAAGTTTTCATTTTCAAACTTAGAAAGTCTTCTTTTATTTATAAGATCCAAAAAATTCCCGGTCCAAAACTTTTTTAATTTACTACtttt------gagttTCTTTTCATTGACAGAGACCCAAGTCATATATTAAAATGATACTGATacttc--------------------------------------cgtaaTGGTCGGCATAGCTTAATTGCGGAGGACTGAAAATCCTTGTGTCACCATTAGTAAAATGATAATGT----------------------------------------------------------------------------AGTAAAATGATAA-TGATACTTCAGTAGATTATACCTCAGTAATGGTGGACATAGC-------TTTTTTG--CAGAGGACTGAAAATC------CTTGTGTCACCATTCGTAAAATGAGGATGATACTTCGGTAATGGCCGGGATAGCTCAgttg

trnLF_HB NNNNNNNNNNNNNNNNNNNNNNNNNNCCTACTAAGTGATAACTTTCAAATTCAGCGAAACCCTGGAATTAAcaatgggcAATCCTGAGCCAAATCCTGGTTTATGTGCACAAACCGGAGTTTAGAAAGCGAGAAAAAA-GGGATAGGTGCAGAGACTCAATGGAAGCTGTTCTAACAAATGGAGTTCACTACCTTGTGTTGATAAAGGAATCCTTCGATCAAAACTTCAAATAAAAAAG-GATGAAGGAGAAAAACCTATATTGTCTAAATATA------GGTAACACAAAA-CGATCTCAAAAATGACGACCTGAATCTCGATTTCTATTTTTTT--ATAAACAAAATCGAAATGATGTGAATAAATTCGAA-----GTTTAAGAAAtaatattcattGATCAAATGATTCTCTTCATAGTCTGATAGATCCGTGGTGGAActtattaaTCGGACGAGAATAAAGATAGAGTCCCATTTTAC-ATGTCAATACTGACAACAATGAAATTTATAGTAAGATGXXXGCTCTACT-CCCCAAAAAGGTCTGTTTGCCACCTTACCTTTTT-GTAG-TTATTATCCA-TTTGAATTATTTAGAATCTATATCAGTTTTCATTTTCAAACTTAGAAAGTCTTCTTTTATTTATAAGATCCAAAAAATTCCCGGTCCTAAACTTTTTTAATTTACTACtttt------gagttTCTTTTCATTGACAGAGACCCAAGTCATATATTAAAATGATACTGATacttc--------------------------------------cgtaaTGGTCGGCATAGCTTAATTGCGGAGGACTGAAAATCCTTGTGTCACCATT--------------------------------------------------------------------------------------------------------------------------------------------------------------------------------------------------------CGTAAAATGAGGATGATACTTCGGTAATGGCCGGGATAGCTCAgttg

trnLF_HC AATTGGATTGAGCCTTGGTATGGAAACCTACTAAGTGATAACTTTCAAATTCAGCGAAACCCTGGAATTAAcaatgggcAATCCTGAGCCAAATCCTGGTTTATGTGCACAAACCGGAGTTTAGAAAGCGAGAAAAAA-GGGATAGGTGCAGAGACTCAATGGAAGCTGTTCTAACAAATGGAGTTCACTACCTTGTGTTGATAAAGGAATCCTTCGATCAAAACTTCAAATAAAAAAG-GATGAAGGAGAAAAACCTATATTGTCTAAATATA------GGTAACACAAAA-CGATCTCAAAAATGACGACCTGAATCTCGATTTCTACTTTTTT--ATAAACAAAATCGAAATGATGTGAATAAATTCGAA-----GTTTAAGAAAtaatattcattGATCAAATGATTCTCTTCATAGTCTGATAGATCCGTGGTGGAActtattaaTCGGACGAGAATAAAGATAGAGTCCCATTTTAC-ATGTCAATACTGACAACAATGAAATTTATAGTAAGATGXXXGCTCTACT-CCCCAAAAAGGTCTGTTTGCCACCTTACCTTTTT-GTAG-TTATTATCCA-TTTGAATTATTTAGAATCTATATCAGTTTTCATTTTCAAACTTAGAAAGTCTTCTTTTATTTATAAGATCCAAAAAATTCCCGGTCCTAAACTTTTTTAATTTACTACtttt------gagttTCTTTTCATTGACAGAGACCCAAGTCATATATTAAAATGATACTGATacttc--------------------------------------cgtaaTGGTCGGCATAGCTTAATTGCGGAGGACTGAAAATCCTTGTGTCACCATT--------------------------------------------------------------------------------------------------------------------------------------------------------------------------------------------------------CGTAAAATGAGGATGATACTTCGGTAATGGCCGGGATAGCTCAgttg

trnLF_HX AATTGGATTGAGCCTTGGTATGGAAACCTACTAAGTGATAACTTTCAAATTCAGAGAAACCCTGGAATTAAcaatgggcAATCCTGAGCCAAATCCTGGTTTATGTGAACAAACCGGAGTTTAGAAAGCGAGAAAAAA-GGGATAGGTGCAGAGACTCAATGGAAGCTGTTCTAACAAATGGAGTTCACAACCTTGTGTTGATAAAGGAATCCTTCGATCAAAACTTCAAATAAAAAAG-GATGAAGGAGAAAAACCTATATTGTCTAAATATA------GGTAACACAAAAACGATCTCAAAAATGACGACCTGAATCTCGATTTCTATTTTTTT--ATAAACAAAATCGAAATGATGTGAATCAATTCGAA-----GTTTAAGAAAtaatattcattGATCAAATGATTCTCTTCATAGTCTGATAGATCCGTGGTGGAACTTCTTAATCGGACGAGAATAAAGATAGAGTCCCATTTTAC-ATGTCAATACTGACAACAATGAAATTTATAGTAAGATGXXXGCTCTACT-CCCCAAAAAGGTCTGTTTGACACCTTACCTtttt-gtag-TTATTATCCA-TTTGAATTATTTAGAATCTATATCAGTTTTCATTTTCAAACTTAGAAAGTCTTCTTTTATTTATAAGATCCAAAAAATTCCCGGTCCAAAACTTTTTTAATTTACTACtttt------gagttTCTTTTCATTGACAGAGACCCAAGTCATATATTAAAATGATACTGATacttc--------------------------------------cgtaaTGGTCGGCATAGCTTAATTGCGGAGGACTGAAAATCCTTGTGTCACCATT--------------------------------------------------------------------------------------------AGTAAAATGATAA-TGATACTTCAGTAGATGATACCTCAGTAATGGTGGACATAGC-------TTTTTTG--CAGAGGACTGAAAATC------CTTGTGTCACCATTCGTAAAATGAGGATGATACTTCGGTAATGGCCGGGATAGCTCAgttg

trnLF_HZ AATTGGATTGAGCCTTGGTATGGAAACCTACTAAGTGATAACTTTCAAATTCAGAGAAACCCTGGAATTAAcaatgggcAATCCTGAGCCAAATCCTGGTTTATGTGAACAAACCGGAGTTTAGAAAGCGAGAAAAAA-GGGATAGGTGCAGAGACTCAATGGAAGCTGTTCTAACAAATGGAGTTCACTACCTTGTGTTGATAAAGGAATCCTTCGATCAAAACTTCAAATAAAAAAG-GATGAAGGAGAAAAACCTATATTGTCTAAATATA------GGTAACACAAAA-CGATCTCAAAAATGACGACCTGAATCTCGATTTCTATTTTTTT--ATAAACAAAATCGAAATGATGTGAATCAATTCGAA-----GTTTAAGAAAtaatattcattGATCAAATGATTCTCTTCATAGTCTGATAGATCCGTGGTGGAActtattaaTCGGACGAGAATAAAGATAGAGTCCCATTTTAC-ATGTCAATACTGACAACAATGAAATTTATAGTAAGATGXXXGCTCTACT-CCCCAAAAAGGTCTGTTTGACACCTTACCTtttt-gtag-TTATTATCCA-TTTGAATTATTTAGAATCTACATAAGTTTTCATTTTCAAACTTAGAAAGTCTTCTTTTATTTATAAGATCCAAAAAATTCCCGGTCCAAAACTTTTTTAATTTACTACtttt------gagttTCTTTTCATTGACAGAGACCCAAGTCATATATTAAAATGATACTGATacttc--------------------------------------cgtaaTGGTCGGCATAGCTTAATTGCGGAGGACTGAAAATCCTTGTGTCACCATT--------------------------------------------------------------------------------------------AGTAAAATGATAA-TGATACTTCAGTAGATGATACCTCAGTAATGGTGGACATAGC-------TTTTTTG--CAGAGGACTGAAAATC------CTTGTGTCACCATTCGTAAAATGAGGATGATACTTCGGTAATGGCCGGGATAGCTCAgttg

trnLF_IN AATTGGATTGAGCCTTGGTATGGAAACCTACTAAGTGATAACTTTCAAATTCAGAGAAACCCTGGAATTAAcaatgggcAATCCTGAGCCAAATCCTGGTTTATGTGCACAAACCGGAGTTTAGAAAGCGAGAAAAAA-GGGATAGGTGCAGAGACTCAATGGAAGCTGTTCTAACAAATGGAGTTCACAACCTTGTGTTGATAAAGGAATCCTTCGATCAAAACTTCAAATAAAAAAG-GATGAAGGAGAAAAACCTATATTGTCTAAATATA------GGTAACACAAAA-CGATCTCAAAAATGACGACCTGAATCTCGATTTCTATTTTTTT--ATAAACAAAATCGAAATGATGTGAATCAATTCGAA-----GTTTAAGAAAtaatattcattGATCAAATGATTCTCTTCATAGTCTGATAGATCCGTGGTGGAActtattaaTCGGACGAGAATAAAGATAGAGTCCCATTTTAC-ATGTCAATACTGACAACAATGAAATTTATAGTAAGATGXXXGCTCTACT-CCCCAAAAAGGTCTGTTTGACACCTTACCTtttt-gtag-TTATTATCCA-TTTGAATTATTTAGAATCTATATCAGTTTTCATTTTCAAACTTAGAAAGTCTTCTTTTATTTATAAGATCCAAAAAATTCCCGGTCCAAAACTTTTTTAATTTACTACtttt------gagttTCTTTTCATTGACAGAGACCCAAGTCATATATTAAAATGATACTGATacttc--------------------------------------cgtaaTGGTCGGCATAGCTTAATTGCGGAGGACTGAAAATCCTTGTGTCACCATT--------------------------------------------------------------------------------------------AGTAAAATGATAA-TGATACTTCAGTAGATGATACCTCAGTAATGGTGGACATAGC-------TTTTTTG--CAGAGGACTGAAAATC------CTTGTGTCACCATTCGTAAAATGAGGATGATACTTCGGTAATGGCCGGGATAGCTCAgttg

trnLF_IQ AATTGGATTGAGCCTTGGTATGGAAACCTACTAAGTGATAACTTTCAAATTCAGAGAAACCCTGGAATTAAcaatgggcAATCCTGAGCCAAATCCTGGTTTATGTGAACAAACCGGAGTTTAGAAAGCGAGAAAAAA-GGGATAGGTGCAGAGACTCAATGGAAGCTGTTCTAACAAATGGAGTTCACAACCTTGTGTTGATAAAGGAATCCTTCGATCAAAACTTCAAATAAAAAAG-GATGAAGGAGAAAAACCTATATTGTCTAAATATA------GGTAACACAAAA-CGATCTCAAAAATGACGACCTGAATCTCGATTTCTATTTTTTT--ATAAACAAAATCGAAATGATGTGAATCAATTCGAA-----GTTTAAGAAAtaatattcattGATCAAATGATTCTCTTCATAGTCTGATAGATCCGTGGTGGAActtattaaTCGGACGAGAATAAAGATAGAGTCCCATTTTAC-ATGTCAATACTGACAACAATGAAATTTATAGTAAGATGXXXGCTCTACT-CCCCAAAAAGGTCTGTTTGACACCTTACCTtttt-ttag-TTATTATCCA-TTTGAATTATTTAGAATCTATATCAGTTTTCATTTTCAAACTTAGAAAGTCTTCTTTTATTTATAAGATCCAAGAAATTCCCGGTCCAAAACTTTTTTAATTTACTACtttt------gagttTCTGTTCATTGACAGAGACCCAAGTCATATATTAAAATGATACTGATacttc--------------------------------------cgtaaTGGTCGGCATAGCTTAATTGCGGAGGACTGAAAATCCTTGTGTCACCATT--------------------------------------------------------------------------------------------AGTAAAATGATAC-TGATACTTCAGTAGATGATACCTCAGTAATGGTGGACATAGC-------TTTTTTG--CAGAGGACTGAAAATC------CTTGTGTCACCATTCGTAAAATGAGGATGATACTTCGGTAATGGCCGGGATAGCTCAgttg

trnLF_IR AATTGGATTGAGCCTTGGTATGGAAACCTACTAAGTGATAACTTTCAAATTCAGAGAAACCCTGGAATTAAcaatgggcAATCCTGAGCCAAATCCTGGTTTATGTGAACAAACCGGAGTTTAGAAAGCGAGAAAAAA-GGGATAGGTGCAGAGACTCAATGGAAGCTGTTCTAACAAATGGAGTTCACAACCTTGTGTTGATAAAGGAATCCTTCGATCAAAACTTCAAATAAAAAAG-GATGAAGGAGAAAAACCTATATTGTCTAAATATA------GGTAACACAAAA-CGATCTCAAAAATGACGACCTGAATCTCGATTTCTTTTTTTTT--ATAAACAAAATCGAAATGATGTGAATCAATTCGAA-----GTTTAAGAAAtaatattcattGATCAAATGATTCTCTTCATAGTCTGATAGATCCGTGGTGGAActtattaaTCGGACGAGAATAAAGATAGAGTCCCATTTTAC-ATGTCAATACTGACAACAATGAAATTTATAGTAAGATGXXXGCTCTACT-CCCCAAAAAGGTCTGTTTGACACCTTACCTtttt-ttag-TTATTATCCA-TTTGAATTATTTAGAATCTATATCAGTTTTCATTTTCAAACTTAGAAAGTCTTCTTTTATTTATAAGATCCAAGAAATTCCCGGTCCAAAACTTTTTTAATTTACTACtttt------gagttTCTGTTCATTGACAGAGACCCAAGTCATATATTAAAATGATACTGATacttc--------------------------------------cgtaaTGGTCGGCATAGCTTAATTGCGGAGGACTGAAAATCCTTGTGTCACCATT--------------------------------------------------------------------------------------------AGTAAAATGATAC-TGATACTTCAGTAGATGATACCTCAGTAATGGTGGACATAGC-------TTTTTTG--CAGAGGACTGAAAATC------CTTGTGTCACCATTCGTAAAATGAGGATGATACTTCGGTAATGGCCGGGATAGCTCAgttg

trnLF_IS AATTGGATTGAGCCTTGGTATGGAAACCTACTAAGTGATAACTTTCAAATTCAGAGAAACCCTGGAATTAAcaatgggcAATCCTGAGCCAAATCCTGGTTTATGTGAACAAACCGGAGTTTAGAAAGCGAGAAAAAA-GGGATAGGTGCAGAGACTCAATGGAAGCTGTTCTAACAAATGGAGTTCACAACCTTGTGTTGATAAAGGAATCCTTCGATCAAAACTTCAAATAAAAAAG-GATGAAGGAGAAAAACCTATATTGTCTAAATATA------GGTAACACAAAA-CGATCTCAAAAATGACGACCTGAATCTCGATTTCTATTTTTTT--ATAAACAAAATCGAAATGATGTGAATCAATTCGAA-----GTTTAAGAAAtaatattcattGATCAAATGATTCTCTTCATAGTCTGATAGATCCGTGGTGGAActtattaaTCGGACGAGAATAAAGATAGAGTCCCATTTTAC-ATGTCAATACTGACAACAATGAAATTTATAGTAAGATGXXXGCTCTACT-CCCCAAAAAGGTCCGTTTGACACCTTACCTtttt-ttag-TTATTATCGA-TTTGAATTATTTCGAATCTATATAAGTTTTCATTTTCAAACTTAGAAAGTCTTCTTTTATTTATAAGATCCAAGAAATTCCCGGTCCAAAACTTTTTTAATTTACTACtttt------gagttTCTTTTCATTGACAGAGACCCAAGTCATATATTAAAATGATACTGATacttc--------------------------------------cgtaaTGGTCGGCATAGCTTAATTGCGGAGGACTGAAAATCCTTGTGTCACCATT--------------------------------------------------------------------------------------------AGTAAAATGAGAA-TGATACTTCAGTAGATGATACCTCAGTAATGGTGGACATAGC--TTTTTTTTTTTG--CAGAGGNNNNNNNNNN------NNNNNNNNNNNNNNNNNNNNNNNNNNNNNNNNNNNNNNNNNNNNNNNNNNNNNNNNNnnnn

trnLF_IX AATTGGATTGAGCCTTGGTATGGAAACCTACTAAGTGATAACTTTCAAATTCAGAGAAACCCTGGAATTAAcaatgggcAATCCTGAGCCAAATCCTGGTTTATGTGAACAAACCGGAGTTTAGAAAGCGAGAAAAAA-GGGATAGGTGCAGAGACTCAATGGAAGCTGTTCTAACAAATGGAGTTCACAACCTTGTGTTGATAAAGGAATCCTTCGATCAAAACTTCAAATAAAAAAG-GATGAAGGAGAAAAACCTATATTGTCTAAATATA------GGTAACACAAAA-CGATCTCAAAAATGACGACCTGAATCTCGATTTCTATTTTTTT--ATAAACAAAATCGAAATGATGTGAATCAATTCGAA-----GTTTAAGAAAtaatattcattGATCAAATGATTCTCTTCATAGTCTGATAGATCCGTGGTGGAActtattaaTCGGACGAGAATAAAGATAGAGTCCCATTTTAC-ATGTCAATACTGACAACAATGAAATTTATAGTAAGATGXXXGCTCTACT-CCCCAAAAAGGTCTGTTTGACACCTTACCTtttt-ttag-TTATTATCGA-TTTGAATTATTTAGAATCTATATCAGTTTTCATTTTCAAACTGAGAAAGTCTTCTTTTATTTATAAGATCCAAGAAATTCCCGGTCCAAAACTTTTTTAATTTACTACtttt------gagttTCTTTTCATTGACAGAGACCCAAGTCATATATTAAAATGATACTGATacttc--------------------------------------cgtaaTGGTCGGCATAGCTTAATTGCGGAGGACTGAAAATCCTTGTGTCACCATT--------------------------------------------------------------------------------------------AGTAAAATGAGAA-TGATACTTCAGTAGATGATACCTCAGTAATGGTGGACATAGC--TTTTTTTTTGTG--CAGAGGACTGAAAATC------CTTGTGTCACCATTCGTAAAATAAGGATGATACTTCGGTAATGGCCGGGATAGCTCAgttg

trnLF_KA AATTGGATTGAGCCTTGGTATGGAAACCTACTAAGTGATAACTTTCAAATTCAGAGAAACCCTGGAATTAAcaatgggcAATCCTGAGCCAAATCCTGGTTTATGTGAACAAACCGGAGTTTAGAAAGCGAGAAAAAA-GGGATAGGTGCAGAGACTCAATGGAAGCTGTTCTAACAAATGGAGTTCACAACCTTGTGTTGATAAAGGAATCCTTCGATCAAAACTTCAAATAAAAAAG-GATGAAGGAGAAAAACCTATATTGTCTAAATATA------GGTAACACAAAA-CGATCTCAAAAATGACGACCTGAATCTCTATTTCTATTTTTTT--ATAAACAAAATCGAAATGATGTGAATCAATTCGAA-----GTTTAAGAAAtaatattcattGATCAAATGATTCTCTTCATAGTCTGATAGATCCGTGGTGGAActtattaaTCGGACGAGAATAAAGATAGAGTCCCATTTTAC-ATGTCAATACTGACAACAATGAAATTTATAGTAAGATGXXXGCTCTACT-CCCCAAAACGGTCTGTTTGACACCTTACCTtttt-gtag-TTATTATCCA-TTTGAATTATTTAGAATCTATATCAGTTTTAATTTTCAAACTTAGAAAGTCTTCTTTTATTTATAAGATCCAAAAAATTCCCGGTCCAAAACTTTTTTAATTTACTACtttt------gagttTCTTTTCATTGACAGAGACCCAAGTCATATATTAAAATGATACTGATacttc--------------------------------------cgtaaTGGTCGGCATAGCTTAATTGCGGAGGACTGAAAATCCTTGTGTCACCATT--------------------------------------------------------------------------------------------AGTAAAATGATAA-TGATACTTCAGTAGATGATACCTCAGTAATGGTGGACATAGC-------TTTTTTG--CAGAGGACTGAAAATC------CTTGTGTCACCATTCGTAAAATGAGGATGATACTTCGGTAATGGCCGGGATAGCTCAgttg

trnLF_KC AATTGGATTGAGCCTTGGTATGGAAACCTACTAAGTGATAACTTTCAAATTCAGAGAAACCCTGGAATTAAcaatgggcAATCCTGAGCCAAATCCTGGTTTATGTGAACAAACCGGAGTTTAGAAAGCGAGAAAAAA-GGGATAGGTGCAGAGACTCAATGGAAGCTGTTCTAACAAATGGAGTTCACAACCTTGTGTTGATAAAGGAATCCTTCGATCAAAACTTCAAATAAAAAAG-GATGAAGGAGAAAAACCTATATTGTCTAAATATA------GGTAACACAAAA-CGATCTCAAAAATGACGACCTGAATCTCGATTTCTATTTTTTT--ATAAACAAAATCGAAATGATGTGAATCAATTCGAA-----GTTTAAGAAAtaatattcattGATCAAATGATTCTCTTCATAGTCTGATAGATCCGTGGTGGAActtattaaTCGGACGAGAATAAAGATAGAGTCCCATTTTAC-ATGTCAATACTGACAACAATGAAATTTATAGTAAGATGXXXGCTCTACT-CCCCAAAAAGGTCTGTTTGACACCTTACCTtttt-gtag-TTATTATCCA-TTTGAATTATTTAGAATCTATATCAGTTTTCATTTTCAAACTTAGAAAGTCTTCTTTTATTTATAAGATCCAAAAAATTCCCGGTCCAAAACTTTTTTAATTTACTACtttt------gagttTCTTTTCATTGACAGAGACCCAAGTCATATATTAAAATGATACTGATacttc--------------------------------------cgtaaTGGTCGGCATAGCTTAATTGCGGAGGACTGAAAATCCTTGTGTCACCATT--------------------------------------------------------------------------------------------AGTAAAATGATAA-TGATACTTCAGTAGATGATACCTCAGTAATGGTGGACATAGC-------TTTTTTG--CAGAGGACTGAAAATC------CTTGTGTCACCATTCGTAAAATGAGGATGATACTTCGGTAATGGCCGGGATAGCTCGgttg

trnLF_KD AATTGGATTGAGCCTTGGTATGGAAACCTACTAAGTGATAACTTTCAAATTCAGAGAAACCCTGGAATTAAcaatgggcAATCCTGAGCCAAATCCTGGTTTATGTGAACAAACCGGAGTTTAGAAAGCGAGAGAAAA-GGGATAGGTGCAGAGACTCAATGGAAGCTGTTCTAACAAATGGAGTTCACAACCTTGTGTTGATAAAGGAATCCTTCGATCAAAACTTCAAATAAAAAAG-GATGAAGGAGAAAAACCTATATTGTCTAAATATA------GGTAACACAAAA-CGATCTCAAAAATGACGACCTGAATCTCGATTTCTATTTTTTT--ATAAACAAAATCGAAATGATGTGAATCAATTCGAA-----GTTTAAGAAAtaatattcattGATCAAATGATTCTCTTCATAGTCTGATAGATCCGTGGTGGAActtattaaTCGGACGAGAATAAAGATAGAGTCCCATTTTAC-ATGTCAATACTGACAACAATGAAATTTATAGTAAGATGXXXGCTCTACT-CCCCAAAAAGGTCTGTTTGACACCTTACCTtttt-gtag-TTATTATCCA-TTTGAATTATTTAGAATCTATATCAGTTTTCATTTTCAAACTTAGAAAGTCTTCTTTTATTTATAAGATCCAAAAAATTCCCGGTCCAAAACTTTTTTAATTTACTACtttt------gagttTCTTTTCATTGACAGAGACCCAAGTCATATATTAAAATGATACTGATacttc--------------------------------------cgtaaTGGTCGGCATAGCTTAATTGCGGAGGACTGAAAATCCTTGTGTCACCATT--------------------------------------------------------------------------------------------AGTAAAATGATAA-TGATACTTCAGTAGATGATACCTCAGTAATGGTGGACATAGC-------TTTTTTG--CAGAGGACTGAAAATC------CTTGTGTCACCATTCGTAAAATGAGGATGATACTTCGGTAATGGCCGGGATAGCTCAgttg

trnLF_KF AATTGGATTGAGCCTTGGTATGGAAACCTACTAAGTGATAACTTTCAAATTCAGAGAAACCCTGGAATTAAcaatgggcAATCCTGAGCCAAATCCTGGTTTATGTGAACAAACCGGAGTTTAGAAAGCGAGAAAAAA-GGGATAGGTGCAGAGACTCAATGGAAGCTGTTCTAACAAATGGAGTTCACTACCTTGTGTTGATAAAGGAATCCTTCGATCAAAACTTCAAATAAAAAAG-GATGAAGGAGAAAAACCTATATTGTCTAAATATA------GGTAACACAAAA-CGATCTCAAAAATGACGACCTGAATCTCGATTTCTATTTTTTT--ATAAACAAAATCGAAATGATGTGAATCAATTCGAA-----GTTTAAGAACtaatattcattGATCAAATGATTCTCTTCATAGTCTGATAGATCCGTGGTGGAActtattaaTCGGACGAGAATAAAGATAGAGTCCCATTTTAC-ATGTCAATACTGACAACAATGAAATTTATAGTAAGATGXXXGCTCTACT-CCCCAAAAAGGTCTGTTTGACACCTTACCTtttt-gtag-TTATTATCCA-TTTGAATTATTTAGAATCTATATCAGTTTTCATTTTCAAACTTAGAAAGTCTTCTTTTATTTATAAGATCCAAAAAATTCCCGGTCCAAAACTTTTTTAATTTACTACtttt------gagttTCTTTTCATTGACAGAGACCCAAGTCATATATTAAAATGATACTGATacttc--------------------------------------cgtaaTGGTCGGCATAGCTTAATTGCGGAGGACTGAAAATCCTTGTGTCACCATT--------------------------------------------------------------------------------------------------------------------------------------------------------------------------------------------------------CGTAAAATGAGGATGATACTTCGGTAATGGCCGGGATAGCTCAgttg

trnLF_KG AATTGGATTGAGCCTTGGTATGGAAACCTACTAAGTGATAACTTTCAAATTCAGAGAAACCCTGGAATTAACAATGGGCAATCCTGAGCCAAATCCTGGTTTATGTGAACAAACCGGAGTTTAGAAAGCGAGAAAAAA-GGGATAGGTGCAGAGACTCAATGGAAGCTGTTCTAACAAATGGAGTTCACTACCTTGTGTTGATAAAGGAATCCTTCGATCAAAACTTCAAATAAAAAAG-GATGAAGGAGAAAAACCTATATTGTCTAAATATA------GGTAACACAAAA-CGATCTCAAAAATGACGACCTGAATCTCGATTTCTATTTTTTT--ATAAACAAAATCGAAATGATGTGAATCAATTCGAA-----GTTTAAGAACTAATATTCATTGATCAAATGATTCTCTTCATAGTCTGATAGATCCGTGGTGGAACTTATTAATCGGACGAGAATAAAGATAGAGTCCCATTTTAC-ATGTCAATACTGACAACAATGAAATTTATAGTAAGATGXXXGCTCTACT-CCCCAAAAAGGTCTGTTTGACACCTTACCTTTTT-GTAG-TTATTATCCA-TTTGAATTATTTAGAATCTATATCAGTTTTCATTTTCAAACTTAGAAAGTCTTCTTTTATTTATAAGATCCAAAAAATTCCCGGTCCAAAACTTTTTTAATTTACTACTTTT------GAGTTTCTTTTCATTGACAGAGACCCAAGTCATATATTAAAATGATACTGATACTTC--------------------------------------CGTAATGGTCGGCATAGCTTAATTGCGGAGGACTGAAAATCCTTGTGTCACCATT--------------------------------------------------------------------------------------------------------------------------------------------------------------------------------------------------------CGTAAAATGAGGATGATACTTCGGTAATGGCCGGGATAGCTCAgttg

trnLF_KH AATTGGATTGAGCCTTGGTATGGAAACCTACTAAGTGATAACTTTCAAATTCAGAGAAACCCTGGAATTAACAATGGGCAATCCTGAGCCAAATCCTGGTTTATGTGAACAAACCGGAGTTTAGAAAGCGAGAAAAAA-GGGATAGGTGCAGAGACTCAATGGAAGCTGTTCTAACAAATGGAGTTCACAACCTTGTGTTGATAAAGGAATCCTTCGATCAAAACTTCAAATAAAAAAG-GATGAAGGAGAAAAACCTATATTGTCTAAATATA------GGTAACACAAAA-CGATCTCAAAAATGACGACCTGAATCTCGATTTCTATTTTTTT--ATAAACAAAATCGAAATGATGTGAATCAATTCGAA-----GTTTAAGAAATAATATTCATTGATCAAATGATTCTCTTCATAGTCTGATAGATCCGTGGTGGAACTTATTAATCGGACGAGAATAAAGATAGAGTCCCATTTTAC-ATGTCAATACTGACAACAATGAAATTTATAGTAAGATGXXXGCTCTACT-CCCCAAAAAGGTCTGTTTGACACCTTACCTTTTT-GTAG-TTATTATCCA-TTTGAATTATTTAGAATCTATATCAGTTTTCATTTTCAAACTTAGAAAGTCTTCTTTTATTTATAAGATCCAAAAAATTCCCGGTCCAAAACTTTTTTAATTTACTACTTTT------GAGTTTCTTTTCATTGACAGAGACCCAAGTCATATATTAAAATGATACTGATACTTC--------------------------------------CGTAATGGTCGGCATAGCTTAATTGCGGAGGACTGAAAATCCTTGTGTCACCATT--------------------------------------------------------------------------------------------AGTAAAATGATAA-TGATACTTCAGTAGATGATACCTCAGTAATGGTGGACATAGC-------TTTTTTG--CAGAGGACTGAAAATC------CTTGTGTCACCATTCGTAAAATGAGGATGnnnnnnnnnnnnnnnnnnnnnnnnnnnnnnnn

trnLF_LN AATTGGATTGAGCCTTGGTATGGAAACCTACTAAGTGATAACTTTCAAATTCAGAGAAACCCTGGAATTAACAATGGGCAATCCTGAGCCAAATCCTGGTTTATGTGAACAAACCGGAGTTTAGAAAGCGAGAAAAAA-GGGATAGGTGCAGAGACTCAATGGAAGCTGTTCTAACAAATGGAGTTCACTACCTTGTGTTGATAAAGGAATCCTTCGATCAAAACTTCAAATAAAAAAG-GATGAAGGAGAAAAACCTATATTGTCTAAATATA------GGTAACACAAAA-CGATCTCAAAAATGACGACCTGAATCTCGATTTCTACTTTTTT--ATAAACAAAATCGAAATGATGTGAATAAATTCGAA-----GTTTAAGAAATAATATTCATTGATCAAATGATTCTCTTCATAGTCTGATAGATCCGTGGTGGAACTTATTAATCGGACGAGAATAAAGATAGAGTCCCATTTTAC-ATGTCAATACTGACAACAATGAAATTTATAGTAAGATGXXXGCTCTACT-CCCCAAAAAGGTCTGTTTGACACCTTACCTTTTTTTTAG-TTATTATCCA-TTTGAATTATTTAGAATCTATATCAGTTTTCATTTTCAAACTTAGAAAGTCTTCTTTTATTTATAAGATCCAAAAAATTCCCGGTCCTAAACTTTTTTAATTTACTACTTTT------GAGTTTCTTTTCATTGACAGAGACCCAAGTCATATATTAAAATGATACTGATACTTC--------------------------------------CGTAATGGTCGGCATAGCTTAATTGCGGAGGACTGAAAATCCTTGTGTCACCATT--------------------------------------------------------------------------------------------------------------------------------------------------------------------------------------------------------CGTAAAATGAGGATGANNNNNNNNNNNNNNNNNNNNNNNNNNNnnnn

trnLF_EI AATTGGATTGAGCCTTGGTATGGAAACCTACTAAGTGATAACTTTCAAATTCAGAGAAACCCTGGAATTAAcaatgggcAATCCTGAGCCAAATCCTGGTTTACGTGAACAAACCGGAGTTTAGAAAGCGAGAAAAAA-GGGATAGGTGCAGAGACTCAATGGAAGCTGTTCTAACAAATGGAGTTCACTACCTTGTGTTGATAAAGGAATCCTTCGATCAAAACTTCAAATCAAAAAG-GATGAAGGAGAAAAACCTATATTGTCTAAATATA------GGTAACACAAAA-CGATCTAAAAAATGACGACCTGAATCTCGATTTCTATTTTTTT--ATAAACAAAATCGAAATAATGTGAATCAATTCGAA-----GTTTAAGAAAtaatattcattGATCAAATGATTCTCTTCATAGTCTGATAGATCCGTGGTGGAActtattaaTCGGCCGAGAATAAAGATAGAGTCCCATTTTAC-ATGTCAATACTGACAACAATGAAATTTATAGTAAGATGXXXGCTCTACT-CCCCAAAAAGGTCTGTTTGACACCTTACCTtttt-ttag-TTATTATCCA-TTTGAATTATTTAGAATCTATATCAGTTTTCATTTTCAAACTTAGAAAGTCTTCTTTTATTTATAAGATCCAAGAAATTCCCGGTCCAAAACTTTTTTAATTTACTACtttt------gagttTCTTTTCATTGACAGAGACCCAAGTCATATATTAAAATGATACTGATACTCC--------------------------------------CGTAATGGTCGGCATAGCTTAATTGCGGAGGACTGAAAATCCTTGTGTCACCATT--------------------------------------------------------------------------------------------------------------------------------------------------------------------------------------------------------AGTAAAATGATACTGATACTTCGGTAATGGCCGGGATAGCTCAgttg

trnLF_EK AATTGGATTGAGCCTTGGTATGGAAACCTACTAAGTGATAACTTTCAAATTCAGAGAAACCCTGGAATTAAcaatgggcAATCCTGAGCCAAATCCTGGTTTACGTGAACAAACCGGAGTTTAGAAAGCGAGAAAAAA-GGGATAGGTGCAGAGACTCAATGGAAGCTGTTCTAAAAAATGGAGTTCACTACCTTGTGTTGATAAAGGAATCCTTCGATCAAAACTTCAAATCAAAAAG-GATGAAGGAGAAAAACCTATATTGTCTAAATATA------GGTAACACAAAA-CGATCTAAAAAATGACGACCTGAATCTCGATTTCTATTTTTTT--ATAAACAAAATCGAAATAATGTGAATCAATTCGAA-----GTTTAAGAAAtaatattcattGATCAAATGATTCTCTTCATAGTCTGATAGATCCGTGGTGGAActtattaaTCGGCCGAGAATAAAGATAGAGTCCCATTTTAC-ATGTCAATACTGACAACAATGAAATTTATAGTAAGATGXXXNCTCTACT-CCCCAAAAAGGTCTGTTTGACACCTTACCTtttt-ttag-TTATTATCCA-TTTGAATTATTTAGAATCTATATCAGTTTTCATTTTCAAACTTAGAAAGTCTTCTTTTATTTATAAGATCCAAGAAATTCCCGGTCCAAAACTTTTTTAATTTACTACtttt------gagttTCTTTTCATTGACAGAGACCCAAGTCATATATTAAAATGATACTGATACTCC--------------------------------------CGTAATGGTCGGCATAGCTTAATTGCGGAGGACTGAAAATCCTTGTGTCACCATT--------------------------------------------------------------------------------------------------------------------------------------------------------------------------------------------------------AGTAAAATGATACTGATACTTCGGTAATGGCCGGGATAGCTCAgttg

trnLF_FV AATTGGATTGAGCCTTGGTATGGAAACCTACTAAGTGATAACTTTCAAATTCAGAGAAACCCTGGAATTAAcaatgggcAATCCTGAGCCAAATCCTGGTTTACGTGAACAAACCGGAGTTTAGAAAGCGAGAAAAAA-GGGATAGGTGCAGAGACTCAATGGAAGCTGTTCTAACAAATGGAGTTCACTACCTTGTGTTGATAAAGGAATCCTTCGATCAAAACTTCAAATCAAAAAG-GATGAAGGAGAAAAACCTATATTGTCTAAATATA------GGTAACACAAAA-CGATCTAAAAAATGACGACCTGAATCTCGATTTCTATTTTTTT--ATAAACAAAATCGAAATAATGTGAATCAATTCGAA-----GTTTAAGAAAtaatattcattGATCAAATGATTCTCTTCATAGTCTGATAGATCCGTGGTGGAActtattaaTCGGCCGAGAATAAAGATAGAGTCCCATTTTAC-ATGTCAATACTGACAACAATGAAATTTATAGTAAGATGXXXGCTCTACT-CCCAAAAAAGGTCTGTTTGACACCTTACCTtttt-ttag-TTATTATCCA-TTTGAATTATTTAGAATCTATATCAGTTTTCATTTTCAAACTTAGAAAGTCTTCTTTTATTTATAAGATCCAAGAAATTCCCGGTCAAAAACTTTTTTAATTTACTACtttt------gagttTCTTTTCATTGACAGAGACCCAAGTCATATAGTAAAATGATACTGATACTCC--------------------------------------CGTAATGGTCGGCATAGCTTAATTGCGGAGGACTGAAAATCCTTGTGTCACCATT--------------------------------------------------------------------------------------------------------------------------------------------------------------------------------------------------------AGTAAAATGATACTGATACTTCGGTAATGGCCGGGATAGCTCAgttg

trnLF_FU AATTGGATTGAGCCTTGGTATGGAAACCTACTAAGTGATAACTTTCAAATTCAGAGAAACCCTGGAATTAAcaatgggcAATCCTGAGCCAAATCCTGGTTTACGTGAACAAACCGGAGTTTAGAAAGCGAGAAAAAA-GGGATAGGTGCAGAGACTCAATGGAAGCTGTTCTAACAAATGGAGTTCACTACCTTGTGTTGATAAAGGAATCCTTCGATCAAAACTTCAAATCAAAAAG-GATGAAGGAGAAAAACCTATATTGTCTAAATATA------GGTCACACAAAA-CGATCTAAAAAATGACGACCTGAATCTCGATTTCTATTTTTTT--ATAAACAAAATCGAAATAATGTGAATCAATTCGAA-----GTTTAAGAAAtaatattcattGATCAAATGATTCTCTTCATAGTCTGATAGATCCGTGGTGGAActtattaaTCGGCCGAGAATAAAGATAGAGTCCCATTTTAC-ATGTCAATACTGACAACAATGAAATTTATAGTAAGATGXXXGCTCTACT-CCCAAAAAAGGTCTGTTTGACACCTTACCTtttt-ttag-TTATTATCCA-TTTGAATTATTTAGAATCTATATCAGTTTTCATTTTCAAACTTAGAAAGTCTTCTTTTATTTATAAGATCCAAGAAATTCCCGGTCAAAAACTTTTTTAATTTACTACtttt------gagttTCTTTTCATTGACAGAGACCCAAGTCATATATTAAAATGATACTGATACTCC--------------------------------------CGTAATGGTCGGCATAGCTTAATTGCGGAGGACTGAAAATCCTTGTGTCACCATT--------------------------------------------------------------------------------------------------------------------------------------------------------------------------------------------------------AGTAAAATGATACTGATACTTCGGTAATGGCCGGGATAGCTCAgttg

trnLF_FX AATTGGATTGAGCCTTGGTATGGAAACCTACTAAGTGATAACTTTCAAATTCAGAGAAACCCTGGAATTAACAATGGGCAATCCTGAGCCAAATCCTGGTTTACGTGAACAAACCGGAGTTTAGAAAGCGAGAAAAAA-GGGATAGGTGCAGAGACTCAATGGAAGCTGTTCTAACAAATGGAGTTCACTACCTTGTGTTGATAAAGGAATCCTTCGATCAAAACTTCAAATCAAAAAG-GATGAAGGAGAAAAACCTATATTGTCTAAATATA------GGTCACACAAAA-CGATCTAAAAAATGACGACCTGAATCTCGATTTCTATTTTTTT--ATAAACAAAATCGAAATAATGTGAATCAATTCGAA-----GTTTAAGAAATAATATTCATTGATCAAATGATTCTCTTCATAGTCTGATAGATCCGTGGTGGAACTTATTAATCGGCCGAGAATAAAGATAGAGTCCCATTTTAC-ATGTCAATACTGACAACAATGAAATTTATAGTAAGATGXXXGCTCTACT-CCCAAAAAAGGTCTGTTTGACACCTTACCTTTTT-TTAG-TTATTATCCA-TTTGAATTATTTAGAATCTATATCAGTTTTCATTTTCAAACTTAGAAAGTCTTCTTTTATTTATAAGATCCAAGAAATTCCCGGTCAAAAACTTTTTTAATTTACTACTTTT------GAGTTTCTTTTCATTGACAGAGACCCAAGTCATATATTAAAATGATACTGATACTTC---------------------------------------------------------------------------------------------------------------------------------------------------------------------------------------------------------------------------------------------------------------------------------------------------------------------------GGTAATGGCCGGGATAGCTCAgttg

trnLF_IB AATTGGATTGAGCCTTGGTATGGAAACCTACTAAGTGATATCTTTCAAATTCAGAGAAACCCTGGAATTAAcaatgggcAATCCTGAGCCAAATCCTGGTTTACGTGAACAAACCGGAGTTTAGAAAGCGAGAAAAAA-GGGATAGGTGCAGAGACTCAATGGAAGCTGTTCTAACAAATGGAGTTCACTACCTTGTGTTGATAAAGGAATCCTTCGATCAAAACTTCAAATCAAAAAG-GATGAAGGAGAAAAATCTATATTGTCTAAATATA------GGTAACACAAAA-CGATCTCAAAAATGACGACCTGAATCTCGATTTCTATTTTTTT--ATAAACAAAATCGAAATGATGTGAATCAATTCGAA-----GTTTAAGAAAtaatattcattGATCAAATGATTCTCTTCATAGTCTGATAGATCCGTGGTGGAActtattaaTCGGACGAGAATAAAGATAGAGTCCCATTTTAC-ATGTCAATACTGACAACAATGAAATTTATAGTAAGATGXXXGCTCTACT-CCCCAAAAAGGTCTGTTTGACACCTTACCTtttt-ttag-TTATTATCCA-TTTGAATTATTTAGAATCTATATCAGTTTTCATTTTCAAACTTAGAAAGTCTTCTTTTATTTATAAGATCCAAGAAATTCCCGGTCCAAAACTTTTTTAATTTACTACtttt------gagttTCTTTTCATTGACAGAGACCCAAGTCATATATTAAAATGATACTGATacttc--------------------------------------cgtaaTGGTCGGCATAGCTTAATTGCGGAGGACTGAAAATCCTTGTGTCACCATT--------------------------------------------------------------------------------------------AGTAAAATGATAA-TGATACTTCAGTAGATGATACCTCAGTAATGGTGGACATAGC-------TTTTTTG--CAGAGGACTGAAAATC------CTTGTGTCACCATTCGTAAAATGAGGATGATACTTCGGTAATGGCCGGGATAGCTCAgttg

trnLF_EF AATTGGATTGAGCCTTGGTATGGAAACCTACTAAGTGATAACTTTCAAATTCAGAGAAACCCTGGAATTAAcaatgggcAATCCTGAGCCAAATCCTGGTTTACGTGAACAAACCGGAGTTTAGAAAGCGAGAAAAAA-GGGATAGGTGCAGAGACTCAATGGAAGCTGTTCTAACAAATGGAGTTCACTACCTTGTGTTGATAAAGGAATCCTTCGATCAAAACTTCAAATCAAAAAG-GATGAAGGAGAAAAATCTATATTGTCTAAATATA------GGTAACACAAAA-CGATCTCAAAAATGACGACCTGAATCTCGATTTCTATTTTTTT--ATAAACAAAATCGAAATGATGTGAATCAATTCGAA-----GTTTAAGAAAtaatattcattGATCAAATGATTCTCTTCATAGTCTGATAGATCCGTGGTGGAActtattaaTCGGACGAGAATAAAGATAGAGTCCCATTTTAC-ATGTCAATACTGACAACAATGAAATTTATAGTAAGATGXXXGCTCTACT-CCCCAAAAAGGTCTGTTTGACACCTTACCTtttt-ttag-TTATTATCCA-TTTGAATTATTTAGAATCTATATCAGTTTTCATTTTCAAACTTAGAAAGTCTTCTTTTATTTATAAGATCCAAGAAATTCCCGGTCCAAAACTTTTTTAATTTACTACtttt------gagttTCTTTTCATTGACAGAGACCCAAGTCATATATTAAAATGATACTGATacttc--------------------------------------cgtaaTGGTCGGCATAGCTTAATTGCGGAGGACTGAAAATCCTTGTGTCACCATT--------------------------------------------------------------------------------------------AGTAAAATGATAA-TGATACTTCAGTAGATGATACCTCAGTAATGGTGGACATAGC-------TTTTTTG--CAGAGGACTGAAAATC------CTTGTGTCACCATTCGTAAAATGAGGATGATACTTCGGTAATGGCCGGGATAGCTCAgttg

trnLF_FG AATTGGATTGAGCCTTGGTATGGAAACCTACTAAGTGATAACTTTCAAATTCAGAGAAACCCTGGAATTAACAATGGGCAATCCTGAGCCAAATCCTGGTTTACGTGAACAAACCGGAGTTTAGAAAGCGAGAAAAAA-GGGATAGGTGCAGAGACTCAATGGAAGCTGTTCTAACAAATGGAGTTCACTACCTTGTGTTGATAAAGGAATCCTTCGATCAAAACTTCAAATCAAAAAG-GATGAAGGAGAAAAATCTATATTGTCTAAATATA------GGTAACACAAAA-CGATCTCAAAAATGACGACCTGAATCTCGATTTCTATTTTTTT--ATAAACAAAATCGAAATGATGTGAATCAATTCGAA-----GTTTAAGAAATAATATTCATTGATCAAATGATTCTCTTCATAGTCTGATAGATCCGTGGTGGAACTTATTAATCGGACGAGAATAAAGATAGAGTCCCATTTTAC-ATGTCAATACTGACAACAATGAAATTTATAGTAAGATGXXXGCTCTACT-CCCCAAAAAGGTCTGTTTGACACCTTACCTTTTT-TTAG-TTATTATCCA-TTTGAATTATTTAGAATCTATATCAGTTTTCATTTTCAAACTTAGAAAGTCTTCTTTTATTTATAAGATCCAAGAAATTCCCGGTCCAAAACTTTTTTAATTTACTACTTTT------GAGTTTCTTTTCATTGACAGAGACCCAAGTCATATATTAAAATGATACTGATACTTC--------------------------------------CGTAATGGTCGGCATAGCTTAATTGCGGAGGACTGAAAATCCTTGTGTCACCATT--------------------------------------------------------------------------------------------AGTAAAATGATAA-TGATACTTCAGTAGATGATACCTCAGTAATGGTGGACATAGC-------TTTTTTG--CAGAGGACTGAAAATC------CTTGTGTCACCATTCGTAAAATGAGGATGATACTTCGGTAATGGCCGGGATAGCTCAgttg

trnLF_FL AATTGGATTGAGCCTTGGTATGGAAACCTACTAAGTGATAACTTTCAAATTCAGAGAAACCCTGGAATTAACAATGGGCAATCCTGAGCCAAATCCTGGTTTACGTGAACAAACCGGAGTTTAGAAAGCGAGAAAAAA-GGGATAGGTGCAGAGACTCAATGGAAGCTGTTCTAACAAATGGAGTTCACTACCTTGTGTTGATAAAGGAATCCTTCGATCAAAACTTCAAATCAAAAAG-GATGAAGGAGAAAAATCTATATTGTCTAAATATA------GGTAACACAAAA-CGATCTCAAAAATGACGACCTGAATCTCGATTTCTATTTTTTT--ATAAACAAAATCGAAATGATGTGAATCAATTCGAA-----GTTTAAGAAATAATATTCATTGATCAAATGATTCTCTTCATAGTCTGATAGATCCGTGGTGGAACTTATTAATCGGACGAGAATAAAGATAGAGTCCCATTTTAC-ATGTCAATACTGACAACAATGAAATTTATAGTAAGATGXXXGCTCTACT-CCCCAAAAAGGTCTGTTTGACACCTTACCTTTTT-TTAG-TTATTATCCA-TTTGAATTATTTAGAATCTATATCAGTTTTCATTTTCAAACTTAGAAAGTCTTCTTTTATTTATAAGATCCAAGAAATTCCCGGTCCAAAACTTTTTGAATTTACTACTTTT------GAGTTTCTTTTCATTGACAGAGACCCAAGTCATATATTAAAATGATACTGATACTTC--------------------------------------CGTAATGGTCGGCATAGCTTAATTGCGGAGGACTGAAAATCCTTGTGTCACCATT--------------------------------------------------------------------------------------------AGTAAAATGATAA-TGATACTTCAGTAGATGATACCTCAGTAATGGTGGACATAGC-------TTTTTTG--CAGAGGACTGAAAATC------CTTGTGTCCTCATTCGTAAAATGAGGATGATACTTCGGTAATGGCCGGGATAGCTCNnnnn

trnLF_HM AATTGGATTGAGCCTTGGTATGGAAACCTACTAAGTGATAACTTTCAAATTCAGAGAAACCCTGGAATTAAcaatgggcAATCCTGAGCCAAATCCTGGTTTACGTGAACAAACCGGAGTTTAGAAAGCGAGAAAAAA-GGGATAGGTGCAGAGACTCAATGGAAGCTGTTCTAACAAATGGAGTTCACTACCTTGTGTTGATAAAGGAATCCTTCGATCAAAACTTCAAATCAAAAAG-GATGAAGGAGAAAAATCTATATTGTCTAAATATA------GGTAACACAAAA-CGATCTCAAAAATGACGACCTGAATCTCGATTTCTATTTTTTT--ATAAACAAAATCGAAATGATGTGAATCAATTCGAA-----GTTTAAGAAAtaatattcattGATCAAATGATTCTCTTCATAGTCTGATAGATCCGTGGTGGAActtattaaTCGGACGAGAATAAAGATAGAGTCCCATTTTAC-ATGTCAATACTGACAACAATGAAATTTATAGTAAGATGXXXGCTCTACT-CCCCAAAAAGGTCTGTTTGACACCTTACCTtttt-ttag-TTATTATCCA-TTTGAATTATTTAGAATCTATATAAGTTTTCATTTTCAAACTTAGAAAGTCTTCTTTTATTTATAAGATCCAAGAAATTCCCGGTCCAAAACTTTTTGAATTTACTACtttt------gagttTCTTTTCATTGACAGAGACCCAAGTCATATATTAAAATGATACTGATacttc--------------------------------------cgtaaTGGTCGGCATAGCTTAATTGCGGAGGACTGAAAATCCTTGTGTCACCATT--------------------------------------------------------------------------------------------AGTAAAATGATAA-TGATACTTCAGTAGATGATACCTCAGTAATGGTGGACATAGC-------TTTTTTG--CGGAGGACTGAAAATC------CTTGTGTCCTCATTCGTAAAATGAGGATGATACTTCGGTAATGGCCGGGATAGCTCAgttg

trnLF_GH NNNNNNNNNNNNNNNNNNNNNNNNNNNNNNNNNNNNNNNNNNNNNNNNNNNNNNNGAAACCCTGGAATTAAcaatgggcAATCCTGAGCCAAATCCTGGTTTACGTGAACAAACCGGAGTTTAGAAAGCGAGAAAAAA-GGGATAGGTGCAGAGACTCAATGGAAGCTGTTCTAACAAATGGAGTTCACTACCTTGTGTTGATAAAGGAATCCTTCGATCAAAACTTCAAATCAAAAAG-GATGAAGGAGAAAAATCTATATTGTCTAAATATA------GGTAACACAAAA-CGATCTCAAAAATGACGACCTGAATCTCGATTTCGATTTTTTT--ATAAACAAAATCGAACTGATGTGAATCAATTCGAA-----GTTTACGAAAtaatattcattGATCAAATGATTCTCTTCATAGTCTGATAGATCCGTGGTGTAActtattaaTCGGACGAGAATAAAGATAGAGTCCCATTTTAC-ATGTCAATACTGACAANNNNNNNNNNNNNNNNNNNNNNXXXNCTCTACT-CCCCAAAAAGGTCTGTTTGACACCTTACCTtttt-ttag-TTATTATCCA-TTTGAATTATTTAGAATCTATATCAGTTTTCATTTTCAAACTTAGAAAGTCTTCTTTTATTTATAAGATCCAAGAAATTCCCGGTCCAAAACTTTTTTAATTTACTACtttt------gagttTCTTTTCATTGACAGAGACCCAAGTCATATATTAAAATGATACTGATacttc--------------------------------------cgtaaTGGTCGGCATAGCTTAATTGCGGAGGACTGAAAATCCTTGTGTCACCATT--------------------------------------------------------------------------------------------AGTAAAATGATAA-TGATACTTCAGTAGATGATACCTCAGTAATGGTGGACATAGC-------TTTTTTG--CAGAGGACTGAAAATC------CTTGTGTCACCATTCGTAAAATGAGGATGATACTTCGGTAATGGCCGGGATAGCTCAgttg

AY257717 AATTGGATTGAGCCTTGGTATGGAAACCTACTAAGTGATAACTTTCAAATTCAGAGAAACCCTGGAATTAAcaatgggcAATCCTGAGCCAAATCCTGGTTTACGTGAACAAACCGGAGTTTAGAAAGCGAGAAAAAA-GGGATAGGTGCAGAGACTCAATGGAAGCTGTTCTAACAAATGGAGTTCACTACCTTGTGTTGATAAAGGAATCCTTCGATCAAAACTTCAAATCAAAAAG-GATGAAGGAGAAAAATCTATATTGTCTAAATATA------GGTAACACAAAA-CGATCTCAAAAATGACGACCTGAATCTCGATTTCGATTTTTTT--ATAAACAAAATCGAAATGATGTGAATCAATTCGAA-----GTTTACGAAAtaatattcattGATCAAATGATTCTCTTCATAGTCTGATAGATCCGTGGTGGAActtattaaTCGGACGAGAATAAAGATAGAGTCCCATTTTAC-ATGTCAATACTGACAACAATGAAATTTATAGTAAGATGXXXGCTCTACT-CCCAAAAAAGGTCTGTTTGACACCTTACCTtttt-ttag-TTATTATCCA-TTTGAATTATTTAGAATCTATATCAGTTTTCATTTTCAAACTTAGAAAGTCTTCTTTTATTTATAAGATCCAAGAAATTCCCGGTCCAAAACTTTTTTAATTTACTACtttt------gagttTCTTTTCATTGACAGAGACCCAAGTCATATATTAAAATGATACTGATacttc--------------------------------------cgtaaTGGTCGGCATAGCTTAATTGCGGAGGACTGAAAATCCTTGTGTCACCATT--------------------------------------------------------------------------------------------AGTAAAATGATAA-TGATACTTCAGTAGATGATACCTCAGTAATGGTGGACATAGC-------TTTTTTG--CAGAGGACTGAAAATC------CTTGTGTCACCATTCGTAAAATGAGGATGATACTTCGGTAATGGCCGGGATAGCTCAgttg

AY257756 AATTGGATTGAGCCTTGGTATGGAAACCTACTAAGTGATAACTTTCAAATTCAGAGAAACCCTGGAATTAAcaatgggcAATCCTGAGCCAAATCCTGGTTTACGTGAACAAACCGGAGTTTAGAAAGCGAGAAAAAA-GGGATAGGTGCAGAGACTCAATGGAAGCTGTTCTAACAAATGGAGTTCACTACCTTGTGTTGATAAAGGAATCCTTCGATCAAAACTTCAAATCAAAAAG-GTTGAAGGAGAAAAACCTATATTGTCTAAATATA------GGTAACACAAAA-CGATCTCAAAAATGACGACCTGAATCTCGATTTCTATTTTTTT--ATAAACAAAATCGAAATGATGTGAATCAATTCGAA-----GTTTAAGAAAtaatattcattGATCAAATGATTCTCTTCATAGTCTGATAGATCCGTGGTGGAActtattaaTCGGCCGAGAATAAAGATAGAGTCCCATTTTAC-ATGTCAATACTGACAACAATGAAATTTATAGTAAGATGXXXGCTCTACT-CCCCAAAAAGGTCTGTTTGACACCTTACCTtttt-ttag-TTATTATCCA-TTTGAATTATTTAGAATCTATATCAGTTTTCATTTTCAAACTTAGAAAGTCTTCTTTTATTTATAAGATCCAAGAAATTCCCGGTCCAAAACTTTTTTAATTTACTACtttt------gagttTCTTTTCATTGACAGAGACCCAAGTCATATATTAAAATGATACTGATacttc--------------------------------------cgtaaTGGTCGGCATAGCTTAATTGCGGAGGACTGAAAATCCTTGTGTCACCATT--------------------------------------------------------------------------------------------AGTAAAA-------TGATACTTCAGTAGATGATACCTCAGTAATGGTGGACATAGC-------TTTTTTG--CAGAGGACTGAAAATC------CTTGTGTCACCATTCGTAAAATGAGGATGATACTTCGGTAATGGCCGGGATAGCTCAgttg

AY257757 AATTGGATTGAGCCTTGGTATGGAAACCTACTAAGTGATAACTTTCAAATTCAGAGAAACCCTGGAATTAAcaatgggcAATCCTGAGCCAAATCCTGGTTTACGTGAACAAACCGGAGTTTAGAAAGCGAGAAAAAA-GGGATAGGTGCAGAGACTCAATGGAAGCTGTTCTAACAAATGGAGTTCACTACCTTGTGTTGATAAAGGAATCCTTCGATCAAAACTTCAAATCAAAAAG-GTTGAAGGAGAAAAACCTATATTGTCTAAATATA------GGTAACACAAAA-CGATCTCAAAAATGACGACCTGAATCTCGATTTCTATTTTTTT--ATAAACAAAATCGAAATGATGTGAATCAATTCGAA-----GTTTAAGAAAtaatattcattGATCAAATGATTCTCTTCATAGTCTGATAGATCCGTGGTGGAActtattaaTCGGCCGAGAATAAAGATAGAGTCCCATTTTAC-ATGTCAATACTGACAACAATGAAATTTATAGTAAGATGXXXGCTCTACT-CCCCAAAAAGGTCTGTTTGACACCTTACCTtttt-ttag-TTATTATCCT-TTTGAATTATTTAGAATCTATATCAGTTTTCATTTTCAAACTTAGAAAGTCTTCTTTTATTTATAAGATCCAAGAAATTCCCGGTCCAAAACTTTTTTAATTTACTACtttt------gagttTCTTTTCATTGACAGAGACCCAAGTCATATATTAAAATGATACTGATacttc--------------------------------------cgtaaTGGTCGGCATAGCTTAATTGCGGAGGACTGAAAATCCTTGTGTCACCATT--------------------------------------------------------------------------------------------AGTAAAA-------TGATACTTCAGTAGATGATACCTCAGTAATGGTGGACATAGC-------TTTTTTG--CAGAGGACTGAAAATC------CTTGTGTCACCATTCGTAAAATGAGGATGATACTTCGGTAATGGCCGGGATAGCTCAgttg

AY257758 AATTGGATTGAGCCTTGGTATGGAAACCTACTAAGTGATAACTTTCAAATTCAGAGAAACCCTGGAATTAAcaatgggcAATCCTGAGCCAAATCCTGGTTTACGTGAACAAACCGGAGTTTAGAAAGCGAGAAAAAA-GGGATAGGTGCAGAGACTCAATGGAAGCTGTTCTAACAAATGGAGTTCACTACCTTGTGTTGATAAAGGAATCCTTCGATCAAAACTTCAAATCAAAAAG-GTTGAAGGAGAAAAACCTATATTGTCTAAATATA------GGTAACACAAAA-CGATCTCAAAAATGACGACCTGAATCTCGATTTCGATTTTTTT--ATAAACAAAATCGAAATGATGTGAATCAATTCGAA-----GTTTAAGAAAtaatattcattGATCAAATGATTCTCTTCATAGTCTGATAGATCCGTGGTGGAActtattaaTCGGCCGAGAATAAAGATAGAGTCCCATTTTAC-ATGTCAATACTGACAACAATGAAATTTATAGTAAGATGXXXGCTCTACT-CCCCAAAAAGGTCTGTTTGACACCTTACCTtttt-ttag-TTATTATCCA-TTTGAATTATTTAGAATCTATATCAGTTTTCATTTTCAAACTTAGAAAGTCTTCTTTTATTTATAAGATCCAAGAAATTCCCGGTCCAAAACTTTTTTAATTTACTACtttt------gagttTCTTTTCATTGACAGAGACCCAAGTCATATAGTAAAATGATACTGATacttc--------------------------------------cgtaaTGGTCGGCATAGCTTAATTGCGGAGGACTGAAAATCCTTGTGTCACCATT--------------------------------------------------------------------------------------------AGTAAAA-------TGATACTTCAGTAGATGATACCTCAGTAATGGTGGACATAGC-------TTTTTTG--CAGAGGACTGAAAATC------CTTGTGTCACCATTCGTAAAATGAGGATGATACTTCGGTAATGGCCGGGATAGCTCAgttg

AY257760 AATTGGATTGAGCCTTGGTATGGAAACCTACTAAGTGATAACTTTCAAATTCAGAGAAACCCTGGAATTAAcaatgggcAATCCTGAGCCAAATCCTGGTTTACGTGAACAAACCGGAGTTTAGAAAGCGAGAAAAAA-GGGATAGGTGCAGAGACTCAATGGAAGCTGTTCTAACAAATGGAGTTCACTACCTTGTGTTGATAAAGGAATCCTTCGATCAAAACTTCAAATCAAAAAG-GTTGAAGGAGAAAAACCTATATTGTCTAAATATA------GGTAACACAAAA-CGATCTCAAAAATGACGACCTGAATCTCGATTTCTATTTTTTT--ATAAACAAAATCGAAATGATGTGAATCAATTCGAA-----GTTTAAGAAAtaatattcattGATCAAATGATTCTCTTCATAGTCTGATAGATCCGTGGTGGAActtattaaTCGGCCGAGAATAAAGATAGAGTCCCATTTTAC-ATGTCAATACTGACAACAATGAAATTTATAGTAAGATGXXXGCTCTACT-CCCCAAAAAGGTCTGTTTGACACCTTACCTtttt-ttag-TTATTATCCA-TTTGAATTATTTAGAATCTATATCAGTTTTCATTTTCAAACTTAGAAAGTCTTCTTTTATTTATAAGATCCAAGAAATTCCCGGTCCAAAACTTTTTTAATTTACTACtttt------gagttTCTTTTCATTGACAGAGACCCAAGTCATATATTAAAATGATACTGATacttc--------------------------------------cgtaaTGGTCGGCATAGCTTAATTGCGGAGGACTGAAAATCCTTGTGTCACCATT--------------------------------------------------------------------------------------------------------------------------------------------------------------------------------------------------------CGTAAAATGAGGATGATACTTCGGTAATGGCCGGGATAGCTCAgttg

trnLF_HL AATTGGATTGAGCCTTGGTATGGAAACCTACTAAGTGATAACTTTCAAATTCAGAGAAACCCTGGAATTAAcaatgggcAATCCTGAGCCAAATCCTGGTTTACGTGAACAAACCGGAGTTTAGAAAGCGAGAAAAAA-GGGATAGGTGCAGAGACTCAATGGAAGCTGTTCTAACAAATGGAGTTCACTACCTTGTGTTGATAAAGGAATCCTTCGATCAAAACTTCAAATCAAAAAG-GTTGAAGGAGAAAAACCTATATTGTCTAAATATA------GGTAACACAAAA-CGATCTCAAAAATGACGACCTGAATCTCGATTTCTATTTTTTT--ATAAACAAAATCGAAATGATGTGAATCAATTCGAA-----GTTTAAGAAAtaatattcattGATCAAATGATTCTCTTCATAGTCTGATAGATCCGTGGTGGAActtattaaTCGGCCGAGAATAAAGATAGAGTCCCATTTTAC-ATGTCAATACTGACAACAATGAAATTTATAGTAAGATGXXXGCTCTACT-CCCCAAAAAGGTCTGTTTGACACCTTACCTtttt-ttag-TTATTATCCA-TTTGAATTATTTAGAATCTATATCAGTTTTCATTTTCAAACTTAGAAAGTCTTCTTTTATTTATAAGATCCAAGAAATTCCCGGTCCAAAACTTTTTTAATTTACTACtttt------gagttTCTTTTCATTGACAGAGACCCAAGTCATATATTAAAATGATACTGATacttc--------------------------------------cgtaaTGGTCGGCATAGCTTAATTGCGGAGGACTGAAAATCCTTGTGTCACCATT--------------------------------------------------------------------------------------------AGTAAAATGATA-------CTTCAGTAGATGATACCTCAGTAATGGTGGACATAGC-------TTTTTTG--CAGAGGACTGAAAATC------CTTGTGTCACCATTCGTAAAATGAGGATGATACTTCGGTAATGGCCGGGATAGCTCAgttg

ay122454 nnnnnnnnnnnnnnnnnnnnnnnnnnnnnnnnnnnnnnnnnctttcaaattcagagaaaccctggaattaacaatgggcaatcctgagccaaatcctggtttacgcgaacaaaccggagtttacaaagcgagaaaaaa-gggataggtgcagagactcaatggaagctgttctaacaaatggagttcactaccttgtgttgataaaggaatccttcgatcgaaacttcaaatcaaaagg-gatgaaggataaaaacctatattgtataaattta------ggtaacacaaaa-tgatctcaaaaatgacgacctgaatctcgatttctttttttttt-ataaacaaaatcgaaatgttgtgaatcaattcgaa-----gtttaagaaataatatttattgatcaaatgattcacttcatagtctgatagatccttgatggaacttattaatcggacgagaataaagatagagtcccattttac-atgtcaatactgacaacaatgaaatttatagtaaga-gXXX-----nct--cncnaaaagg-ntg----acaccttaccttttt-ttcg-ttattatata-tttgagttatttagaatctatatcatttttcattttaaaacttagaacgtctttttttatttagaagatccaagaaattcccgatccaaaactttttgaatttactactttt------gaggttcttttcattgacatagacctaagtcatatattaaaatgatactgatacttc----------------------------------------------------------------------------------------------------------------------------------------------------------------------------------------------------------------agtagatgatacttcggtaatggtagacatagc-------ttttttg--caaaggactcgaaatc------ctnnnnnnnnnnnnnnnnnnnnnnnnnnnnnnnnnnnnnnnnnnnnnnnnnnnnnnnnnnn

ES503 AATTGGATTGAGCCTTGGTATGGAAACCTACTAAGTGATAACTTTCAAATTCAGAGAAACCCTGGAATTAACAATGGGCAATCCTGAGCCAAATCCTGGTTTACGTGAACAAACCGGAGTTTAGAAAGCGAGAAAAAA-GGGATAGGTGCAGAGACTCAATGGAAGCTGTTCTAACAAATGGAGTTCACTACCTTGTGTTGATAAAGGAATCCTTCGATCAAAACTTCAAATCAAAAAG-GATGAAGGAGAAAAACCTATATTGTCTAAATATA------GGTAACACAAAA-CGATCGCAAAAATGACGACCTGAATCTCGATTTCTATTTTTTT--ATAAACAAAATCGAAATGATGTGAATCAATTCGAA-----GTTTAAGAAATAATATTCATTGATCAAATGATTCTCTTCATAGTCTGATAGATCCGTGGTGGAACTTATTAATCGGCCGAGAATAAAGATAGAGTCCCATTTTAC-ATGTCAATACTGACAACAATGAAATTTATAGTAAGATGXXXnnnnnnnn-nnnnnnnnnnnnnnnnnnnnnnnnnnnnnnnnnn-nnnn-TTATTATCCA-TTTGAATTATTTAGAATCTATATCAGTTTTCATTTTCAAACTTAGAAAGTCTTCTTTTATTTATAAGATCCAAGAAATTCCCGGTCCAAAACTTTTTTAATTTACTACTTTT------GAGTTTCTGTTCATTGACAGAGACCCAAGTCATATATTAAAATGATACTGATACTTC--------------------------------------CGTAATGGTCGGCATAGCTTAATTGCGGAGGACTGAAAATCCTTGTGTCACCATT--------------------------------------------------------------------------------------------AGTAAAATGATAC-TGATACTTCAGTAGATGATACCTCAGTAATGGTGGACATAGC-------TTTTTTG--CAGAGGACTGAAAATC------CTTGTGTCACCATTCGTAAAATGAGGAnnnnnnnnnnnnnnnnnnnnnnnnnnnnnnnnnn

ES514 AATTGGATTGAGCCTTGGTATGGAAACCTACTAAGTGATAACTTTCAAATTCAGAGAAACCCTGGAATTAACAATGGGCAATCCTGAGCCAAATCCTGGTTTATGTGAACAAACCGGAGTTTAGAAAGCGAGAAAAAA-GGGATAGGTGCAGAGACTCAATGGAAGCTGTTCTAACAAATGGAGTTCACTACCTTGTGTTGATAAAGGAATCCTTCGATCAAAACTTCAAATAAAAAAG-GATGAAGGAGAAAAACCTATATTGTATAAATATA------GGTAACACAAAA-CGATCTCAAAAATGACGACCTGAATCTCGATTTCTATTTTTTT--ATAAACAAAATCGAAATGATGTGAATCAATTCGAA-----GTTTAAGAAATAATATTCATTGATCAAATGATTCTCTTCATAGTCTGATAGATCCGTGGTGGAACTTATTAATCGGACGAGAATAAAGATAGAGTCCCATTTTAC-ATGTCAATACTGACAACAATGAAATTTATAGTAAGATGXXXGCTCTACT-CCCCAAAAAGGTCTGTTTGACACCTTACCTTTTT-GTAG-TTATTATCCA-TTTGAATTATTTAGAATCTATATCAGTTTTCATTTTCAAACTTAGAAAGTCTTCTTTTATTTATAAGATCCAAAAAATTCCCGGTCCAAAACTTTTTTAATTTACTACTTTT------GAGTTTCTTTTCATTGACAGAGACCCAAGTCATATATTAAAATGATACTGATACTTC--------------------------------------CGTAATGGTCGGCATAGCTTAATTGCGGAGGACTGAAAATCCTTGTGTCACCATT--------------------------------------------------------------------------------------------AGTAAAATGATAA-TGATACTTCAGTAGATGATACCTCAGTAATGGTGGACATAGC-------TTTTTTG--CAGAGGACTGAAAATC------CTTGTGTCACCATTCGTAAAATGAGGATGATACTTCGGTAATGGCCGGGATAGCTCAGTTG

ES524 AATTGGATTGAGCCT-GGTATGGAAACCTACTAAGTGATAACTTTCAAATTCAGAGAAACCCTGGAATTAACAATGGGCAATCCTGAGCCAAATCCTGGTTTACGTGAACAAACCGGAGTTTAGAAAGCGAGAAAAAA-GGGATAGGTGCAGAGACTCAATGGAAGCTGTTCTAACAAATGGAGTTCACTACCTTGTGTTGATAAAGGAATCCTTCGATCAAAACTTCAAATCAAAAAG-GATGAAGGAGAAAAACCTATATTGTCTAAATATA------GGTAACACAAAA-CGATCGCAAAAATGACGACCTGAATCTCGATTTCTATTTTTTT--ATAAACAAAATCGAAATGATGTGAATCAATTCGAA-----GTTTAAGAAATAATATTCATTGATCAAATGATTCTCTTCATAGTCTGATAGATCCGTGGTGGAACTTATTAATCGGCCGAGAATAAAGATAGAGTCCCATTTTAC-ATGTCAATACTGACAACAATGAAATTTATAGTAAGAnnXXXGCTCTACT-CCCCAAAAAGGTCTGTTTGACACCTTACCTTTTT-TTAG-TTATTATCCA-TTTGAATTATTTAGAATCTATATCAGTTTTCATTTTCAAACTTAGAAAGTCTTCTTTTATTTATAAGATCCAAGAAATTCCCGGTCCAAAACTTTTTTAATTTACTACTTTT------GAGTTTCTGTTCATTGACAGAGACCCAAGTCATATATTAAAATGATACTGATACTTC--------------------------------------CGTAATGGTCGGCATAGCTTAATTGCGGAGGACTGAAAATCCTTGTGTCACCATT--------------------------------------------------------------------------------------------AGTAAAATGATAC-TGATACTTCAGTAGATGATACCTCAGTAATGGTGGACATAGC-------TTTTTTG--CAGAGGACTGAAAATC------CTTGTGTCACCATTCGTAAAATGAGGATGATACTTCGGTAATGGCCGGGATAGCTCAGTTG

ES526 AATTGGATTGAGCCTTGGTATGGAAACCTACTAAGTGATAACTTTCAAATTCAGAGAAACCCTGGAATTAACAATGGGCAATCCTGAGCCAAATCCTGGTTTACGTGAACAAACCGGAGTTTAGAAAGCGAGAAAAAA-GGGATAGGTGCAGAGACTCAATGGAAGCTGTTCTAACAAATGGAGTTCACTACCTTGTGTTGATAAAGGAATCCTTCGATCAAAACTTCAAATCAAAAAG-GATGAAGGAGAAAAACCTATATTGTCTAAATATA------GGTAACACAAAA-CGATCGCAAAAATGACGACCTGAATCTCGATTTCTATTTTTTT--ATAAACAAAATCGAAATGATGTGAATCAATTCGAA-----GTTTAAGAAATAATATTCATTGATCAAATGATTCTCTTCATAGTCTGATAGACCCGTGGTGGAACTTATTAATCGGCCGAGAATAAAGATAGAGTCCCATTTTAC-ATGTCAATACTGACANCNATGAAATTTATAGTAAGATGXXXnnnnnnnn-nnnnnnnnnnnnnnnnnnnnnnCCTTACCTTTTT-TTAG-TTATTATCCA-TTTGAATTATTTAGAATCTATATCAGTTTTCATTTTCAAACTTAGAAAGTCTTCTTTTATTTATAAGATCCAAGAAATTCCCGGTCCAAAACTTTTTTAATTTACTACTTTT------GAGTTTCTGTTCATTGACAGAGACCCAAGTCATATATTAAAATGATACTGATACTTC--------------------------------------CGTAATGGTCGGCATAGCTTAATTGCGGAGGACTGAAAATCCTTGTGTCACCATT--------------------------------------------------------------------------------------------AGTAAAATGATAC-TGATACTTCAGTAGATGATACCTCAGTAATGGTGGACATAGC-------TTTTTTG--CAGAGGACTGAAAATC------CTTGTGTCACCATTCGTAAAATGAGGATGATACTTCGGTAATGGCCGGGATAGCTCAGTTG

ES645 AATTGGATTGAGCCTTGGTATGGAAACCTACTAAGTGATAACTTTCAAATTCAGAGAAACCCTGGAATTAACAATGGGCAATCCTGAGCCAAATCCTGGTTTACGGGAACAAACCGGAGTTTAGAAAGCGAGAAAAAA-GGGATAGGTGCAGAGACTCAATGGAAGCTGTTCTAACAAATGGAGTTCACTACCTTGTGTTGATAAAGGAATCCTTCGATCAAAACTTCAAATAAAAAAG-GATGAAGGAGAAAAACCTATATTGTCTAAATATA------GGTAACACAAAA-CGATCGCAAAAATGACGACCTGAATCTCGATTTCTATTTTTTT--ATAAACAAAATCGAAATGATGTGAATCAATTCGAA-----GTTTAAGAAATAATATTCATTGATCAAATGATTCTCTTCATAGTCTGATAGATCCGTGGTGGAACTTATTAATCGGCCGAGAATAAAGATAGAGTCCCATTTTAC-ATGTCAATACTGACAACAATGAAATTTATAGTAAGATGXXXGCTCTACT-CCCCAAAAAGGTCTGTTTGACACCTTACCTTTTT-TTAG-TTATTATCCA-TTTGAATTATTTAGAATCTATATCAGTTTTCATTTTCAAACTTAGAAAGTCTTCTTTTATTTATAAGATCCAAGAAATTCCCGGTCCAAAACTTTTTTAATTTACTACTTTT------GAGTTTCTGTTCATTGACAGAGACCCAAGTCATATATTAAAATGATACTGATACTTC--------------------------------------CGTAATGGTCGGCATAGCTTAATTGCGGAGGACTGAAAATCCTTGTGTCACCATT--------------------------------------------------------------------------------------------AGTAAAATGATAC-TGATACTTCAGTAGATGATACCTCAGTAATGGTGGACATAGC-------TTTTTTG--CAGAGGACTGAAAATC------CTTGTGTCACCATTCGTAAAATGAGGATGATACTTCGGTAATGGCCGGGATAGCTCAGTTG

ES649 AATTGGATTGAGCCTTGGTATGGAAACCTACTAAGTGATAACTTTCAAATTCAGAGAAACCCTGGAATTAACAATGGGCAATCCTGAGCCAAATCCTGGTTTACGTGAACAAACCGGAGTTTAGAAAGCGAGAAAAAA-GGGATAGGTGCAGAGACTCAATGGAAGCTGTTCTAACAAATGGAGTTCACTACCTTGTGTTGATAAAGGAATCCTTCGATCAAAACTTCAAATAAAAAAG-GATGAAGGAGAAAAACCTATATTGTCTAAATATA------GGTAACACAAAA-CGATCGCAAAAATGACGACCTGAATCTCGATTTCTATTTTTTT--ATAAACAAAATCGAAATGATGTTAATCAATTCGAA-----GTTTAAGAAATAATATTCATTGATCAAATGATTCTCTTCATAGTCTGATAGATCCGTGGTGGAACTTATTAATCGGCCGAGAATAAAGATAGAGTCCCATTTTAC-ATGTCAATACTGACAACAATGAAATTTATAGTAAGATGXXXnnnnnnnn-nnnnnnnnnnnnnnnnnnnnnnCCTTACCTTTTT-TTAG-TTATTATCCA-TTTGAATTATTTAGAATCTATATCAGTTTTCATTTTCAAACTTAGAAAGTCTTCTTTTATTTATAAGATCCAAGAAATTCCCGGTCCAAAACTTTTTTAATTTACTACTTTT------GAGTTTCTGTTCATTGACAGAGACCCAAGTCATATATTAAAATGATACTGATACTTC--------------------------------------CGTAATGGTCGGCATAGCTTAATTGCGGAGGACTGAAAATCCTTGTGTCACCATT--------------------------------------------------------------------------------------------AGTAAAATGATAC-TGATACTTCAGTAGATGATACCTCAGTAATGGTGGACATAGC-------TTTTTTG--CAGAGGACTGAAAATC------CTTGTGTCACCATTCGTAAAATGAGGATGATACTTCGGTAATGGCCGGGATAGCTCAGTTG

ES736 AATTGGATTGAGCCTTGGTATGGAAACCTACTAAGTGATAACTTTCAAATTCAGAGAAACCCTGGAATTAACAATGGGCAATCCTGAGCCAAATCCTGGTTTATGTGAACAAACCGGAGTTTAGAAAGCGAGAAAAAA-GGGATAGGTGCAGAGACTCAATGGAAGCTGTTCTAACAAATGGAGTTCACAACCTTGTGTTGATAAAGGAATCCTTCGATCAAAACTTCAAATAAAAAAG-GATGAAGGAGAAAAACCTATATTGTCTAAATATA------GGTAACACAAAA-CGATCTCAAAAATGACGACCTGAATCTCGATTTCTATTTTTTT--ATAAACAAAATCGAAATGATGTGAATCAATTCGAA-----GTTTAAGAAATAATATTCATTGATCAAATGATTCTCTTCATAGTCTGATAGATCCGTGGTGGAACTTCTTAATCGGACGAGAATAAAGATAGAGTCCCATTTTAC-ATGTCAATACTGACAACAATGAAATTnnnnnnnnnnnnXXXnnnnTACT-CCCCAAAAAGGTCTGTTTGACACCTTACCTTTTT-GTAG-TTATTATCCA-TTTGAATTATTTAGAATCTATATCAGTTTTCATTTTCAAACTTAGAAAGTCTTCTTTTATTTATAAGATCCAAAAAATTCCCGGTCCAAAACTTTTTTAATTTACTACTTTT------GAGTTTCTTTTCATTGACAGAGACCCAAGTCATATATTAAAATGATACTGATACTTC--------------------------------------CGTAATGGTCGGCATAGCTTAATTGCGGAGGACTGAAAATCCTTGTGTCACCATT--------------------------------------------------------------------------------------------AGTAAAATGATAA-TGATACTTCAGTAGATGATACCTCAGTAATGGTGGACATAGC-------TTTTTTG--CAGAGGACTGAAAATC------CTTGTGTCACCATTCGTAAAATGAGGATGATACTTCGGTAATGGCCGGGATAGCTCAGTTG

ES903 AATTGGATTGAGCCTTGGTATGGAAACCTACTAAGTGATAACTTTCAAATTCAGAGAAACCCTGGAATTAACAATGGGCAATCCTGAGCCAAATCCTGGTTTACGTGAACAAACCGGAGTTTAGAAAGCGAGAAAAAA-GGGATAGGTGCAGAGACTCAATGGAAGCTGTTCTAACAAATGGAGTTCACTACCTTGTGTTGATAAAGGAATCCTTCGATCAAAACTTCAAATCAAAAAG-GATGAAGGAGAAAAACCTATATTGTCTAAATATA------GGTAACACAAAA-CGATCGCAAAAATGACGACCTGAATCTCGATTTCTATTTTTTT--ATAAACAAAATCGAAATGATGTGAATCAATTCGAA-----GTTTAAGAAATAATATTCATTGATCAAATGATTCTCTTCATAGTCTGATAGATCCGTGGTGGAACTTATTAATCGGCCGAGAATAAAGATAGAGTCCCATTTTAC-ATGTCAATACTGACAACAATGAAATTTATAGTAAGATGXXXGCTCTACT-CCCCAAAAAGGTCTGTTTGACACCTTACCTTTTT-TTAG-TTATTATCCA-TTTGAATTATTTAGAATCTATATCAGTTTTCATTTTCAAACTTAGAAAGTCTTCTTTTATTTATAAGATCCAAGAAATTCCCGGTCCAAAACTTTTTTAATTTACTACTTTT------GAGTTTCTGTTCATTGACAGAGACCCAAGTCATATATTAAAATGATACTGATACTTC--------------------------------------CGTAATGGTCGGCATAGCTTAATTGCGGAGGACTGAAAATCCTTGTGTCACCATT--------------------------------------------------------------------------------------------AGTAAAATGATAC-TGATACTTCAGTAGATGATACCTCAGTAATGGTGGACATAGC-------TTTTTTG--CAGAGGACTGAAAATC------CTTGTGTCACCATTCGTAAAATGAGGATGATACTTCGGTAATGGCCGGGATAGCTCAGTTG

ES904 AATTGGATTGAGCCTTGGTATGGAAACCTACTAAGTGATAACTTTCAAATTCAGAGAAACCCTGGAATTAACAATGGGCAATCCTGAGCCAAATCCTGGTTTATGTGAACAAACCGGAGTTTAGAAAGCGAGAAAAAA-GGGATAGGTGCAGAGACTCAATGGAAGCTGTTCTAACAAATGGAGTTCACAACCTTGTGTTGATAAAGGAATCCTTCGATCAAAACTTCAAATAAAAAAG-GATGAAGGAGAAAAACCTATATTGTCTAAATATA------GGTAACACAAAA-CGATCTCAAAAATGACGACCTGAATCTCGATTTCTATTTTTTT--ATAAACAAAATCGAAATGATGTGAATCAATTCGAA-----GTTTAAGAAATAATATTCATTGATCAAATGATTCTCTTCATAGTCTGATAGATCCNTGGTGGAACTTATTAATCGGACGAGAATAAAGATAGAGTCCCATTTTAC-ATGTCAATACTGACAACAATGAAATTTATAGTAAGATGXXXGCTCTACT-CCCCAAAAAGGTCTGTTTGACACCTTACCTTTTT-GTAG-TTATTATCCA-TTTGAATTATTTAGAATCTATATCAGTTTTCATTTTCAAACTTAGAAAGTCTTCTTTTATTTATAAGATCCAAAAAATTCCCGGTCCAAAACTTTTTTAATTTACTACTTTT------GAGTTTCTTTTCATTGACAGAGACCCAAGTCATATATTAAAATGATACTGATACTTC--------------------------------------CGTAATGGTCGGCATAGCTTAATTGCGGAGGACTGAAAATCCTTGTGTCACCATT--------------------------------------------------------------------------------------------AGTAAAATGATAA-TGATACTTCAGTAGATGATACCTCAGTAATGGTGGACATAGC-------TTTTTTG--CAGAGGACTGAAAATC------CTTGTGTCACCATTCGTAAAATGAGGATGATACTTCGGTAATGGCCGGGATAGCTCAGTTG

ES910 AATTGGATTGAGCCTTGGTATGGAAACCTATTAAGTGATAACTTTCAAATTCAGAGAAACCCTGGAATTAACAATGGGCAATCCTGAGCCAAATCCTGGTTTATGTGAACAAACCGGAGTTTAGAAAGCGAGAAAAAA-GGGATAGGTGCAGAGACTCAATGGAAGCTGTTCTAACAAATGGAGTTCACAACCTTGTGTTGATAAAGGAATCCTTCGATCAAAACTTCAAATAAAAAAG-GATGAAGGAGAAAAACCTATATTGTCTAAATATA------GGTAACACAAAA-CGATCTCAAAAATGACGACCTGAATCTCGATTTCTATTTTTTT--ATAAACAAAATCGAAATGATGTGAATCAATTCGAA-----GTTTAAGAAATAATATTCATTGATCAAATGATTCTCTTCATAGTCTGATAGATCCGTGGTGGAACTTATTAATCGGACGAGAATAAAGATAGAGTCCCATTTTAC-ATGTCAATACTGACAACAATGAAATTnnnnnnnnnnnnXXXGCTCTACT-CCCCAAAAAGGTCTGTTTGACACCTTACCTTTTT-GTAG-TTATTATCCA-TTTGAATTATTTAGAATCTATATCAGTTTTCATTTTCAAACTTAGAAAGTCTTCTTTTATTTATAAGATCCAAAAAATTCCCGGTCCAAAACTTTTTTAATTTACTACTTTT------GAGTTTCTTTTCATTGACAGAGACCCAAGTCATATATTAAAATGATACTGATACTTC--------------------------------------CGTAATGGTCGGCATAGCTTAATTGCGGAGGACTGAAAATCCTTGTGTCACCATT--------------------------------------------------------------------------------------------AGTAAAATGATAA-TGATACTTCAGTAGATGATACCTCAGTAATGGTGGACATAGC-------TTTTTTG--CAGAGGACTGAAAATC------CTTGTGTCACCATTCGTAAAATGAGGATGATACTTCGGTAATnnnnnnnnnnnnnnnnnnn

ES913 AATTGGATTGAGCCTTGGTATGGAAACCTACTAAGTGATAACTTTCAAATTCAGAGAAACCCTGGAATTAACAATGGGCAATCCTGAGCCAAATCCTGGTTTATGTGAACAAACCGGAGTTTAGAAAGCGAGAAAAAA-GGGATAGGTGCAGAGACTCAATGGAAGCTGTTCTAACAAATGGAGTTCACTACCTTGTGTTGATAAAGGAATCCTTCGATCAAAACTTCAAATAAAAAAG-GATGAAGGAGAAAAACCTATATTGTCTAAATATA------GGTAACACAAAA-CGATCTCAAAAATGACGACCTGAATCTCGATTTCTATTTTTTT--ATAAACAAAATCGAAATGATGTGAATCAATTCGAA-----GTTTAAGAAATAATATTCATTGATCAAATGATTCTCTTCATAGTCTGATAGATCCGTGGTGGAACTTATTAATCGGACGAGAATAAAGATAGAGTCCCATTTTAC-ATGTCAATACTGACAACAATGAAATTTATAGTAAGATGXXXGCTCTACT-CCCCAAAAAGGTCTGTTTGACACCTTACCTTTTT-GTAG-TTATTATCCA-TTTGAATTATTTAGAATCTACATCAGTTTTCATTTTCAAACTTAGAAAGTCTTCTTTTATTTATAAGATCCAAAAAATTCCCGGTCCAAAACTTTTTTAATTTACTACTTTT------GAGTTTCTTTTCATTGACAGAGACCCAAGTCATATATTAAAATGATACTGATACTTC--------------------------------------CGTAATGGTCGGCATAGCTTAATTGCGGAGGACTGAAAATCCTTGTGTCACCATT--------------------------------------------------------------------------------------------AGTAAAATGATAA-TGATACTTCAGTAGATGATACCTCAGTAATGGTGGACATAGC-------TTTTTTG--CAGAGGACTGAAAATC------CTTGTGTCACCATTCGTAAAATGAGGATGATACTTCGGTAATGGCCGGGATAGCTCAGTTG

TS105 AATTGGATTGAGCCTTGGTATGGAAACCTACTAAGTGATAACTTTCAAATTCAGAGAAACCCTGGAATTAACAATGGGCAATCCTGAGCCAAATCCTGGTTTATGTGAACAAACCGGAGTTTAGAAAGCGAGAAAAAA-GGGATAGGTGCAGAGACTCAATGGAAGCTGTTCTAACAAATGGAGTTCACTACCTTGTGTTGATAAAGGAATCCTTCGATCAAAACTTCAAATAAAAAAG-GATGAAGGAGAAAAACCTATATTGTCTAAATATA------GGTAACACAAAA-CGATCTCAAAAATGACGACCTGAATCTCGATTTCTATTTTTTT--ATAAACAAAATCGAAATGATGTGAATCAATTCGAA-----GTTTAAGAAATAATATTCATTGATCAAATGATTCTCTTCATAGTCTGATAGATCCGTGGTGGAACTTATTAATCGGACGAGAATAAAGATAGAGTCCCATTTTAC-ATGTCAATACTGACAACAATGAAATTTATAGTAAGATGXXXGCTCTACT-CCCCAAAAAGGTCTGTTTGACACCTTACCTTTTT-GTAG-TTATTATCCA-TTTGAATTATTTAGAATCTACATCAGTTTTCATTTTCAAACTTAGAAAGTCTTCTTTTATTTATAAGATCCAAAAAATTCCCGGTCCAAAACTTTTTTAATTTACTACTTTT------GAGTTTCTTTTCATTGACAGAGACCCAAGTCATATATTAAAATGATACTGATACTTC--------------------------------------CGTAATGGTCGGCATAGCTTAATTGCGGAGGACTGAAAATCCTTGTGTCACCATT--------------------------------------------------------------------------------------------AGTAAAATGATAA-TGATACTTCAGTAGATGATACCTCAGTAATGGTGGACATAGC-------TTTTTTG--CAGAGGACTGAAAATC------CTTGTGTCACCATTCGTAAAATGAGGATGATACTTCGGTAATGGCCGGGATAGCTCAGTTG

TS120 AATTGGATTGAGCCTTGGTATGGAAACCTACTAAGTGATAACTTTCAAATTCAGAGAAACCCTGGAATTAACAATGGGCAATCCTGAGCCAAATCCTGGTTTACGTGAACAAACCGGAGTTTAGAAAGCGAGAAAAAA-GGGATAGGTGCAGAGACTCAATGGAAGCTGTTCTAACAAATGGAGTTCACTACCTTGTGTTGATAAAGGAATCCTTCGATCAAAACTTCAAATCAAAAAG-GATGAAGGAGAAAAACCTATATTGTCTAAATATA------GGTAACACAAAA-CGATCGCAAAAATGACGACCTGAATCTCGATTTCTATTTTTTT--ATAAACAAAATCGAAATGATGTGAATCAATTCGAA-----GTTTAAGAAATAATATTCATTGATCAAATGATTCTCTTCATAGTCTGATAGATCCGTGGTGGAACTTATTAATCGGCCGAGAATAAAGATAGAGTCCCATTTTAC-ATGTCAATACTGACAACAATGAAATTTATAGTAAGATGXXXGCTCTACT-CCCCAAAAAGGTCTGTTTGACACCTTACCTTTTT-TTAG-TTATTATCCA-TTTGAATTATTTAGAATCTATATCAGTTTTCATTTTCAAACTTAGAAAGTCTTCTTTTATTTATAAGATCCAAGAAATTCCCGGTCCAAAACTTTTTTAATTTACTACTTTT------GAGTTTCTGTTCATTGACAGAGACCCAAGTCATATATTAAAATGATACTGATACTTC--------------------------------------CGTAATGGTCGGCATAGCTTAATTGCGGAGGACTGAAAATCCTTGTGTCACCATT--------------------------------------------------------------------------------------------AGTAAAATGATAC-TGATACTTCAGTAGATGATACCTCAGTAATGGTGGACATAGC-------TTTTTTG--CAGAGGACTGAAAATC------CTTGTGTCACCATTCGTAAAATGAGGATGATACTTCGGTAATGGCCGGGATAGCTCAGTTG

TS29 nnnnnnnnnnnnCCTTGGTATGGAAACCTACTAAGTGATAACTTTCAAATTCAGAGAAACCCTGGAATTAACAATGGGCAATCCTGAGCCAAATCCTGGTTTATGTGAACAAACCGGAGTTTAGAAAGCGAGAAAAAA-GGGATAGGTGCAGAGACTCAATGGAAGCTGTTCTAACAAATGGAGTTCACAACCTTGTGTTGATAAAGGAATCCTTCGATCAAAACTTCAAATAAAAAAG-GATGAAGGAGAAAAACCTATATTGTCTAAATATA------GGTAACACAAAA-CGATCTCAAAAATGACGACCTGAATCTCGATTTCTATTTTTTT--ATAAACAAAATCGAAATGATGTGAATCAATTCGAA-----GTTTAAGAAATAATATTCATTGATCAAATGATTCTCTTCATAGTCTGATAGATCCGTGGTGGAACTTATTAATCGGACGAGAATAAAGATAGAGTCCCATTTTAC-ATGTCAATACTGACAACAATGAAATTTATAGTAAGATGXXXGCTCTACT-CCCCAAAAAGGTCTGTTTGACACCTTACCTTTTT-GTAG-TTATTATCCA-TTTGAATTATTTAGAATCTATATCAGTTTTCATTTTCAAACTTAGAAAGTCTTCTTTTATTTATAAGATCCAAAAAATTCCCGGTCCAAAACTTTTTTAATTTACTACTTTT------GAGTTTCTTTTCATTGACAGAGACCCAAGTCATATATTAAAATGATACTGATACTTC--------------------------------------CGTAATGGTCGGCATAGCTTAATTGCGGAGGACTGAAAATCCTTGTGTCACCATT--------------------------------------------------------------------------------------------AGTAAAATGATAA-TGATACTTCAGTAGATGATACCTCAGTAATGGTGGACATAGC-------TTTTTTG--CAGAGGACTGAAAATC------CTTGTGTCACCATTCGTAAAATGAGGATGATACTTCGGTAATGGCCGGGATAGCTCAGTTG

TS67 AATTGGATTGAGCCTTGGTATGGAAACCTACTAAGTGATAACTTTCAAATTCAGAGAAACCCTGGAATTAACAATGGGCAATCCTGAGCCAAATCCTGGTTTATGTGAACAAACCGGAGTTTAGAAAGCGAGAAAAAA-GGGATAGGTGCAGAGACTCAATGGAAGCTGTTCTAACAAATGGAGTTCACAACCTTGTGTTGATAAAGGAATCCTTCGATCAAAACTTCAAATAAAAAAG-GATGAAGGAGAAAAACCTATATTGTCTAAATATA------GGTAACACAAAA-CGATCTCAAAAATGACGACCTGAATCTCGATTTCTATTTTTTT--ATAAACAAAATCGAAATGATGTGAATCAATTCGAA-----GTTTAAGAAATAATATTCATTGATCAAATGATTCTCTTCATAGTCTGATAGATCCGTGGTGGAACTTATTAATCGGACGAGAATAAAGATnnnnnnnnnnnnnnnnnnnnnnnnnnnnnnnnnnnnnnnnnnnnnnnnnnnnnnXXXGCTCTACT-CCCCAAAAAGGTCTGTTTGACACCTTACCTTTTT-GTAG-TTATTATCCA-TTTGAATTATTTAGAATCTATATCAGTTTTCATTTTCAAACTTAGAAAGTCTTCTTTTATTTATAAGATCCAAAAAATTCCCGGTCCAAAACTTTTTTAATTTACTACTTTT------GAGTTTCTTTTCATTGACAGAGACCCAAGTCATATATTAAAATGATACTGATACTTC--------------------------------------CGTAATGGTCGGCATAGCTTAATTGCGGAGGACTGAAAATCCTTGTGTCACCATT--------------------------------------------------------------------------------------------AGTAAAATGATAA-TGATACTTCAGTAGATGATACCTCAGTAATGGTGGACATAGC-------TTTTTTG--CAGAGGACTGAAAATC------CTTGTGTCACCATTCGTAAAATGAGGATGATACTTCGGTAATGGCCGGGATAGCTCAnnnn

TS300 AATTGGATTGAGCCTTGGTATGGAAACCTACTAAGTGATAACTTTCAAATTCAGAGAAACCCTGGAATTAACAATGGGCAATCCTGAGCCAAATCCTGGTTTACGTGAACAAACCGGAGTTTAGAAAGCGAGAAAAAA-GGGATAGGTGCAGAGACTCAATGGAAGCTGTTCTAACAAATGGAGTTCACTACCTTGTGTTGATAAAGGAATCCTTCGATCAAAACTTCAAATCAAAAAG-GATGAAGGAGAAAAACCTATATTGTCTAAATATA------GGTAACACAAAA-CGATCTCAAAAATGACGACCTGAATCTCGATTTCTATTTTTTT--ATAAACAAAATCGAAATGATGTGAATCAATTCGAA-----GTTTAAGAAATAATATTCATTGATCAAATGATTCTCTTCATAGTCTGATAGATCCGTGGTGGAACTTATTAATCGGACGAGAATAAAGATAGAGTCCCATTTTAC-ATGTCAATACTGACAACAATGAAATTTATAGTAAGATGXXXnnnnnnnnNnnnnnnnnnnnnnnnnnnnnnnCCTTACCTTTTT-TTAG-TTATTATCGA-TTTGAATTATTTAGAATCTATATCAGTTTTCATTTTCAAACTTAGAAAGTCTTCTTTTATTTATAAGATCCAAGAAATTCCCGGTCCAAAACTTTTTTAATTTACTACTTTT------GAGTTTCTTTTCATTGACAGAGACCCAAGTCATATATTAAAATGATACTGATACTTC--------------------------------------CGTAATGGTCGGCATAGCTTAATTGCGGAGGACTGAAAATCCTTGTGTCACCATT--------------------------------------------------------------------------------------------AGTAAAATGAGAA-TGATACTTCAGTAGATGATACCTCAGTAATGGTGGACATAGC-------TTTTTTnnnnnnnnnnnnnnnnnnnnnnnnnnnnnnnnnnnnnnnnnnnnnnnnnnnnnnnnnnnnnnnnnnnnnnnnnnnnnnnnnnnnnn

TS28 nnnnnnnnnnnnCCTTGGTATGGAAACCTACTAAGTGATAACTTTCAAATTCAGAGAAACCCTGGAATTAACAATGGGCAATCCTGAGCCAAATCCTGGTTTACGTGAACAAACCGGAGTTTAGAAAGCGAGAAAAAA-GGGATAGGTGCAGAGACTCAATGGAAGCTGTTCTAACAAATGGAGTTCACTACCTTGTGTTGATAAAGGAATCCTTCGATCAAAACTTCAAATCAAAAAG-GATGAAGGAGAAAAACCTATATTGTCTAAATATA------GGTAACACAAAA-CGATCTCAAAAATGACGACCTGAATCTCGATTTCTATTTTTTT--ATAAACAAAATCGAAATGATGTGAATCAATTCGAA-----GTTTAAGAAATAATATTCATTGATCAAATGATTCTCTTCATAGTCTGATAGATCCGTGGTGGAACTTATTAATCGGACGAGAATAAAGATAGAGTCCCATTTTAC-ATGTCAATACTGACAACAATGAAATTTATAGTAAGATnXXXnnnnnnnnnnnnnnnnnnnnnnnnnnnnnnnnnnnnnnnnnnT-TTAG-TTATTATCGA-TTTGAATTATTTAGAATCTATATCAGTTTTCATTTTCAAACTTAGAAAGTCTTCTTTTATTTATAAGATCCAAGAAATTCCCGGTCCAAAACTTTTTTAATTTACTACTTTT------GAGTTTCTTTTCATTGACAGAGACCCAAGTCATATATTAAAATGATACTGATACTTC--------------------------------------CGTAATGGTCGGCATAGCTTAATTGCGGAGGACTGAAAATCCTTGTGTCACCATT--------------------------------------------------------------------------------------------AGTAAAATGAGAA-TGATACTTCAGTAGATGATACCTCAGTAATGGTGGACATAGC-------TTTTTTnnnnnnnnnnnnnnnnnnnnnnnnnnnnnnnnnnnnnnnnnnnnnnnnnnnnnnnnnnnnnnnnnnnnnnnnnnnnnnnnnnnnnn

TS78 AATTGGATTGAGCCTTGGTATGGAAACCTACTAAGTGATAACTTTCAAATTCAGAGAAACCCTGGAATTAACAATGGGCAATCCTGAGCCAAATCCTGGTTTACGTGAACAAACCGGAGTTTAGAAAGCGAGAAAAAA-GGGATAGGTGCAGAGACTCAATGGAAGCTGTTCTAACAAATGGAGTTCACTACCTTGTGTTGATAAAGGAATCCTTCGATCAAAACTTCAAATCAAAAAG-GATGAAGGAGAAAAACCTATATTGTCTAAATATA------GGTAACACAAAA-CGATCTCAAAAATGACGACCTGAATCTCGATTTCTATTTTTTT--ATAAACAAAATCGAAATGATGTGAATCAATTCGAA-----GTTTAAGAAATAATATTCATTGATCAAATGATTCTCTTCATAGTCTGATAGATCCGTGGTGGAACTTATTAATCGGACGAGAATAAAGATAGAGTCCCATTTTAC-ATGTCAATACTGACAACAATGAAATTTATAGTAAGATGXXXnnnnnnnnNnnnnnnnnnnnnnnnnnnnnnnCCTTACCTTTTT-TTAG-TTATTATCGA-TTTGAATTATTTAGAATCTATATCAGTTTTCATTTTCAAACTTAGAAAGTCTTCTTTTATTTATAAGATCCAAGAAATTCCCGGTCCAAAACTTTTTTAATTTACTACTTTT------GAGTTTCTTTTCATTGACAGAGACCCAAGTCATATATTAAAATGATACTGATACTTC--------------------------------------CGTAATGGTCGGCATAGCTTAATTGCGGAGGACTGAAAATCCTTGTGTCACCATT--------------------------------------------------------------------------------------------AGTAAAATGAGAA-TGATACTTCAGTAGATGATACCTCAGTAATGGTGGACATAGC-------TTTTTTnnnnnnnnnnnnnnnnnnnnnnnnnnnnnnnnnnnnnnnnnnnnnnnnnnnnnnnnnnnnnnnnnnnnnnnnnnnnnnnnnnnnnn

TS290 nnnnnnnnTGAGCCTTGGTATGGAAACCTACTAAGTGATAACTTTCAAATTCAGAGAAACCCTGGAATTAACAATGGGCAATCCTGAGCCAAATCCTGGTTTATGTGAACAAACCGGAGTTTAGAAAGCGAGAAAAAA-GGGATAGGTGCAGAGACTCAATGGAAGCTGTTCTAACAAATGGAGTTCACAACCTTGTGTTGATAAAGGAATCCTTCGATCAAAACTTCAAATAAAAAAG-GATGAAGGAGAAAAACCTATATTGTCTAAATATA------GGTAACACAAAA-CGATCTCAAAAATGACGACCTGAATCTCGATTTCTATTTTTTT--ATAAACAAAATCGAAATGATGTGAATCAATTCGAA-----GTTTAAGAAATAATATTCATTGATCAAATGATTCTCTTCATAGTCTGATAGATCCGTGGTGGAACTTATTAATCGGACGAGAATAAAGATAGAGTCCCATTTTAC-ATGTCAATACTGACAACAATGAAATTTATAGTAAGATGXXXGCTCTACT-CCCCAAAAAGGTCTGTTTGACACCTTACCTTTTT-GTAG-TTATTATCCA-TTTGAATTATTTAGAATCTATATCAGTTTTCATTTTCAAACTTAGAAAGTCTTCTTTTATTTATAAGATCCAAAAAATTCCCGGTCCAAAACTTTTTTAATTTACTACTTTT------GAGTTTCTTTTCATTGACAGAGACCCAAGTCATATATTAAAATGATACTGATACTTC--------------------------------------CGTAATGGTCGGCATAGCTTAATTGCGGAGGACTGAAAATCCTTGTGTCACCATT--------------------------------------------------------------------------------------------AGTAAAATGATAA-TGATACTTCAGTAGATGATACCTCAGTAATGGTGGACATAGC-------TTTTTTG--CAGAGGACTGAAAATC------CTTGTGTCACCATTCGTAAAATGAGGATGATACTTCGGTAATGGCCGGGATAGCTCAGTTG

TS195 AATTGGATTGAGCCTTGGTATGGAAACCTACTAAGTGATAACTTTCAAATTCAGAGAAACCCTGGAATTAACAATGGGCAATCCTGAGCCAAATCCTGGTTTACGTGAACAAACCGGAGTTTAGAAAGCGAGAAAAAA-GGGATAGGTGCAGAGACTCAATGGAAGCTGTTCTAACAAATGGAGTTCACTACCTTGTGTTGATAAAGGAATCCTTCGATCAAAACTTCAAATCAAAAAG-GATGAAGGAGAAAAACCTATATTGTCTAAATATA------GGTAACACAAAA-CGATCTCAAAAATGACGACCTGAATCTCGATTTCTATTTTTTT--ATAAACAAAATCGAAATGATGTGAATCAATTCGAA-----GTTTAAGAAATAATATTCATTGATCAAATGATTCTCTTCATAGTCTGATAGATCCGTGGTGGAACTTATTAATCGGACGAGAATAAAGATAGAGTCCCATTTTAC-ATGTCAATACTGACAACAATGAAATTTATAGTAAGATGXXXNNNNNNNNNNNNNnAAAAGGTTTGTTTGACACCTTACCTTTTT-TTAG-TTATTATCGA-TTTGAATTATTTAGAATCTATATCAGTTTTCATTTTCAAACTTAGAAAGTCTTCTTTTATTTATAAGATCCAAGAAATTCCCGGTCCAAAACTTTTTTAATTTACTACTTTT------GAGTTTCTTTTCATTGACAGAGACCCAAGTCATATATTAAAATGATACTGATACTTC--------------------------------------CGTAATGGTCGGCATAGCTTAATTGCGGAGGACTGAAAATCCTTGTGTCACCATT--------------------------------------------------------------------------------------------CGTAAAATAAGGA-TGATACTTC-----------------------------------------------------------------------------------------------------------GGTAATGGCnnnnnnnnnnnnnnnn

TS218 AATTGGATTGAGCCTTGGTATGGAAACCTACTAAGTGATAACTTTCAAATTCAGAGAAACCCTGGAATTAACAATGGGCAATCCTGAGCCAAATCCTGGTTTACGTGAACAAACCGGAGTTTAGAAAGCGAGAAAAAA-GGGATAGGTGCAGAGACTCAATGGAAGCTGTTCTAACAAATGGAGTTCACTACCTTGTGTTGATAAAGGAATCCTTCGATCAAAACTTCAAATCAAAAAG-GATGAAGGAGAAAAACCTATATTGTCTAAATATA------GGTAACACAAAA-CGATCTCAAAAATGACGACCTGAATCTCGATTTCTATTTTTTT--ATAAACAAAATCGAAATGATGTGAATCAATTCGAA-----GTTTAAGAAATAATATTCATTGATCAAATGATTCTCTTCATAGTCTGATAGATCCGTGGTGGAACTTATTAATCGGACGAGAATAAAGATAGAGTCCCATTTTAC-ATGTCAATACTGACAACAATGAAATTTATAGTAAGATGXXXGCTCTACT-CCCCAAAAAGGTCTGTTTGACACCTTACCTTTTT-TTAG-TTATTATCGA-TTTGAATTATTTAGAATCTATATCAGTTTTCATTTTCAAACTTAGAAAGTCTTCTTTTATTTATAAGATCCAAGAAATTCCCGGTCCAAAACTTTTTTAATTTACTACTTTT------GAGTTTCTTTTCATTGACAGAGACCCAAGTCATATATTAAAATGATACTGATACTTC--------------------------------------CGTAATGGTCGGCATAGCTTAATTGCGGAGGACTGAAAATCCTTGTGTCACCATT--------------------------------------------------------------------------------------------CGTAAAATAAGGA-TGATACTTC-----------------------------------------------------------------------------------------------------------GGTAATGGCCGGGATAGCTCAnnnn

;

End;
