# Supplementary material for: Seed size, endosperm and germination variation in sexual and apomictic Boechera
Source: Front Plant Sci. 2022 Nov 18;13:991531. doi: 10.3389/fpls.2022.991531 (PMC9716183; doi:10.3389/fpls.2022.991531)
Supplement: Supplementary file 3 [file DataSheet_3.docx]

**Supplementary_tables_3-21**

**Supplementary table 3.** Model selection: results of likelihood ratio tests for the general linear mixed-effects model of seed area. Accession (62 accessions) and individual (nested under accession) were used as random factors. Model 2 had the best fit to the data. Significance levels: ⋅ < 0.1, ∗ < 0.05, ∗∗ < 0.01, ∗∗∗ < 0.001.

| Model number | fixed effects structure | DF | AIC | log likelihood | deviance | comparison | χ^2^ | DF (χ^2^) | *P*(χ^2^) |
| --- | --- | --- | --- | --- | --- | --- | --- | --- | --- |
| 1 | ploidy level + rep. mode + cp DNA lineage + species | 21 | -19950 | 9996.1 | -19992 | – | – | – | – |
| **2** | **ploidy level + cp DNA lineage + species** | 20 | -19952 | 9995.9 | -19992 | 2 – 1 | 0.455 | 1 | 0.500 |
| 3 | ploidy level + cp DNA lineage | 7 | -19953 | 9983.5 | -19967 | 3 – 2 | 24.85 | 13 | 0.024 * |
| 4 | ploidy level + species | 18 | -19947 | 9991.7 | -19983 | 4 – 2 | 8.4746 | 2 | 0.014 * |
| 5 | cp DNA lineage + species | 19 | -19942 | 9989.9 | -19980 | 5 – 2 | 12.036 | 1 | 0.001 *** |

**Supplementary table 4.** Results of the best-fitting model (GLMM) of seed area. CI are 95% confidence intervals; ICC is the interclass correlation coefficient.

Model: Seed area [mm^2^] = ploidy level + cpDNA lineage + species + (1|Accession/Individual)

|  | **Seed area [mm^2^]** | | | |
| --- | --- | --- | --- | --- |
| Predictors | Estimates | CI | *P* | DF |
| (Intercept) | 0.47 | -0.17 – 1.11 | 0.147 | 44.97 |
| ploidy level: triploid | 0.29 | 0.11 – 0.48 | 0.002 | 44.97 |
| cpDNA lineage: 2 | 0.23 | -0.31 – 0.77 | 0.405 | 44.96 |
| cpDNA lineage: 3 | -0.04 | -0.54 – 0.45 | 0.866 | 44.96 |
| Species: *stricta×retrofracta* | -0.11 | -0.72 – 0.50 | 0.719 | 45.05 |
| Species: *williamsii* | -0.06 | -0.64 – 0.51 | 0.825 | 44.97 |
| Species: *divaricarpa* | 0.11 | -0.36 – 0.58 | 0.642 | 44.98 |
| Species: *lasiocarpa* | 0.10 | -0.51 – 0.71 | 0.758 | 44.97 |
| Species: *lignifera* | 0.12 | -0.64 – 0.87 | 0.766 | 44.96 |
| Species: *microphylla* | 0.14 | -0.43 – 0.72 | 0.624 | 44.96 |
| Species: *polyantha* | 0.22 | -0.22 – 0.66 | 0.321 | 44.98 |
| Species: *polyantha×retrofracta* | 0.37 | -0.32 – 1.07 | 0.290 | 44.97 |
| Species: *retrofracta* | 0.67 | 0.18 – 1.17 | 0.008 | 44.98 |
| Species: *selbyi* | 0.41 | -0.17 – 0.98 | 0.165 | 44.97 |
| Species: *shistacea* | 0.25 | -0.50 – 1.01 | 0.511 | 44.97 |
| Species: *sp.* | 0.45 | -0.06 – 0.95 | 0.081 | 44.98 |
| Species: *stricta* | 0.17 | -0.29 – 0.63 | 0.469 | 44.98 |
| Random Effects | | | | |
| σ^2^ | 0.05 | | | |
| τ_00_ _Individual:Accession_ | 0.01 | | | |
| τ_00_ _Accession_ | 0.04 | | | |
| ICC | 0.48 | | | |
| N _Individual_ | 186 | | | |
| N _Accession_ | 62 | | | |
| Observations | 121272 | | | |
| Marginal R^2^ / Conditional R^2^ | 0.241 / 0.609 | | | |

**Supplementary table 5.** Model selection: results of likelihood ratio tests for the generalized linear mixed-effects models of seed size coefficient of variation (CV). We used Gamma-distributed errors and a log link function; accession (62 accessions) was used as random factor. Model 3 had the best fit to the data. Significance levels: ⋅ < 0.1, ∗ < 0.05, ∗∗ < 0.01, ∗∗∗ < 0.001.

| Model number | fixed effects structure | DF | AIC | log likelihood | deviance | comparison | χ^2^ | DF (χ^2^) | *P*(χ^2^) |
| --- | --- | --- | --- | --- | --- | --- | --- | --- | --- |
| 1 | ploidy level + rep. mode | 5 | 1111.6 | -550.80 | 1101.6 | – | – | – | – |
| 2 | ploidy level | 4 | 1114.5 | -553.24 | 1106.5 | 2 – 1 | 4.865 | 1 | 0.027 * |
| **3** | **rep. mode** | 4 | 1110.3 | -551.16 | 1102.3 | 3 – 1 | 0.712 | 1 | 0.399 |
| 4 | intercept only | 3 | 1112.6 | -553.31 | 1106.6 | 4 – 3 | 4.302 | 1 | 0.038 * |

**Supplementary table 6.** Results of the best-fitting model (GLMM) of seed size variability (coefficient of variation, CV). CI are 95% confidence intervals; ICC is the interclass correlation coefficient.

Model: CV = rep. mode + (1|Accession)

|  | **Coefficient of variation (CV)** | | |
| --- | --- | --- | --- |
| Predictors | Estimates | CI | *P* |
| (Intercept) | 28.32 | 24.53 – 32.70 | <0.001 |
| Rep. mode: sex | 0.82 | 0.68 – 0.98 | 0.032 |
| Random Effects | | | |
| σ^2^ | 0.00 | | |
| τ_00 Accession_ | 0.04 | | |
| ICC | 1.00 | | |
| N _Accession_ | 62 | | |
| Observations | 186 | | |
| Marginal R^2^ / Conditional R^2^ | 0.197 / 0.999 | | |

**Supplementary table 7.** Results of the best-fitting models (GLMMs) of the effects of seed type (dominant *vs*. non dominant) on seed mass for all accessions, and separately for the two types of apomictic and the sexual accessions. CI are 95% confidence intervals; ICC is the interclass correlation coefficient.

Model: log(seed mass) = seed type +(1|Accession)

Seed type is a binary factor where the dominant seed type for each accession is coded as 0, and the non-dominant seed types are coded as 1.

|  | **All accessions**  **log(seed mass)** | | | | **Apomictic, unreduced pollen**  **log(seed mass)** | | | | **Apomictic, reduced pollen**  **log(seed mass)** | | | | **Sexual**  **log(seed mass)** | | | |
| --- | --- | --- | --- | --- | --- | --- | --- | --- | --- | --- | --- | --- | --- | --- | --- | --- |
| *Predictors* | *Estimates* | *CI* | *p* | *df* | *Estimates* | *CI* | *p* | *df* | *Estimates* | *CI* | *p* | *df* | *Estimates* | *CI* | *p* | *df* |
| (Intercept) | 4.93 | 4.78 – 5.08 | **<0.001** | 28.10 | 5.05 | 4.82 – 5.28 | **<0.001** | 13.08 | 4.90 | 4.20 – 5.60 | **0.001** | 2.04 | 4.80 | 4.56 – 5.04 | **<0.001** | 11.00 |
| seed type | -0.06 | -0.11 – -0.01 | **0.011** | 1989.45 | -0.02 | -0.08 – 0.03 | 0.417 | 920.14 | -0.19 | -0.31 – -0.08 | **0.001** | 167.53 | -0.14 | -0.31 – 0.04 | 0.117 | 894.18 |
| **Random Effects** | | | | | | | | | | | | | | | | |
| σ^2^ | 0.10 | | | | 0.11 | | | | 0.11 | | | | 0.09 | | | |
| τ_00_ | 0.15 _Accession_ | | | | 0.15 _Accession_ | | | | 0.08 _Accession_ | | | | 0.14 _Accession_ | | | |
| ICC | 0.61 | | | | 0.59 | | | | 0.42 | | | | 0.63 | | | |
| N | 29 _Accession_ | | | | 14 _Accession_ | | | | 3 _Accession_ | | | | 12 _Accession_ | | | |
| Observations | 2010 | | | | 932 | | | | 171 | | | | 907 | | | |
| Marginal R^2^ / Conditional R^2^ | 0.002 / 0.607 | | | | 0.000 / 0.589 | | | | 0.038 / 0.446 | | | | 0.001 / 0.626 | | | |

**Supplementary table 8** Results of the GLMM models of the effect of ploidy class for three groups: apomictic with unreduced pollen, apomictic with reduced pollen, and sexual. CI are 95% confidence intervals; ICC is the interclass correlation coefficient.

Model: log(seed mass) = ploidy class + (1|Accession)

Ploidy classes for which there were less than 3 seeds in any of the groups were excluded. The reference class for apomictic with unreduced pollen is 2x:6x (embryo:endosperm ploidy), for apomictic with reduced pollen – 2x:5x, and for sexual group – 2x:3x.

|  | **Apomictic, unreduced pollen**  **log(seed mass)** | | | | **Apomictic, reduced pollen**  **log(seed mass)** | | | | **Sexual**  **log(seed mass)** | | | |
| --- | --- | --- | --- | --- | --- | --- | --- | --- | --- | --- | --- | --- |
| *Predictors* | *Estimates* | *CI* | *p* | *df* | *Estimates* | *CI* | *p* | *df* | *Estimates* | *CI* | *p* | *df* |
| (Intercept) | 5.05 | 4.83 – 5.28 | **<0.001** | 13.07 | 4.90 | 4.22 – 5.58 | **0.001** | 2.03 | 4.80 | 4.56 – 5.04 | **<0.001** | 11.00 |
| ploidy class 2:3 | 0.04 | -0.07 – 0.15 | 0.476 | 907.02 | -0.22 | -0.41 – -0.04 | **0.017** | 160.57 |  |  |  |  |
| ploidy class 2:4 | -0.10 | -0.20 – -0.01 | **0.034** | 906.08 | -0.38 | -0.54 – -0.22 | **<0.001** | 160.58 | -0.39 | -0.65 – -0.14 | **0.003** | 890.17 |
| ploidy class 2:5 | 0.03 | -0.22 – 0.27 | 0.828 | 904.67 |  |  |  |  |  |  |  |  |
| ploidy class 2:8 | 0.04 | -0.09 – 0.16 | 0.594 | 905.06 |  |  |  |  |  |  |  |  |
| ploidy class 4:6 | 0.03 | -0.10 – 0.17 | 0.648 | 906.76 |  |  |  |  |  |  |  |  |
| ploidy class 4:8 | -0.19 | -0.46 – 0.08 | 0.164 | 904.66 |  |  |  |  |  |  |  |  |
| ploidy class 4:10 | 0.01 | -0.19 – 0.21 | 0.950 | 906.16 |  |  |  |  |  |  |  |  |
| ploidy class 4:12 | -0.04 | -0.33 – 0.25 | 0.794 | 904.38 |  |  |  |  |  |  |  |  |
| ploidy class 2:7 |  |  |  |  | 0.10 | -0.13 – 0.33 | 0.403 | 160.01 |  |  |  |  |
| ploidy class 2:6 |  |  |  |  |  |  |  |  | 0.06 | -0.27 – 0.39 | 0.728 | 890.23 |
| **Random Effects** | | | | | | | | | | | | |
| σ^2^ | 0.11 | | | | 0.10 | | | | 0.08 | | | |
| τ_00_ | 0.15 _Accession_ | | | | 0.07 _Accession_ | | | | 0.14 _Accession_ | | | |
| ICC | 0.59 | | | | 0.42 | | | | 0.63 | | | |
| N | 14 _Accession_ | | | | 3 _Accession_ | | | | 12 _Accession_ | | | |
| Observations | 926 | | | | 166 | | | | 904 | | | |
| Marginal R^2^/ Conditional R^2^ | 0.004 / 0.587 | | | | 0.093 / 0.478 | | | | 0.004 / 0.628 | | | |

**Supplementary table 9.** Model selection: results of likelihood ratio tests for the generalized linear mixed-effects models of germination success; accession (62 accessions) and plate nested under sub-block (529 plates, 4 sub-blocks) was used as random factor. Model 2 had the best fit to the data. Significance levels: ⋅ < 0.1, ∗ < 0.05, ∗∗ < 0.01, ∗∗∗ < 0.001.

| Model number | fixed effects structure | DF | AIC | log likelihood | deviance | comparison | χ^2^ | DF (χ^2^) | *P*(χ^2^) |
| --- | --- | --- | --- | --- | --- | --- | --- | --- | --- |
| 1 | seed mass + seed area + rep. mode + seed mass*rep. mode + seed area*rep. mode | 9 | 2802.2 | -1392.1 | 2784.2 | – | – | – | – |
| **2** | **seed mass + seed area + rep. mode + seed mass*rep. mode** | 8 | 2800.5 | -1392.3 | 2784.5 | 2 –1 | 0.3197 | 1 | 0.5718 |
| 3 | seed mass + seed area + rep. mode | 7 | 2805.3 | -1395.7 | 2791.3 | 3 –2 | 6.793 | 1 | 0.0092 ** |
| 4 | seed mass + rep. mode + seed mass*rep. mode | 7 | 2803.1 | -1394.6 | 2789.1 | 4 –2 | 4.6017 | 1 | 0.0319 * |

**Supplementary table 10.** Results of the best-fitting model (GLMM) of germination success using binomial distribution and logit link function. CI are 95% confidence intervals; ICC is the interclass correlation coefficient.

Model:

germination success = seed mass + seed area+ reproductive mode +reproductive mode*seed mass+(1|Accession)+(1|sub-block/plate)

|  | **germination success** | | |
| --- | --- | --- | --- |
| Predictors | Odds Ratios | CI | *P* |
| (Intercept) | 6.07 | 0.66 – 56.23 | 0.112 |
| seed mass | 3.34 | 2.59 – 4.32 | <0.001 |
| seed area | 1.28 | 1.02 – 1.61 | 0.033 |
| reproductive mode: sex | 0.66 | 0.03 – 15.19 | 0.792 |
| seed mass*reproductive mode(sex) | 0.64 | 0.46 – 0.90 | 0.009 |
| Random Effects | | | |
| σ^2^ | 3.29 | | |
| τ_00_ _plate:sub-block_ | 1.89 | | |
| τ_00_ _Accession_ | 9.54 | | |
| τ_00_ _sub-block_ | 0.83 | | |
| ICC | 0.79 | | |
| N _Accession_ | 23 | | |
| N _plate_ | 529 | | |
| N _sub-block_ | 4 | | |
| Observations | 4184 | | |
| Marginal R^2^ / Conditional R^2^ | 0.078 / 0.805 | | |

**Supplementary table 11.** Model selection: results of likelihood ratio tests for the generalized linear mixed-effects models of germination timing. We used binomial distribution and a logit link function; accession (23 accessions) and plate nested under sub-block (512 plates, 4 sub-blocks) were used as random factors. Model 2 had the best fit to the data at 90% confidence level. Significance levels: ⋅ < 0.1, ∗ < 0.05, ∗∗ < 0.01, ∗∗∗ < 0.001.

| Model number | fixed effects structure | DF | AIC | log likelihood | deviance | comparison | χ^2^ | DF (χ^2^) | *P*(χ^2^) |
| --- | --- | --- | --- | --- | --- | --- | --- | --- | --- |
| 1 | seed mass + rep. mode + rep. mode*seed mass | 7 | 1794.4 | -890.19 | 1780.4 | – | – | – | – |
| **2** | **seed mass + rep. mode** | 6 | 1793.7 | -890.83 | 1781.7 | 2 – 1 | 1.296 | 1 | 0.255 |
| 3 | seed mass | 5 | 1794.7 | -892.34 | 1784.7 | 3 – 2 | 3.020 | 1 | 0.082 ⋅ |
| 4 | rep. mode | 5 | 1797.9 | -893.96 | 1787.9 | 4 – 2 | 6.244 | 1 | 0.013 * |

**Supplementary table 12.** Results best-fitting model (GLMM) of germination timing. Germination timing is a binary response variable where “early” seeds are coded as 0 and “late” as 1. CI are 95% confidence intervals; ICC is the interclass correlation coefficient.

Model:

germination timing = seed mass + reproductive mode +(1|Accession)+(1|sub-block/plate)

|  | **germination timing** | | |
| --- | --- | --- | --- |
| Predictors | Odds Ratios | CI | *P* |
| (Intercept) | 0.03 | 0.01 – 0.08 | <0.001 |
| seed mass | 0.77 | 0.63 – 0.94 | 0.009 |
| reproductive mode: sex | 4.13 | 0.83 – 20.43 | 0.082 |
| Random Effects | | | |
| σ^2^ | 3.29 | | |
| τ_00_  _plate:sub-block_ | 1.13 | | |
| τ_00_ _Accession_ | 2.60 | | |
| τ_00_ _sub-block_ | 0.17 | | |
| ICC | 0.54 | | |
| N _Accession_ | 23 | | |
| N _plate_ | 512 | | |
| N _sub-block_ | 4 | | |
| Observations | 3800 | | |
| Marginal R^2^ / Conditional R^2^ | 0.077 / 0.578 | | |

**Supplementary table 13.** Model selection: results of likelihood ratio tests for the generalized linear mixed-effects models of seedling development. Seedling development was treated as a binary variable: normal seedlings were coded as 1, and abnormal as 0. We used binomial distribution and a logit link function; accession (23 accessions) and plate nested under sub-block (512 plates, 4 sub-blocks) were used as random factors. Model 1 had the best fit to the data. Significance levels: ⋅ < 0.1, ∗ < 0.05, ∗∗ < 0.01, ∗∗∗ < 0.001.

| Model number | fixed effects structure | DF | AIC | log likelihood | deviance | comparison | χ^2^ | DF (χ^2^) | *P*(χ^2^) |
| --- | --- | --- | --- | --- | --- | --- | --- | --- | --- |
| **1** | **seed mass + rep. mode + seed mass*rep. mode** | 7 | 1317.6 | -651.81 | 1303.6 | – | – | – | – |
| 2 | seed mass + rep. mode | 6 | 1328.0 | -658.01 | 1316.0 | 2 – 1 | 12.389 | 1 | <0.001 *** |

**Supplementary table 14.** Results of best-fitting model (GLMM) of seedling development. Seedling development is a binary response variable where normal seedlings are coded as 1 and abnormal as 0. CI are 95% confidence intervals; ICC is the interclass correlation coefficient.

Model:

seedling development = seed mass + reproductive mode + seed mass*reproductive mode + (1|Accession)+(1|subblock/plate)

|  | **seedling development** | | |
| --- | --- | --- | --- |
| Predictors | Odds Ratios | CI | *P* |
| (Intercept) | 88.46 | 26.62 – 293.90 | <0.001 |
| seed mass | 2.48 | 1.50 – 4.09 | <0.001 |
| reproductive mode: sex | 0.20 | 0.05 – 0.76 | 0.018 |
| seed mass*reproductive mode(sex) | 0.37 | 0.21 – 0.66 | 0.001 |
| Random Effects | | | |
| σ^2^ | 3.29 | | |
| τ_00_ _plate:sub-block_ | 1.30 | | |
| τ_00_ _Accession_ | 1.33 | | |
| τ_00_ _sub-block_ | 0.63 | | |
| ICC | 0.50 | | |
| N _Accession_ | 23 | | |
| N _plate_ | 511 | | |
| N _sub-block_ | 4 | | |
| Observations | 3782 | | |
| Marginal R^2^ / Conditional R^2^ | 0.142 / 0.570 | | |

**Supplementary table 15.** Model selection: results of likelihood ratio tests for the linear mixed-effects models of seedling total root growth (RGR-T). We used accession (23 accessions) as random factor. Model 2 had the best fit to the data. Significance levels: ⋅ < 0.1, ∗ < 0.05, ∗∗ < 0.01, ∗∗∗ < 0.001.

| Model number | fixed effects structure | DF | AIC | log likelihood | deviance | comparison | χ^2^ | DF (χ^2^) | *P*(χ^2^) |
| --- | --- | --- | --- | --- | --- | --- | --- | --- | --- |
| 1 | seed mass + rep. mode + seedling development +  rep. mode * seed mass + rep. mode * seedling development | 8 | 9141.7 | -4562.9 | 9125.7 | – | – | – | – |
| **2** | **seed mass + rep. mode + seedling development + rep. mode * seedling development** | 7 | 9141.9 | -4563.9 | 9127.9 | 2 – 1 | 2.122 | 1 | 0.145 |
| 3 | seed mass + rep. mode + seedling development | 6 | 9146.6 | -4567.3 | 9134.6 | 3 – 2 | 6.749 | 1 | 0.009 ** |
| 4 | rep. mode + seedling development + rep. mode * seedling development | 6 | 9245.1 | -4616.6 | 9233.1 | 4 – 2 | 105.27 | 1 | <0.001 *** |

**Supplementary table 16.** Results of best-fitting model (GLMM) of seedling total root growth rate (RGR-T). Seedling development is a binary trait where normal seedlings are coded as 1, and abnormal as 0. CI are 95% confidence intervals; ICC is the interclass correlation coefficient.

Model:

log(seedling root growth rate (RGR-T))= seed mass + reproductive mode + seedling development + reproductive mode * seedling development + (1|Accession)

|  | **log(seedling total root growth rate (RGR-T)) [mm^2^/day]** | | | |
| --- | --- | --- | --- | --- |
| Predictors | Estimates | CI | *P* | DF |
| (Intercept) | -1.88 | -2.40 – -1.35 | <0.001 | 232.41 |
| Rep mode sex:seedling dev 1 | 0.68 | 0.17 – 1.20 | <0.001 | 3335.32 |
| seed mass | 0.00 | 0.00 – 0.00 | 0.010 | 140.49 |
| seedling dev:  seedling dev 1 | 1.55 | 1.11 – 1.99 | <0.001 | 3486.40 |
| Rep mode: sex | -0.86 | -1.50 – -0.22 | 0.009 | 3487.83 |
| Random Effects | | | | |
| σ^2^ | 0.77 | | | |
| τ_00_ _Accession_ | 0.22 | | | |
| ICC | 0.22 | | | |
| N _Accession_ | 23 | | | |
| Observations | 3504 | | | |
| Marginal R^2^ / Conditional R^2^ | 0.124 / 0.320 | | | |

**Supplementary table 17.** Model selection: results of likelihood ratio tests for the linear mixed-effects models of individual variation (measured as coefficient of variation, CV) in seedling root growth (RGR-T). We used accession (23 accessions) as random factor. Model 2 had the best fit to the data. Significance levels: ⋅ < 0.1, ∗ < 0.05, ∗∗ < 0.01, ∗∗∗ < 0.001.

| Model number | fixed effects structure | DF | AIC | log likelihood | deviance | comparison | χ^2^ | DF (χ^2^) | *P*(χ^2^) |
| --- | --- | --- | --- | --- | --- | --- | --- | --- | --- |
| 1 | rep. mode | 4 | 54.299 | -23.149 | 46.299 | – | – | – | – |
| **2** | **intercept only** | 3 | 52.392 | -23.196 | 46.392 | 2 – 1 | 0.0927 | 1 | 0.7608 |

**Supplementary table 18.** Model selection: results of likelihood ratio tests for the linear mixed-effects models of initial root growth rate (RGR-I). We used accession (23 accessions), and sub-block (4 sub-blocks) as random factors. Model 2 had the best fit to the data. Significance levels: ⋅ < 0.1, ∗ < 0.05, ∗∗ < 0.01, ∗∗∗ < 0.001.

| Model number | fixed effects structure | DF | AIC | log likelihood | deviance | comparison | χ^2^ | DF (χ^2^) | *P*(χ^2^) |
| --- | --- | --- | --- | --- | --- | --- | --- | --- | --- |
| 1 | rep. mode + seed mass | 6 | -7908.0 | 3960.0 | -7920.0 | – | – | – | – |
| **2** | **seed mass** | 5 | -7909.4 | 3959.7 | -7919.4 | 2 – 1 | 0.654 | 1 | 0.419 |
| 3 | intercept only | 4 | -7859.8 | 3933.9 | -7867.8 | 3 – 2 | 51.509 | 1 | >0.001 *** |

**Supplementary table 19.** Results of best-fitting model (GLMM) of initial root growth rate (RGR-I). CI are 95% confidence intervals; ICC is the interclass correlation coefficient.

Model:

seedling root growth rate (RGR-I) = log(seed mass) + (1|Accession) + (1|sub-block)

|  | **seedling initial root growth rate (RGR-I) [mm/day]** | | | |
| --- | --- | --- | --- | --- |
| Predictors | Estimates | CI | *P* | DF |
| (Intercept) | 0.06 | 0.02 – 0.10 | 0.008 | 241.39 |
| log(seed mass) | 0.03 | 0.02 – 0.04 | <0.001 | 1889.56 |
| Random Effects | | | | |
| σ^2^ | 0.01 | | | |
| τ_00_ _Accession_ | 0.00 | | | |
| τ_00_ _sub-block_ | 0.00 | | | |
| ICC | 0.11 | | | |
| N _Accession_ | 23 | | | |
| N _sub-block_ | 4 | | | |
| Observations | 3441 | | | |
| Marginal R^2^ / Conditional R^2^ | 0.029 / 0.132 | | | |

**Supplementary table 20.** Model selection: results of likelihood ratio tests for the linear mixed-effects models of flowering time. We used accession (12 accessions), as random factor. Model 4 had the best fit to the data; however we were not able to test it against an intercept-only model, which failed to converge. Significance levels: ⋅ < 0.1, ∗ < 0.05, ∗∗ < 0.01, ∗∗∗ < 0.001.

| Model number | fixed effects structure | DF | AIC | log likelihood | deviance | comparison | χ^2^ | DF (χ^2^) | *P*(χ^2^) |
| --- | --- | --- | --- | --- | --- | --- | --- | --- | --- |
| 1 | seed mass + rep. mode +  rep. mode * seed mass | 6 | 7687.4 | -3837.7 | 7675.4 | – | – | – | – |
| 2 | seed mass + rep. mode | 5 | 7686.9 | -3838.5 | 7676.9 | 2 – 1 | 1.515 | 1 | 0.218 |
| 3 | seed mass | 4 | 7684.9 | -3838.5 | 7676.9 | 3 – 2 | 0.003 | 1 | 0.954 |
| **4** | **rep. mode** | 4 | 7687.0 | -3839.5 | 7679.0 | 4 – 2 | 2.026 | 1 | 0.155 |

**Supplementary table 21.** Results of best-fitting model (GLMM) of flowering time.

CI are 95% confidence intervals; ICC is the interclass correlation coefficient.

Model:

Flowering time = reproductive mode + (1|Accession)

|  | **Flowering time** | | | |
| --- | --- | --- | --- | --- |
| Predictors | Estimates | CI | *P* | DF |
| (Intercept) | 52160.92 | 32245.43 – 72076.41 | 0.001 | 8.98 |
| rep. mode: sex | 404.02 | -26571.75 – 27379.79 | 0.977 | 9.00 |
| Random Effects | | | | |
| σ^2^ | 81816525.35 | | | |
| τ_00_ _Accession_ | 513953753.58 | | | |
| ICC | 0.86 | | | |
| N _Accession_ | 11 | | | |
| Observations | 362 | | | |
| Marginal R^2^ / Conditional R^2^ | 0.000 / 0.863 | | | |
