# Supplementary material for: Seed size, endosperm and germination variation in sexual and apomictic Boechera
Source: Front Plant Sci. 2022 Nov 18;13:991531. doi: 10.3389/fpls.2022.991531 (PMC9716183; doi:10.3389/fpls.2022.991531)
Supplement: Supplementary file 4 [file DataSheet_4.docx]

**Supplementary_figures_1-11**


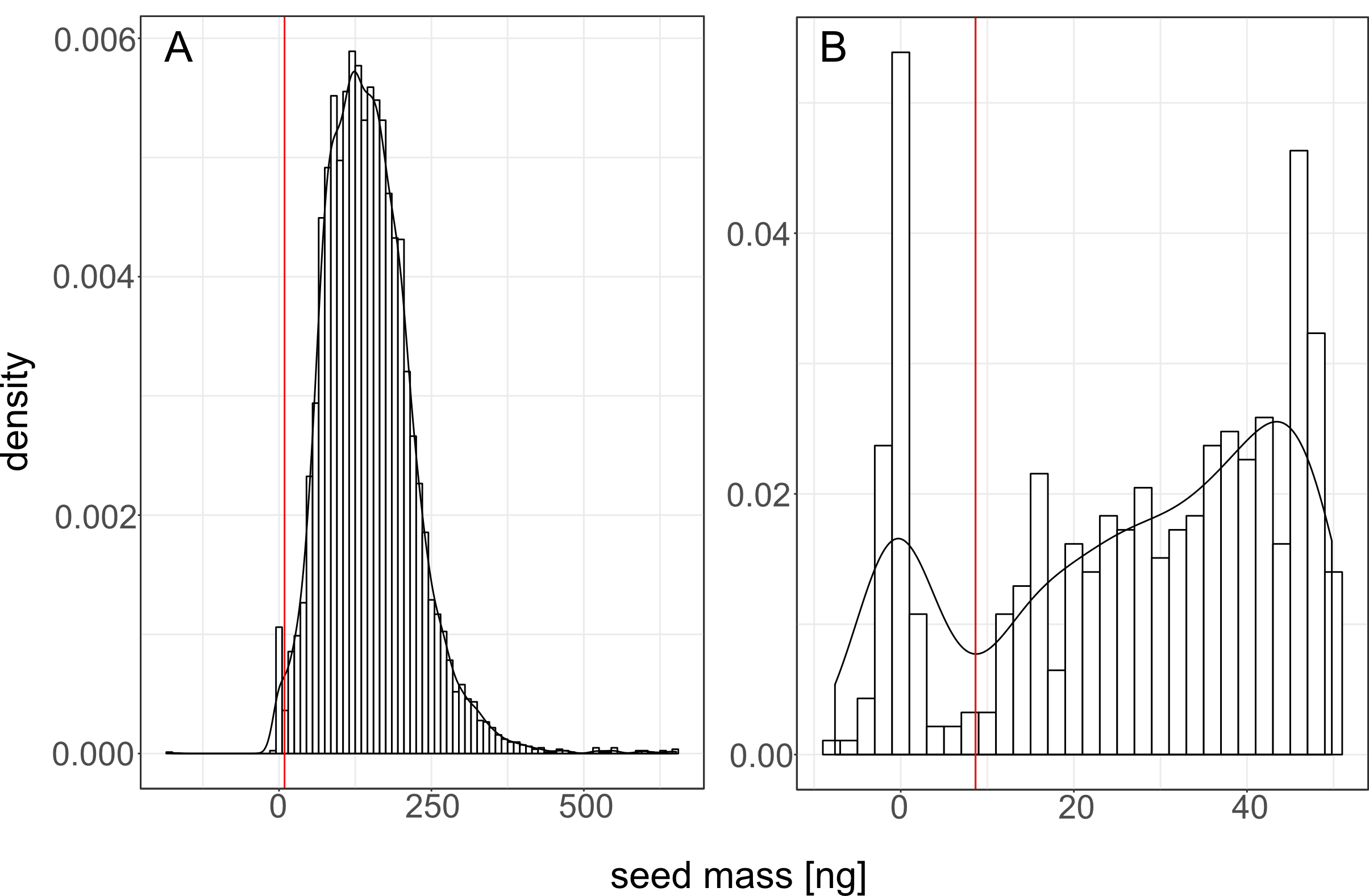


**Supplementary figure 1.** Histograms of seed mass with overlaid kernel density functions. **A.** Full range of seed mass measurements. **B.** Seed mass measurements between -10 and 50 ng. The local minimum of density function in this region was identified at 8.64 (red line); the measurements below this threshold are likely due to errors with balance tarring and have been removed from subsequent analyses of seed size.


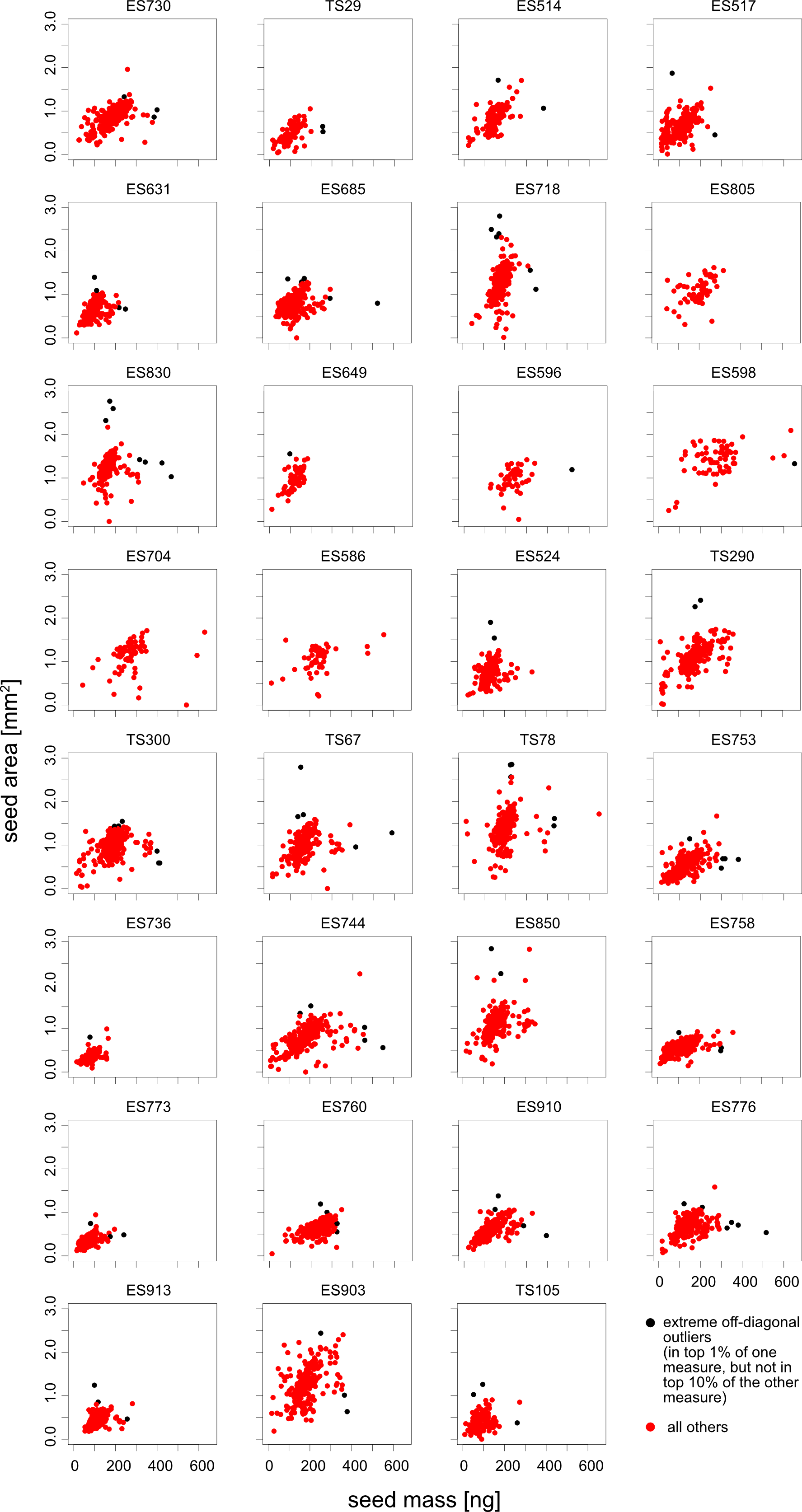


**Supplementary figure 2.** Relationship between seed mass and seed area for 31 *Boechera* accessions. Extreme off-diagonal outliers, defined as within top 1^st^ percentile of one measure and not within top 10^th^ percentile of the other measure, are marked in black.


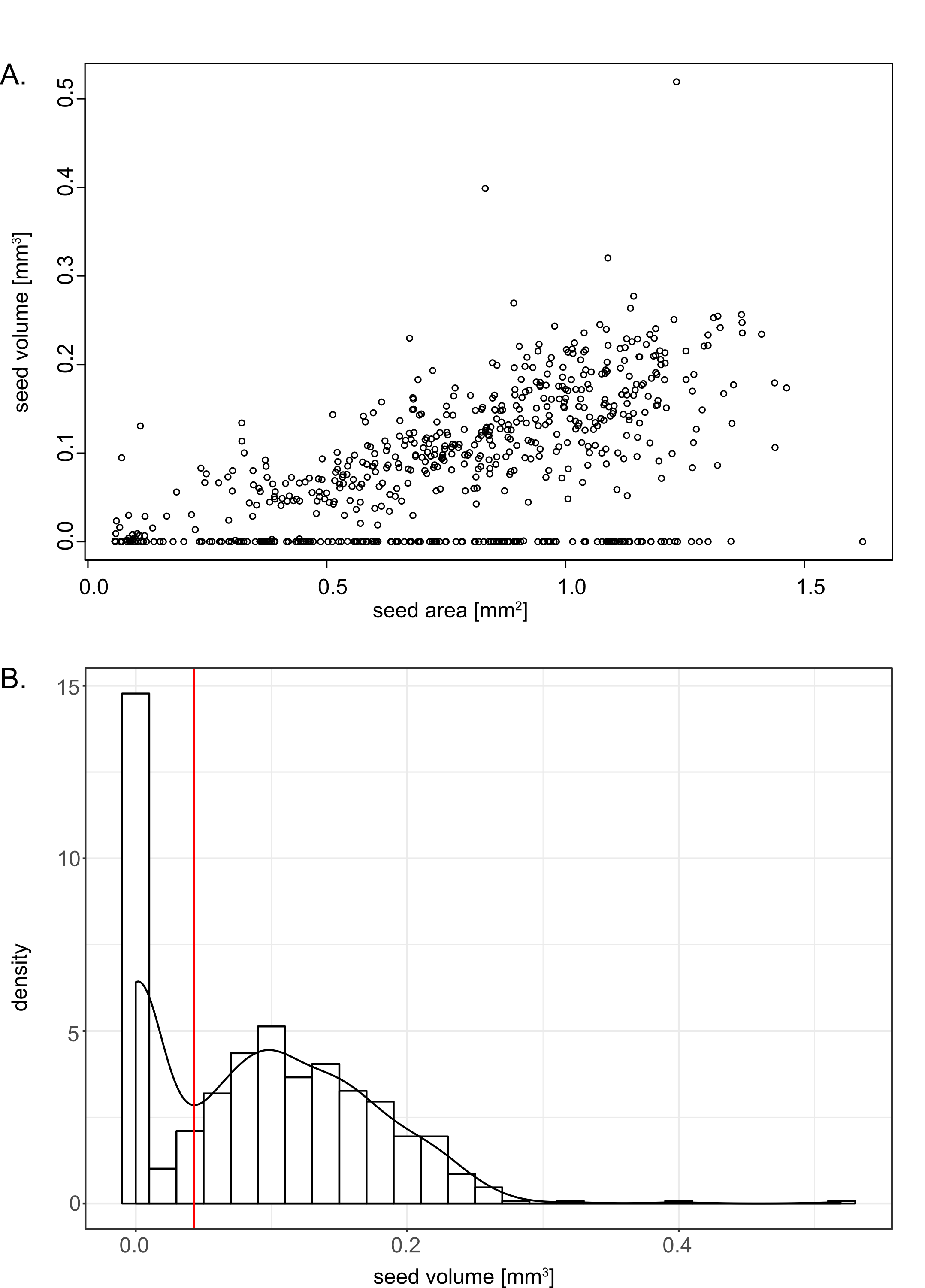


**Supplementary figure 3.** Seed volume measured with *phenoSeeder* in 7 *Boechera* accessions **A.** The relationship between seed volume and seed area*.* **B.** The histogram of seed volume with overlaid density plot. The cut-off value for failed measurements was defined as a local minimum of density function at 0.043 mm^3^ (red line).


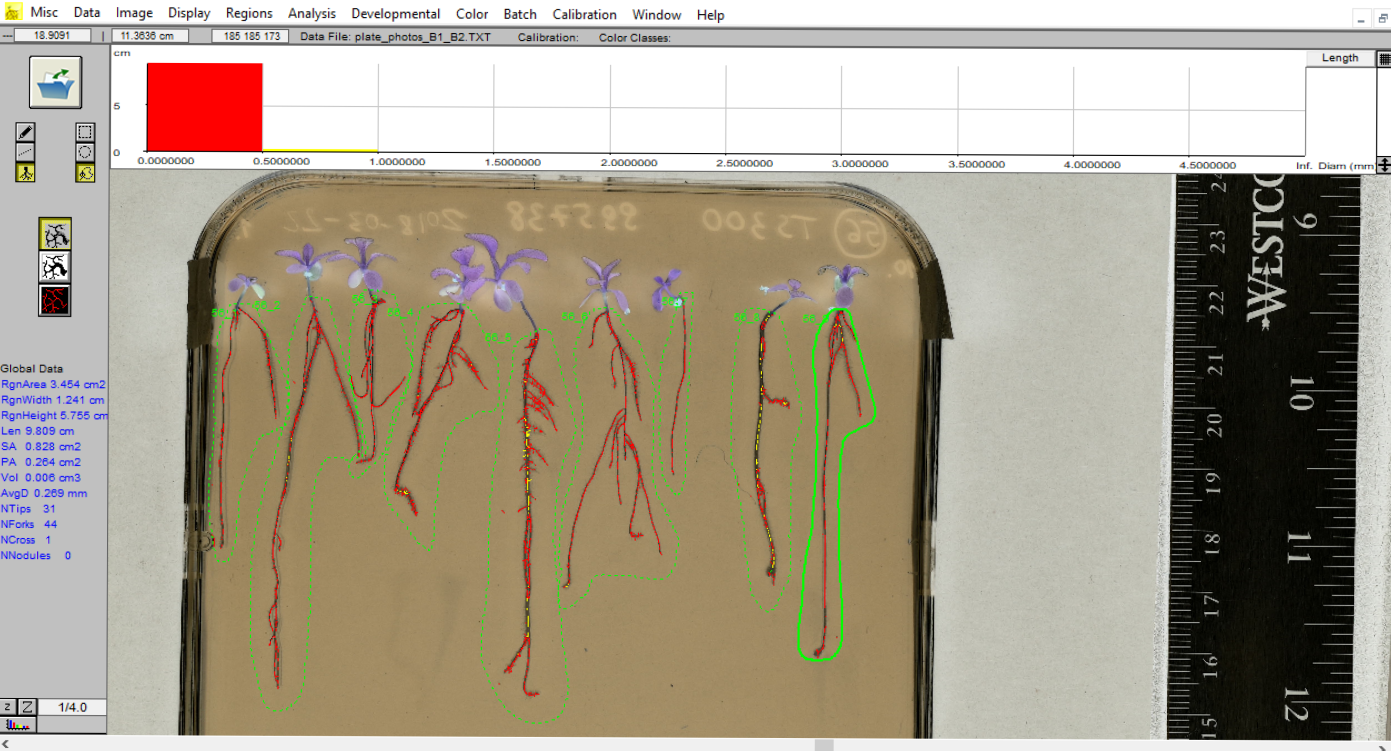


**Supplementary figure 4.** Example of root measurements in WinRHIZO showing adjacent roots being recognized as a single root. Root diameter classes are marked with colours (red, yellow).


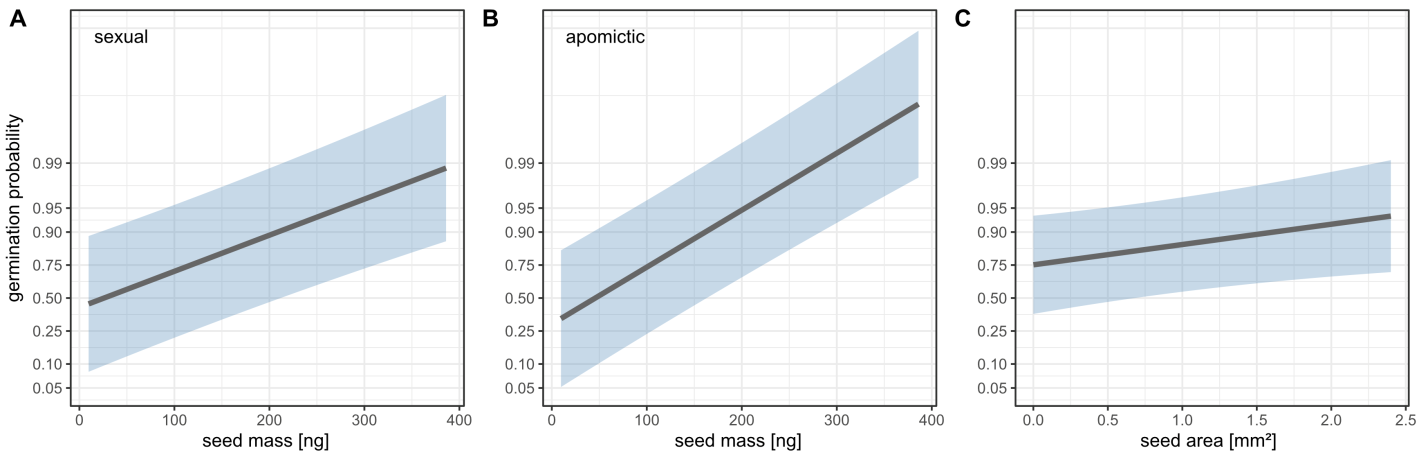


**Supplementary figure 5.** Effects of seed mass and seed area on germination success. **A.** Effect of seed mass in sexual accessions, **B.** Effect of seed mass in apomictic accessions, **C.** Effect of seed area in all accessions. Germination probability is plotted on a logit scale. Shaded area represents 95% confidence intervals.


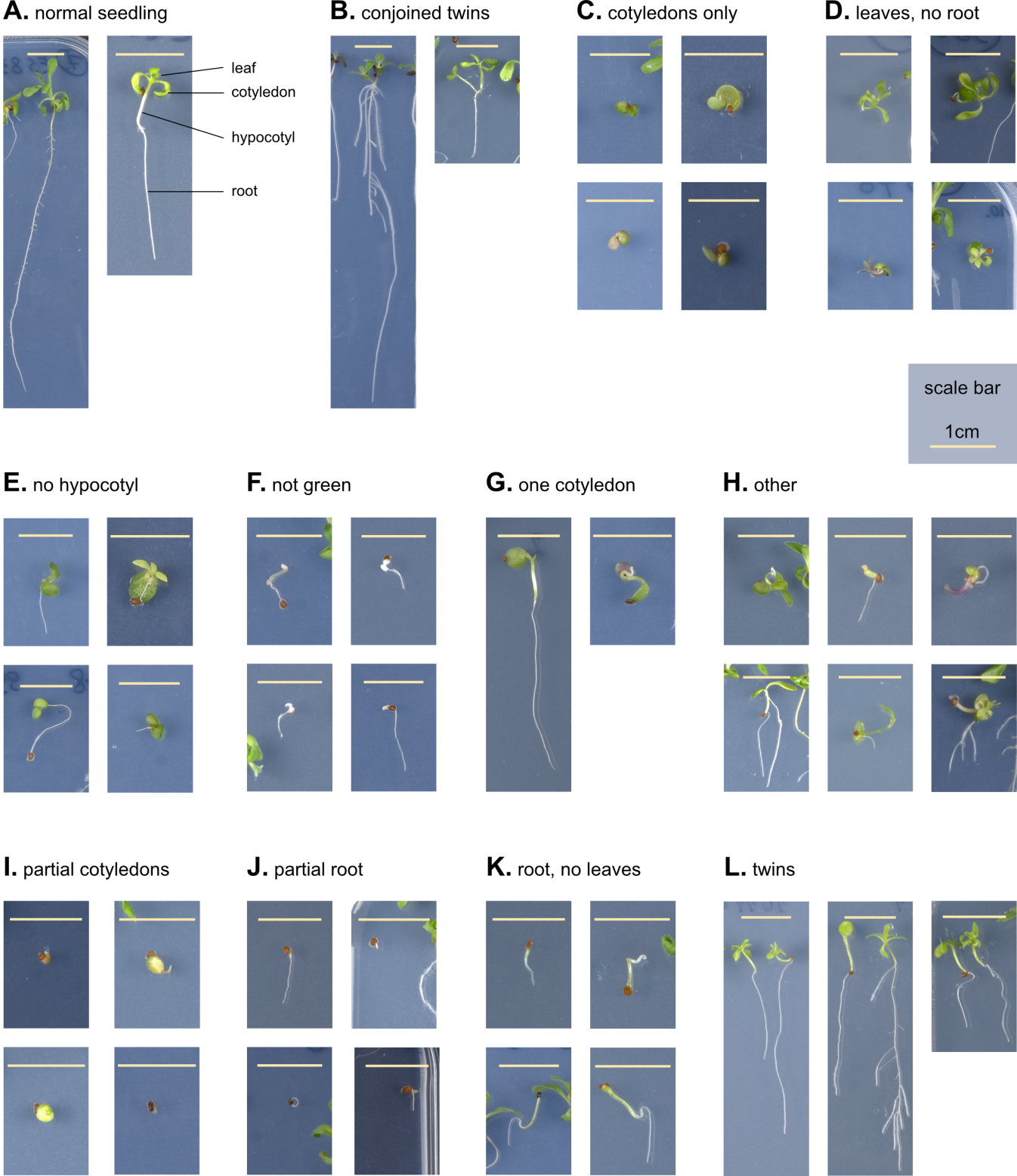


**Supplementary figure 6.** Normal seedling development (**A**) and examples of developmental abnormalities (**B-L**).


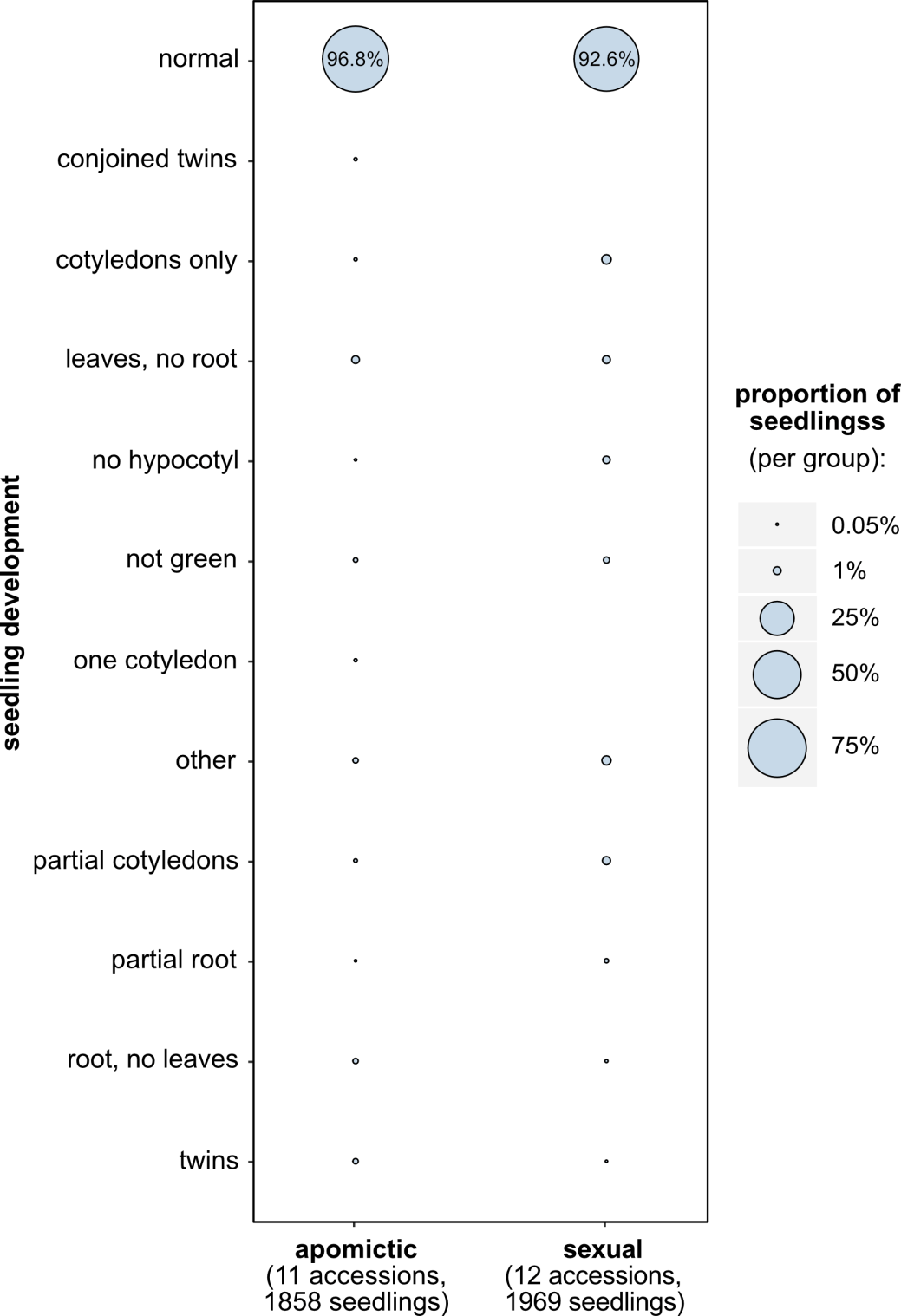


**Supplementary figure 7.** Proportions of normally-developed seedlings and different types of developmental abnormalities among apomictic and sexual accessions.

**
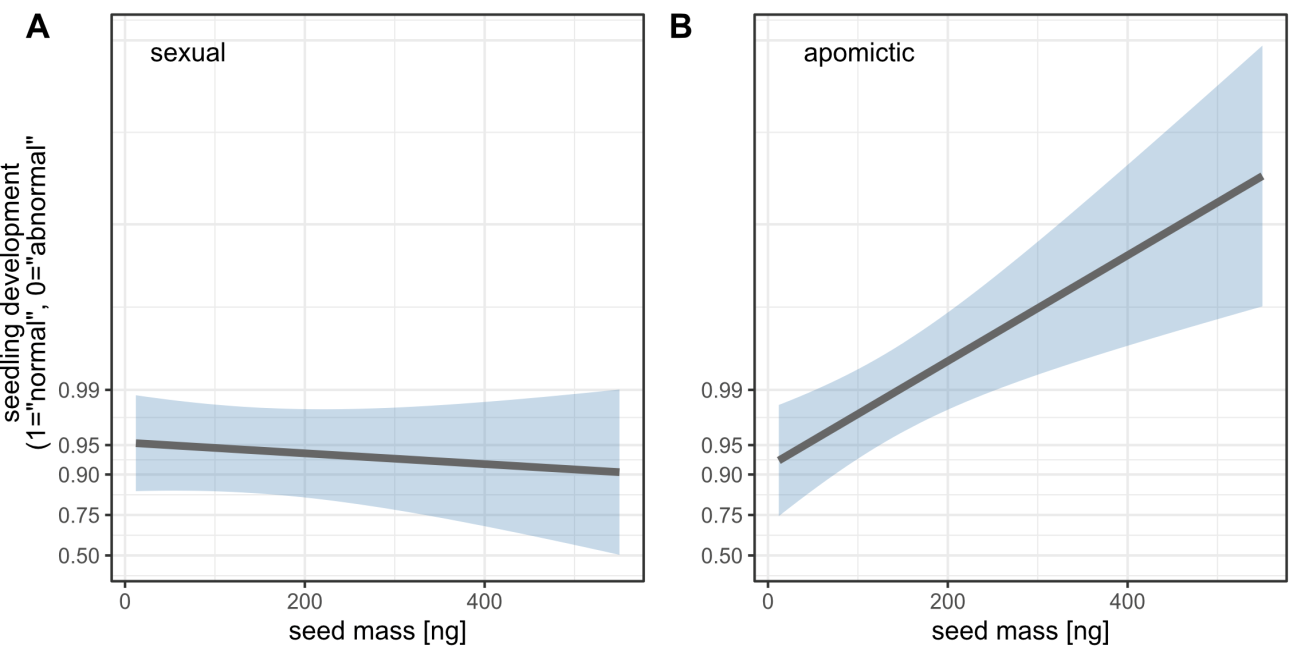
**

**Supplementary figure 8.** The effects of seed mass and reproductive mode on seedling development in sexual **(A)** and apomictic **(B)** accessions. Seedling development is plotted on a logit scale. Shaded area represents 95% confidence intervals.


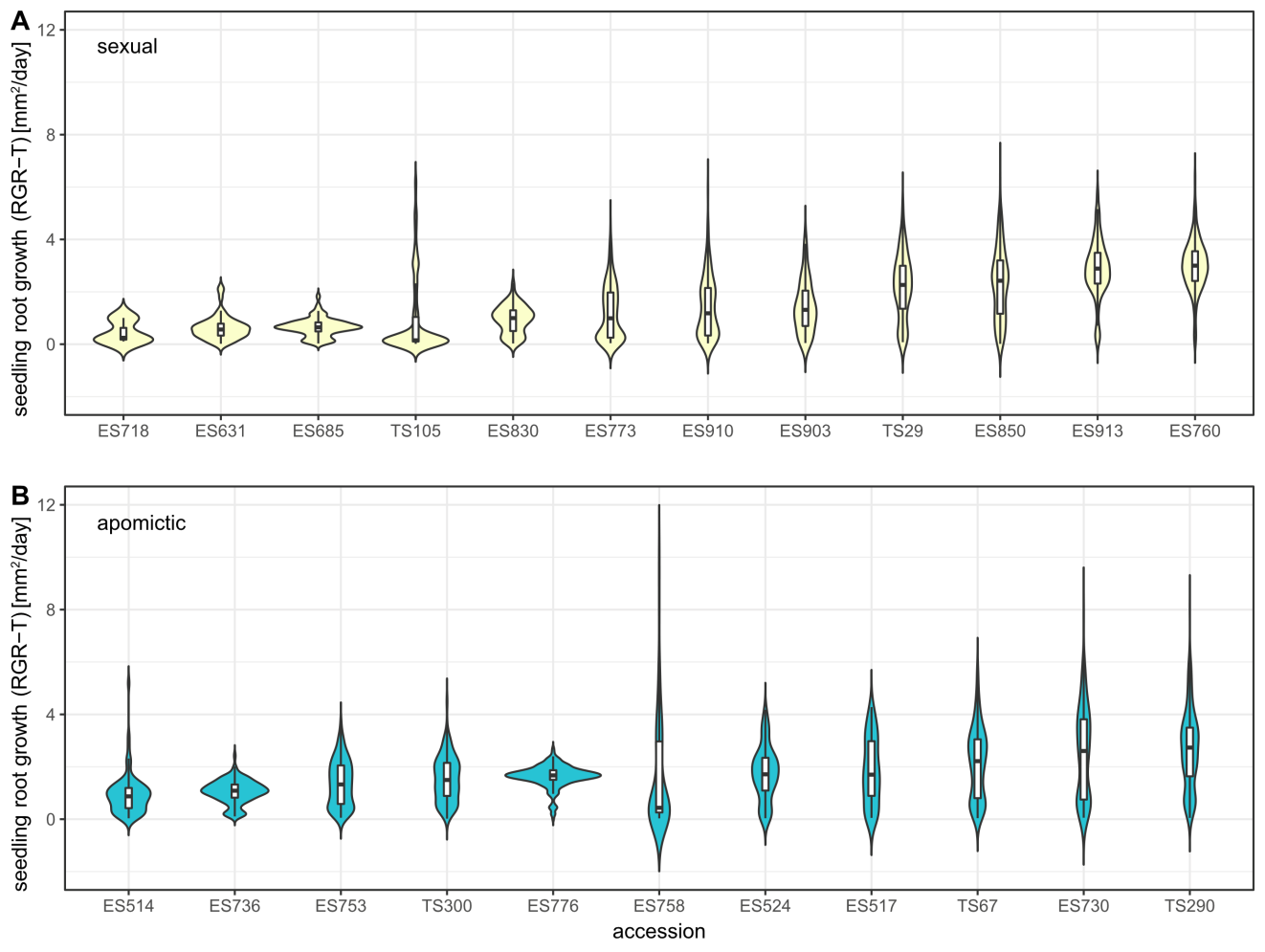


**Supplementary figure 9.** Violin plots with overlaid boxplots of total seedling root growth (RGR-T) in sexual (A) and apomictic (B) accessions. Accessions are ordered by mean RGR-T within each group.


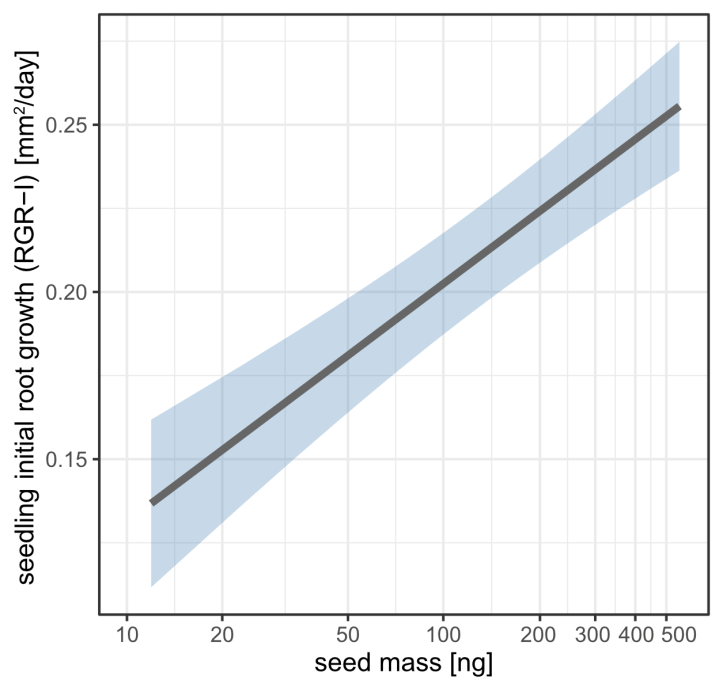


**Supplementary figure 10.** The effect of seed mass on initial root growth rate (RGR-I). Seed mass is plotted on log scale. Shaded area represents 95% confidence intervals.


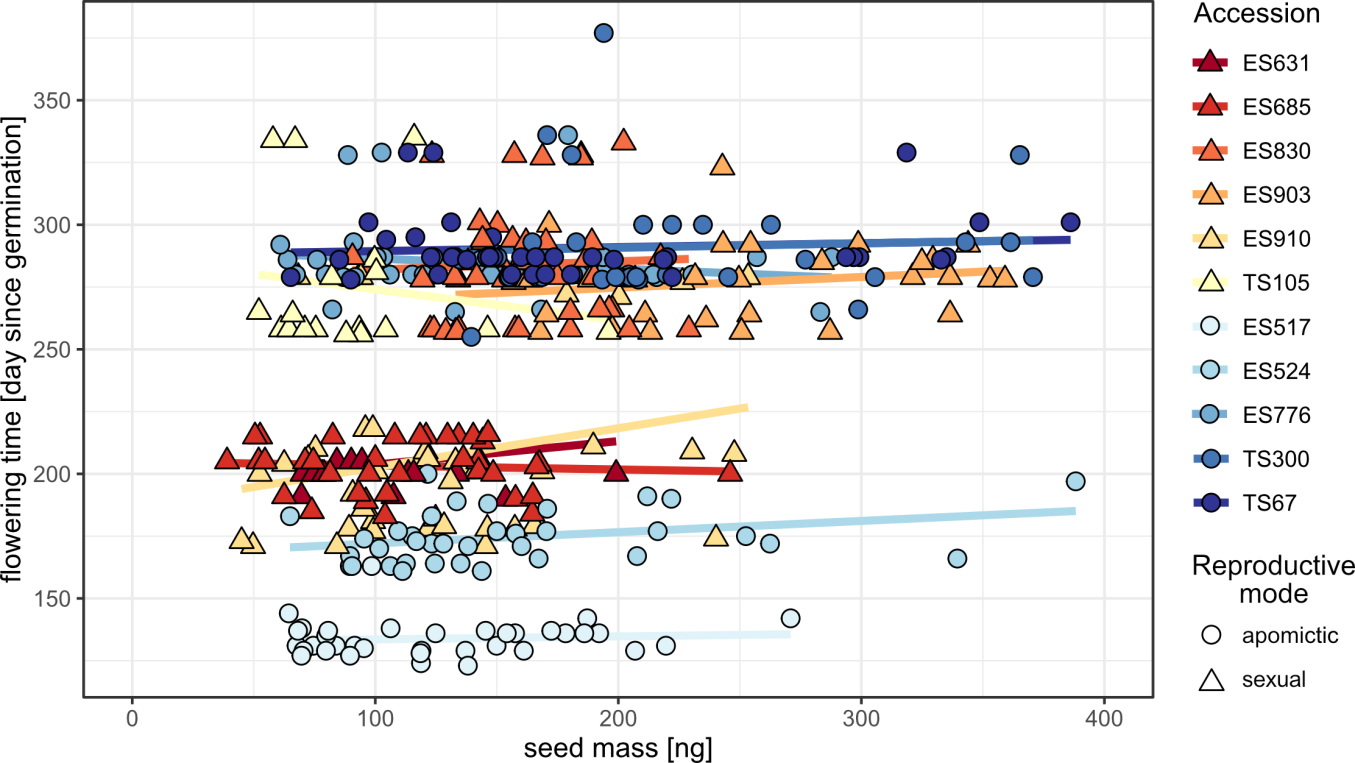


**Supplementary figure 11.** The relationship between initial seed mass and flowering time in 11 Boechera accessions.
